# Factors correlated with pain after total knee arthroplasty: A systematic review and meta-analysis

**DOI:** 10.1371/journal.pone.0283446

**Published:** 2023-03-24

**Authors:** Unni Olsen, Maren Falch Lindberg, Christopher Rose, Eva Denison, Caryl Gay, Arild Aamodt, Jens Ivar Brox, Øystein Skare, Ove Furnes, Kathryn A. Lee, Anners Lerdal

**Affiliations:** 1 Department of Public Health Science, Institute of Health and Society, Faculty of Medicine, University of Oslo, Oslo, Norway; 2 Department of Orthopaedic Surgery, Lovisenberg Diaconal Hospital, Oslo, Norway; 3 Division for Health Services, Norwegian Institute of Public Health, Oslo, Norway; 4 Center for Epidemic Interventions Research, Norwegian Institute of Public Health, Oslo, Norway; 5 Department of Family Health Care Nursing, University of California, San Francisco, San Francisco, CA, United States of America; 6 Research Department, Lovisenberg Diaconal Hospital, Oslo, Norway; 7 Department of Physical Medicine and Rehabilitation, Oslo University Hospital, Oslo, Norway; 8 Institute of Clinical Medicine, Faculty of Medicine, University of Oslo, Oslo, Norway; 9 The Norwegian Arthroplasty Register, Department of Orthopaedic Surgery, Haukeland University Hospital, Bergen, Norway; 10 Department of Clinical Medicine, University of Bergen, Bergen, Norway; 11 Department of Interdisciplinary Health Sciences, Institute of Health and Society, Faculty of Medicine, University of Oslo, Oslo, Norway; Baqai Medical University, PAKISTAN

## Abstract

**Main objective:**

Systematically review and synthesize preoperative and intraoperative factors associated with pain after total knee arthroplasty (TKA) in patients with osteoarthritis.

**Methods:**

Based on a peer-reviewed protocol, we searched Medline, Embase, CINAHL, Cochrane Library, and PEDro for prospective observational studies (January 2000 to February 2023) investigating factors associated with pain after TKA. The primary outcome was pain twelve months after TKA. Pain at three and six months were secondary outcomes. Multivariate random-effects meta-analyses were used to estimate mean correlation (95% CIs) between factors and pain. Sensitivity analysis was performed for each risk of bias domain and certainty of evidence was assessed.

**Results:**

Of 13,640 studies, 29 reports of 10,360 patients and 61 factors were analysed. The mean correlation between preoperative factors and more severe pain at twelve months was estimated to be 0.36 (95% CI, 0.24, 0.47; P < .000; moderate-certainty evidence) for more catastrophizing, 0.15 (95% CI; 0.08, 0.23; P < .001; moderate-certainty evidence) for more symptomatic joints, 0.13 (95% CI, 0.06, 0.19; P < .001; very low-certainty evidence) for more preoperative pain. Mean correlation between more severe radiographic osteoarthritis and less pain was -0.15 (95% CI; -0.23, -0.08; P < .001; low-certainty evidence). In sensitivity analysis, the estimated correlation coefficient for pain catastrophizing factor increased to 0.38 (95% CI 0.04, 0.64). At six and three months, more severe preoperative pain was associated with more pain. Better preoperative mental health was associated with less pain at six months.

**Conclusion and relevance:**

More pain catastrophizing, more symptomatic joints and more pain preoperatively were correlated with more pain, while more severe osteoarthritis was correlated with less pain one year after TKA. More preoperative pain was correlated with more pain, and better mental health with less pain at six and three months. These findings should be further tested in predictive models to gain knowledge which may improve TKA outcomes.

## Introduction

Total knee arthroplasty (TKA) is one of the most common surgical procedures [1, 2], and is considered as an effective procedure in relieving pain and restore physical function in patients with end-stage osteoarthritis (OA). Although TKA surgery is effective for most, one in five patients may experience chronic postsurgical pain [3, 4]. Chronic postsurgical pain is typically defined as pain that develops after a surgical procedure and persists at least three months [5, 6]. Chronic postsurgical pain is associated with lower patient satisfaction and higher societal and health care expenses due to resource-intensive revision surgery and long-term recovery [4, 7–10].

A comprehensive understanding of factors associated with poor pain outcomes is imperative for the development of a prediction model needed to identify patients at higher risk for chronic postsurgical pain [11, 12]. Although numerous preoperative and intra-operative factors have been studied, synthesizing the available evidence has yielded contradictory findings, perhaps related to certainty of evidence, merging data from short- and long-term outcomes, or pooling estimates from prospective and retrospective study designs [13–21]. Some authors did not perform meta-analysis due to heterogeneity in design and methods [14, 22–24]. Thus, we aimed to build from previous reviews and synthesize current evidence between preoperative and intraoperative factors associated with pain twelve months (primary outcome) and three and six months (secondary outcomes) after TKA.

## Methods

We performed our systematic review and meta-analysis according to an a priori peer-reviewed protocol and a preprint [25, 26]. The study was registered in International Prospective Register of Systematic Reviews (PROSPERO; CRD42018079069) [26]. We followed Cochrane Handbook guidelines [27], and reported the study using the Preferred Reporting Items for Systematic Reviews and Meta-analysis (PRISMA) reporting guideline (S1 Checklist).

### Search strategy and data sources

Two researchers (UO, MFL) and research librarians developed the search strategy with input from the research team [25]. The research librarian performed a systematic search for publications in MEDLINE (Ovid), Embase (Ovid), Cumulative Index to Nursing and Allied Health Literature (CINAHL; EBSCO), Cochrane Library and Physiotherapy Evidence Database between January 1, 2000, and February 6, 2023. No language restrictions were set. References were imported to Endnote X8 Software version 20.2.1 (Clarivate Analytics).

### Eligibility criteria

We included peer-reviewed published studies that reported estimates of association between preoperative or intraoperative factors and pain at three, six and twelve months after TKA. Studies were eligible if participants were 18 years or older, diagnosed with osteoarthritis, and scheduled for primary TKA. Eligible study designs were prospective longitudinal observational studies and randomized clinical trials that provided estimates of association. Conference abstracts, retrospective studies, case-control studies, studies of uni-compartmental surgery and studies that lacked clear pain outcome measures were not eligible. Studies that merged data from mixed patient populations or did not report separate data for the osteoarthritis or TKA population were excluded

### Outcomes

The primary outcome was pain at twelve months following TKA. Secondary outcomes were pain at three and six months.

### Study selection and data extraction

We used a standardized data extraction form customized to the research question as explained in the published protocol [25] which included study design, country, participant characteristics, sample size, measures and outcomes, statistical analyses, and estimates of association. Two reviewers (UO, MFL) independently screened titles and abstracts for relevance, assessed full-text publications against eligibility criteria and assessed risk of bias. Disagreements were resolved by consensus or by consulting a third author (ED).

### Methodological quality

The Quality in Prognosis Studies (QUIPS) tool [28] was used to systematically evaluate risk of bias in the retrieved studies according to the protocol [25]. The six QUIPS domains include study participation, study attrition, prognostic factor measurement, outcome measurement, confounding, and statistical analysis and reporting [27].

### Certainty of evidence

We assessed certainty of evidence using the Grading of Recommendations, Assessment, Development and Evaluation (GRADE) framework [29]. Two researchers (UO and MFL) judged certainty of evidence, with a third researcher involved in discussing cases of disagreement (ED). GRADEpro GDT (McMaster University) was used to manage and summarize the evidence.

### Statistical analysis

We synthesised results from all included studies at three, six, and twelve months post-surgery according to our pre-specified protocol [25], with the exception that we used a multivariate random-effects meta-analysis that accounts for the sparse data (many factors relative to the number of studies), as in our recent review of factors for post-surgical function [30]. Further protocol deviations are noted below, in the discussion and in the Methods in the Supplement.

The included studies reported associations as odds ratios (ORs), risk ratios (RRs), linear model coefficients (including differences), or correlations using discrete or continuous scales to measure factors and outcomes. Correlation coefficients were meta-analyzed on the arctangent scale [31], and estimates were back-transformed to the correlation scale for reporting.

We expected within-study correlation and between-study heterogeneity and therefore used a multivariate random-effects model to estimate mean rather than common correlations between factors and pain.

Heterogeneity was quantified by using *I*^2^ statistics. P scores were calculated to evaluate the certainty that the mean correlation for each factor is larger in magnitude than the mean correlations for all other factors [32]. We also explored how estimates may depend on the choice of model: we removed factors supported by few studies (to decrease the impact of sparsity) and compared estimates from the two multivariate models and univariate meta-analyses for consistency. We then performed sensitivity analyses on pain at twelve months, and excluded studies judged to have high risk of bias for each QUIPS domain and re-ran the multivariate meta-analysis (S4 Appendix).

Statistical analyses were performed using Stata 16 (StataCorp LLC, College Station, Texas, USA). Mean correlations with 95% confidence intervals (CIs) are reported. Hypothesis testing was not predefined, but 2-sided P values are reported for completeness.

## Results

**Fig 1:**
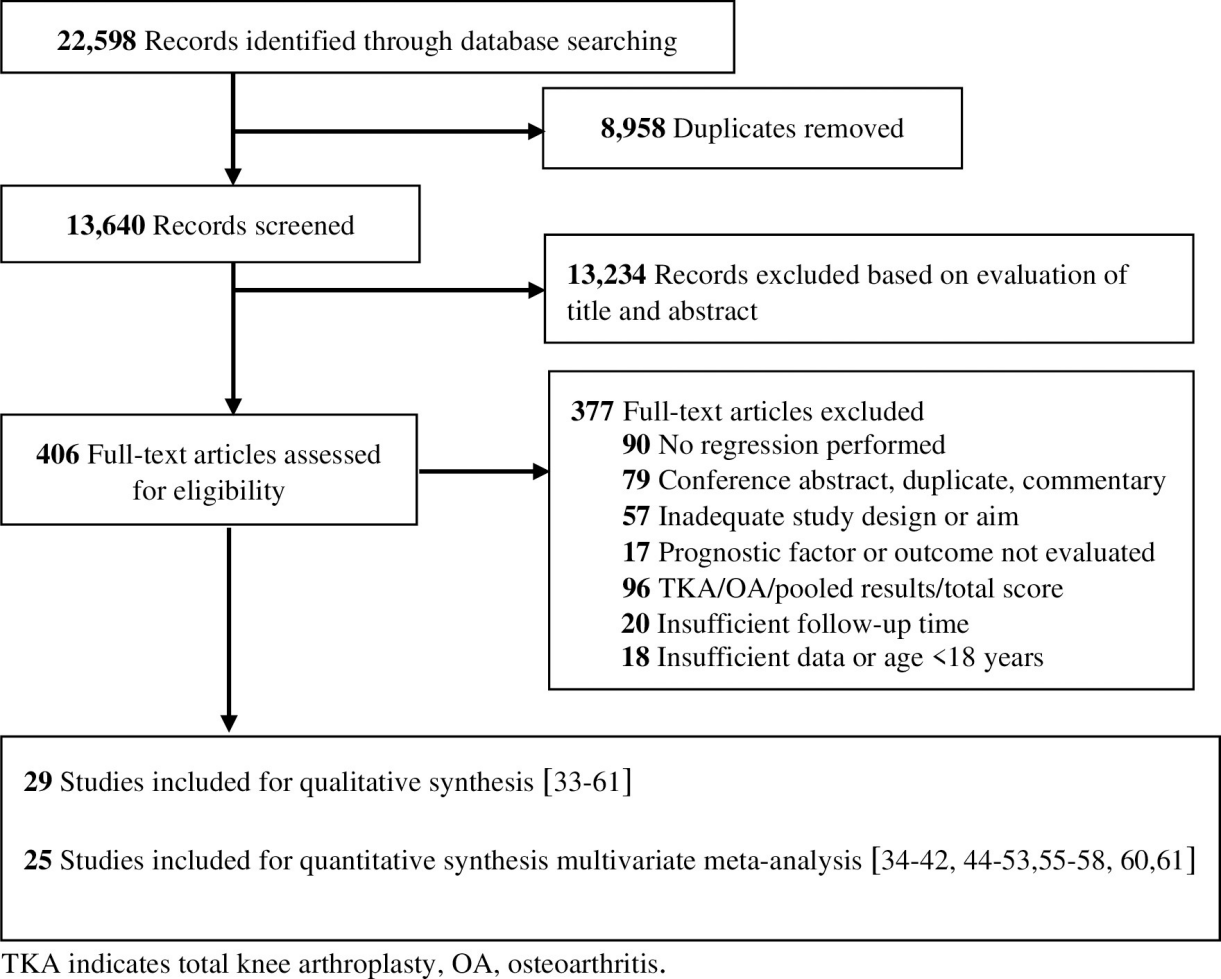
Flow chart of included studies.

The search yielded 13,640 studies. After title and abstract screening, 406 studies were assessed in full text and 374 were ineligible, leaving 29 studies [33–61] with a total sample of 10,360 patients (Fig 1). Sample sizes ranged from 26 [43] to 5309 [50]. We excluded eight studies from analysis because attempts to obtain missing data from authors were unsuccessful or insufficient [62–69]. The search strategy, subject headings and keywords customized for all databases is presented in S8 Appendix and reasons for study exclusion are in S9 Appendix.

In all, 61 preoperative and intraoperative factors were identified in the 29 studies [33–61]. All studies used prospective longitudinal observational designs, and most were single-center studies [33, 36–45, 48–51, 54, 55, 58–61] and conducted in European countries [33, 37, 39–48, 52, 57, 61]. No randomized trial met inclusion criteria. Mean age ranged from 63 [40] to 73 years [48], and the percentages of women in the samples varied from 49% [58] to 95% [40]. As shown in the Table 1, most studies used the Western Ontario and McMaster Universities Arthritis Index (WOMAC) to measure pain [34, 35, 37, 38, 47, 49, 51, 52, 58, 61].

**Table 1:** Characteristics of reviewed studies.

Study, country	Country	Design	Patients analyzed, No.	Data collection	Follow-up, mo	Baseline Age, y	Patients, No./Total No (%) Female Male		Analysis	Factors measured	Outcome measure
Cremeans-Smith et al, 2016^a^ [49]	United States	PC	101	NA	3	Mean, 69	75/110 (68)	35/110 (32)	Hierarchical linear regression	Education (level), pain (WOMAC), Cortisol (level), anaesthesia type (general vs spinal)	WOMAC
Lindner et al, 2018 [61]	Germany	PC	61	NA	3	Mean, 67	37/61 (61)	24/61 (39)	Stepwise multiple linear regression	Pain (WOMAC)	WOMAC
Lingard et al, 2007 [35]	UK, US, Canada, Australia	PC	676	1997–1999	3	Distress: median, 70 Non-distress: median, 71	574/676 (85)	102/676 (15)	Repeated measures	Psychological distress (SF-36)	WOMAC
Luo et al,2019 [59]	PC	PC	471	2017–2018	3	Mean, 64	357/471 (76)	114/471 (24)	Pearson correlation	Sleep dysfunction (PSQI), daytime sleepiness (ESS), sleep quality (self-developed scale)	KSS
Perruccio et al,2019 [60]	Canada	PC	477	2014–2016	3	Mean, 65	279/477 (58)	198/477 (42)	Linear regression	Age (y), sex (men/women), BMI, comorbidity (AAOs comorbidity Scale), symptomatic joint count, pain (KOOS), low back pain (yes/no), depression (HADS)	KOOS
Attal et al, 2012^a^ [33]	France	PC	81	2008–2011	6	Mean, 69	58/89 (65)	31/89 (35)	Stepwise logistic regression	Trail Making Time (TMT-B time)	Brief Pain Inventory (BPI)
Bossman et al, 2017 [52]	Germany	PC	47	NA	6	Mean, 69	37/56 (66)	19/56 (34)	Analysis of variance (bootstrap)	Age (y), sex (men/women), BMI, pain (WOMAC), conditioned pain modulation (pressure pain algometry), heart rate variability (SDNN), temporal summation (pin-prick stimulator), pain catastrophizing (PCS), Sympathetic/ parasympathetic activity (LogLF)	WOMAC
Bruehl et al, 2023 [54]	US	PC	91	NR	6	Mean, 67	57 (63)	34 (37)	Generalized linear density ratio model	Ischemia duration (blood sample), oxidative stress (blood sample)	MPQ-2
Bugada et al, 2017 [57]	Italy	PC	563	2012–2015	6	Median, 72	421/606 (69)	185/606 (31)	Logistic regression	Age (y),	NRS
Chen et al, 2021 [55]	China	PC	220	2019–2020	6	Pain ≥4: median, 70 Pain <4: median,71	102/220 (46)	118/220 (54)	Logistic regression	Age (y), serum angiotensin II Type 2 receptor (AT_2_R), temporal summation (PD-Q), Anxiety and depression (HADS), disability (WOMAC). pain expectation (NRS), pain sites (count)	VAS
Edwards et al, 2022 [56]	US	PC	248	NA	6	Mean, 65	147 (59.5)	101 (40.5)	Backwards selection regression	Pain (BPI), State catastrophizing (PCS), catastrophizing (PCS), opioid use, sleep efficiency (PSQI), other chronic pain sites (count), painful areas (count), anxiety (PROMIS), agreeableness (NEO Inventory)	BPI
Engel et al, 2014 [58]	US	Case-control	54	NA	6	Mean, 68	36/74 (49)	38/74 (51)	Multiple hierarchical regression	Arthritis helplessness (AHI), coping efficacy (scale)	WOMAC
Escobar et al, 2007 [47]	Spain	PC	640	1999–2000	6	Mean, 72	473/640 (74)	167/640 (26)	General linear model	Age (y), sex (men/women), social support (yes/no), comorbidity (CCI), pain (WOMAC), low back pain (yes/no), mental health (SF-36)	WOMAC
Fitz-simmons et al, 2018 [53]	Canada	PC	74	2014	6	Mean, 65	67/99 (68)	32/99 (32)	Multiple linear regression	Suspected neuropathic pain (SNEP), Preoperative pain (ICOAP), Pain catastrophizing (PCS), depression (PHQ, comorbidity (count)	ICOAP
Pua et al, 2019 [50]	Singapore	PC	4026	2013–2017	6	Mean, 68	3003/4026 (75)	1023/4026 (25)	Proportion-al odds regression	Age (y), Sex (Men/women), BMI, education (primary, secondary, tertiary), ethnicity (Chinese, Indian, Malay, other), social support (yes/no), comorbidities (yes/no), contralateral knee pain (KSS), pain (OKQ), Knee extension and flexion (goniometer), physical function (categories), depression (SF-36)	OKQ
Yang et al, 2019 [51]	US	PC	107	2010–2011	6	Mean, 65	55/107 (51)	52/107 (49)	Multiple logistic regression	Mental health (SF-36), Pain catastrophizing (PCS), use device (yes/no)	WOMAC
Attal et al, 2012a [33]	France	PC	69	2008–2011	12	Mean, 69	58/89 (65)	31/89 (35)	Stepwise logistic regression	Recall (ROCF)	BPI
Dave et al, 2017 [34]	United States	PC	241	2012–2014	12	Mean, 67	146/241 (61)	95/241 (39)	Poisson regression	Painful body regions (count), pain (WOMAC), pain catastrophizing (PCS)	WOMAC
Dowsey et al, 2012 [36]	Australia	PC	473	2006–2007	12	Mean, 71	331/473 (70)	142/473 (30)	Multivariate linear regression	Age (y), sex (men/women), BMI, comorbidity (CCI), pain (IKSS), physical function (IKSS), mental health (SF-12), Osteoarthritis severity (K-L grade), cruciate retaining, patella resurface	IKSS
Getachew et al, 2020 [39]	Norway	PC	185	2012–2014	12	Mean, 68	137/202 (68)	65/202 (32)	Multiple logistic regression	Age (y), Sex (men/women), Pain (NRS), fatigue (LFS) Sleep quality (PSQI), depression (HAD)	BPI
Giordiano et al, 2020 [41]	Denmark	PC	136	NR	12	High pain relief: mean, 69 Low pain relief: mean, 68	82/136 (60)	54/136 (40)	Linear regression	Pain (VAS), circulating micromiRna-146a-5p (venous blood)	VAS
Hardy et al, 2022 [48]	France	PC	103	2014–2015	12	Mean, 73	67/36	65/35	Logistic regression	Catastrophizing (PCS)	VAS
Kornilov et al, 2018 [40]	Russia	PC	79	2014	12	Mean, 63	75/79 (95)	4/79 (5)	Logistic regression	Pain (BPI), physical activity (HUNT 2 physical activity score)	BPI
Lingard et al,2007a [35]	UK, US, Canada, Australia	PC	676	1997–1999	12	Distress: median, 70 Non-distress: median, 71	574/676 (85)	102/676 (15)	Repeated measures	Psychological distress (SF-36)	WOMAC
Petersen et al, 2015 [42]	Denmark	PC	78	NA	12	Low pain: mean, 68 High pain group: mean, 72	50/78 (59)	28/78 (41)	Multi-variate logistic regression	Pain (VAS), temporal summation (von Frey stimulator)	VAS
Petersen et al, 2017 [44]	Denmark	PC	130	NA	12	Chronic pain: mean, 69 Normal recovery: mean, 68	Chronic pain: 14/19 (74) Normal recovery: 59/105 (56)	Chronic pain: 5/19 (26) Normal recovery: 46/105 (44)	Linear regression	Temporal summation (von Frey stimulator), K-L grade, warm detection-/heat pain threshold	VAS
Petersen et al, 2020 [43]	Denmark	PC	26	2011–2012	12	High pain: Mean, 64 Low pain: mean, 70	14/26 (54)	12/26 (46)	Pearson correlation	Synovial membrane thickness (CE-MRI), degree perfusion (voxels*ME), volume perfusion (IRE), synovitis severity	VAS
Tilbury et al, 2018 [45]	Netherlands	PC	146	2011–2012	12	Mean, 67	101/146 (69)	87/146 (31)	Multi-variate linear regression	BMI, mental health (SF-36), outcome expectancies (HSS)	KOOS
Sullivan et al, 2011 [38]	Canada	PC	120	NA	12	67 (mean)	73/120 (61)	47/120 (39)	Multiple regression	Age (y), sex (men/women), BMI, comorbidity (CCI), pain (WOMAC), pain catastrophizing (PCS), depression (PHQ-9), kinesophobia (TSK), surgery duration (minutes)	WOMAC
Van de Water et al, 2019 [46]	Netherlands	PC	559	2012–2015	12	Mean, 67	378/559 (68)	181/559 (32)	Multi-variate linear regression	Pain (KOOS), K-L grade	KOOS
Wylde et al, 2012 [37]	United Kingdom	PC	220	NA	12	Median, 70	136/220 (62)	84/220 (38)	Ordinary least squares regression	Age (y), sex (men/women), comorbidity (SCQ), pain (WOMAC), depression (HADS), anxiety (HADS), pain-self efficacy (PSEQ)	WOMAC

AAOS comorbidity Scale, American Academy of Orthopaedic Surgeons comorbidity scale; AHI, Arthritis Helplessness Index; AT_2_R, Angiotensin Type 2 receptor; BMI, Body Mass Index; BPI, Brief Pain Inventory; CCI, Charlson Comorbidity Index; CE-MRI, contrast-enhanced magnetic resonance imaging; ESS, Epworth; Sleepiness Scale; HADS, Hospital Anxiety and Depression Scale; HSS, Hospital for Special Surgery; HUNT 2, The Trøndelag Health Study 2; ICOAP, Intermittent and Constant Osteoarthritis Pain; IKSS, International Knee Society Score; IRE, Initial Rate of Enhancement; K-L Grade, Kellgren Lawrence Grade; KOOS, Knee Injury and Osteoarthritis Outcome Score; KSS, Knee Society Rating System; LFS, Lee Fatigue Scale; LogLF; Low-Frequency Power (log-transformed); ME, Maximum Enhancement; MPQ-2, short Form-McGill Pain Questionnaire-2; NA, not applicable; Neo Inventory, NEO Personality Inventory; NRS, Numerical Rating Scale; PCS, Pain catastrophizing Scale; PROMIS, Patient-Reported Outcomes Measurement Information System (PROMIS); OKQ, Oxford Knee Questionnaire; PC, prospective cohort; PCS, Pain Catastrophizing Scale; PD-Q, Pain Detect Questionnaire; PHQ, Patient Health Questionnaire; PSEQ, Pain Self-Efficacy Scale; PSQI, Pittsburgh Sleep Quality Index; ROCF, Rey Osterreich Complex Figure; SCQ, Self-Administered Comorbidity questionnaire; SDNN, standard deviation RR-intervals; SF-12, 12-Item Short-Form Health Survey; SF-36, 36-Item, Short Form Health Survey; SNEP, Self-Leeds Assessment of Neuropathic Symptoms and Signs; TMT-B time, Trail Making Time; TSK, Tampa Scale of Kinesophobia; TUG, Timed Up and Go; VAS, Visual Analogue Scale; WOMAC, Western Ontario and McMaster Universities Osteoarthritis Index

^a^ Study with 2 follow-up time

**Fig 2:**
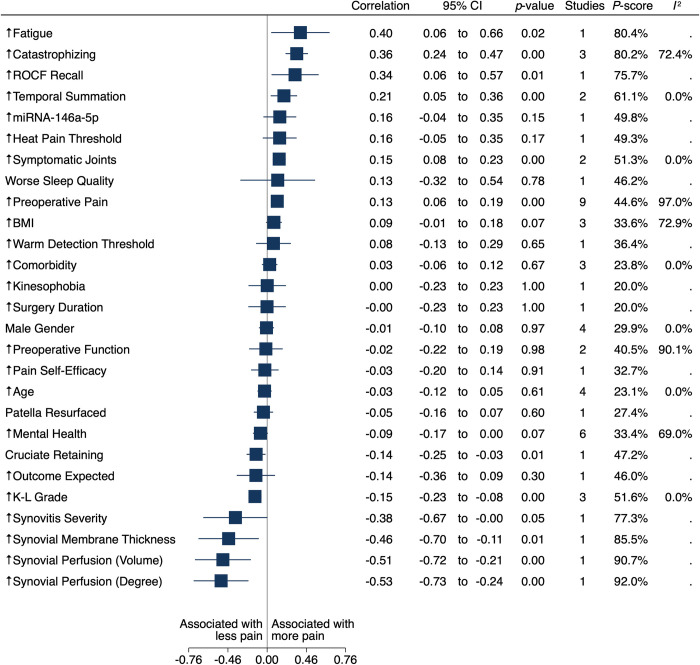
Forest plot of factors associated with pain at twelve months.

**Fig 3:**
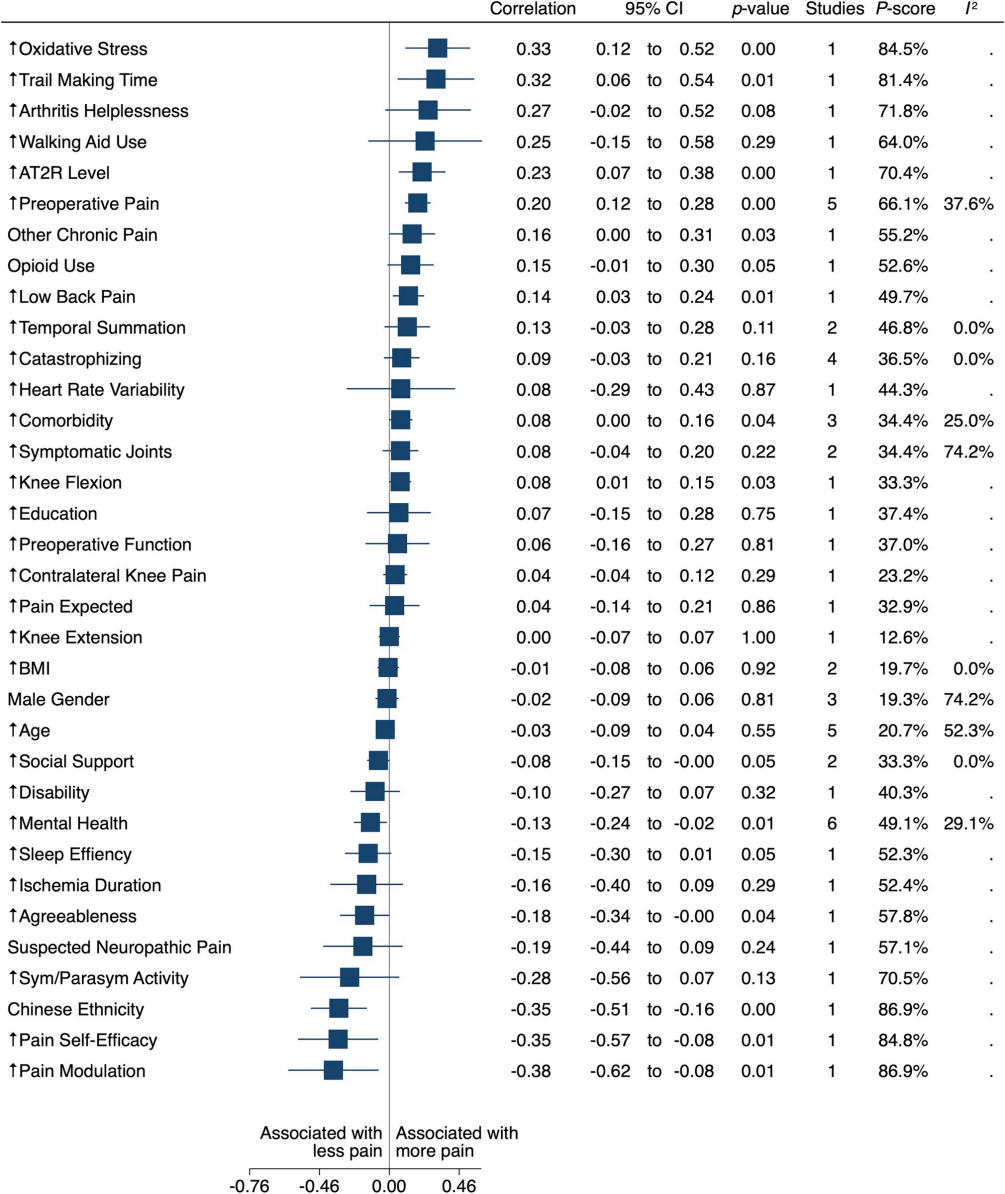
Forest plot of factors associated with pain at six months.

**Fig 4:**
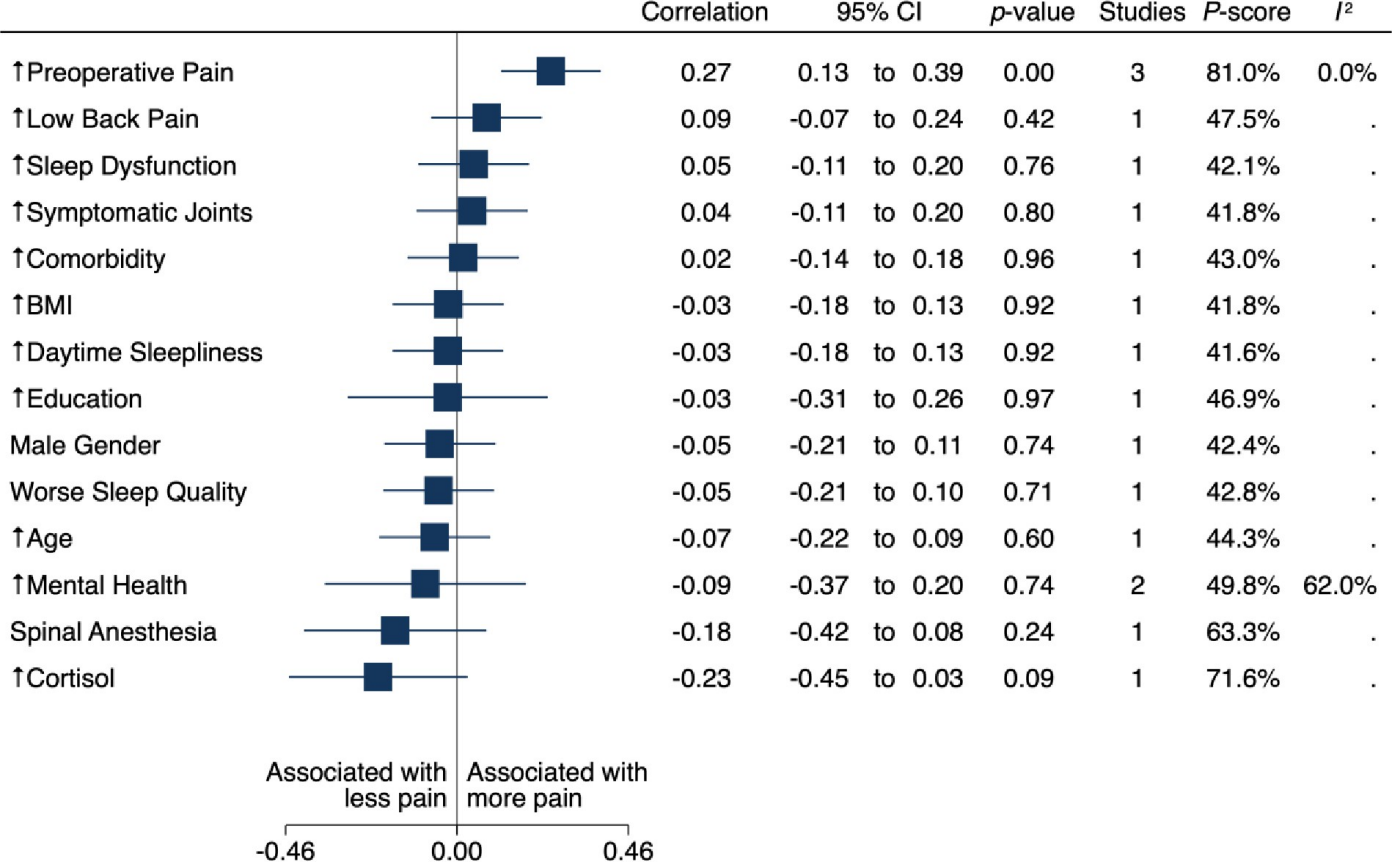
Forest plot of factors associated with pain at three months.

We present separate estimates of mean correlations between preoperative and intraoperative factors and the three-, six- and twelve- month pain outcomes in multivariate meta-analysis (Figs 2–4). Multivariate meta-analytical estimates of correlation at each postoperative follow-up time are shown in Fig 2 and S1 Appendix. Descriptions of potential inconsistencies at three, six and twelve months are in S2 Appendix, and univariate meta-analyses for associations between individual factors and the outcomes are in S4 Appendix. Results from sensitivity analysis are presented in S1 Appendix. We provide a full glossary of labels for included factors in the Table in S5 Appendix. We report all estimates between preoperative and intraoperative factors and pain during the year (three, six and twelve months) after TKA as mean correlations, with positive correlations indicating more postoperative pain.

A total of 15 studies with 3,241 participants [33–46, 48] reported estimates for 34 factors correlated with pain twelve months after TKA (Fig 2). The two most common factors were preoperative pain [34, 36–42, 46] reported in nine studies and mental health (including anxiety, depression, psychological distress) reported in six studies [35–39, 45]. Most of these studies were judged as having high risk of bias on one or more domain (S6 Appendix).

Mean correlation between preoperative pain catastrophizing and pain twelve months after TKA was estimated to be 0.36 (95% CI, 0.24 to 0.47; P < .001; P score = 80.2%; three studies [34, 38, 48]; moderate-certainty evidence and substantial heterogeneity among reported estimates of association [I^2^ = 72.4%], while mean correlation for more temporal summation was estimated as 0.21 (95% CI, 0.05 to 0.36; P < .000; P score = 61.1%; two studies [42, 44]; very low-certainty evidence and heterogeneity among reported estimates of association might not be important [I^2^ = 0%]), more symptomatic joints was estimated to be 0.15 (95% CI, 0.08 to 0.23; P < .001; P score = 51.3%; two studies [34, 37]; moderate-certainty evidence and heterogeneity among reported estimates of association might not be important [I^2^ = 0%]), and more preoperative pain was estimated to be 0.13 (95% CI, 0.06 to 0.19; P < .001; P score = 44.6%; nine studies [34, 36–42, 46]; very low-certainty evidence and considerable heterogeneity among reported estimates of association [I^2^ = 97.0%]).

In contrast, mean correlation for more severe osteoarthritis and pain at twelve months was negative. The estimated correlation was -0.15 (95% CI, -0.23 to -0.08; P < .001; P score = 51.6%; three studies [36, 44, 46]; low-certainty evidence and heterogeneity among reported estimates of association might not be important [I^2^ = 0%]),

Results from the prespecified sensitivity analysis (S4 Appendix), estimated a mean correlation of 0.38 (95% CI, 0.04 to 0.64) between pain catastrophizing and more pain, compared to 0.28 (95% CI, 0.11 to 0.43) when including all studies. The mean correlation estimate was 0.15 (95% CI 0.06 to 0.24) for symptomatic joints compared to 0.15 (95% CI 0.07 to 0.23) when including all studies. The mean correlation estimate was 0.16 (95% CI -0.00 to 0.25) for level of pain compared to 0.13 (95% CI 0.06 to 0.19) when including all studies. Mean correlation estimate was -0.15 (95% CI -0.24 to -0.06) for more severe osteoarthritis compared to -0.15 (95% CI -0.23 to -0.08) when including all studies. The association for temporal summation identified in the multivariate meta-analysis was obscured in the sensitivity analysis as the statistical analysis domain was judged high risk of bias.

There was 11 studies with 6,078 participants that included estimates for 34 potential factors associated with pain six months after TKA (Fig 3) [33, 47, 50–58]. Mean correlation with preoperative pain was 0.20 (95% CI 0.12 to 0.28; P < .000; P score = 66.1%; five studies [47, 50, 52, 53, 56]; low-certainty evidence and heterogeneity among reported estimates of association may not be important [I^2^ = 37.6%]). Mean correlation with better mental health was -0.13 (95% CI -0.24 to -0.02; P = 0.01; P score = 49.1%; six studies [47, 50, 52, 53, 56]; moderate-certainty evidence and heterogeneity among reported estimates of association may not be important [I^2^ = 29.1%]).

For the other secondary outcome, pain three months after TKA, five studies with 1786 patients provided pain outcome data at three months after TKA for 14 potential factors (Fig 4) [35, 49, 59–61]; Mean correlation with preoperative pain was 0.27 (95% CI 0.13 to 0.39; p < .001; P score = 81.0%; three studies [49, 60, 61]; low-certainty evidence and heterogeneity among reported estimates of association may not be important [I^2^ = 0%]).

Meta-analytical estimates for the other factors do not exclude the possibility of no correlation with pain at three, six, and twelve months. It is plausible that these factors are uncorrelated with pain, but also possible that important correlations exist but cannot be estimated with much precision.

We compared meta-analytic estimates from three models and there was reasonable consistency between the univariate and multivariate meta-analysis for all factors with respect to direction of association (S2 Appendix).

Decisions regarding risk of bias for each QUIPS domain are shown in S4 Fig in S1 Appendix. We judged the included studies to be generally low risk of bias for prognostic factor measurement (n = 16) and outcome measurement (n = 21). In contrast, some studies were judged high risk of bias for study participation (n = 12), study attrition (n = 16), and statistical analysis (n = 13).

Full details of our certainty of evidence (GRADE) judgements are provided in S7 Appendix. Risk of bias and imprecision were the most common reasons for downgrading the certainty of evidence.

## Discussion

To our knowledge this is the first systematic review and meta-analysis examining factors correlated with pain at three, six and twelve months after TKA that also evaluated certainty of evidence. For the primary outcome at twelve months and based on at total sample of 3,241 patients, we estimated that pain catastrophizing, more symptomatic joints, and higher level of preoperative pain were correlated with worse pain outcomes, while more severe radiographic osteoarthritis were correlated with better pain outcome twelve months later. Our findings suggest that more severe preoperative pain is correlated with worse pain outcomes and that better mental health is associated with better pain outcomes at three and six months. It is worth noting that our findings do not indicate that the individual patient with a poor risk profile will experience chronic postsurgical pain if they undergo TKA surgery. Findings simply suggest that the identified factors were correlated with less or worse pain in an absolute sense. Thus, our results should be interpreted accordingly.

We estimated moderate-certainty evidence that pain catastrophizing is correlated with worse pain outcomes at twelve months. The correlation was larger in sensitivity analysis where we removed a study with high risk of bias. Our findings are similar to results from prior systematic reviews or meta-analyses [18, 22, 70]. However, our study differs in two critical ways: our results are entirely based on prospective studies, and we did not pool results from studies with short-term and longer-term follow-up. Efficacy for cognitive behavioral therapy to enhance skills for coping with pain remains unknown [71, 72], and still TKA surgery may be the most effective intervention, giving more pain relief, than non-operative treatment.

We found moderate-certainty evidence that a higher number of symptomatic joints was associated with more pain twelve months after TKA, with equal correlation in the sensitivity analysis. This result is supported by findings from a previous univariate meta-analysis that identified multiple painful sites as a factor influencing the pain outcome [18] but the association was not significant in the multivariate meta-analysis. Degenerated cartilage and subchondral bone are removed during surgery; however, pain may also be generated from other structures or tissue surrounding the knee, which might influence pain outcome.

We found positive correlations between more preoperative pain and pain severity at twelve months (very-low certainty evidence). Positive correlations were also identified for the secondary outcomes at three and six months (low-certainty evidence). Our findings are in consistency with other reviews and meta-analysis [13, 18]. There is emerging evidence that improvement in pain for most patients usually follows a steep trajectory in the first three to six postoperative months, before pain levels seems to plateau at twelve months [73–75]. Accordingly, we have added new evidence on preoperative factors correlated with adverse pain outcomes at three, six and twelve months after TKA. There were no intraoperative factors that correlated with pain outcomes at three, six or twelve months.

We found a negative correlation between severity of osteoarthritis and pain at twelve months, i.e., the more severe the osteoarthritis before surgery, the lower the pain severity twelve months later. Although the evidence was rated as low-certainty, the correlation persisted in the sensitivity analysis. Another meta-analysis has shown that patients with mild radiographic osteoarthritis reported more pain after TKA [16]. In contrast to our study, evidence was not graded and retrospective study designs with follow-up from one to six years were included. Results from our and their meta-analyses indicate that patients with severe osteoarthritis might gain more from TKA surgery than patients with less severe osteoarthritis. Non-operative treatment options should be considered to all patients with low-grade radiographic OA findings before surgery [76].

This study had many strengths, including up-to-date robust methods that followed Cochrane Handbook guidelines with descriptions in a pre-specified peer-reviewed protocol [25], a preprint [26], assessing risk of bias using QUIPS, and judging certainty of evidence using GRADE. We included only longitudinal prospective studies with associations reported at pre-defined time points in the first postoperative year and applied multivariate meta-analysis when the number of variables was large relative to number of studies [26].

There are some limitations that need to be addressed. First, we included studies that were largely heterogeneous for measurement of factors. Less heterogeneity existed in postoperative pain measures, with WOMAC being the most common. We used a number of exploratory statistics to estimate associations. Researchers either opt for narrow eligibility criteria and risk excluding potentially useful evidence, or wider eligibility criteria that require appropriate methods to address the heterogeneity [27]. We chose the latter, but results should be interpreted carefully due to underlying heterogeneity. Some included studies had large sample sizes that resulted in narrow CIs, and I^2^ for the pooled results tend to be very high and might be misleading [29]. Our estimates may also be biased by including several studies judged high risk of bias. To address this issue, we performed pre-specified sensitivity analyses excluding studies with high risk of bias for each QUIPS domain. We were unable to perform planned analyses of non-reporting bias and small study effects, or planned subgroup analyses, because the number of included studies did not meet our pre-specified criterion. We had also planned leave-one-study-out sensitivity analysis to explore the influence of each study on meta-analysis results, but this was not feasible. Many of the studies in our review had limitations that resulted in downgrading our certainty of the evidence. This does not necessarily indicate that those studies were of poor quality, but that important areas requiring documentation according to methodological standards were not reported. The importance of consistent reporting following these standards should be stressed so that evidence can be evaluated with high certainty. We suggest that researchers design studies using tools such as QUIPS to minimize risk of bias. We did not address the magnitude of change in pain score, which probably would be the most interesting for the patients, but only the degree of pain at twelve months.

## Conclusions

Our findings suggest that the preoperative factors of pain catastrophizing, symptomatic joints, pain, and radiographic osteoarthritis are correlated with pain one year after TKA. Pain are correlated with the six- and three- months pain outcomes, while mental health is correlated with pain at six months. However, our result highlights the need for further investigation on several factors that have been evaluated only once or in studies with small sample sizes. These factors should be considered when developing predictive models to identify patients most likely to experience chronic post-surgical pain. Accurately identifying factors associated with the pain outcome will be crucial for the development of effective predictive models.

## Supporting information

S1 Checklist PRISMA 2020 checklist. (PDF)

S1 Appendix Methods and results multivariate meta-analyses. (PDF)

S2 Appendix Exploring potential inconsistency. (PDF)

S3 Appendix Univariate meta-analysis. (PDF)

S4 Appendix Sensitivity analysis. (PDF)

S5 Appendix Definition and labels of the factors. (PDF)

S6 Appendix Risk of bias. (PDF)

S7 Appendix Grading of recommendation assessment, development and evaluation. (PDF)

S8 Appendix Search strategy. (DOCX)

S9 Appendix Reason for exclusion of individual studies. (DOCX)

## References

[1] Price AJ, Alvand A, Troelsen A, Katz JN, Hooper G, Gray A, et al. Knee replacement. Lancet. 2018;392(10158):1672–82. doi: 10.1016/S0140-6736(18)32344-4 30496082

[2] Healthcare Costs and Utilization Project (HCUP). National Inpatient Sample (NIS), 2019 [Internet]. Available from: https://datatools.ahrq.gov/hcup-fast-stats?type=subtab&tab=hcupfsis&count=4.

[3] Beswick AD, Wylde V, Gooberman-Hill R, Blom A, Dieppe P. What proportion of patients report long-term pain after total hip or knee replacement for osteoarthritis? A systematic review of prospective studies in unselected patients. BMJ Open. 2012;2(1):e000435. doi: 10.1136/bmjopen-2011-000435 22357571 PMC3289991

[4] Petersen KK, Simonsen O, Laursen MB, Nielsen TA, Rasmussen S, Arendt-Nielsen L. Chronic postoperative pain after primary and revision total knee arthroplasty. Clin J Pain. 2015;31(1):1–6. doi: 10.1097/AJP.0000000000000146 25485953

[5] Schug SA, Lavand’homme P, Barke A, Korwisi B, Rief W, Treede RD, et al. The IASP classification of chronic pain for ICD-11: chronic postsurgical or posttraumatic pain. Pain. 2019;160(1):45–52. doi: 10.1097/j.pain.0000000000001413 30586070

[6] Treede RD, Rief W, Barke A, Aziz Q, Bennett MI, Benoliel R, et al. Chronic pain as a symptom or a disease: the IASP Classification of Chronic Pain for the International Classification of Diseases (ICD-11). Pain. 2019;160(1):19–27. doi: 10.1097/j.pain.0000000000001384 30586067

[7] Kahlenberg CA, Nwachukwu BU, McLawhorn AS, Cross MB, Cornell CN, Padgett DE. Patient Satisfaction After Total Knee Replacement: A Systematic Review. HSS J. 2018;14(2):192–201. doi: 10.1007/s11420-018-9614-8 29983663 PMC6031540

[8] Pinedo-Villanueva R, Kolovos S, Maronga C, Delmestri A, Howells N, Judge A, et al. Primary care consultations and pain medicine prescriptions: a comparison between patients with and without chronic pain after total knee replacement. BMC Musculoskelet Disord. 2022;23(1):548. doi: 10.1186/s12891-022-05492-6 35672693 PMC9172077

[9] Nakano N, Shoman H, Olavarria F, Matsumoto T, Kuroda R, Khanduja V. Why are patients dissatisfied following a total knee replacement? A systematic review. Int Orthop. 2020;44(10):1971. doi: 10.1007/s00264-020-04607-9 32642827 PMC7584563

[10] Zaffagnini S, Di Paolo S, Meena A, Alesi D, Zinno R, Barone G, et al. Causes of stiffness after total knee arthroplasty: a systematic review. Int Orthop. 2021;45(8):1983–99. doi: 10.1007/s00264-021-05023-3 33821306

[11] Kent P, Cancelliere C, Boyle E, Cassidy JD, Kongsted A. A conceptual framework for prognostic research. BMC Med Res Methodol. 2020;20(1):172. doi: 10.1186/s12874-020-01050-7 32600262 PMC7325141

[12] Riley RD, Moons KGM, Snell KIE, Ensor J, Hooft L, Altman DG, et al. A guide to systematic review and meta-analysis of prognostic factor studies. BMJ. 2019;364:k4597. doi: 10.1136/bmj.k4597 30700442

[13] Harmelink KEM, Zeegers A, Hullegie W, Hoogeboom TJ, Nijhuis-van der Sanden MWG, Staal JB. Are There Prognostic Factors for One-Year Outcome After Total Knee Arthroplasty? A Systematic Review. J Arthroplasty. 2017;32(12):3840–53 e1. doi: 10.1016/j.arth.2017.07.011 28927646

[14] Vissers MM, Bussmann JB, Verhaar JA, Busschbach JJ, Bierma-Zeinstra SM, Reijman M. Psychological factors affecting the outcome of total hip and knee arthroplasty: a systematic review. Semin Arthritis Rheum. 2012;41(4):576–88. doi: 10.1016/j.semarthrit.2011.07.003 22035624

[15] Santaguida PL, Hawker GA, Hudak PL, Glazier R, Mahomed NN, Kreder HJ, et al. Patient characteristics affecting the prognosis of total hip and knee joint arthroplasty: a systematic review. Can J Surg. 2008;51(6):428–36. 19057730 PMC2592576

[16] Shohat N, Heller S, Sudya D, Small I, Khawalde K, Khatib M, et al. Mild radiographic osteoarthritis is associated with increased pain and dissatisfaction following total knee arthroplasty when compared with severe osteoarthritis: a systematic review and meta-analysis. Knee Surg Sports Traumatol Arthrosc. 2022;30(3):965–81. doi: 10.1007/s00167-021-06487-x 33604736

[17] Pozzobon D, Ferreira PH, Blyth FM, Machado GC, Ferreira ML. Can obesity and physical activity predict outcomes of elective knee or hip surgery due to osteoarthritis? A meta-analysis of cohort studies. BMJ Open. 2018;8(2):e017689. doi: 10.1136/bmjopen-2017-017689 29487072 PMC5855486

[18] Lewis GN, Rice DA, McNair PJ, Kluger M. Predictors of persistent pain after total knee arthroplasty: a systematic review and meta-analysis. Br J Anaesth. 2015;114(4):551–61. doi: 10.1093/bja/aeu441 25542191

[19] Khatib Y, Madan A, Naylor JM, Harris IA. Do Psychological Factors Predict Poor Outcome in Patients Undergoing TKA? A Systematic Review. Clin Orthop Relat Res. 2015;473(8):2630. doi: 10.1007/s11999-015-4234-9 25791440 PMC4488213

[20] Duan G, Liu C, Lin W, Shao J, Fu K, Niu Y, et al. Different Factors Conduct Anterior Knee Pain Following Primary Total Knee Arthroplasty: A Systematic Review and Meta-Analysis. J Arthroplasty. 2018;33(6):1962–71 e3. doi: 10.1016/j.arth.2017.12.024 29398258

[21] Youlden DJ, Dannaway J, Enke O. Radiographic severity of knee osteoarthritis and its relationship to outcome post total knee arthroplasty: a systematic review. ANZ J Surg. 2020;90(3):237–42. doi: 10.1111/ans.15343 31338950

[22] Burns LC, Ritvo SE, Ferguson MK, Clarke H, Seltzer Z, Katz J. Pain catastrophizing as a risk factor for chronic pain after total knee arthroplasty: a systematic review. J Pain Res. 2015;8:21–32. doi: 10.2147/JPR.S64730 25609995 PMC4294690

[23] Lungu E, Vendittoli PA, Desmeules F. Preoperative Determinants of Patient-reported Pain and Physical Function Levels Following Total Knee Arthroplasty: A Systematic Review. Open Orthop J. 2016;10:213–31. doi: 10.2174/1874325001610010213 27398109 PMC4920971

[24] van Jonbergen HP, Reuver JM, Mutsaerts EL, Poolman RW. Determinants of anterior knee pain following total knee replacement: a systematic review. Knee Surg Sports Traumatol Arthrosc. 2014;22(3):478–99. doi: 10.1007/s00167-012-2294-x 23160846

[25] Olsen U, Lindberg MF, Denison EM, Rose CJ, Gay CL, Aamodt A, et al. Predictors of chronic pain and level of physical function in total knee arthroplasty: a protocol for a systematic review and meta-analysis. BMJ Open. 2020;10(9):e037674. doi: 10.1136/bmjopen-2020-037674 32912987 PMC7485240

[26] Rose CJ, Olsen U, Lindberg M, Denison E-L, Aamodt A, Lerdal A. A new multivariate meta-analysis model for many variates and few studies. arXiv. GIT revision: 72ca65b. arXiv. Published online September 24, 2020. [updated February 12, 2021. doi: 10.48550/arXiv.2009.11808].

[27] Higgins JPT, Thomas J, Chandler J, Cumpston M, Li T, Page M, et al. Cochrane Handbook for Systematic Reviews of Interventions version 6.2 2020. Available from: https://www.training.cochrane.org/handbook.

[28] Hayden JA, van der Windt DA, Cartwright JL, Cote P, Bombardier C. Assessing bias in studies of prognostic factors. Ann Intern Med. 2013;158(4):280–6. doi: 10.7326/0003-4819-158-4-201302190-00009 23420236

[29] Iorio A, Spencer FA, Falavigna M, Alba C, Lang E, Burnand B, et al. Use of GRADE for assessment of evidence about prognosis: rating confidence in estimates of event rates in broad categories of patients. BMJ. 2015;350:h870. doi: 10.1136/bmj.h870 25775931

[30] Olsen U, Lindberg MF, Rose C, Denison E, Gay C, Aamodt A, et al. Factors Correlated With Physical Function 1 Year After Total Knee Arthroplasty in Patients With Knee Osteoarthritis: A Systematic Review and Meta-analysis. JAMA Netw Open. 2022;5(7):e2219636. doi: 10.1001/jamanetworkopen.2022.19636 35816307 PMC9274324

[31] Borenstein M, Hedges LV, Higgins JP, Rothstein HR. Introduction to meta-analysis. Cornwall, United Kingdom: John Wiley & Sons; 2009.

[32] Rucker G, Schwarzer G. Ranking treatments in frequentist network meta-analysis works without resampling methods. BMC Med Res Methodol. 2015;15:58. doi: 10.1186/s12874-015-0060-8 26227148 PMC4521472

[33] Attal N, Masselin-Dubois A, Martinez V, Jayr C, Albi A, Fermanian J, et al. Does cognitive functioning predict chronic pain? Results from a prospective surgical cohort. Brain. 2014;137(Pt 3):904–17. doi: 10.1093/brain/awt354 24441173

[34] Dave AJ, Selzer F, Losina E, Usiskin I, Collins JE, Lee YC, et al. The association of pre-operative body pain diagram scores with pain outcomes following total knee arthroplasty. Osteoarthritis Cartilage. 2017;25(5):667–75. doi: 10.1016/j.joca.2016.12.013 27986621 PMC5403582

[35] Lingard EA, Riddle DL. Impact of psychological distress on pain and function following knee arthroplasty. J Bone Joint Surg Am. 2007;89(6):1161–9. doi: 10.2106/JBJS.F.00914 17545417

[36] Dowsey MM, Nikpour M, Dieppe P, Choong PF. Associations between pre-operative radiographic changes and outcomes after total knee joint replacement for osteoarthritis. Osteoarthritis Cartilage. 2012;20(10):1095–102. doi: 10.1016/j.joca.2012.05.015 22800770

[37] Wylde V, Dixon S, Blom AW. The role of preoperative self-efficacy in predicting outcome after total knee replacement. Musculoskeletal Care. 2012;10(2):110–8. doi: 10.1002/msc.1008 22368121

[38] Sullivan M, Tanzer M, Reardon G, Amirault D, Dunbar M, Stanish W. The role of presurgical expectancies in predicting pain and function one year following total knee arthroplasty. Pain. 2011;152(10):2287–93. doi: 10.1016/j.pain.2011.06.014 21764515

[39] Getachew M, Lerdal A, Smastuen MC, Gay CL, Aamodt A, Tesfaye M, et al. High levels of preoperative pain and fatigue are red flags for moderate-severe pain 12 months after total knee arthroplasty-A longitudinal cohort study. Musculoskeletal Care. 2021;19(2):186–92. doi: 10.1002/msc.1522 33085181 PMC8247059

[40] Kornilov N, Lindberg MF, Gay C, Saraev A, Kuliaba T, Rosseland LA, et al. Higher physical activity and lower pain levels before surgery predict non-improvement of knee pain 1 year after TKA. Knee Surg Sports Traumatol Arthrosc. 2018;26(6):1698–708. doi: 10.1007/s00167-017-4713-5 28916991

[41] Giordano R, Petersen KK, Andersen HH, Lichota J, Valeriani M, Simonsen O, et al. Preoperative serum circulating microRNAs as potential biomarkers for chronic postoperative pain after total knee replacement. Mol Pain. 2020;16:1744806920962925. doi: 10.1177/1744806920962925 33021154 PMC7543153

[42] Petersen KK, Arendt-Nielsen L, Simonsen O, Wilder-Smith O, Laursen MB. Presurgical assessment of temporal summation of pain predicts the development of chronic postoperative pain 12 months after total knee replacement. Pain. 2015;156(1):55–61. doi: 10.1016/j.pain.0000000000000022 25599301

[43] Petersen KK, Arendt-Nielsen L, Vela J, Skou ST, Eld M, Al-Mashkur NM, et al. Less Severe Preoperative Synovitis is Associated With Higher Self-reported Pain Intensity 12 Months After Total Knee Arthroplasty-An Exploratory Prospective Observational Study. Clin J Pain. 2020;36(1):34–40. doi: 10.1097/AJP.0000000000000768 31794440

[44] Petersen KK, Simonsen O, Laursen MB, Arendt-Nielsen L. The Role of Preoperative Radiological Severity, Sensory Testing, and Temporal Summation on Chronic Postoperative Pain following Total Knee Arthroplasty. Clin J Pain. 2017;34(3):193–7. 10.1097/AJP.0000000000000528 28654559

[45] Tilbury C, Haanstra TM, Verdegaal SHM, Nelissen R, de Vet HCW, Vliet Vlieland TPM, et al. Patients’ pre-operative general and specific outcome expectations predict postoperative pain and function after total knee and total hip arthroplasties. Scand J Pain. 2018;18(3):457–66. doi: 10.1515/sjpain-2018-0022 29794270

[46] van de Water R, Leichtenberg C, Nelissen R, Kroon H, Kaptijn H, Onstenk R, et al. Preoperative Radiographic Osteoarthritis Severity Modifies the Effect of Preoperative Pain on Pain/Function After Total Knee Arthroplasty: Results at 1 and 2 Years Postoperatively. J Bone Joint Surgery Am. 2019;101(10):879. 10.2106/JBJS.18.00642 31094979

[47] Escobar A, Quintana JM, Bilbao A, Azkarate J, Guenaga JI, Arenaza JC, et al. Effect of patient characteristics on reported outcomes after total knee replacement. Rheumatology (Oxford). 2007;46(1):112–9. doi: 10.1093/rheumatology/kel184 16735451

[48] Hardy A, Sandiford MH, Menigaux C, Bauer T, Klouche S, Hardy P. Pain catastrophizing and pre-operative psychological state are predictive of chronic pain after joint arthroplasty of the hip, knee or shoulder: results of a prospective, comparative study at one year follow-up. Int Orthop. 2022;46(11):2461–9. doi: 10.1007/s00264-022-05542-7 35999466 PMC9556350

[49] Cremeans-Smith JK, Greene K, Delahanty DL. Physiological Indices of Stress Prior to and Following Total Knee Arthroplasty Predict the Occurrence of Severe Post-Operative Pain. Pain Med. 2016;17(5):970–9. doi: 10.1093/pm/pnv043 26814277

[50] Pua YH, Poon CL, Seah FJ, Thumboo J, Clark RA, Tan MH, et al. Predicting individual knee range of motion, knee pain, and walking limitation outcomes following total knee arthroplasty. Acta Orthop. 2019;90(2):179–86. doi: 10.1080/17453674.2018.1560647 30973090 PMC6461070

[51] Yang HY, Losina E, Lange JK, Katz JN, Collins JE. Longitudinal Trajectories of Pain and Function Improvement Following Total Knee Replacement. ACR Open Rheumatol. 2019;1(5):308–17. doi: 10.1002/acr2.1041 31777807 PMC6858006

[52] Bossmann T, Brauner T, Wearing S, Horstmann T. Predictors of chronic pain following total knee replacement in females and males: an exploratory study. Pain Manag. 2017;7(5):391–403. doi: 10.2217/pmt-2017-0023 28936909

[53] Fitzsimmons M, Carr E, Woodhouse L, Bostick GP. Development and Persistence of Suspected Neuropathic Pain After Total Knee Arthroplasty in Individuals With Osteoarthritis. PM R. 2018;10(9):903–9. doi: 10.1016/j.pmrj.2018.01.010 29452296

[54] Bruehl S, Milne G, Schildcrout J, Shi Y, Anderson S, Shinar A, et al. Perioperative oxidative stress predicts subsequent pain-related outcomes in the 6 months after total knee arthroplasty. Pain. 2023;164(1):111–8. doi: 10.1097/j.pain.0000000000002670 35507374 PMC9633585

[55] Chen F, Gao W, Hu J, Yang X, Chai X, Wang D. Preoperative angiotensin II type 2 receptor is a predictor for developing chronic post-surgical pain after total knee arthroplasty surgery. Life Sci. 2021;278:119654. doi: 10.1016/j.lfs.2021.119654 34043993

[56] Edwards RR, Campbell C, Schreiber KL, Meints S, Lazaridou A, Martel MO, et al. Multimodal prediction of pain and functional outcomes 6 months following total knee replacement: a prospective cohort study. BMC Musculoskelet Disord. 2022;23(1):302. doi: 10.1186/s12891-022-05239-3 35351066 PMC8966339

[57] Bugada D, Allegri M, Gemma M, Ambrosoli AL, Gazzerro G, Chiumiento F, et al. Effects of anaesthesia and analgesia on long-term outcome after total knee replacement: A prospective, observational, multicentre study. Eur J Anaesthesiol. 2017;34(10):665–72. doi: 10.1097/EJA.0000000000000656 28767456 PMC5588609

[58] Engel C, Hamilton NA, Potter PT, Zautra AJ. Impact of two types of expectancy on recovery from total knee replacement surgery (TKR) in adults with osteoarthritis. Behav Med. 2004;30(3):113–23. doi: 10.3200/BMED.30.3.113-123 15816314

[59] Luo ZY, Li LL, Wang D, Wang HY, Pei FX, Zhou ZK. Preoperative sleep quality affects postoperative pain and function after total joint arthroplasty: a prospective cohort study. J Orthop Surg Res. 2019;14(1):378. doi: 10.1186/s13018-019-1446-9 31752947 PMC6868862

[60] Perruccio A, Fitzpatrick J, Power J, Gandhi R, Rampersaud Y, Mahomed N, et al. The effects of depression, low back pain and comorbidities on pain after total knee arthroplasty for osteoarthritis are modified by sex. Arthritis Care Res (Hoboken). 2019. 10.1002/acr.24002 31199582

[61] Lindner M, Nosseir O, Keller-Pliessnig A, Teigelack P, Teufel M, Tagay S. Psychosocial predictors for outcome after total joint arthroplasty: a prospective comparison of hip and knee arthroplasty. BMC Musculoskelet Disord. 2018;19(1):159. doi: 10.1186/s12891-018-2058-y 29788969 PMC5964720

[62] Amusat N, Beaupre L, Jhangri GS, Pohar SL, Simpson S, Warren S, et al. Diabetes that impacts on routine activities predicts slower recovery after total knee arthroplasty: an observational study. J Physiother. 2014;60(4):217–23. doi: 10.1016/j.jphys.2014.09.006 25443651

[63] Lingard EA, Katz JN, Wright EA, Sledge CB, Kinemax Outcomes G. Predicting the outcome of total knee arthroplasty. J Bone Joint Surg Am. 2004;86(10):2179–86. doi: 10.2106/00004623-200410000-00008 15466726

[64] Papakostidou I, Dailiana ZH, Papapolychroniou T, Liaropoulos L, Zintzaras E, Karachalios TS, et al. Factors affecting the quality of life after total knee arthroplasties: a prospective study. BMC Musculoskelet Disord. 2012;13:116. doi: 10.1186/1471-2474-13-116 22748117 PMC3476961

[65] Belford K, Gallagher N, Dempster M, Wolfenden M, Hill J, Blaney J, et al. Psychosocial predictors of outcomes up to one year following total knee arthroplasty. Knee. 2020;27(3):1028–34. doi: 10.1016/j.knee.2020.03.006 32299757

[66] Sharma S, Kumar V, Sood M, Malhotra R. Effect of Preoperative Modifiable Psychological and Behavioural Factors on Early Outcome Following Total Knee Arthroplasty in an Indian Population. Indian J Orthop. 2021;55(4):939–47. doi: 10.1007/s43465-020-00325-x 34194651 PMC8192610

[67] Barroso J, Wakaizumi K, Reckziegel D, Pinto-Ramos J, Schnitzer T, Galhardo V, et al. Prognostics for pain in osteoarthritis: Do clinical measures predict pain after total joint replacement? PLoS One. 2020;15(1):e0222370. doi: 10.1371/journal.pone.0222370 31914126 PMC6948829

[68] Carriere JS, Martel MO, Loggia ML, Campbell CM, Smith MT, Haythornthwaite JA, et al. The Influence of Expectancies on Pain and Function Over Time After Total Knee Arthroplasty. Pain Med. 2022;23(10):1767–76. doi: 10.1093/pm/pnac067 35482515 PMC9527599

[69] Chodor P, Kruczynski J. Preoperative Risk Factors of Persistent Pain following Total Knee Arthroplasty. BioMed Res Int. 2022;2022:4958089. doi: 10.1155/2022/4958089 36567908 PMC9780009

[70] Sorel JC, Veltman ES, Honig A, Poolman RW. The influence of preoperative psychological distress on pain and function after total knee arthroplasty: a systematic review and meta-analysis. Bone Joint J. 2019;101-B(1):7–14. doi: 10.1302/0301-620X.101B1.BJJ-2018-0672.R1 30601044

[71] Birch S, Stilling M, Mechlenburg I, Hansen TB. No effect of cognitive behavioral patient education for patients with pain catastrophizing before total knee arthroplasty: a randomized controlled trial. Acta Orthop. 2020;91(1):98–103. doi: 10.1080/17453674.2019.1694312 31762342 PMC7006640

[72] Bay S, Kuster L, McLean N, Byrnes M, Kuster MS. A systematic review of psychological interventions in total hip and knee arthroplasty. BMC Musculoskelet Disord. 2018;19(1):201. doi: 10.1186/s12891-018-2121-8 30037341 PMC6055334

[73] Dowsey MM, Smith AJ, Choong PFM. Latent Class Growth Analysis predicts long term pain and function trajectories in total knee arthroplasty: a study of 689 patients. Osteoarthritis Cartilage. 2015;23(12):2141–9. doi: 10.1016/j.joca.2015.07.005 26187575

[74] Lindberg MF, Rustoen T, Miaskowski C, Rosseland LA, Lerdal A. The relationship between pain with walking and self-rated health 12 months following total knee arthroplasty: a longitudinal study. BMC Musculoskelet Disord. 2017;18(1):75. doi: 10.1186/s12891-017-1430-7 28183297 PMC5301389

[75] Sayah SM, Karunaratne S, Beckenkamp PR, Horsley M, Hancock MJ, Hunter DJ, et al. Clinical Course of Pain and Function Following Total Knee Arthroplasty: A Systematic Review and Meta-Regression. J Arthroplasty. 2021;36(12):3993–4002 e37. doi: 10.1016/j.arth.2021.06.019 34275710

[76] Bannuru RR, Osani MC, Vaysbrot EE, Arden NK, Bennell K, Bierma-Zeinstra SMA, et al. OARSI guidelines for the non-surgical management of knee, hip, and polyarticular osteoarthritis. Osteoarthritis Cartilage. 2019;27(11):1578–89. doi: 10.1016/j.joca.2019.06.011 31278997

