# Supplementary material for: Factors correlated with pain after total knee arthroplasty: A systematic review and meta-analysis
Source: PLoS One. 2023 Mar 24;18(3):e0283446. doi: 10.1371/journal.pone.0283446 (PMC10038299; doi:10.1371/journal.pone.0283446)
Supplement: S1 Appendix — (PDF) [file pone.0283446.s002.pdf]

S1 Appendix Methods and results multivariate-meta-analyses

Predictors of chronic pain in total knee arthroplasty: meta-analyses

Chris Rose, Norwegian Institute of Public Health (22 Feb 2023)

Generated using git revision: d58fd76

Introduction

This document presents the meta-analyses of predictors of chronic pain after total knee arthroplasty in detail.

Methods

Except where noted, we performed statistical analyses according to the method prespecified in our protocol (Olsen 2020).

We imputed correlation coefficients from estimates of association expressed as odds ratios, risk ratios, and linear model coefficients (including differences) as described in our protocol's supplementary materials. Where it was necessary to impute odds ratios from risk ratios prior to imputing correlation, we assumed a prespecified baseline probability of postsurgical pain of 20%. We defined canonical directions for all outcomes and predictors and inverted reported directions of association as appropriate to ensure consistent directions of association in meta-analysis.

If studies did not report confidence intervals or sampling variances, we imputed them as appropriate (Higgins 2019). If a study did not report exact statements of uncertainty but provided statements about "statistical significance", we used a conservative approach in which we imputed "worst case" standard errors. For example, we imputed $P \leq 0.01$ to mean $P=0.01$ and "not statistically significant" to mean $P=0.99$. We performed all meta-analyses on the scale of Fisher's z (hyperbolic arctangent, not Z-score; Borenstein 2009). We used the inverse transform (hyperbolic tangent) to report meta-analytical estimates as correlation coefficients.

We anticipated that predictors may be correlated and that there may be important differences in the methods used to quantify associations. We therefore planned to perform multivariate meta-analysis for each outcome using a random-effects model. We had planned to use the multivariate extension to Riley's (2008) bivariate model, implemented in the MVMETA add-on command for Stata by White (2009, 2011), which assumes a common within-study correlation matrix. Unfortunately, the data were too sparse for this model to be fitted without substantially limiting the number of predictors included in the analysis. Even then, the model appears to dramatically underestimate standard error for pain 12 months post-surgery. We therefore developed a multivariate model based on that of Lin and Chu (2018). We approximate common within-study correlations and between-study heterogeneity using a low-dimensional variance-covariance matrix (Rose 2020). We had planned to identify predictors likely to be most strongly associated with postoperative pain by estimating the probability of superiority of each predictor using the pbest option of MVMETA. Because that model could not be used, we assessed superiority using P-

scores (cf. p-values; Rücker and Schwarzer 2015), in which larger magnitudes were defined to be superior to those with smaller magnitudes. Unlike the probabilities we had planned to estimate, P-scores are not as heavily influenced by imprecisely estimated predictors with small point estimates whose confidence intervals extend far beyond those of more precisely estimated predictors with larger point estimates. This is particularly important for multivariate meta-analysis of correlations, in which the superiority of a predictor is a function of the magnitude of its coefficient rather than its magnitude and direction, as is the case in multiple treatment comparison via network meta-analysis. P-scores are therefore likely to better identify good predictors. Multivariate estimates of correlation are presented as forest plots, which also show I^2 statistics (the percentage of heterogeneity attributable to between-study differences rather than sampling error) and the numbers of studies that provided usable estimates for each predictor. We also performed exploratory univariate meta-analyses for each predictor and outcome, but which do not account for correlation. We compared estimates from the three approaches to identify possible inconsistency. We report 95% confidence intervals throughout. Statistical analyses were performed using Stata 16 (StataCorp LLC, College Station, Texas, USA).

We had planned to investigate non-reporting bias and small study effects for predictors supported by at least 10 results. However, none of the predictors met this criterion. Similarly, we had planned to perform subgroup analyses with respect to study design, type of outcome measurement, and intervention if at least five studies could be included in each subgroup. However, this criterion was not satisfied, and no subgroup analyses were performed.

We performed a sensitivity analysis for the primary outcome (pain 12 months post-surgery). For each of the six QUIPS risk of bias domains, we excluded studies judged to be at high risk of bias, re-ran the multivariate meta-analysis, and compared the estimated correlations with those obtained when all studies are included. We had planned to do a leave-one-study-out sensitivity analysis to explore the influence of each study on the meta-analysis results. Unfortunately, this was not feasible. However, the effect of particular studies can be inferred by inspecting the univariate meta-analyses.

Results

The results of the multivariate meta-analyses are presented in the following subsections.

S1 Fig. Postsurgical Pain (3 months) — Multivariate meta-analysis

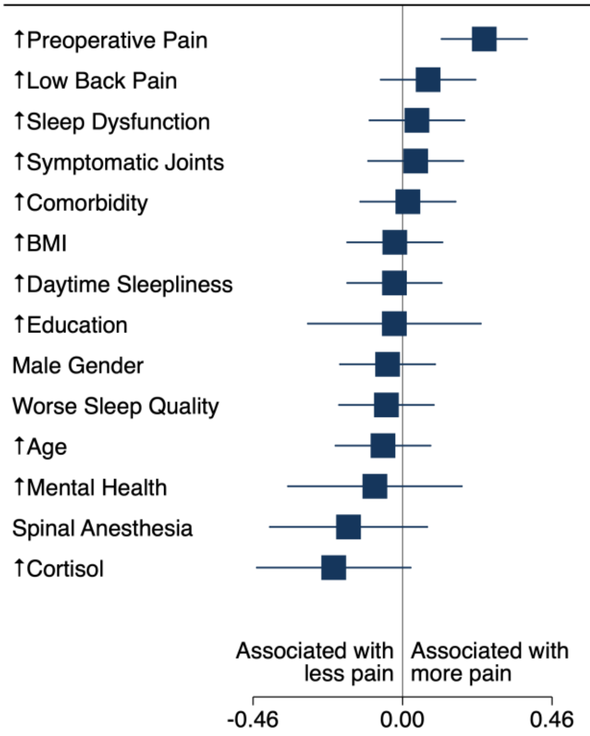

	Correlation	95% CI			*p*-value	Studies	*P*-score	I^2
↑Preoperative Pain	0.27	0.13	to	0.39	0.00	3	81.0%	0.0%
↑Low Back Pain	0.09	-0.07	to	0.24	0.42	1	47.5%	.
↑Sleep Dysfunction	0.05	-0.11	to	0.20	0.76	1	42.1%	.
↑Symptomatic Joints	0.04	-0.11	to	0.20	0.80	1	41.8%	.
↑Comorbidity	0.02	-0.14	to	0.18	0.96	1	43.0%	.
↑BMI	-0.03	-0.18	to	0.13	0.92	1	41.8%	.
↑Daytime Sleepliness	-0.03	-0.18	to	0.13	0.92	1	41.6%	.
↑Education	-0.03	-0.31	to	0.26	0.97	1	46.9%	.
Male Gender	-0.05	-0.21	to	0.11	0.74	1	42.4%	.
Worse Sleep Quality	-0.05	-0.21	to	0.10	0.71	1	42.8%	.
↑Age	-0.07	-0.22	to	0.09	0.60	1	44.3%	.
↑Mental Health	-0.09	-0.37	to	0.20	0.74	2	49.8%	62.0%
Spinal Anesthesia	-0.18	-0.42	to	0.08	0.24	1	63.3%	.
↑Cortisol	-0.23	-0.45	to	0.03	0.09	1	71.6%	.

S2 Fig.. Postsurgical Pain (6 months) — Multivariate meta-analysis

	Correlation	95% CI			p-value	Studies	P-score	I^2
↑Oxidative Stress	0.33	0.12	to	0.52	0.00	1	84.5%	.
↑Trail Making Time	0.32	0.06	to	0.54	0.01	1	81.4%	.
↑Arthritis Helplessness	0.27	-0.02	to	0.52	0.08	1	71.8%	.
↑Walking Aid Use	0.25	-0.15	to	0.58	0.29	1	64.0%	.
↑AT2R Level	0.23	0.07	to	0.38	0.00	1	70.4%	.
↑Preoperative Pain	0.20	0.12	to	0.28	0.00	5	66.1%	37.6%
Other Chronic Pain	0.16	0.00	to	0.31	0.03	1	55.2%	.
Opioid Use	0.15	-0.01	to	0.30	0.05	1	52.6%	.
↑Low Back Pain	0.14	0.03	to	0.24	0.01	1	49.7%	.
↑Temporal Summation	0.13	-0.03	to	0.28	0.11	2	46.8%	0.0%
↑Catastrophizing	0.09	-0.03	to	0.21	0.16	4	36.5%	0.0%
↑Heart Rate Variability	0.08	-0.29	to	0.43	0.87	1	44.3%	.
↑Comorbidity	0.08	0.00	to	0.16	0.04	3	34.4%	25.0%
↑Symptomatic Joints	0.08	-0.04	to	0.20	0.22	2	34.4%	74.2%
↑Knee Flexion	0.08	0.01	to	0.15	0.03	1	33.3%	.
↑Education	0.07	-0.15	to	0.28	0.75	1	37.4%	.
↑Preoperative Function	0.06	-0.16	to	0.27	0.81	1	37.0%	.
↑Contralateral Knee Pain	0.04	-0.04	to	0.12	0.29	1	23.2%	.
↑Pain Expected	0.04	-0.14	to	0.21	0.86	1	32.9%	.
↑Knee Extension	0.00	-0.07	to	0.07	1.00	1	12.6%	.
↑BMI	-0.01	-0.08	to	0.06	0.92	2	19.7%	0.0%
Male Gender	-0.02	-0.09	to	0.06	0.81	3	19.3%	74.2%
↑Age	-0.03	-0.09	to	0.04	0.55	5	20.7%	52.3%
↑Social Support	-0.08	-0.15	to	-0.00	0.05	2	33.3%	0.0%
↑Disability	-0.10	-0.27	to	0.07	0.32	1	40.3%	.
↑Mental Health	-0.13	-0.24	to	-0.02	0.01	6	49.1%	29.1%
↑Sleep Effiency	-0.15	-0.30	to	0.01	0.05	1	52.3%	.
↑Ischemia Duration	-0.16	-0.40	to	0.09	0.29	1	52.4%	.
↑Agreeableness	-0.18	-0.34	to	-0.00	0.04	1	57.8%	.
Suspected Neuropathic Pain	-0.19	-0.44	to	0.09	0.24	1	57.1%	.
↑Sym/Parasym Activity	-0.28	-0.56	to	0.07	0.13	1	70.5%	.
Chinese Ethnicity	-0.35	-0.51	to	-0.16	0.00	1	86.9%	.
↑Pain Self-Efficacy	-0.35	-0.57	to	-0.08	0.01	1	84.8%	.
↑Pain Modulation	-0.38	-0.62	to	-0.08	0.01	1	86.9%	.

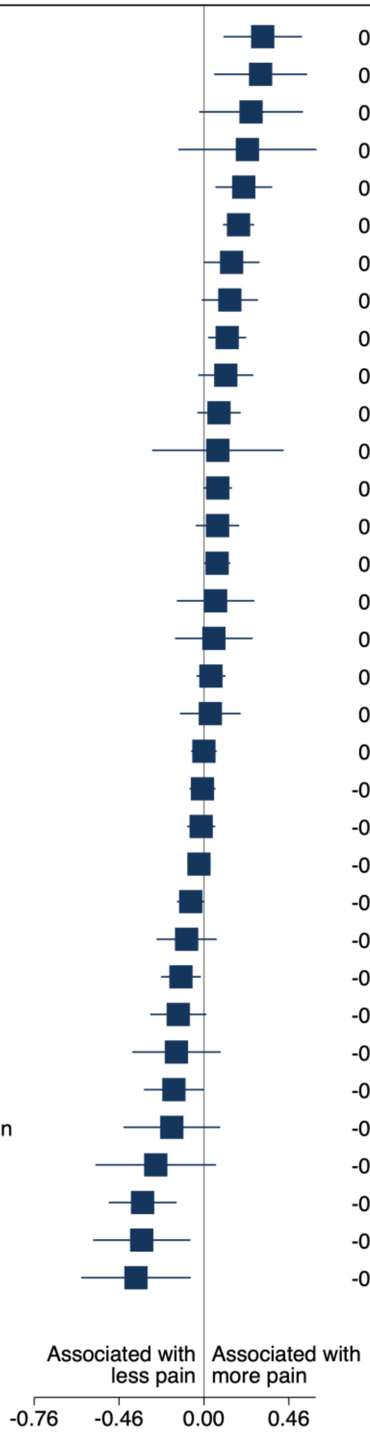

S3 Fig. Postsurgical Pain (12 months) — Multivariate meta-analysis

	Correlation	95% CI			*p*-value	Studies	*P*-score	I^2
↑Fatigue	0.40	0.06	to	0.66	0.02	1	80.4%	.
↑Catastrophizing	0.36	0.24	to	0.47	0.00	3	80.2%	72.4%
↑ROCF Recall	0.34	0.06	to	0.57	0.01	1	75.7%	.
↑Temporal Summation	0.21	0.05	to	0.36	0.00	2	61.1%	0.0%
↑miRNA-146a-5p	0.16	-0.04	to	0.35	0.15	1	49.8%	.
↑Heat Pain Threshold	0.16	-0.05	to	0.35	0.17	1	49.3%	.
↑Symptomatic Joints	0.15	0.08	to	0.23	0.00	2	51.3%	0.0%
Worse Sleep Quality	0.13	-0.32	to	0.54	0.78	1	46.2%	.
↑Preoperative Pain	0.13	0.06	to	0.19	0.00	9	44.6%	97.0%
↑BMI	0.09	-0.01	to	0.18	0.07	3	33.6%	72.9%
↑Warm Detection Threshold	0.08	-0.13	to	0.29	0.65	1	36.4%	.
↑Comorbidity	0.03	-0.06	to	0.12	0.67	3	23.8%	0.0%
↑Kinesophobia	0.00	-0.23	to	0.23	1.00	1	20.0%	.
↑Surgery Duration	-0.00	-0.23	to	0.23	1.00	1	20.0%	.
Male Gender	-0.01	-0.10	to	0.08	0.97	4	29.9%	0.0%
↑Preoperative Function	-0.02	-0.22	to	0.19	0.98	2	40.5%	90.1%
↑Pain Self-Efficacy	-0.03	-0.20	to	0.14	0.91	1	32.7%	.
↑Age	-0.03	-0.12	to	0.05	0.61	4	23.1%	0.0%
Patella Resurfaced	-0.05	-0.16	to	0.07	0.60	1	27.4%	.
↑Mental Health	-0.09	-0.17	to	0.00	0.07	6	33.4%	69.0%
Cruciate Retaining	-0.14	-0.25	to	-0.03	0.01	1	47.2%	.
↑Outcome Expected	-0.14	-0.36	to	0.09	0.30	1	46.0%	.
↑K-L Grade	-0.15	-0.23	to	-0.08	0.00	3	51.6%	0.0%
↑Synovitis Severity	-0.38	-0.67	to	-0.00	0.05	1	77.3%	.
↑Synovial Membrane Thickness	-0.46	-0.70	to	-0.11	0.01	1	85.5%	.
↑Synovial Perfusion (Volume)	-0.51	-0.72	to	-0.21	0.00	1	90.7%	.
↑Synovial Perfusion (Degree)	-0.53	-0.73	to	-0.24	0.00	1	92.0%	.

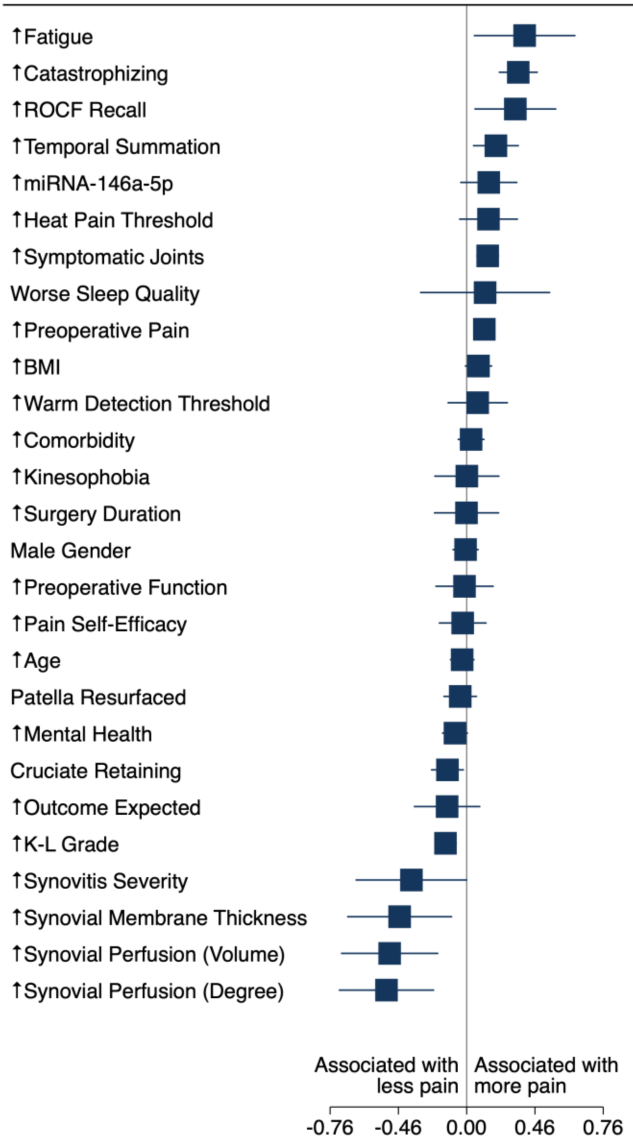

S4 Fig. Postsurgical pain at 3, 6, and 12 months

The following plot shows the multivariate meta-analytical estimates of correlation at each postoperative follow-up time. Estimates for predictors studied at only one postoperative time point are omitted.

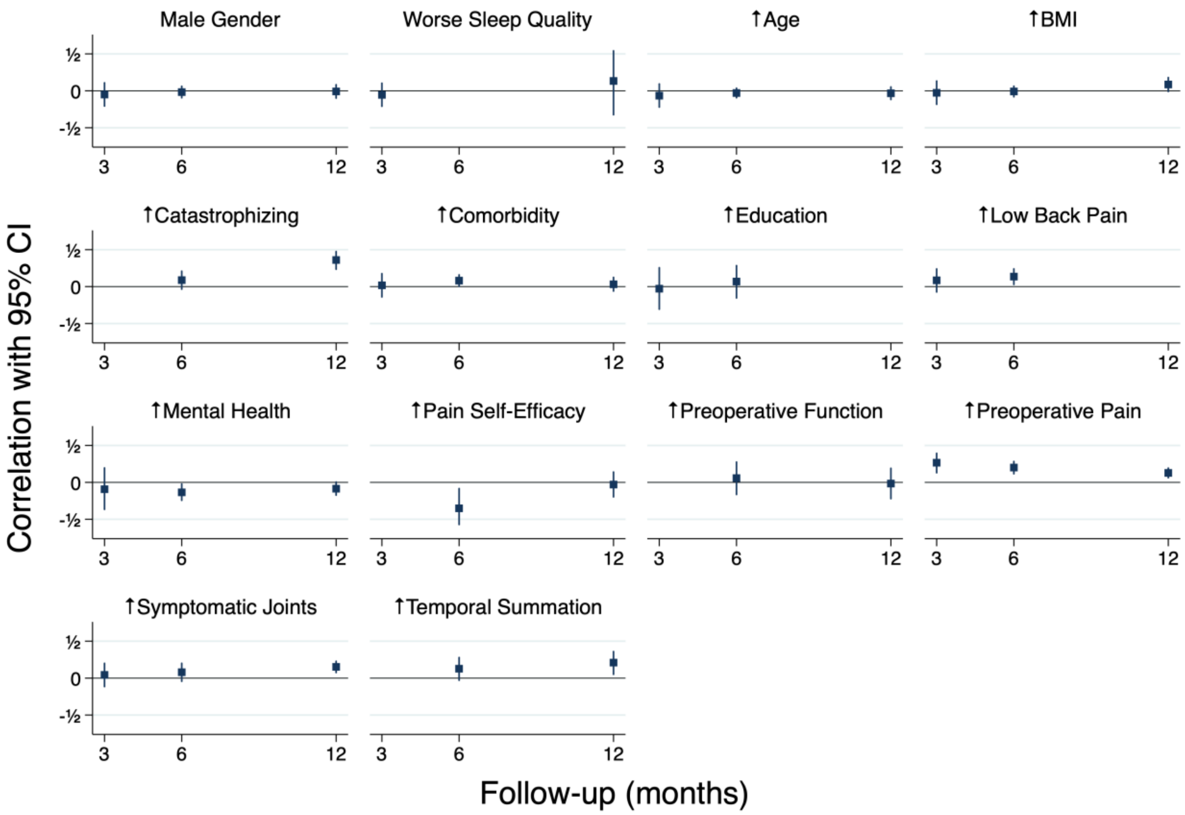

Predictors studied at only one postoperative time point are omitted

S5 Fig. Sensitivity analysis

The following plot shows the results of a sensitivity analysis in which the multivariate meta-analysis model was used to estimate correlations for each predictor, omitting all estimates from studies judged to be at high risk of bias for each of the six QUIPS domains. Estimates from the full meta-analysis are also included for comparison (shown as "All Studies").

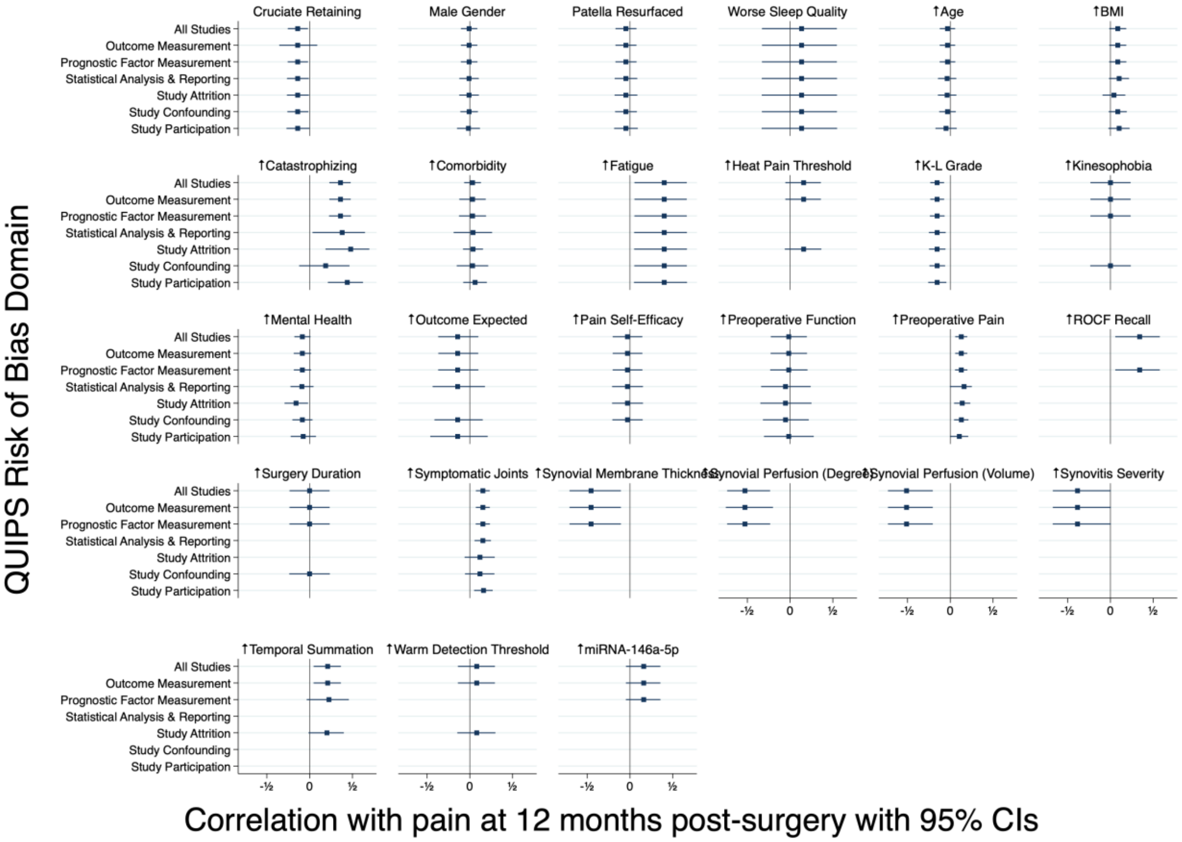

References

Borenstein M, Hedges LV, Higgins JP, et al. Introduction to meta-analysis: John Wiley & Sons; 2009.

Higgins JPT, Li T, Deeks JJ (editors). Chapter 6: Choosing effect measures and computing estimates of effect. In: Higgins JPT, Thomas J, Chandler J, Cumpston M, Li T, Page MJ, Welch VA (editors). Cochrane Handbook for Systematic Reviews of Interventions version 6.0 (updated July 2019). Cochrane, 2019.

Lin L and Chu H. Bayesian multivariate meta-analysis of multiple factors. Research Synthesis Methods 2018, 9(2): 261-272.

Olsen U, Lindberg MF, Denison EML, Rose CJ, Gay CL, Aamodt A, Brox IB, Skare Ø, Furnes O, Lee KA, Lerdal A. Predictors of chronic pain and level of physical function in total knee arthroplasty: a protocol for a systematic review and meta-analysis. BMJ Open 2020, 10(9): e037674.

Riley RD, Thompson JR, Abrams KR. An alternative model for bivariate random-effects meta-analysis when the within-study correlations are unknown. Biostatistics 2008, 9: 172-186

Rose CJ, Olsen U, Lindberg MF, Denison EL, Aamodt A, Lerdal A. A new multivariate meta-analysis model for many variates and few studies. arXiv preprint 2020, arXiv:2009.11808. Available at https://arxiv.org/abs/2009.11808

Rücker G, Schwarzer, G. Ranking treatments in frequentist network meta-analysis works without resampling methods. BMC Medical Research Methodology 2015, 15(58).

White IR. Multivariate random-effects meta-analysis. Stata Journal 2009, 9: 40-56.

White IR. Multivariate random-effects meta-regression: Updates to mvmeta. Stata Journal 2011, 11: 255-270.
