# Supplementary material for: Factors correlated with pain after total knee arthroplasty: A systematic review and meta-analysis
Source: PLoS One. 2023 Mar 24;18(3):e0283446. doi: 10.1371/journal.pone.0283446 (PMC10038299; doi:10.1371/journal.pone.0283446)

S2 Appendix — Exploring potential inconsistency

The following forest plots compare estimates between all models (where possible).S6 Fig. Postsurgical Pain (3 months) — Model comparison

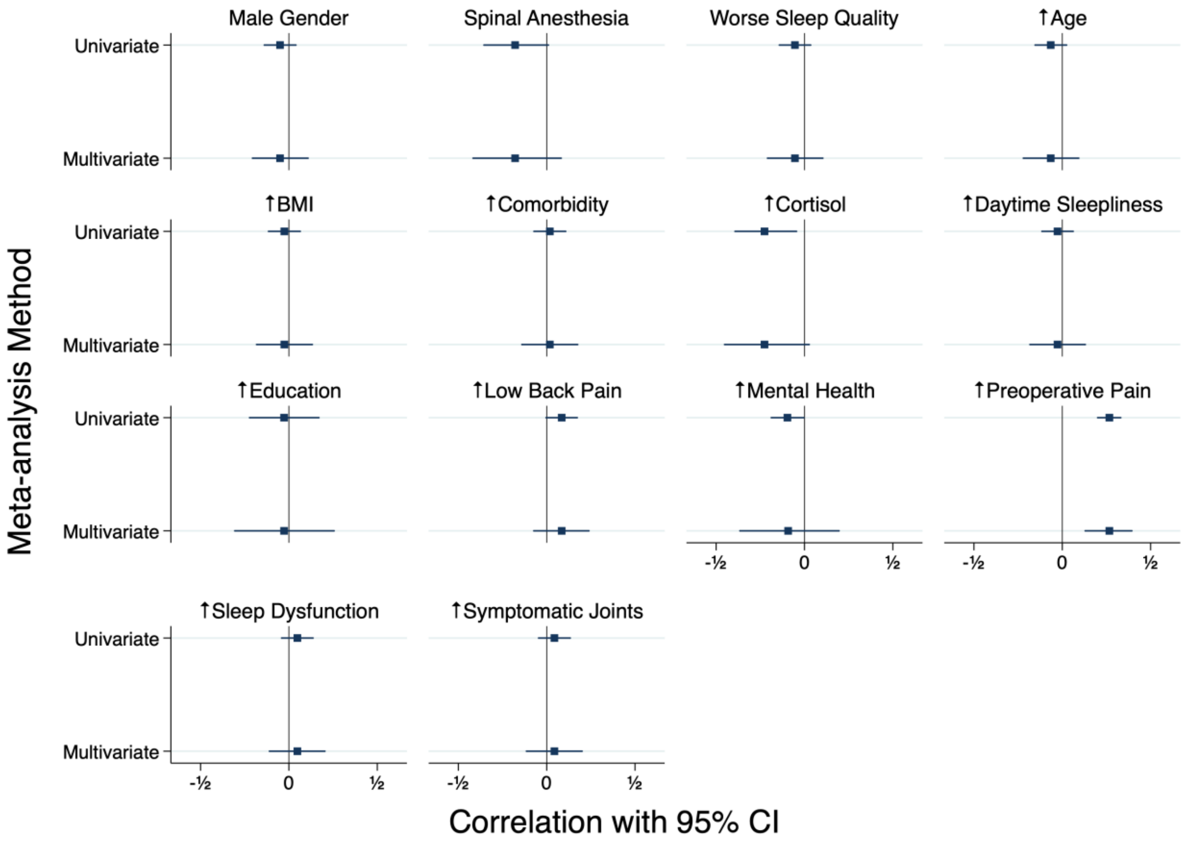

S7 Fig. Postsurgical Pain (6 months) — Model comparison

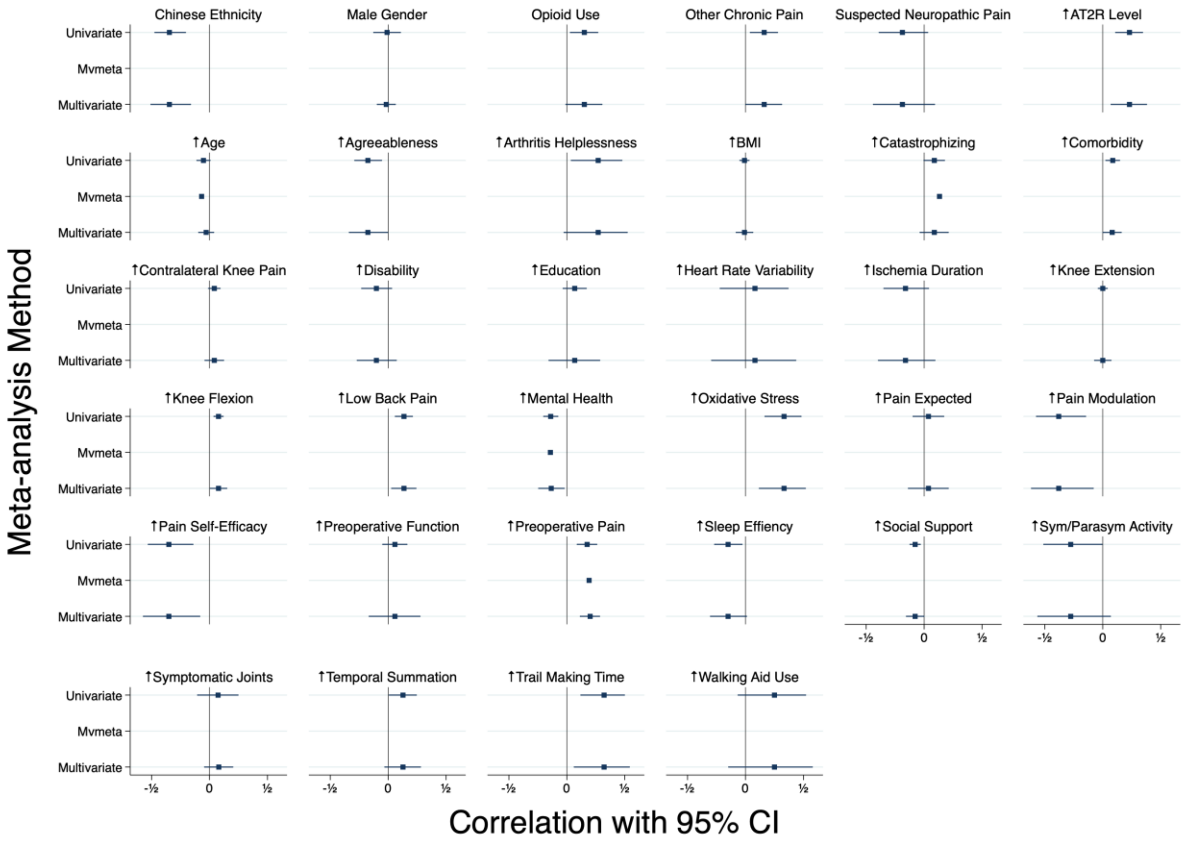

S8 Fig. Postsurgical Pain (12 months) — Model comparison

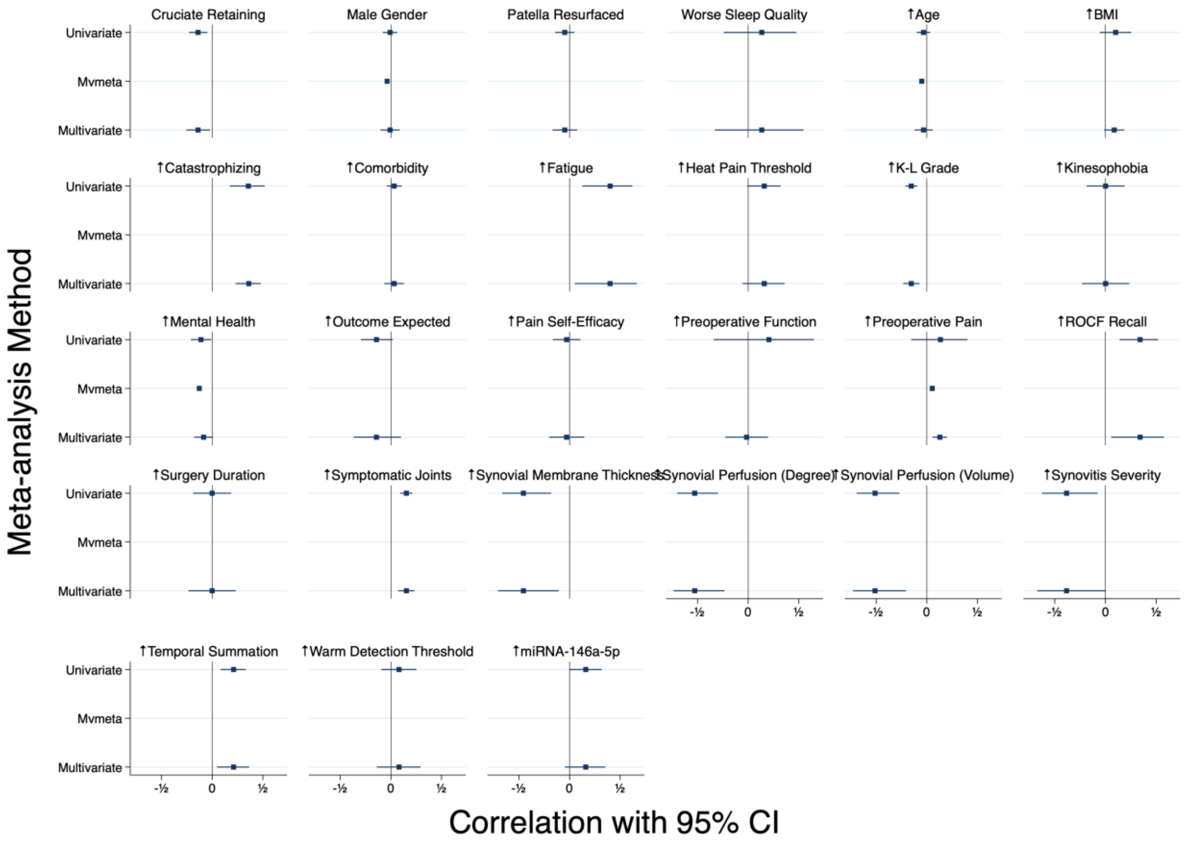

Supplement: S2 Appendix — (PDF) [file pone.0283446.s003.pdf]
