# Supplementary material for: Factors correlated with pain after total knee arthroplasty: A systematic review and meta-analysis
Source: PLoS One. 2023 Mar 24;18(3):e0283446. doi: 10.1371/journal.pone.0283446 (PMC10038299; doi:10.1371/journal.pone.0283446)

S3 Appendix — Univariate meta-analyses

The following forest plots show the results of exploratory univariate meta-analyses of the association between individual predictors and the outcomes. Note that these results do not account for any correlation between the predictors.

Pain (3 months)

S9 Fig. ↑Education

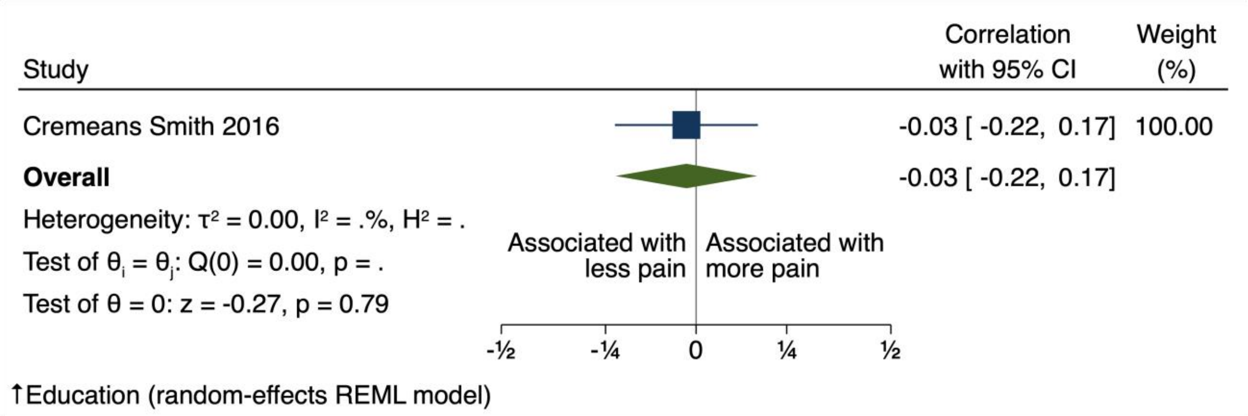

S10 Fig. ↑Mental Health

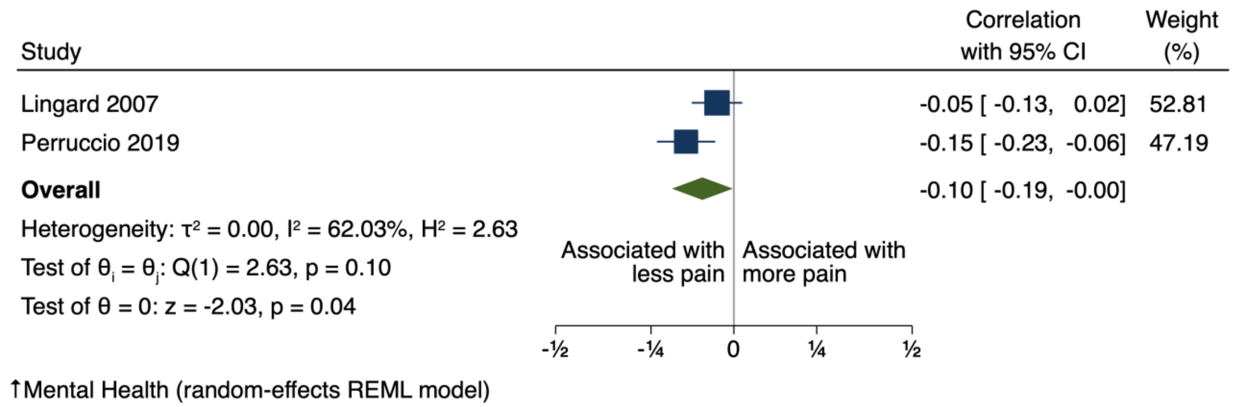

S11 Fig. ↑Comorbidity

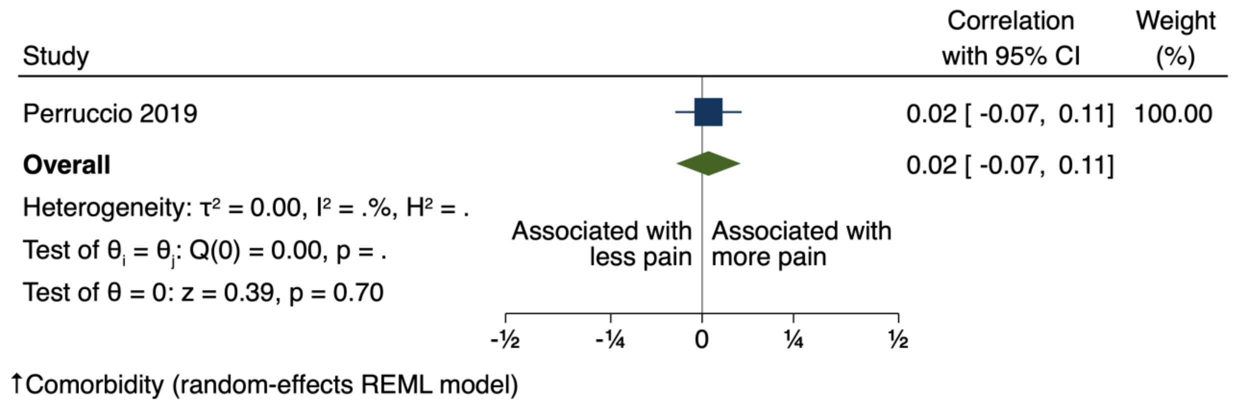

S12 Fig. ↑BMI

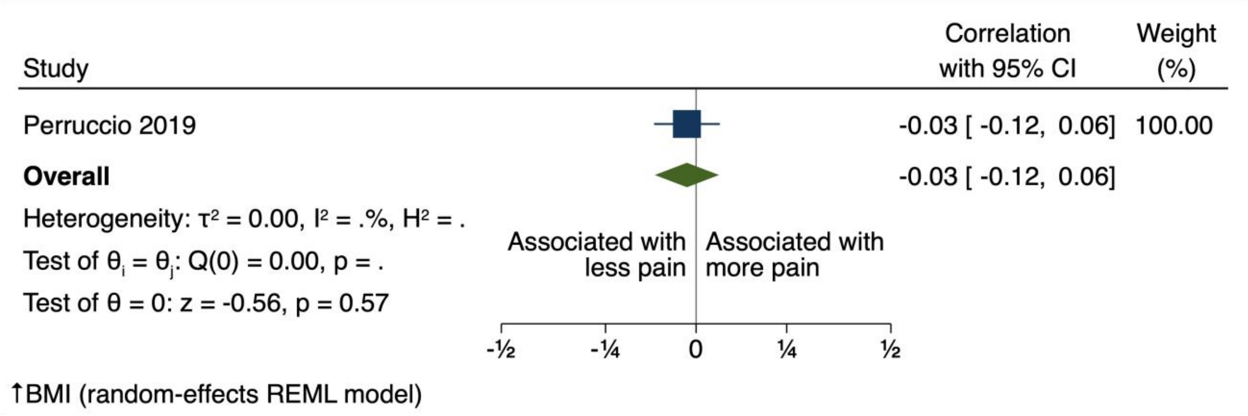

S13 Fig. ↑Cortisol

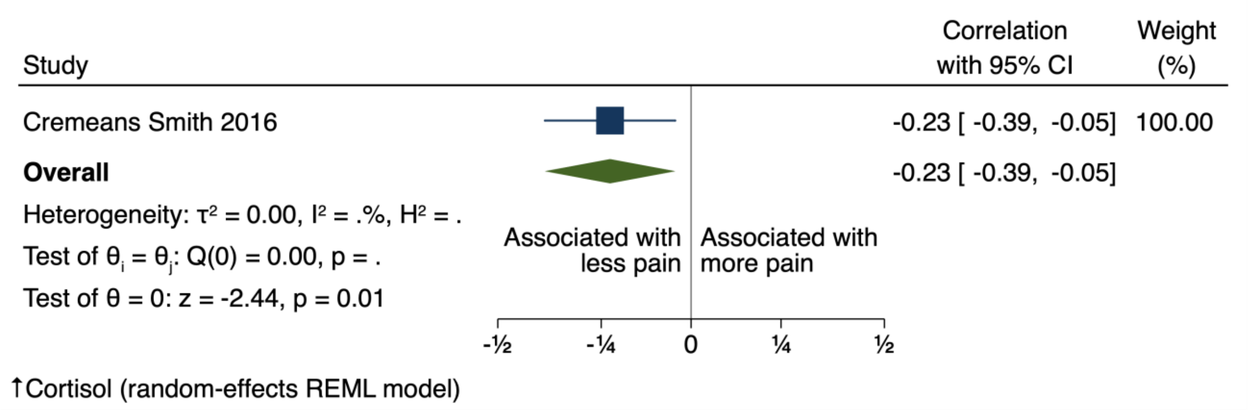

S14 Fig. ↑Low Back Pain

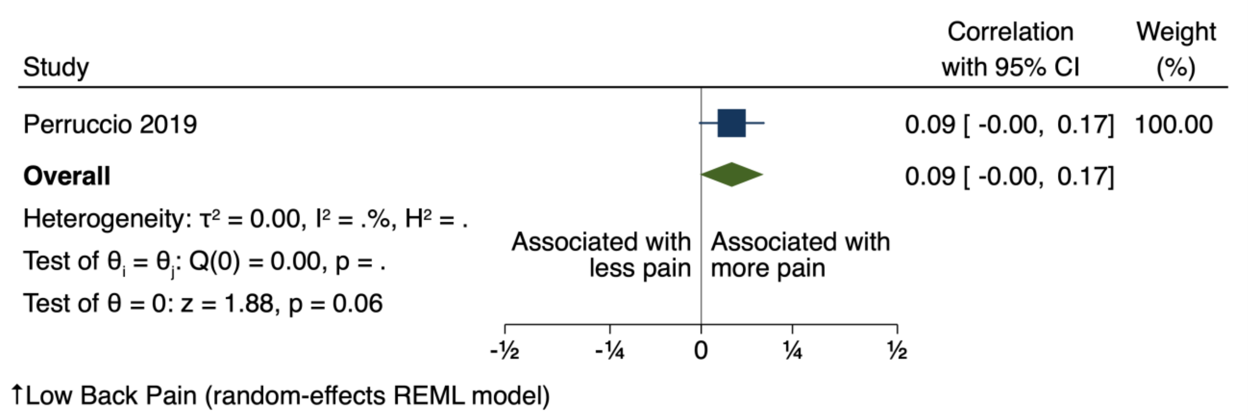

S15 Fig. Male Gender

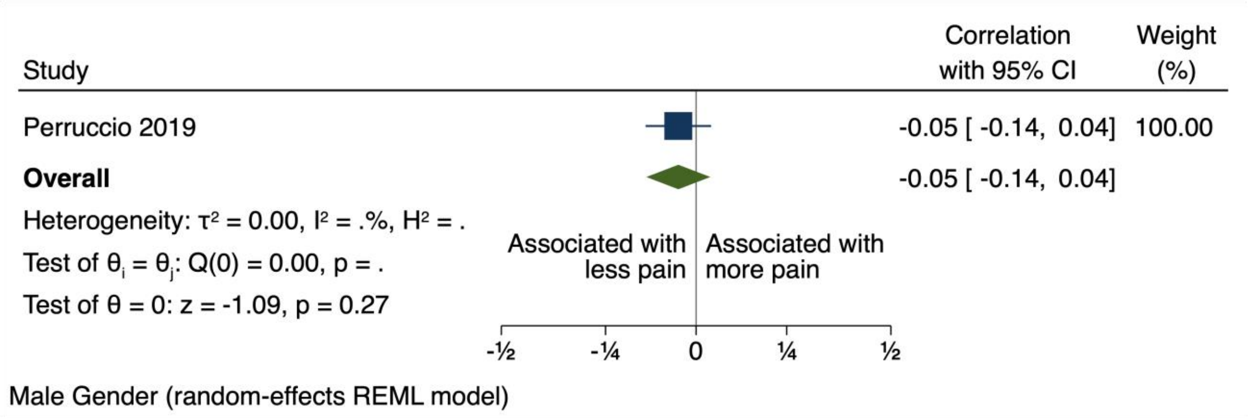

S16 Fig. 16. ↑Daytime Sleepliness

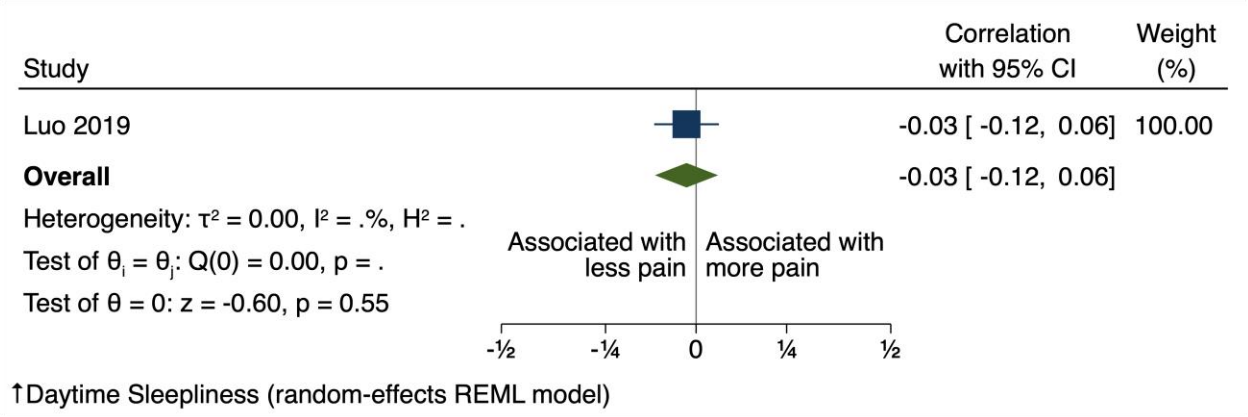

S17 Fig. 17. ↑Sleep Dysfunction

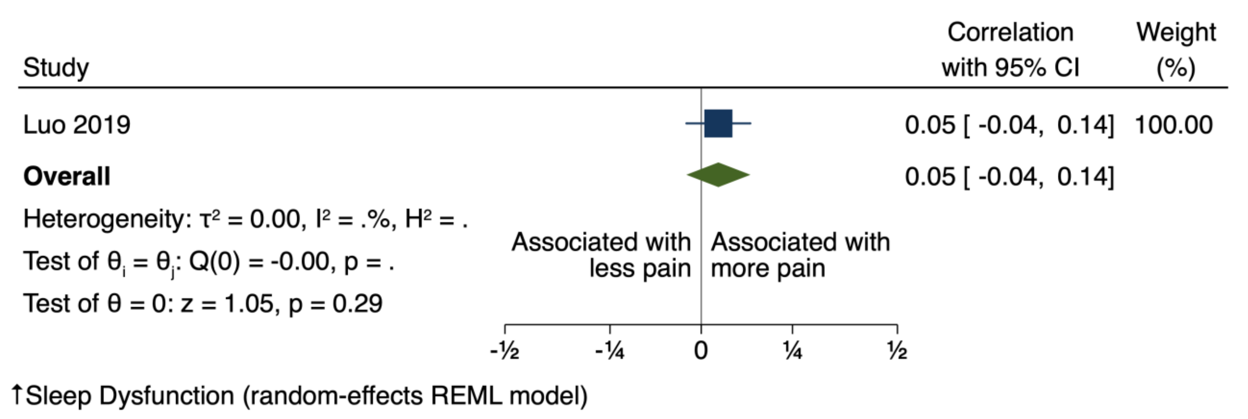

S18 Fig. ↑Age

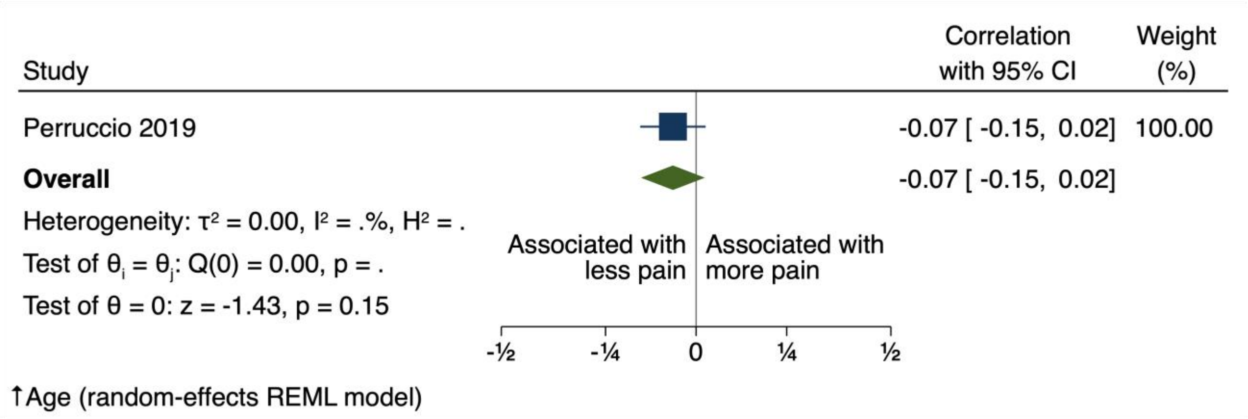

S19 Fig. ↑Preoperative Pain

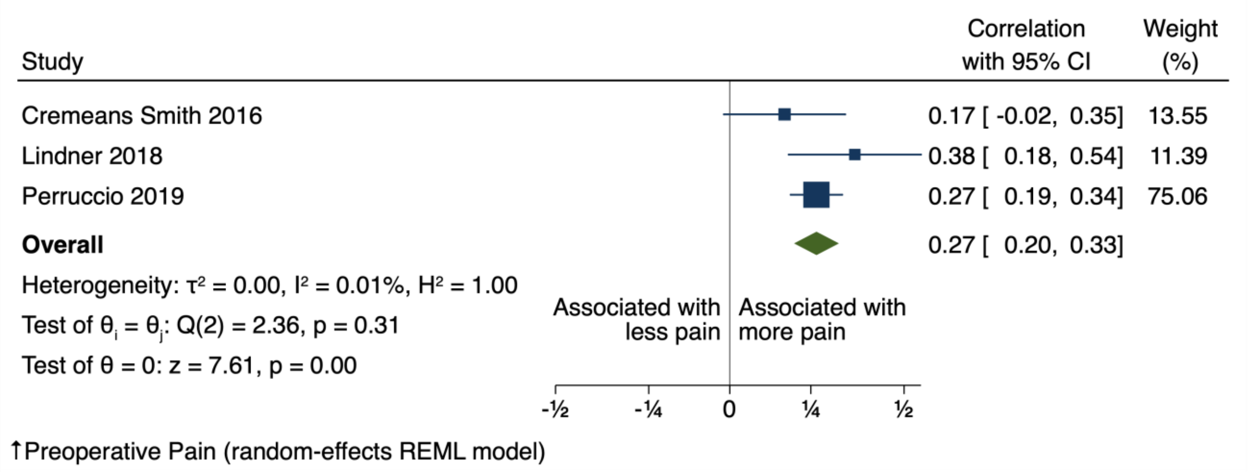

S20 Fig. Spinal Anesthesia

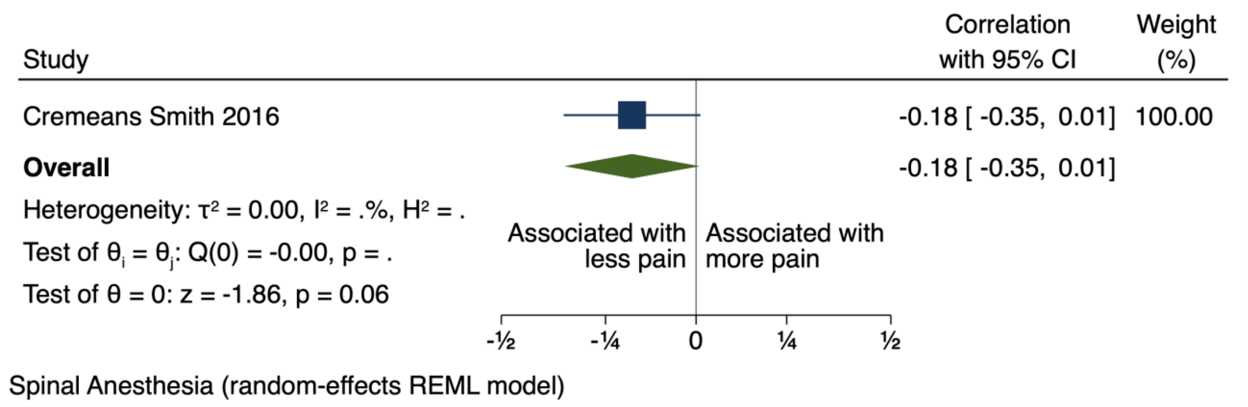

S21 Fig. ↑Symptomatic Joints

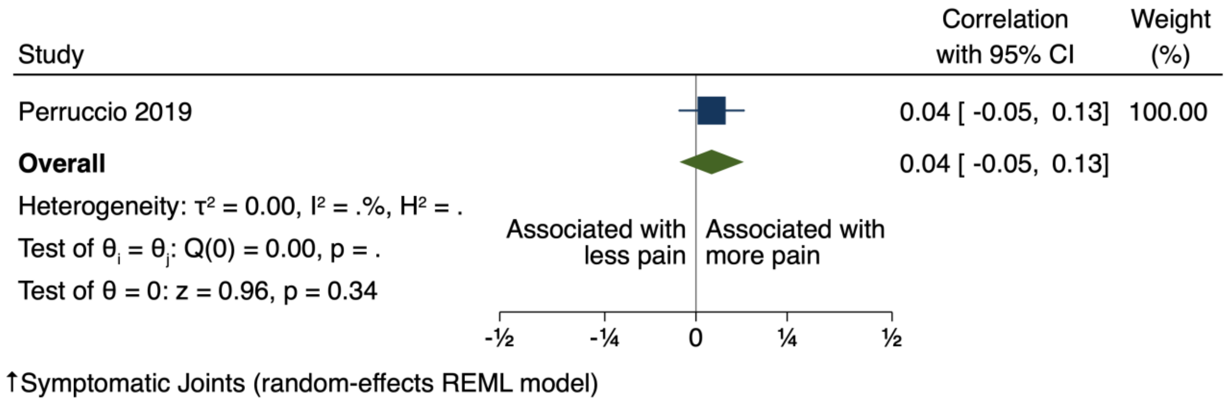

S22 Fig. Worse Sleep Quality

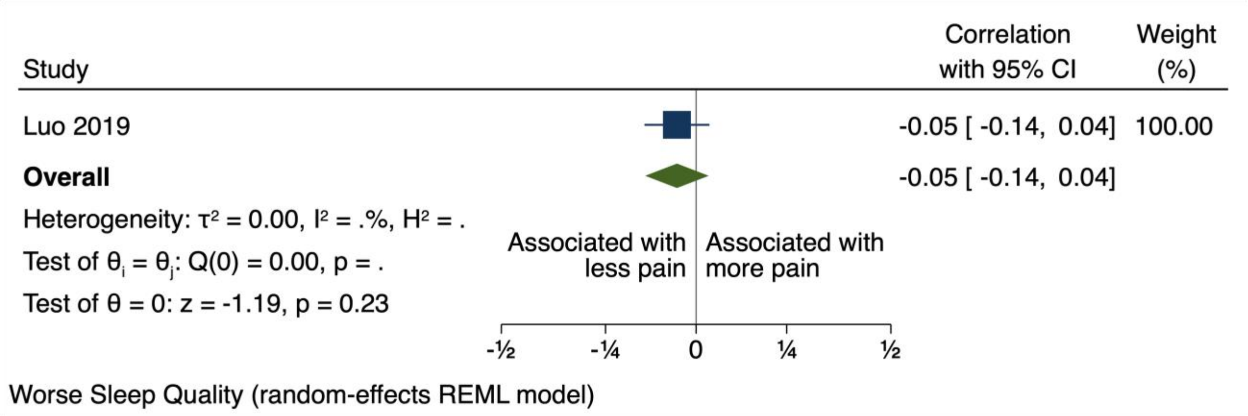

Pain (6 months)

S23 Fig. ↑Agreeableness

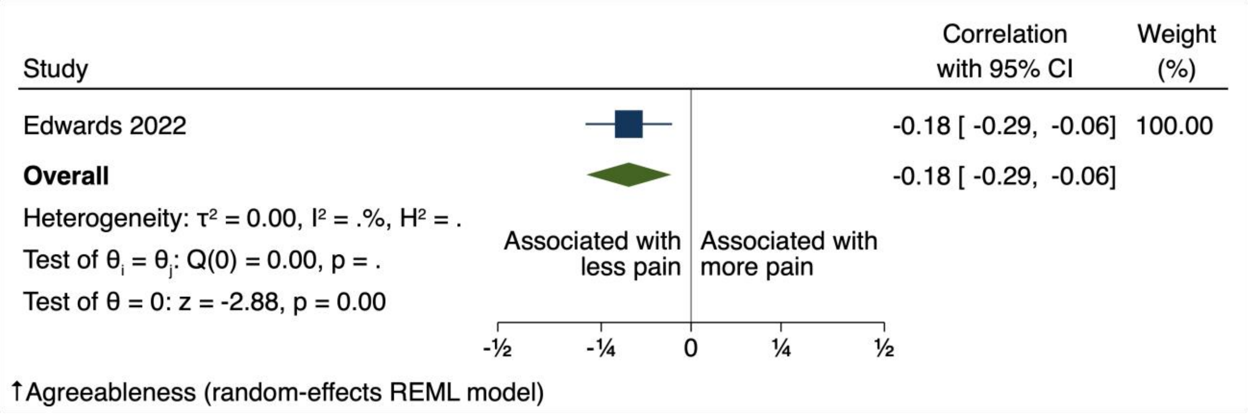

S24 Fig. ↑Arthritis Helplessness

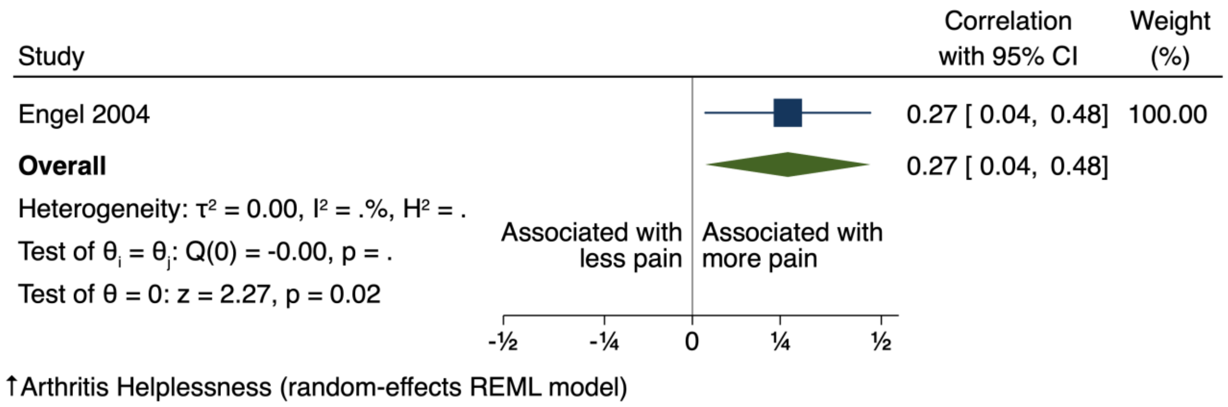

S25 Fig. ↑Education

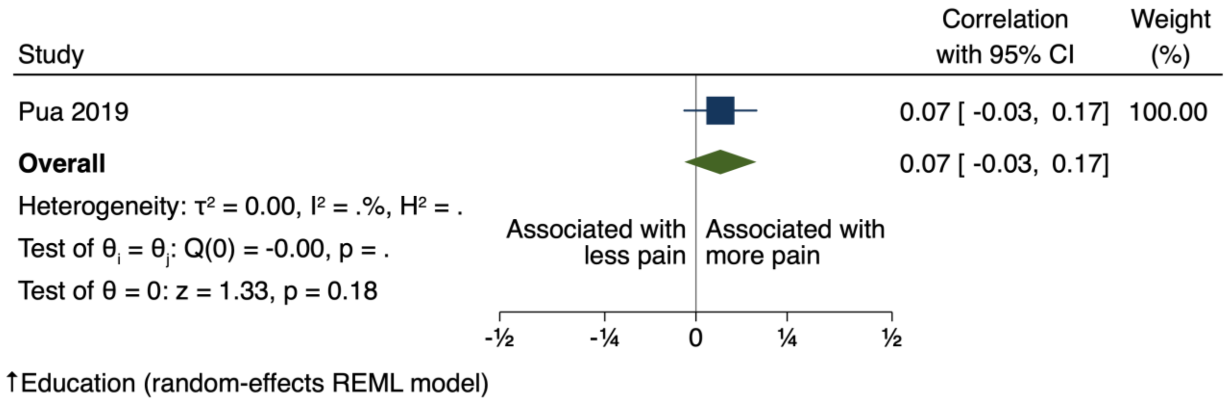

S26 Fig. ↑Mental Health

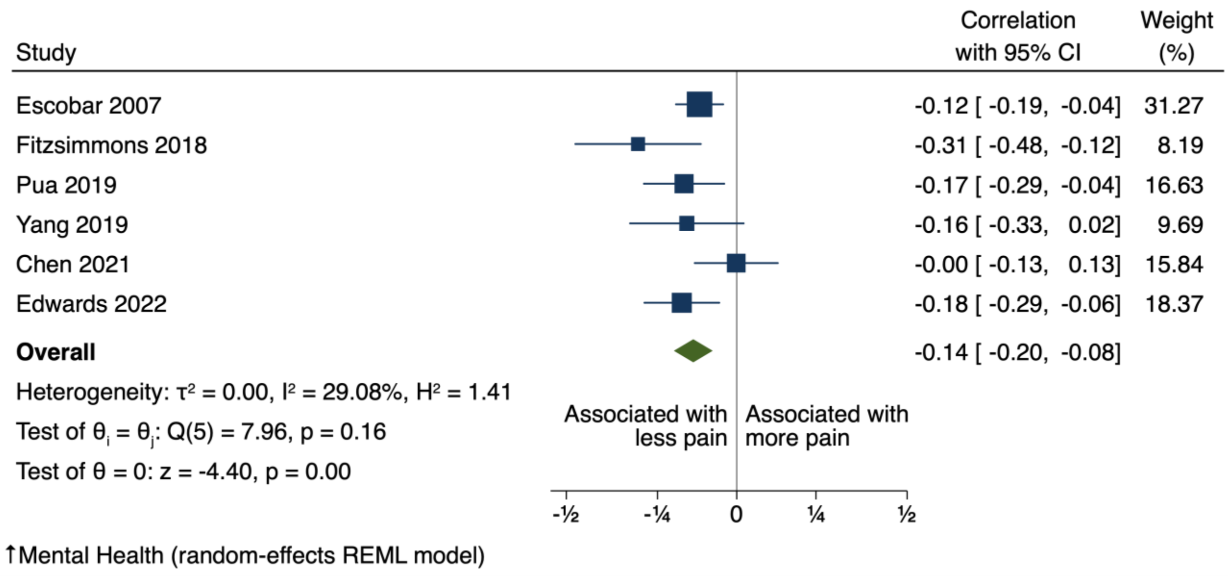

S 27 Fig. ↑Preoperative Function

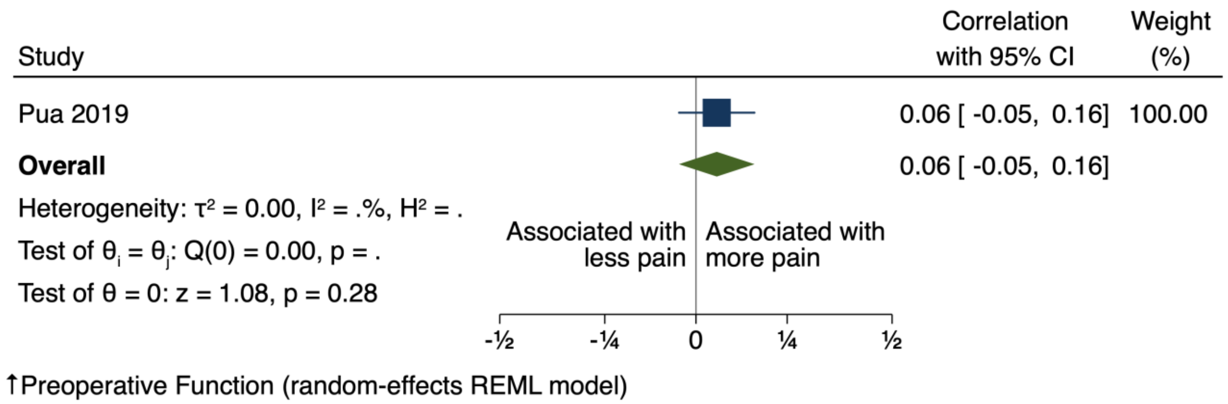

S28 Fig. ↑Catastrophizing

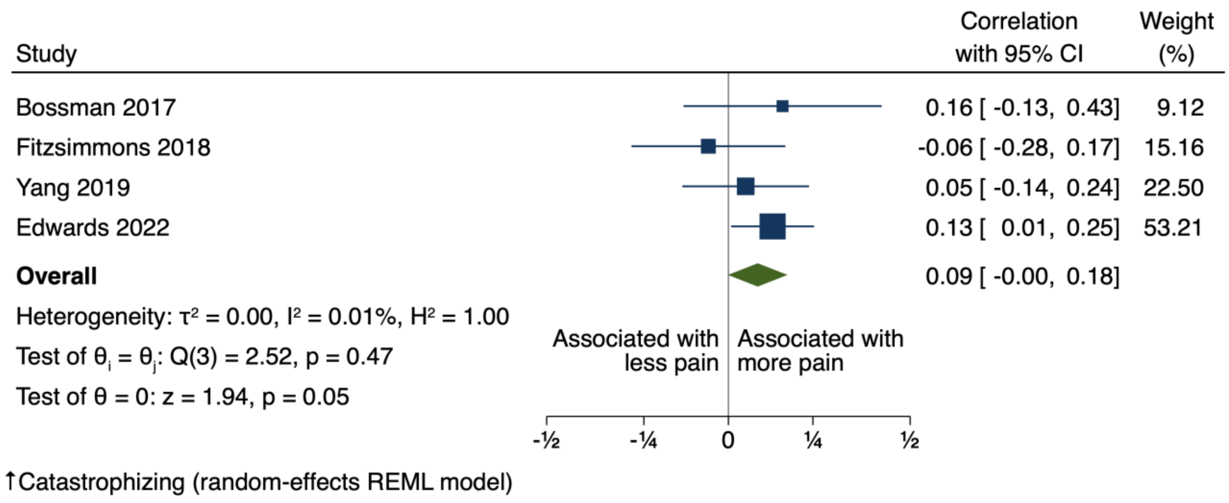

S29 Fig. Chinese Ethnicity

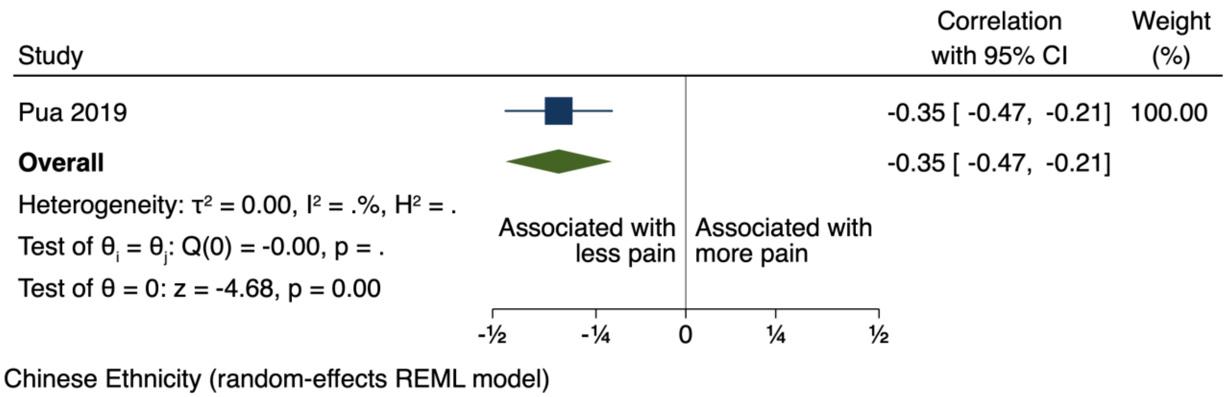

S30 Fig. ↑Comorbidity

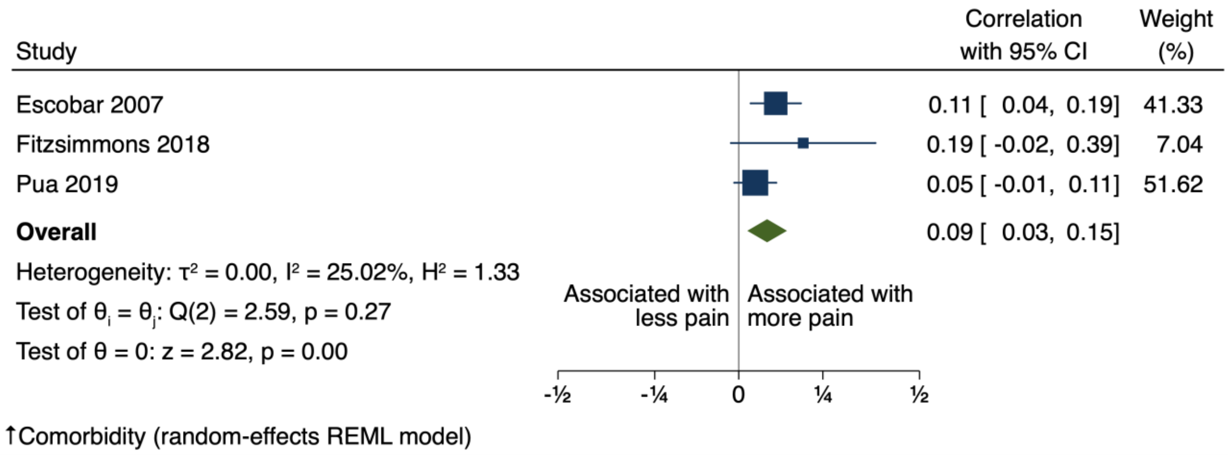

S31 Fig. ↑Pain Modulation

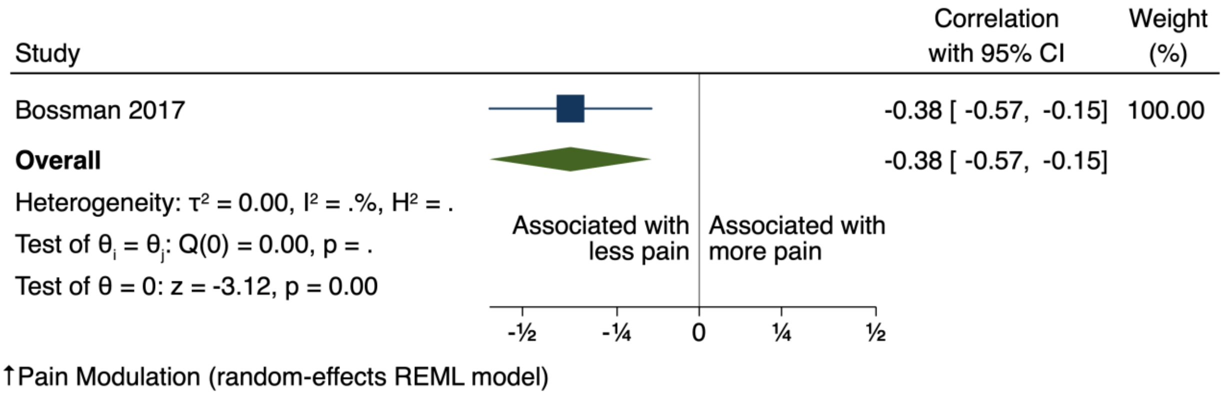

S32 Fig. ↑Contralateral Knee Pain

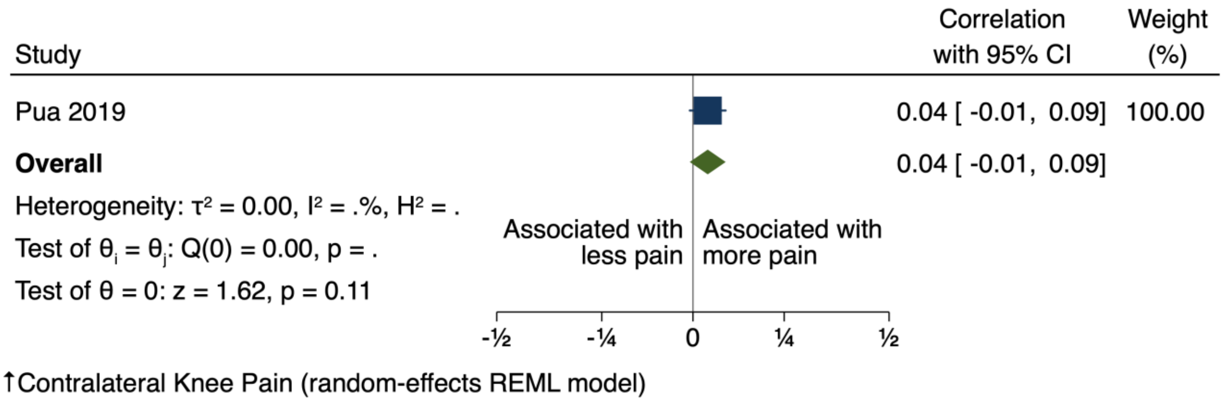

S33 Fig. ↑Heart Rate Variability

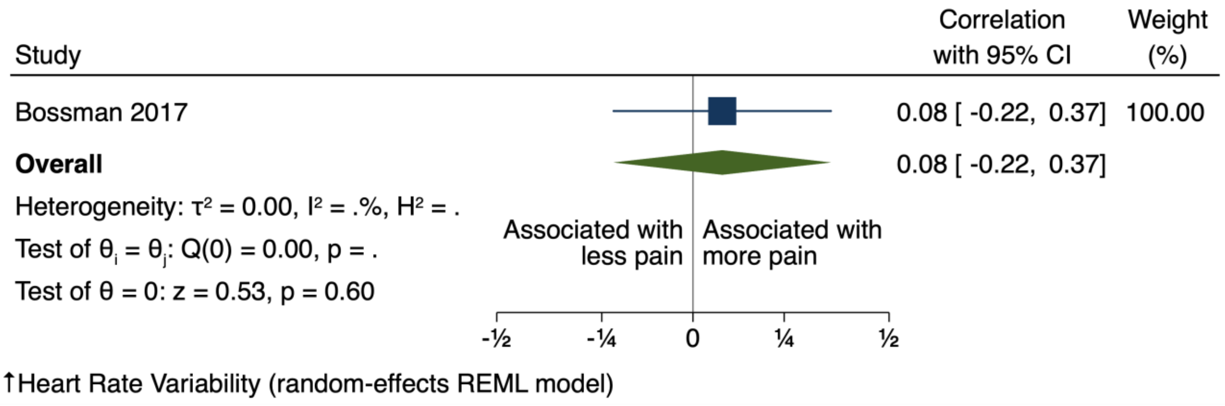

S34 Fig. ↑AT2R Level

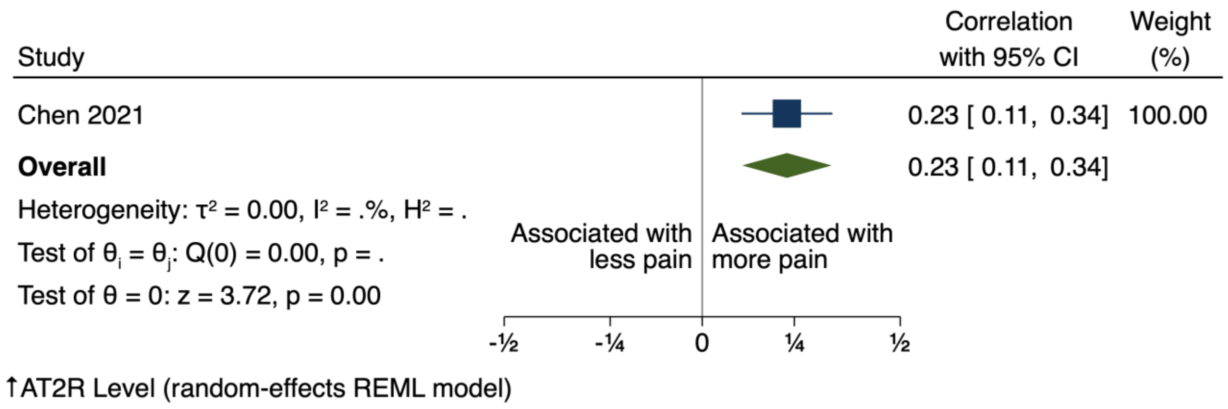

S35 Fig. ↑BMI

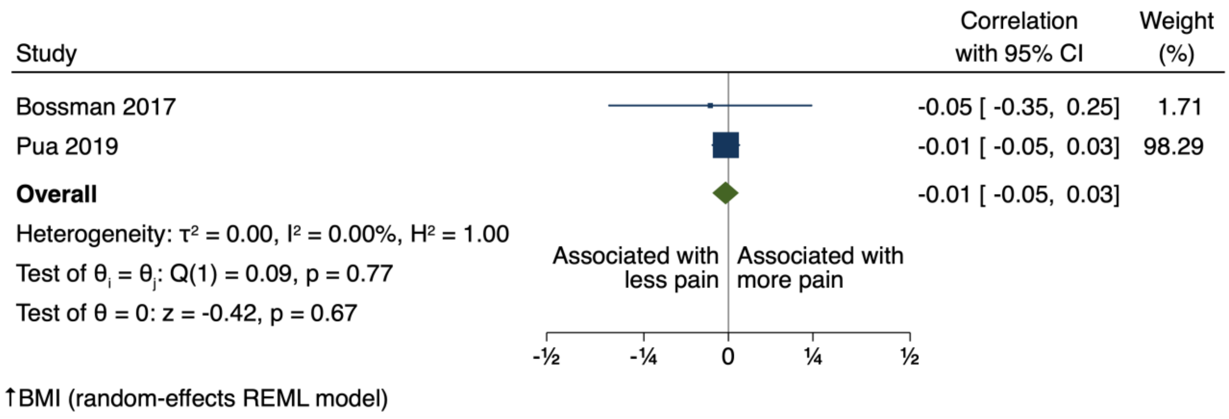

S36 Fig. ↑Sleep Effiency

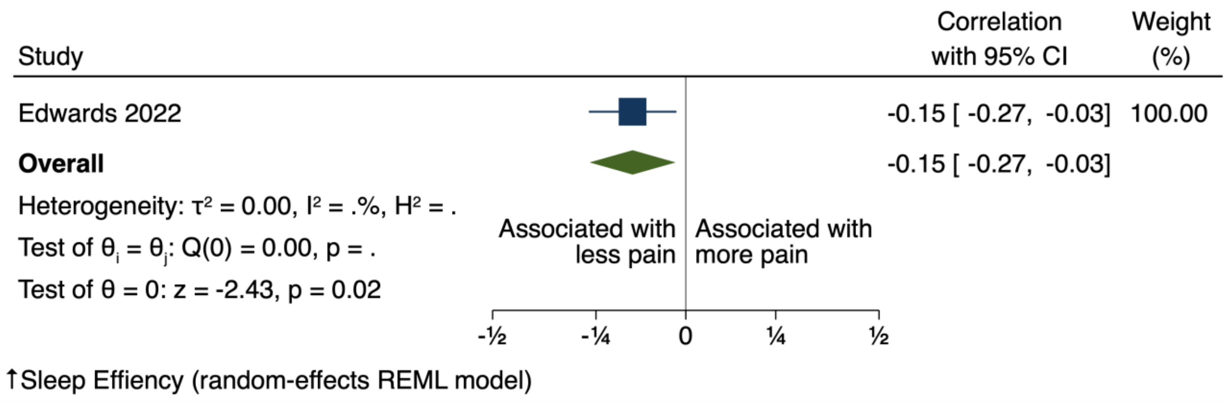

S37 Fig. ↑Oxidative Stress

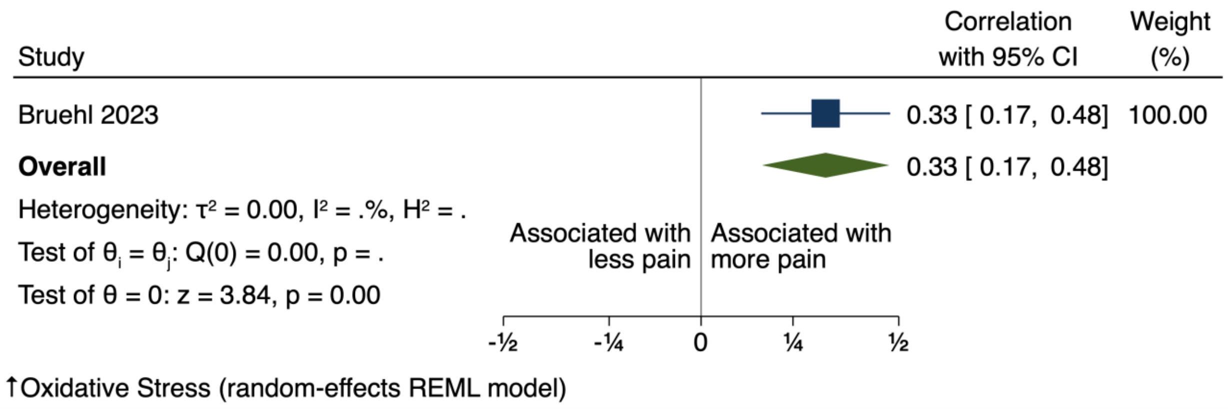

S38 Fig. ↑Ischemia Duration

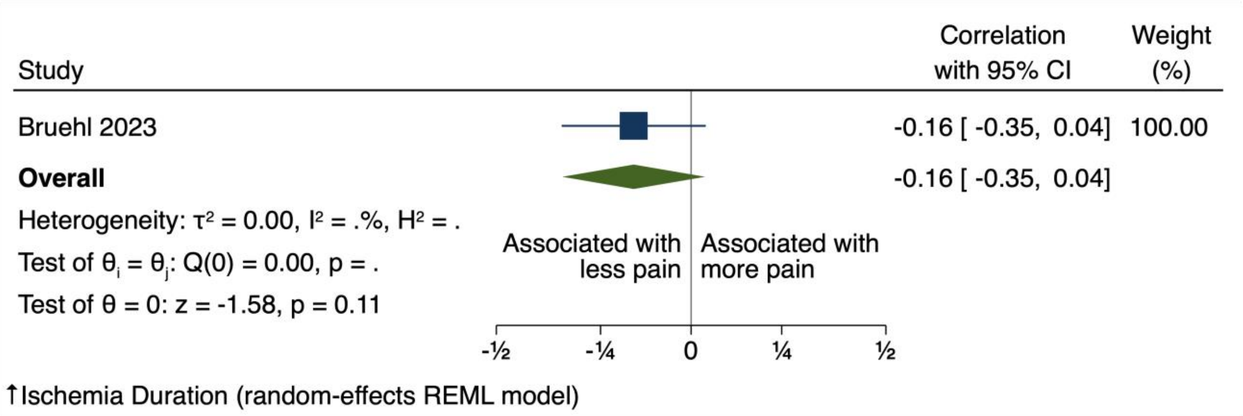

S39 Fig. ↑Trail Making Time

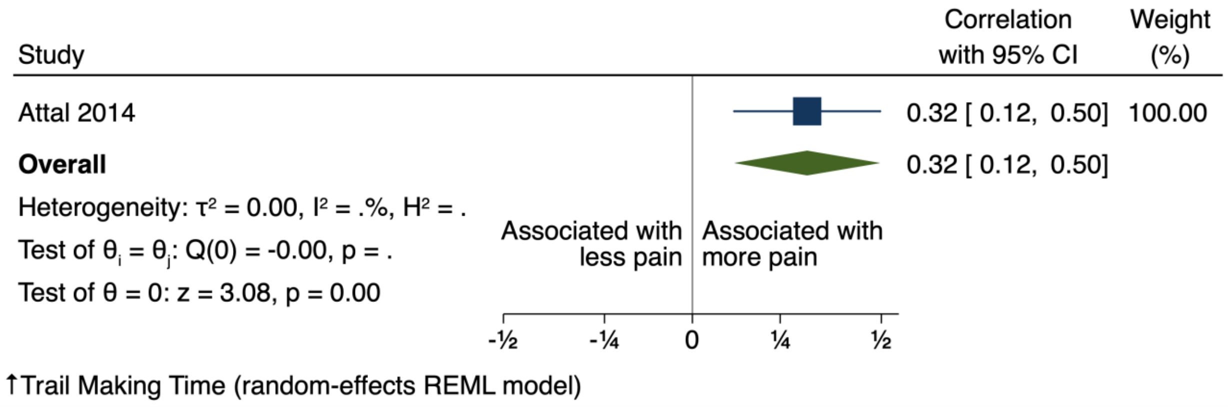

S40 Fig. ↑Low Back Pain

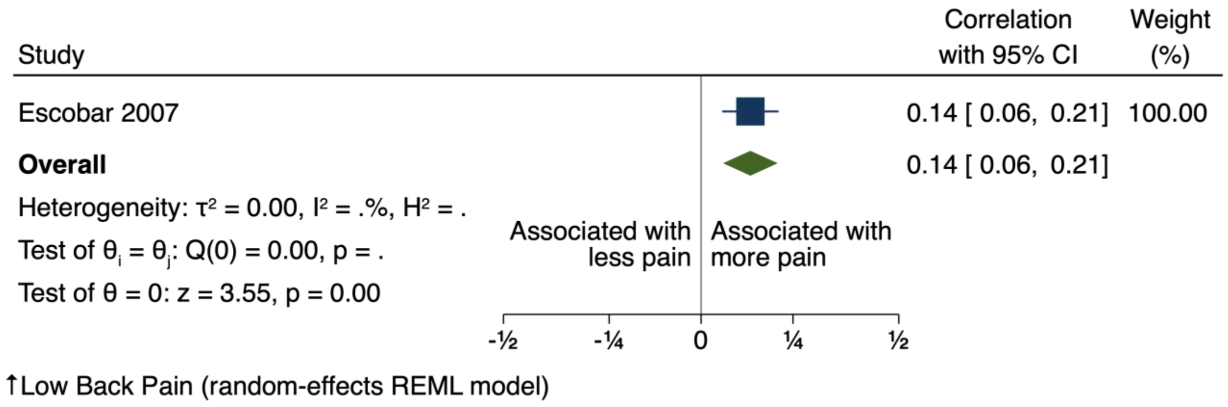

S41 Fig. Male Gender

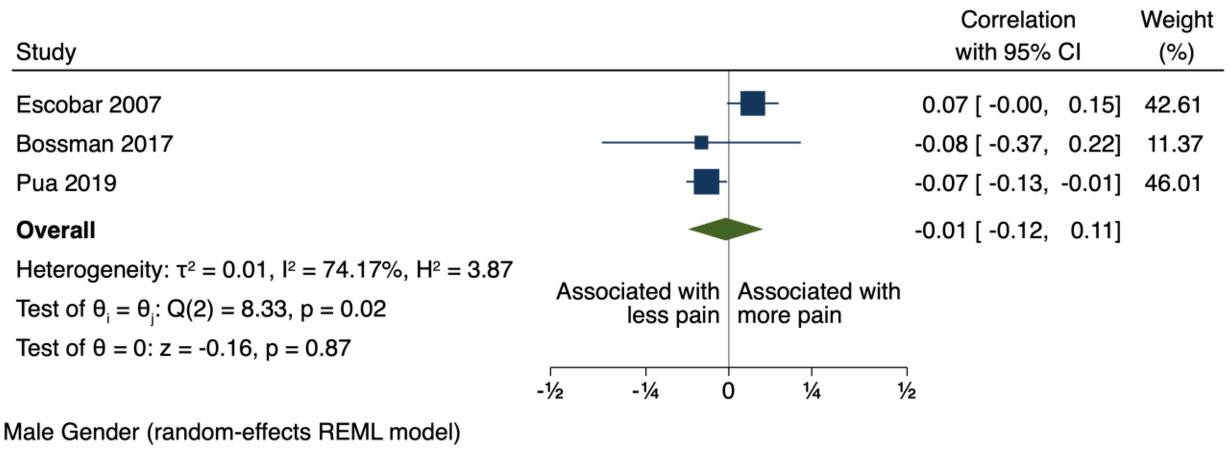

S42 Fig. ↑Knee Extension

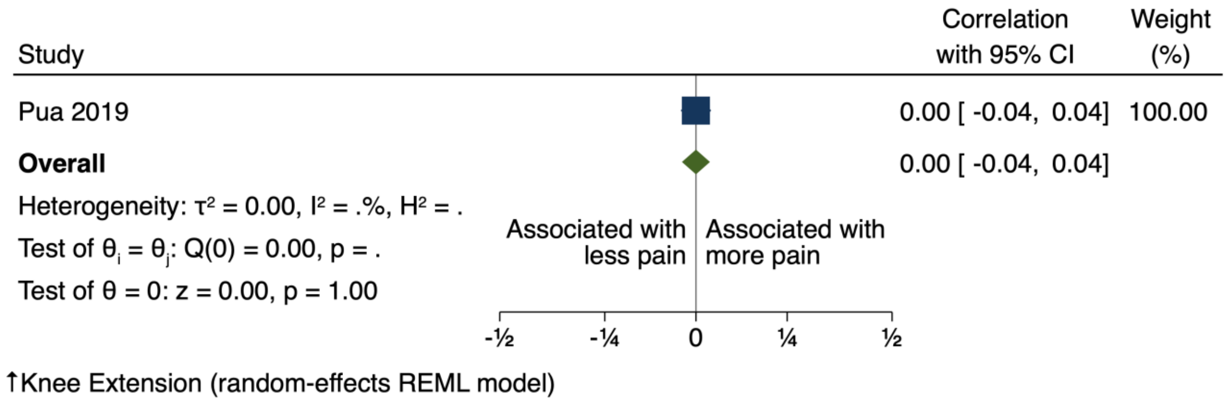

S43 Fig. ↑Knee Flexion

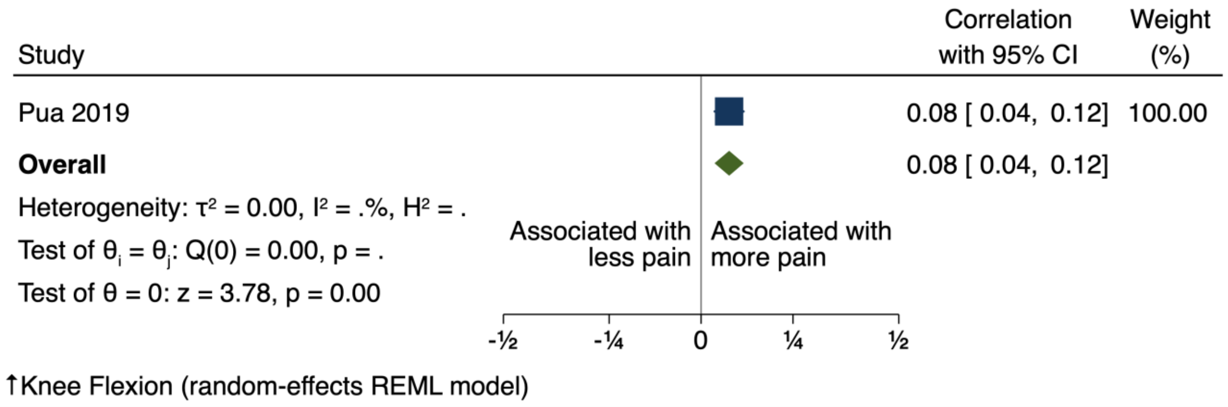

S44 Fig. ↑Walking Aid Use

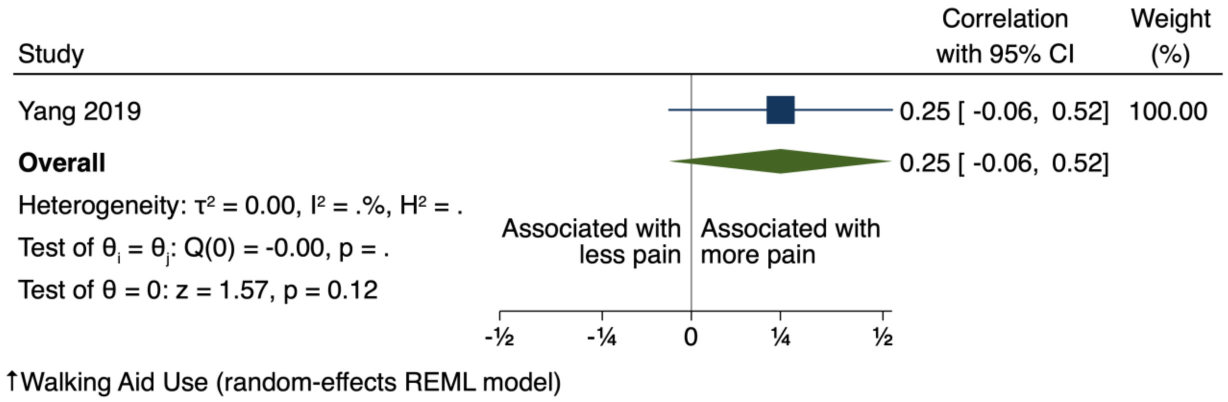

S45 Fig. ↑Age

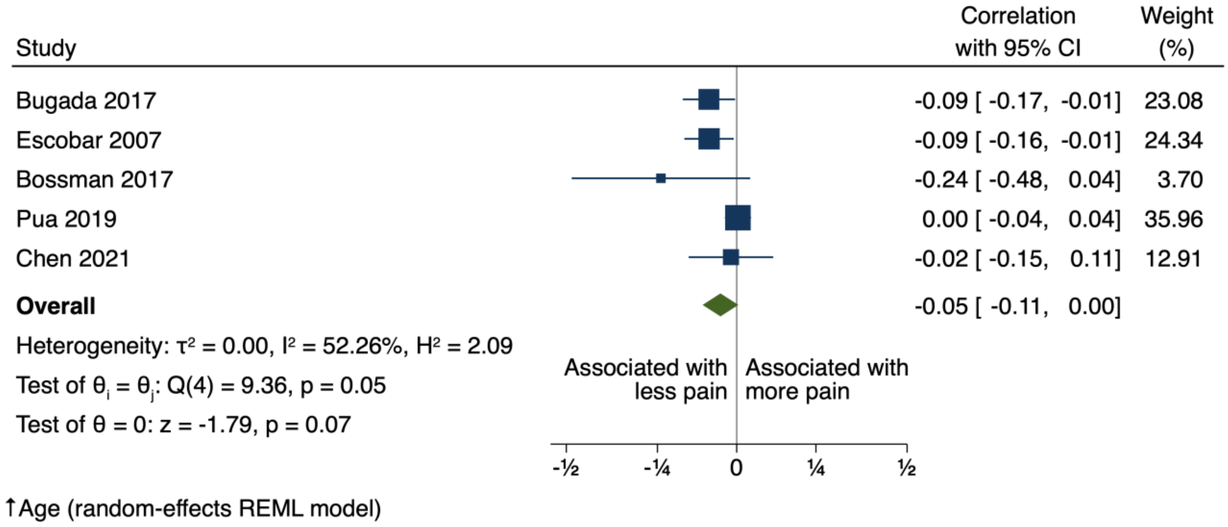

S46 Fig. Opioid Use

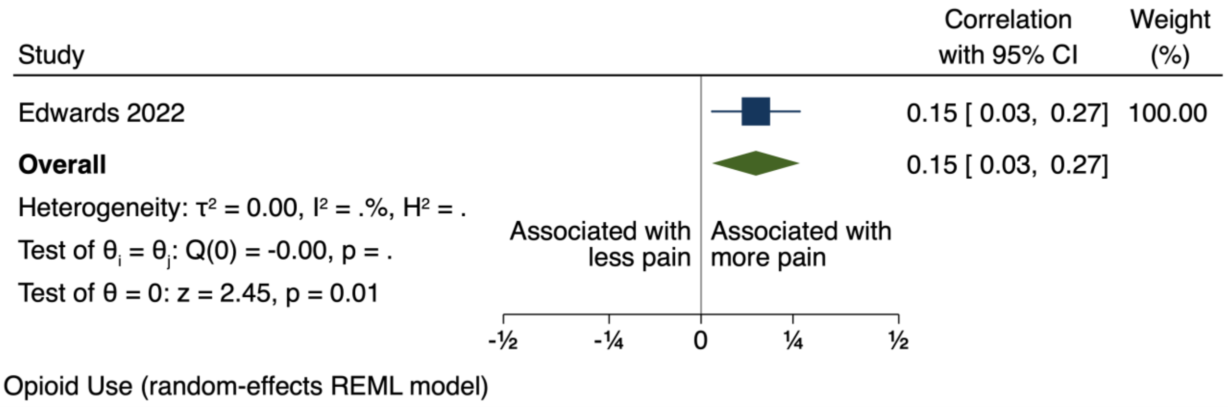

S47 Fig. Other Chronic Pain

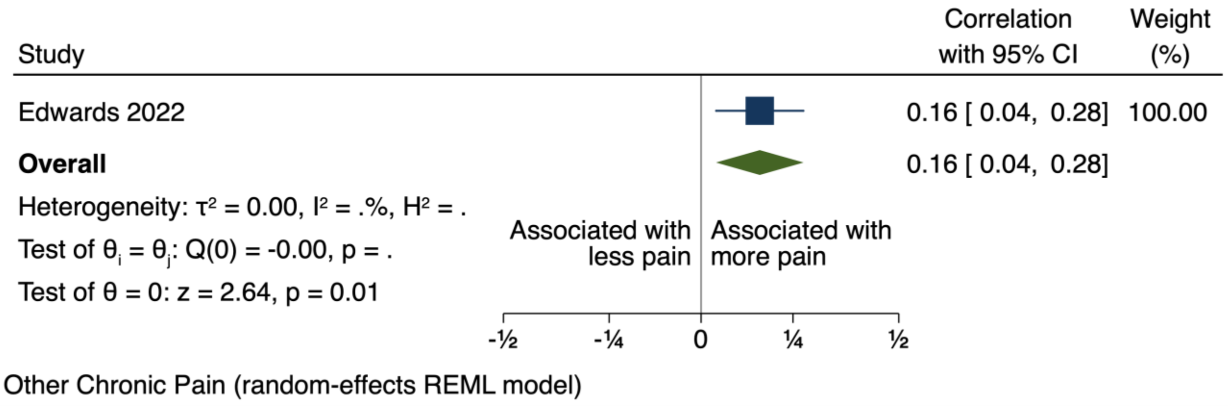

S48 Fig. ↑Disability

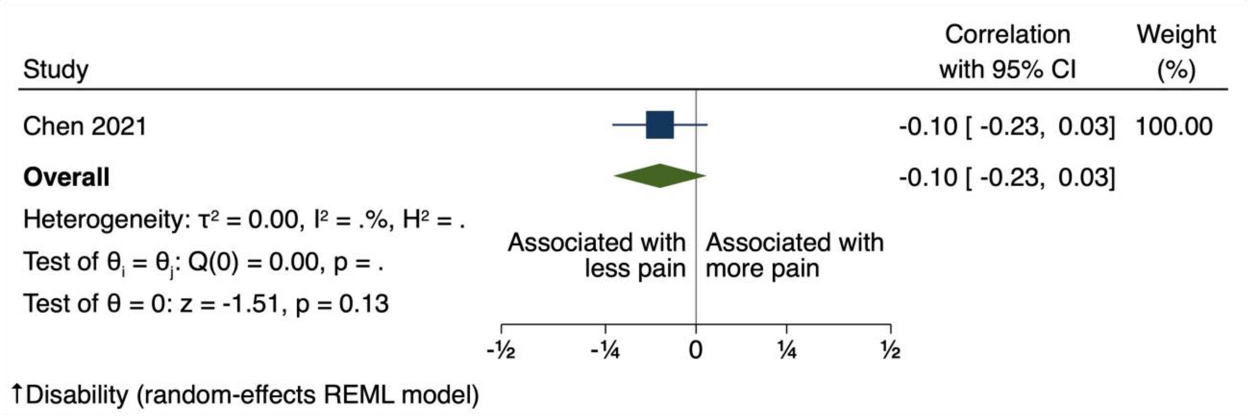

S49 Fig. ↑Pain Expected

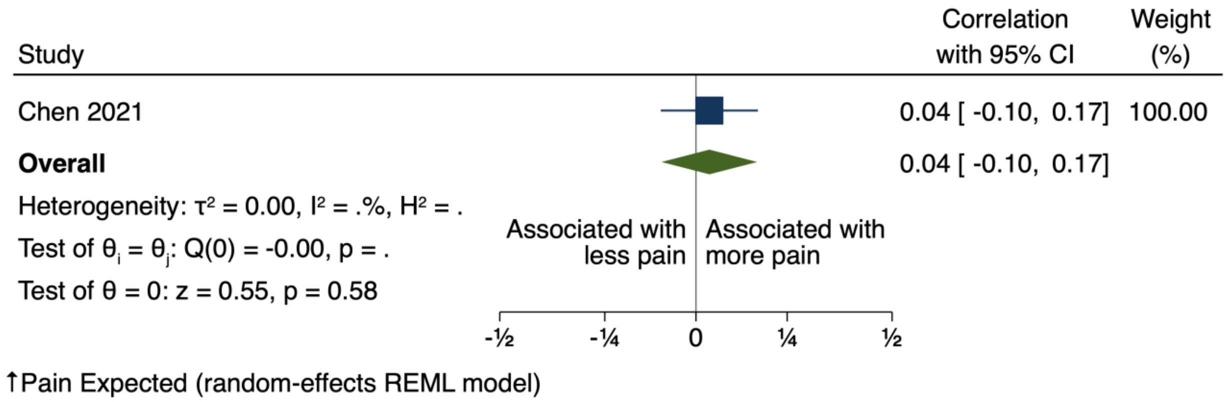

S50 Fig. ↑Pain Self-Efficacy

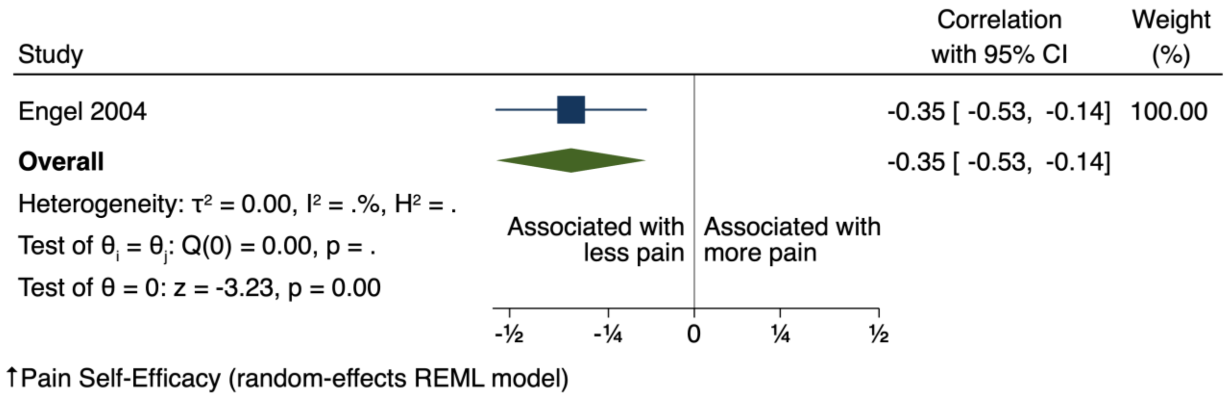

S51 Fig. ↑Preoperative Pain

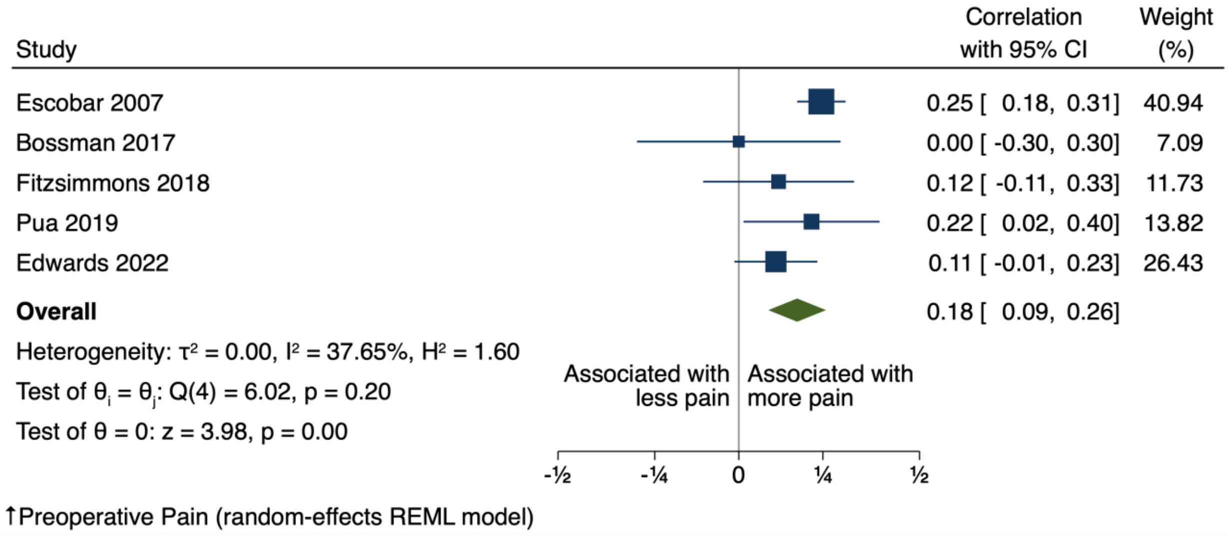

S52 Fig. ↑Social Support

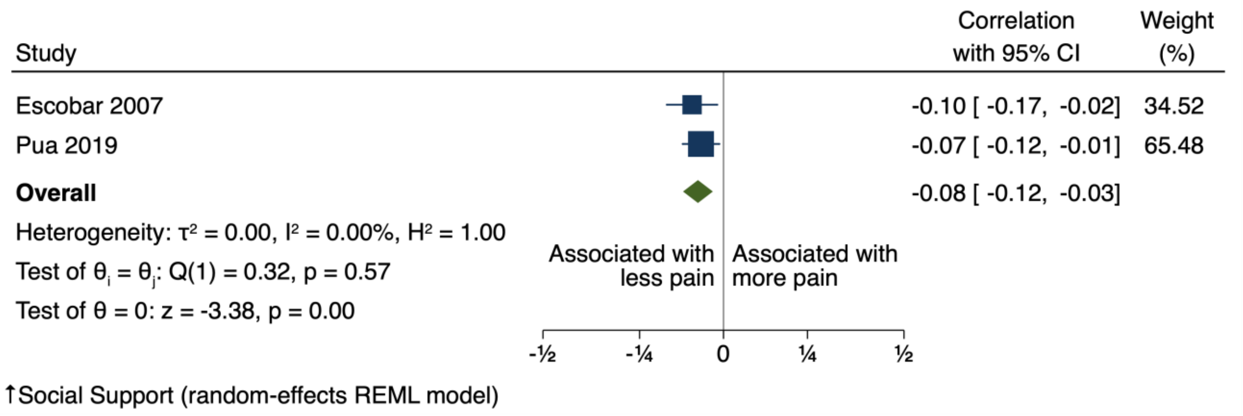

S53 Fig. Suspected Neuropathic Pain

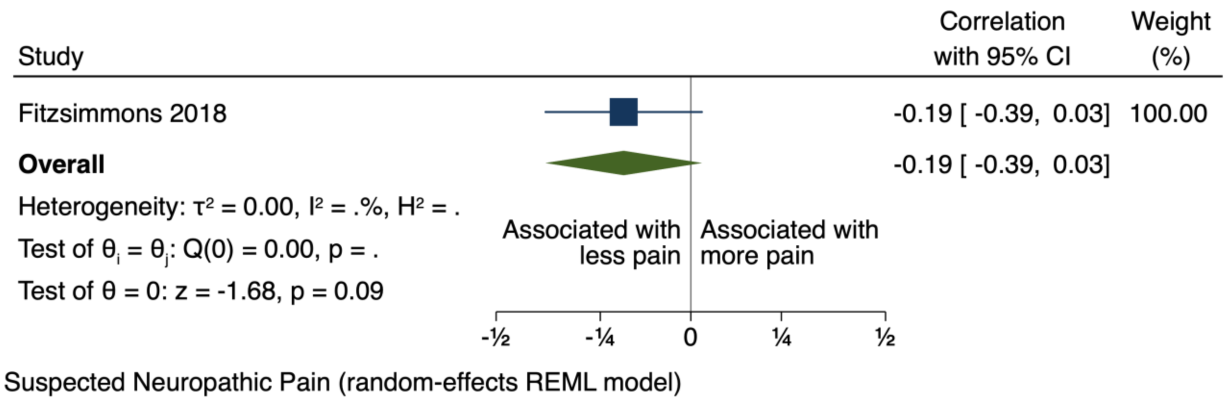

S54 Fig. ↑Sym/Parasym Activity

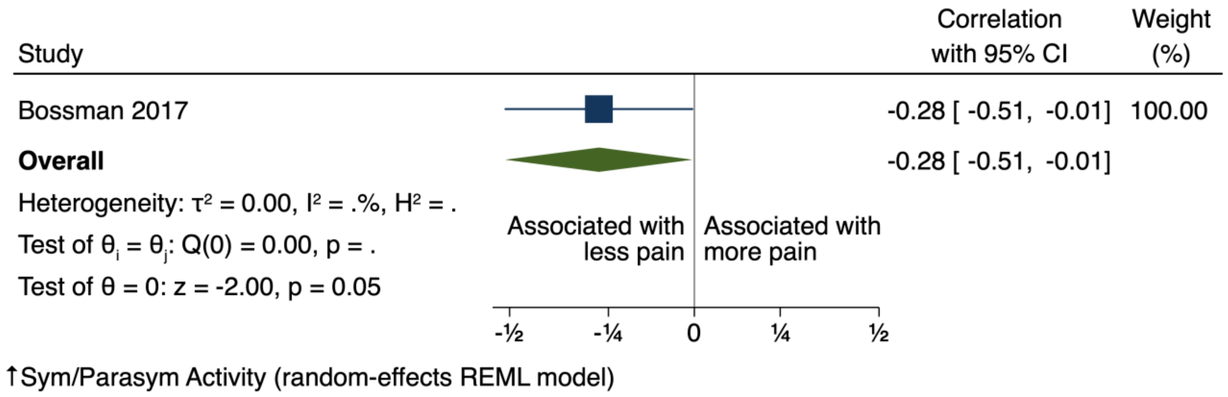

S55 Fig. ↑Symptomatic Joints

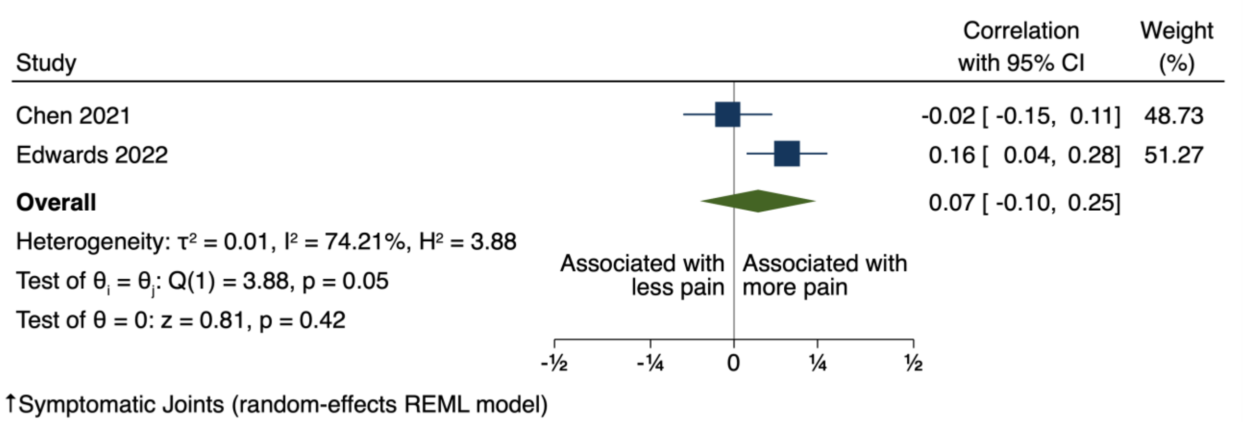

S56 Fig. ↑Temporal Summation

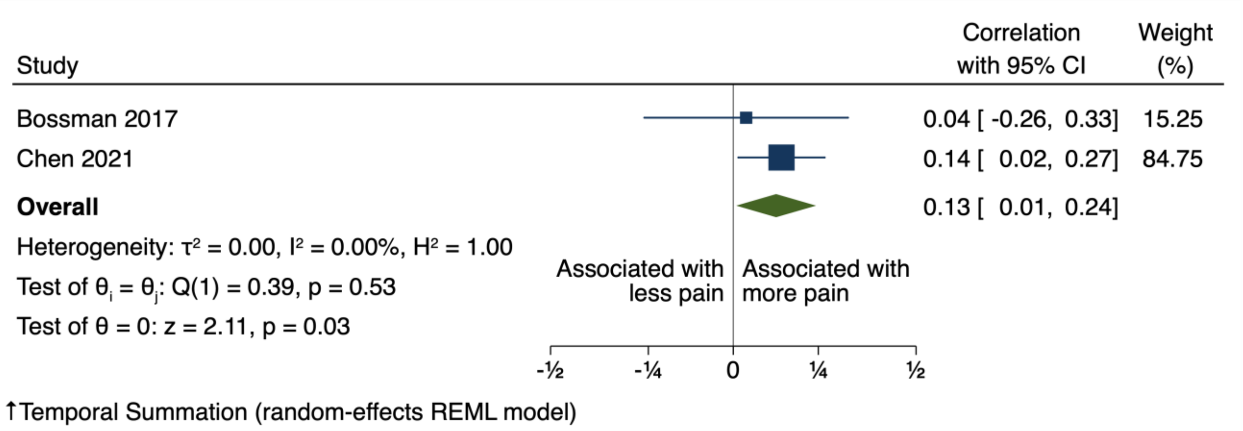

Pain (12 months)

S57 Fig. ↑Mental Health

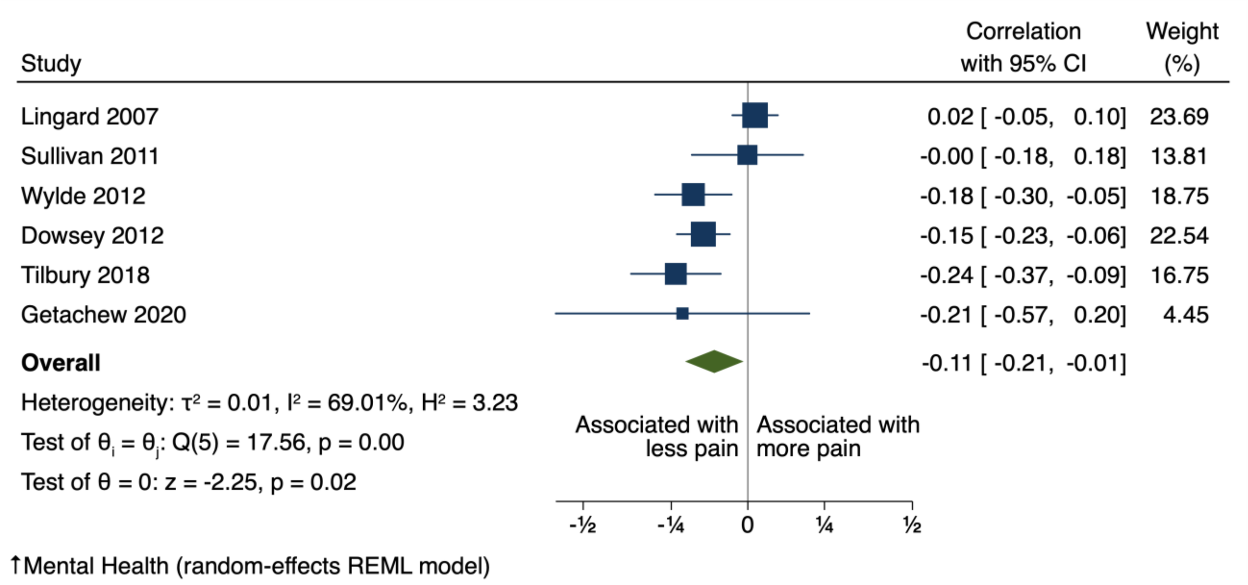

S58 Fig. ↑Outcome Expected

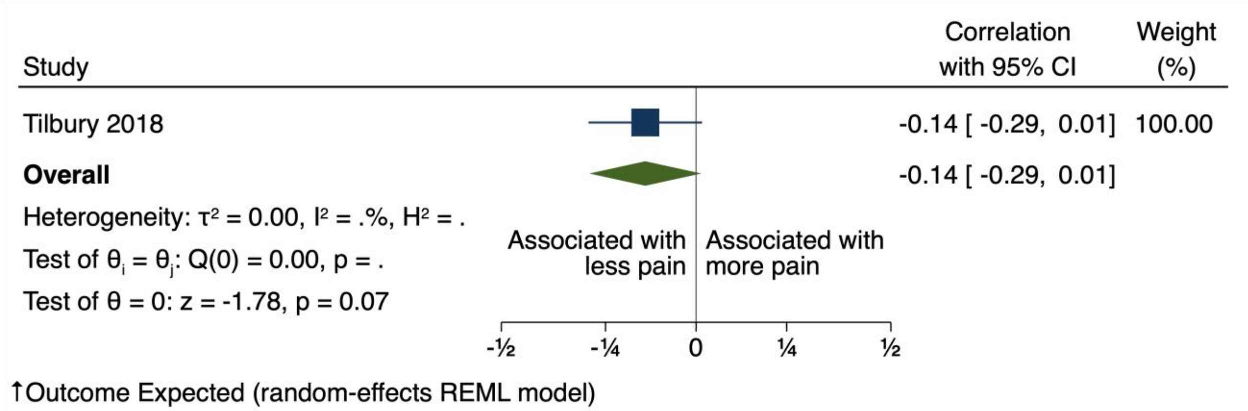

S59 Fig. ↑Preoperative Function

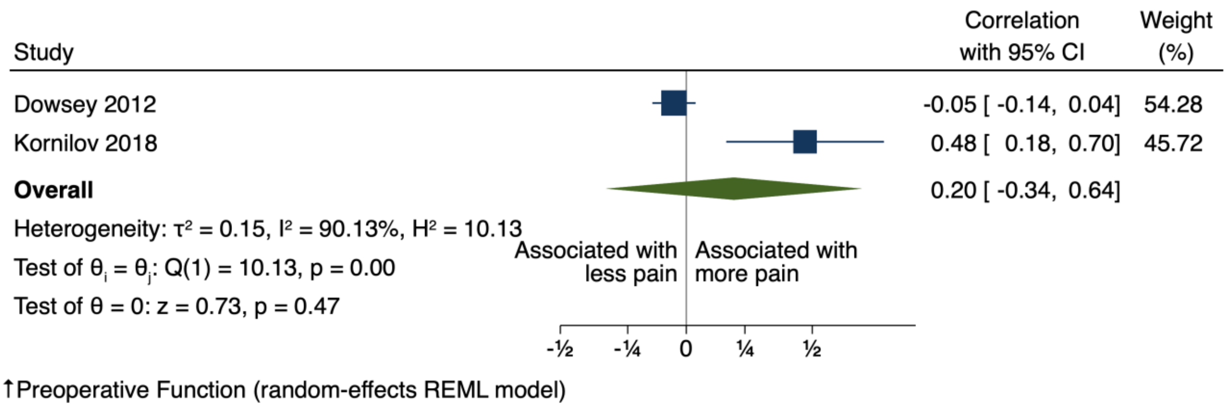

S60 Fig. ↑ROCF Recall

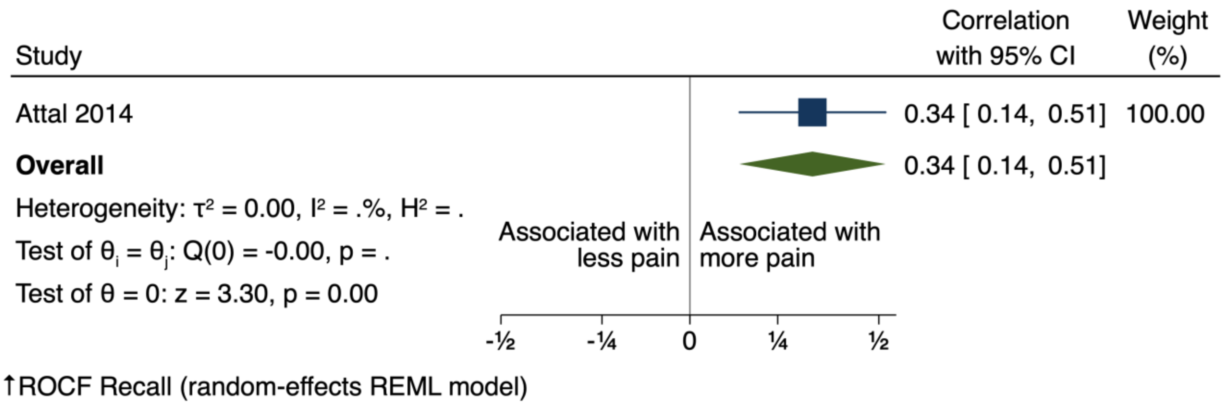

S61 Fig. ↑Catastrophizing

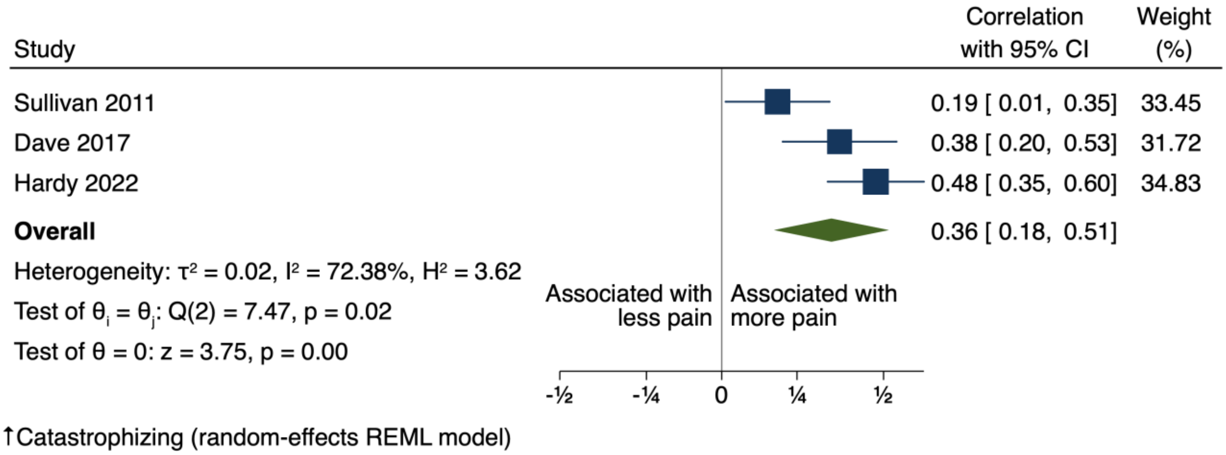

S62 Fig. ↑Comorbidity

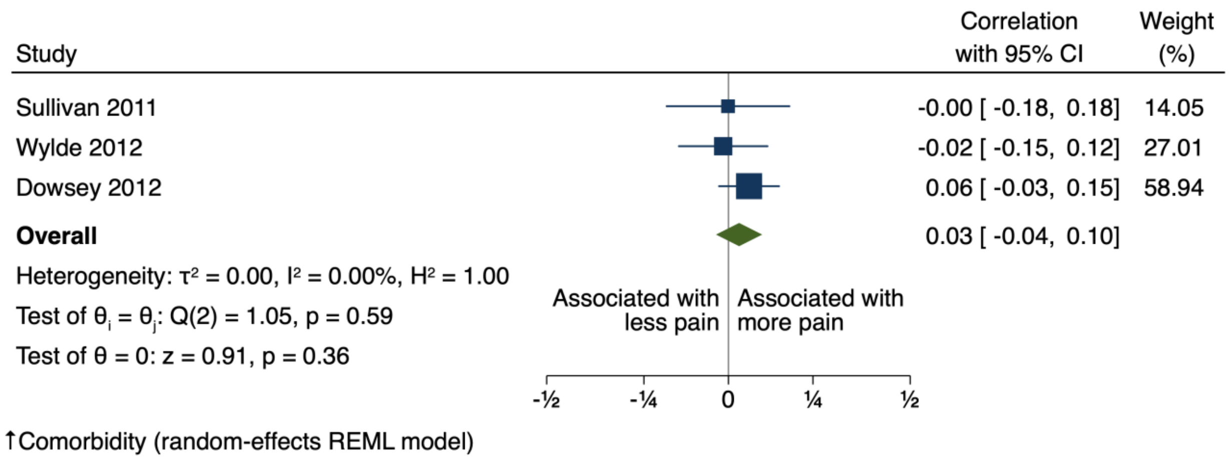

S63 Fig. Cruciate Retaining

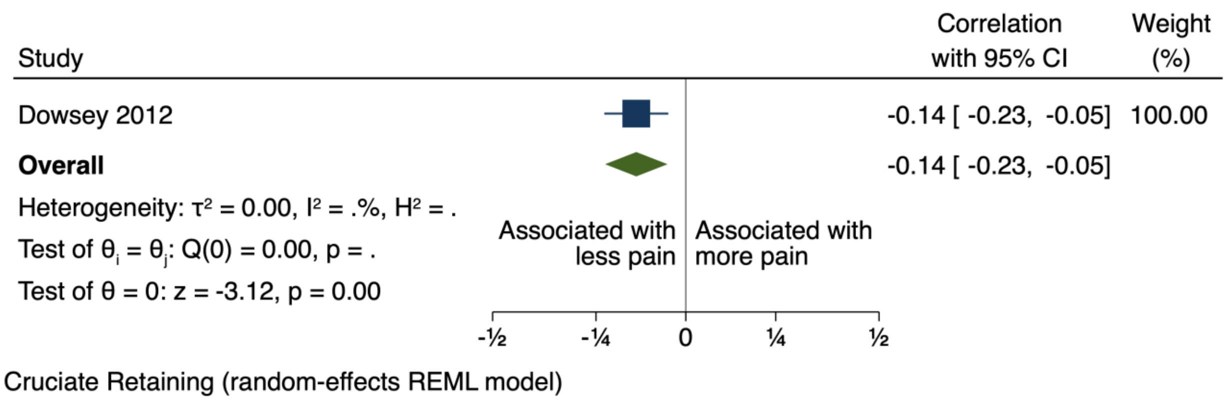

S64 Fig. ↑Synovial Perfusion (Degree)

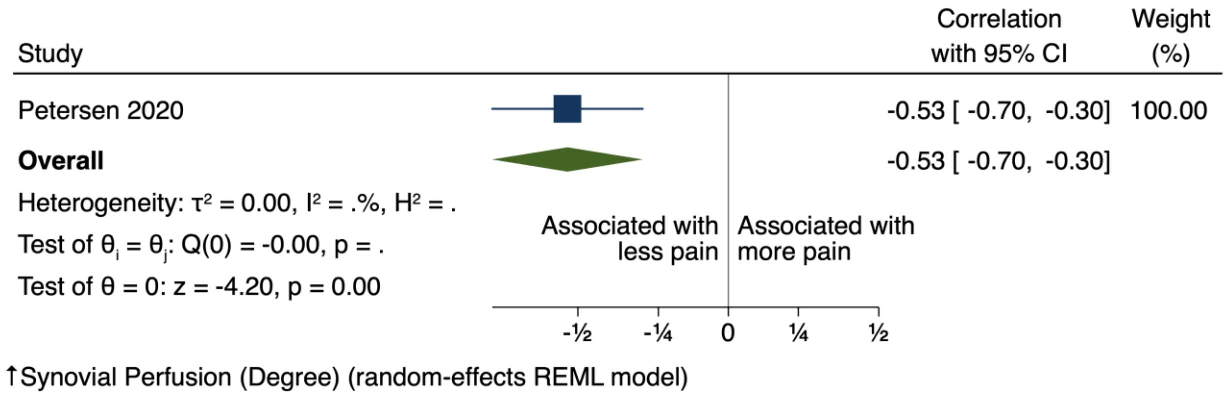

S65 Fig. ↑Fatigue

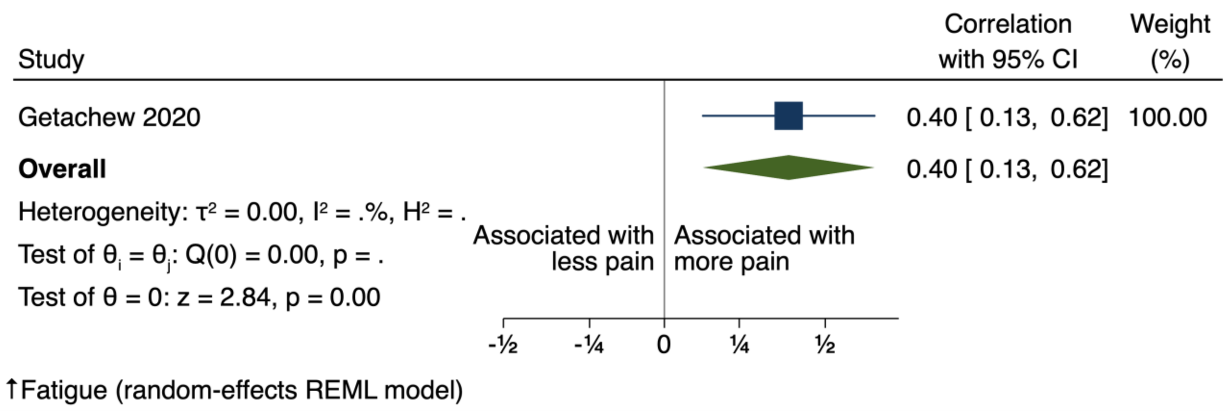

S66 Fig. ↑BMI

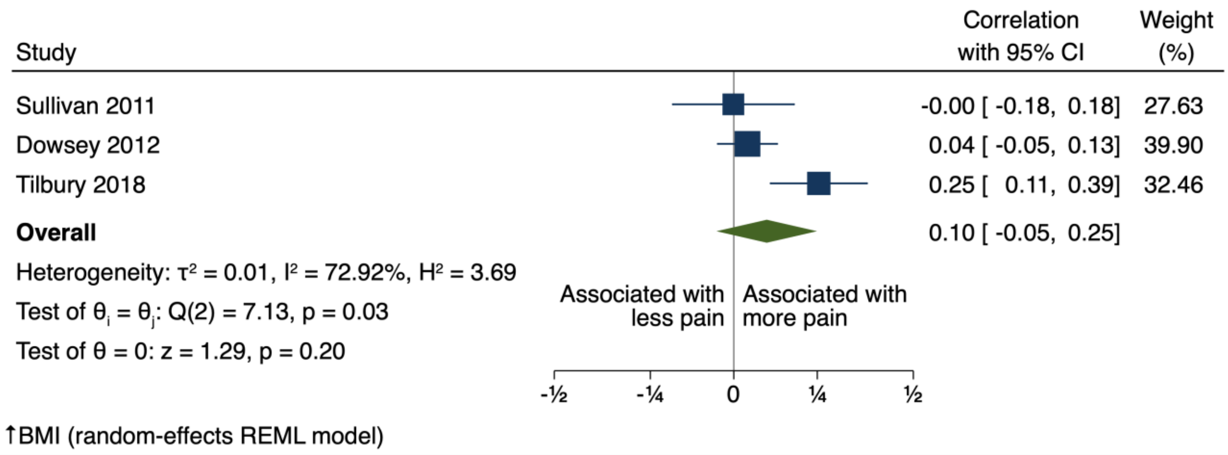

S67 Fig. ↑Heat Pain Threshold

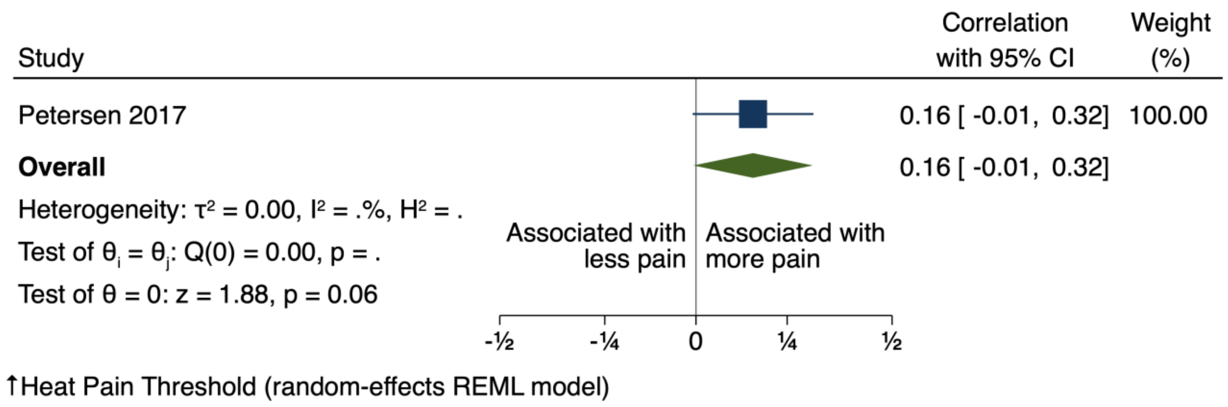

S68 Fig. ↑K-L Grade

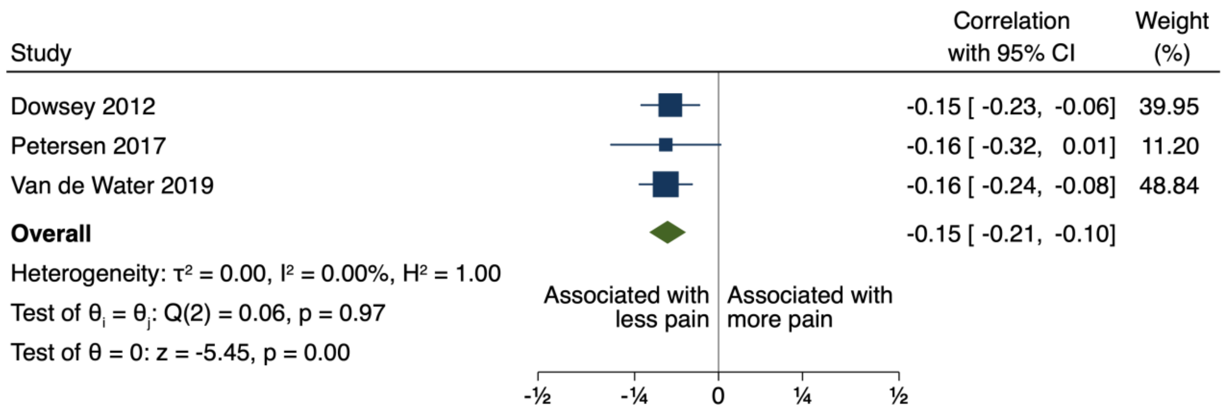

↑K-L Grade (random-effects REML model)

S69 Fig. ↑Warm Detection Threshold

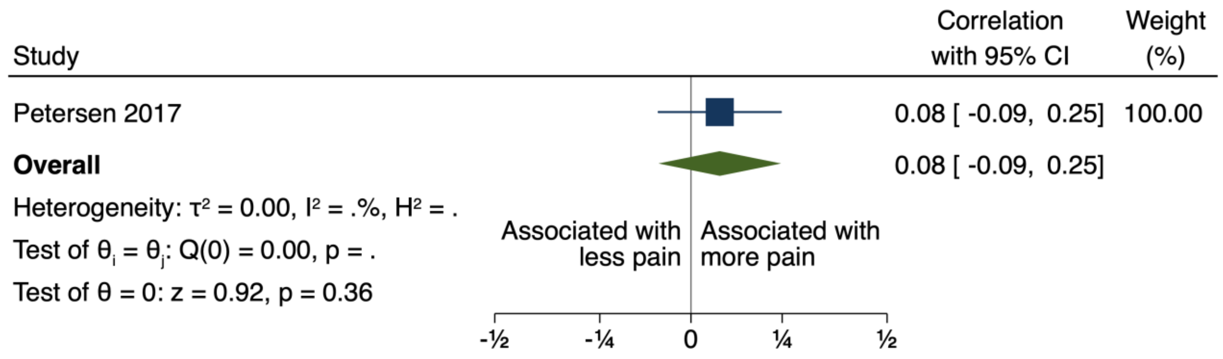

↑Warm Detection Threshold (random-effects REML model)

S70 Fig. ↑miRNA-146a-5p

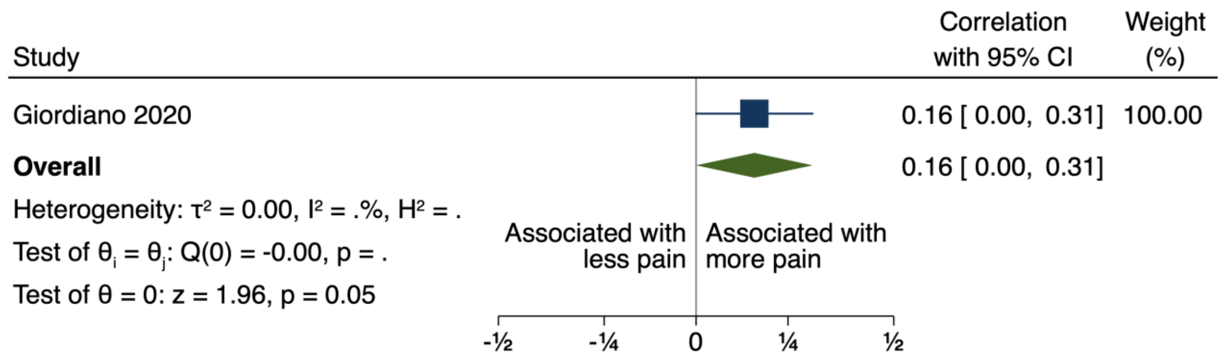

↑miRNA-146a-5p (random-effects REML model)

S71 Fig. ↑Kinesophobia

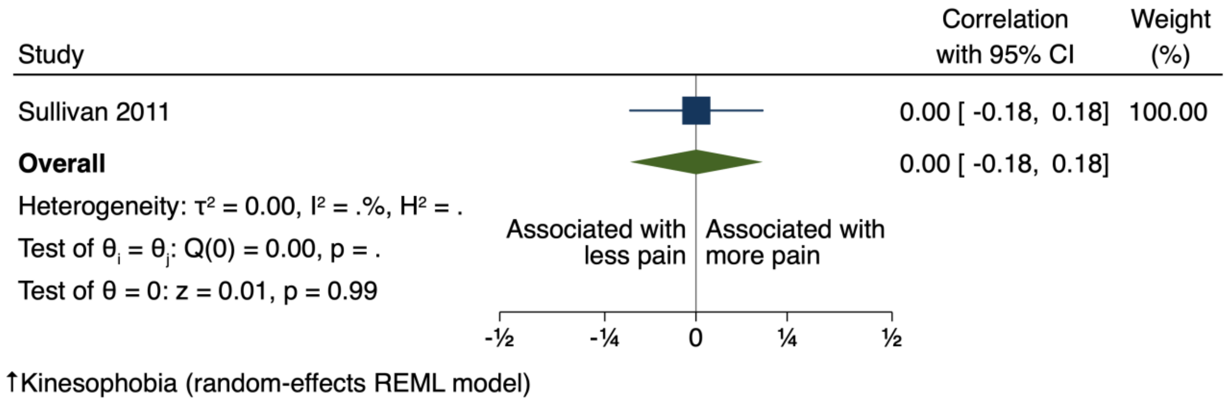

S72 Fig. Male Gender

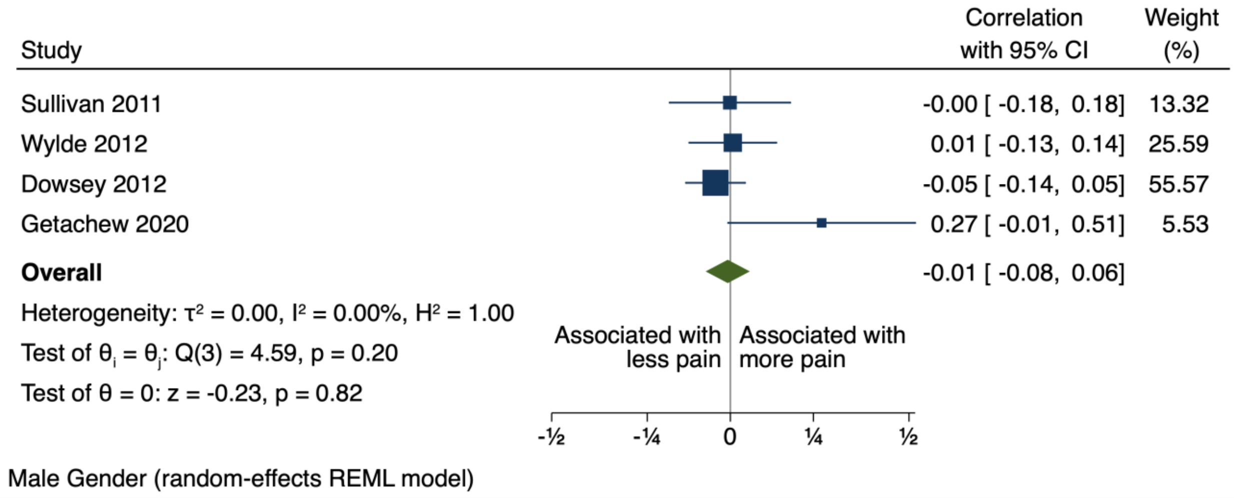

S73 Fig. ↑Age

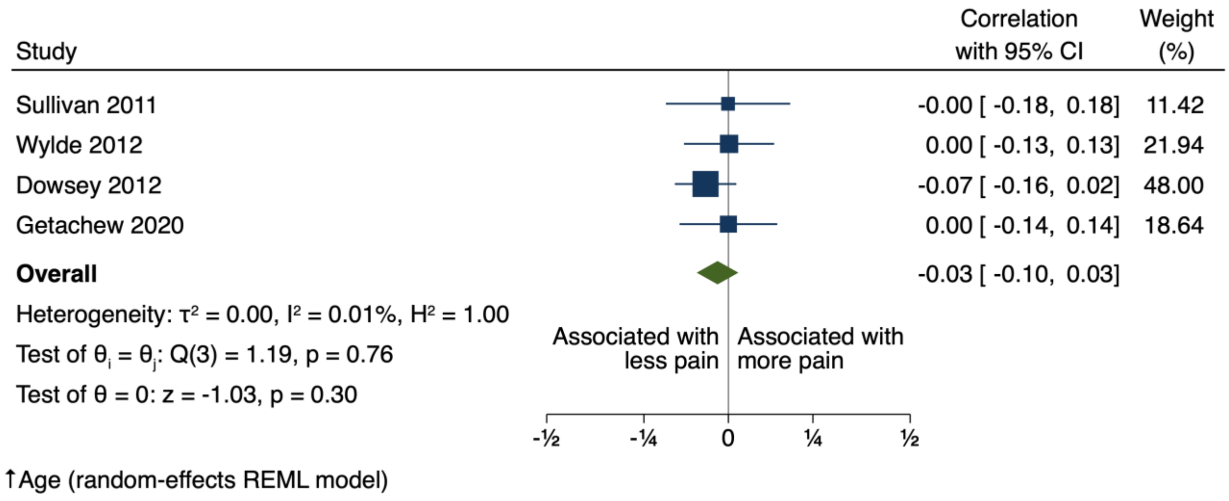

S74 Fig. ↑Pain Self-Efficacy

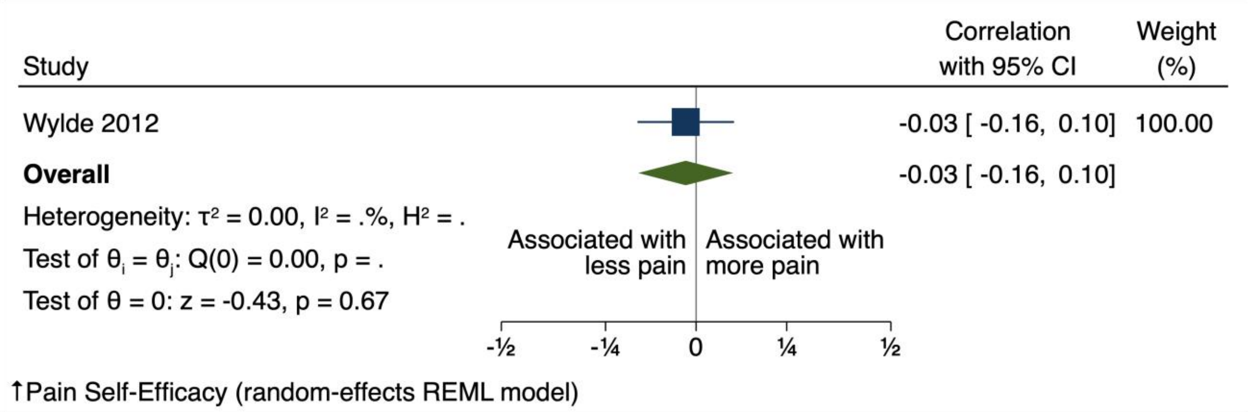

S75 Fig. Patella Resurfaced

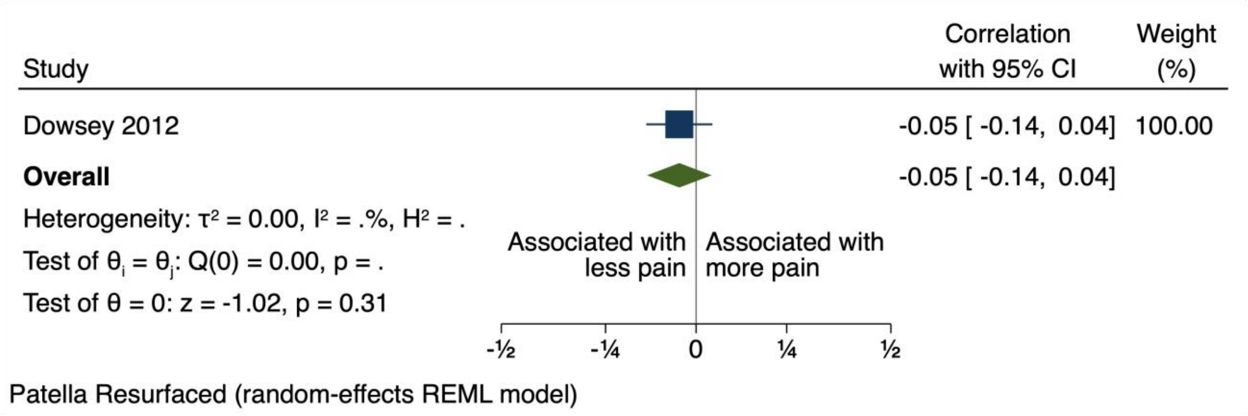

S76 Fig. ↑Preoperative Pain

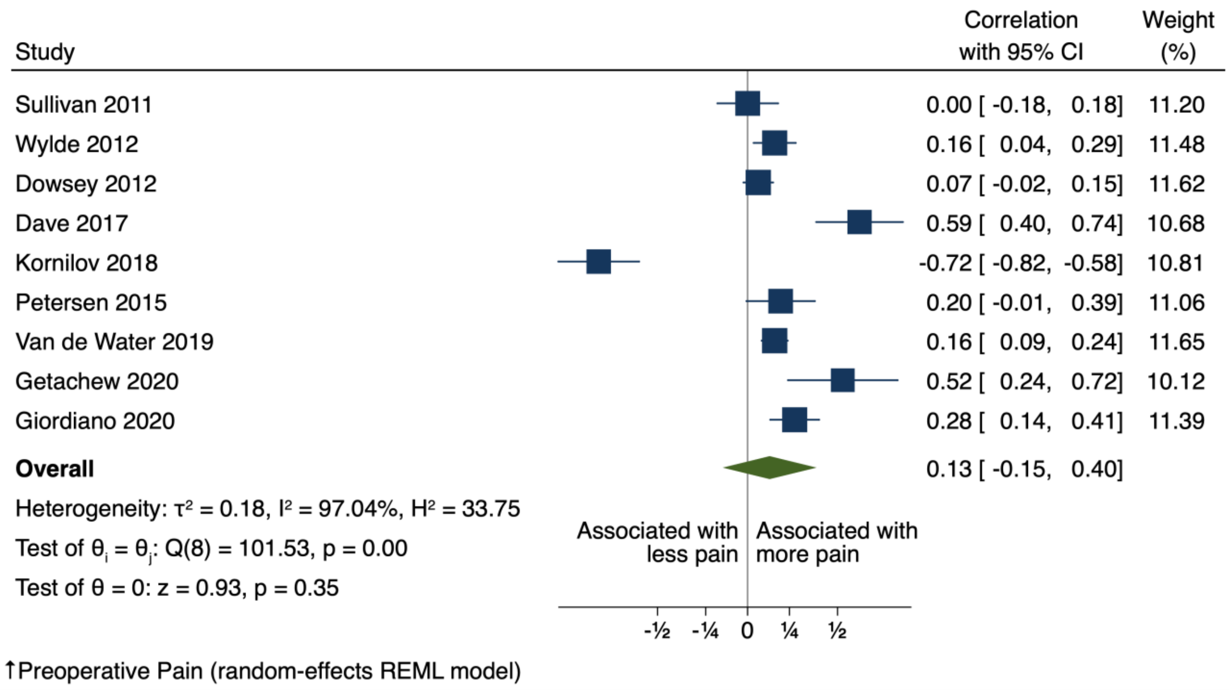

S77 Fig. ↑Surgery Duration

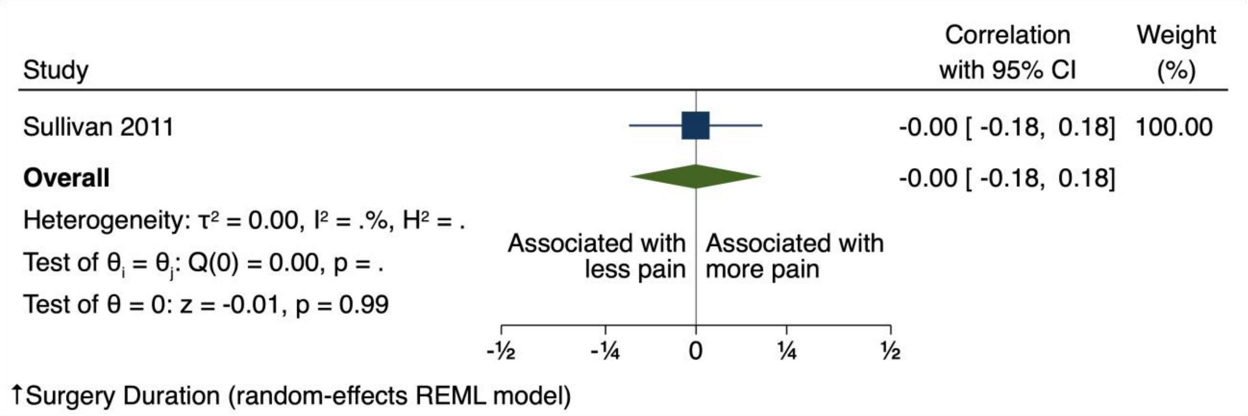

S78 Fig. ↑Symptomatic Joints

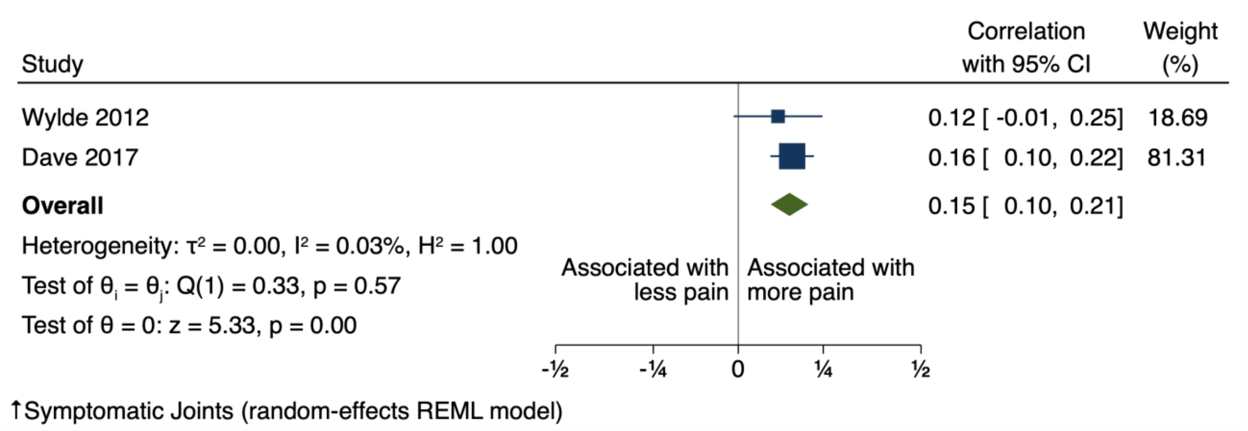

S79 Fig. ↑Synovial Membrane Thickness

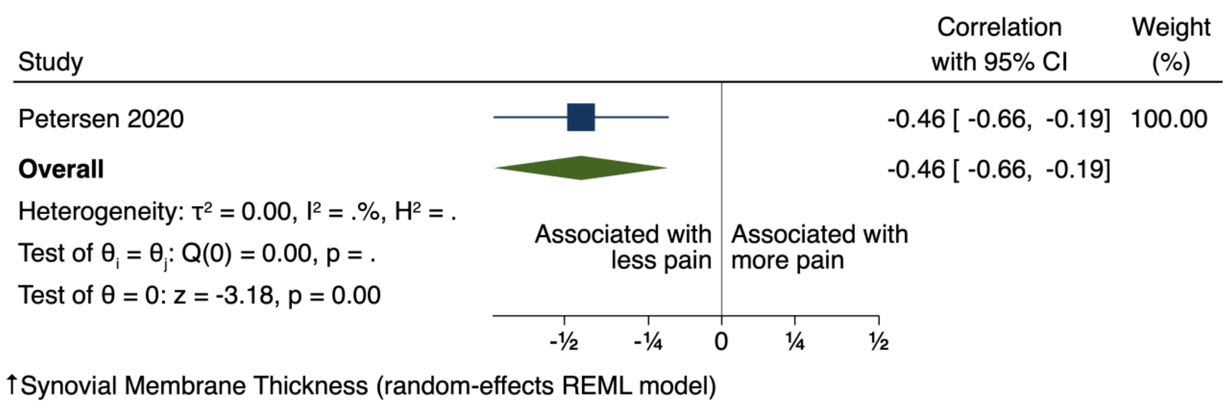

S80 Fig. ↑Synovitis Severity

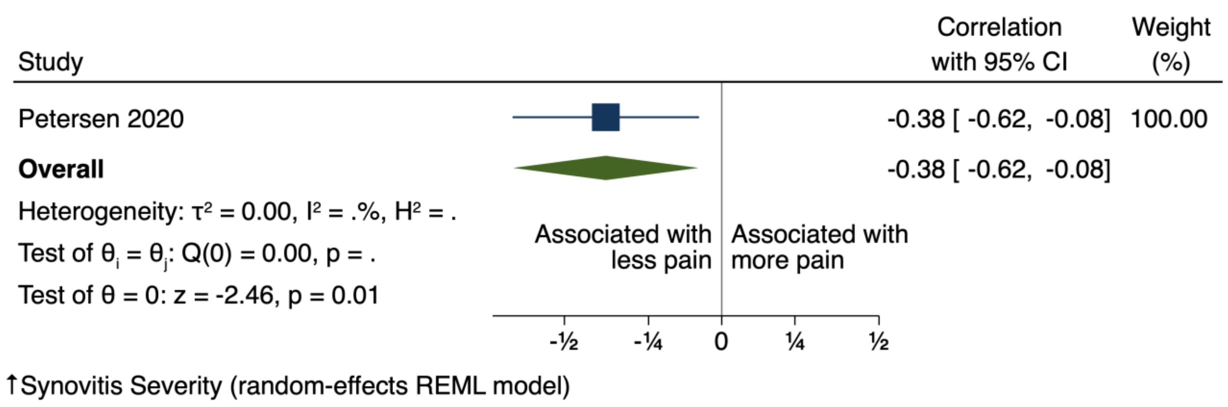

S81 Fig. ↑Temporal Summation

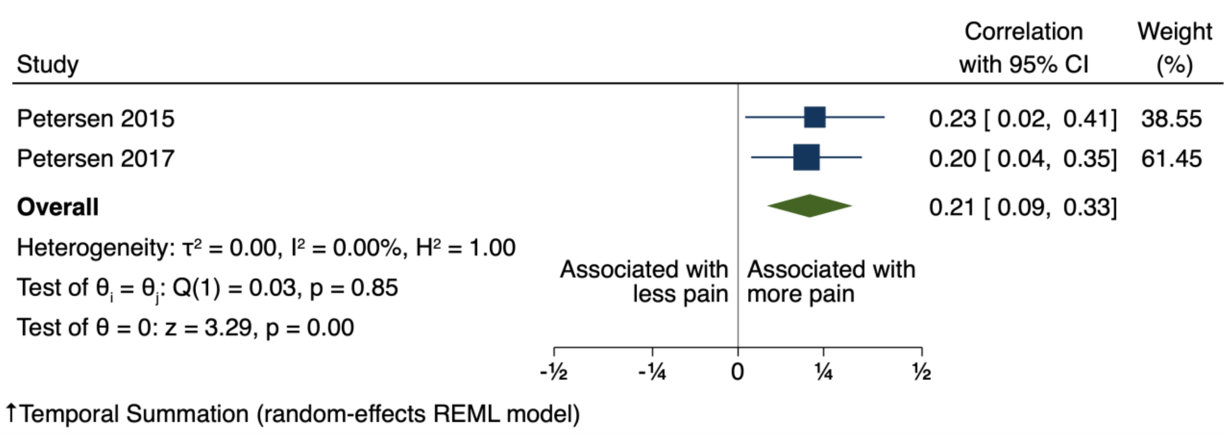

S82 Fig. ↑Synovial Perfusion (Volume)

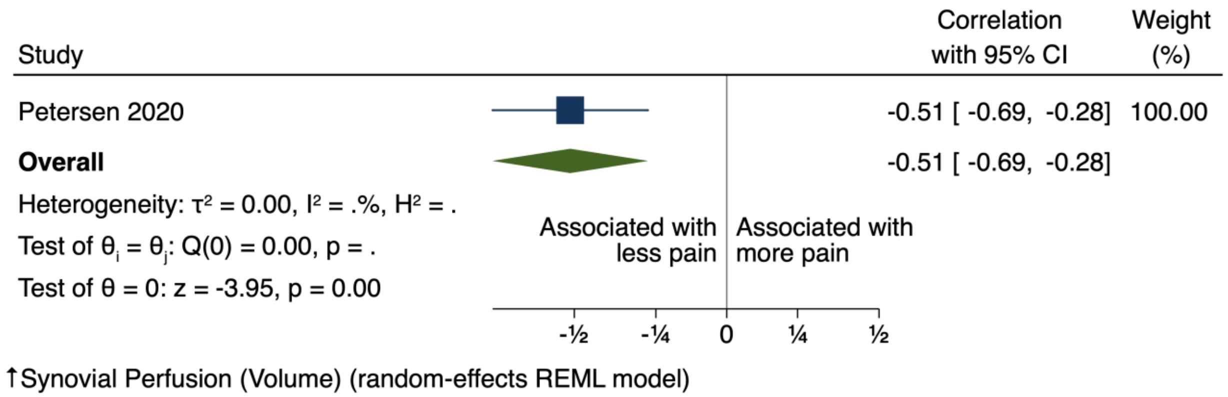

S83 Fig. Worse Sleep Quality

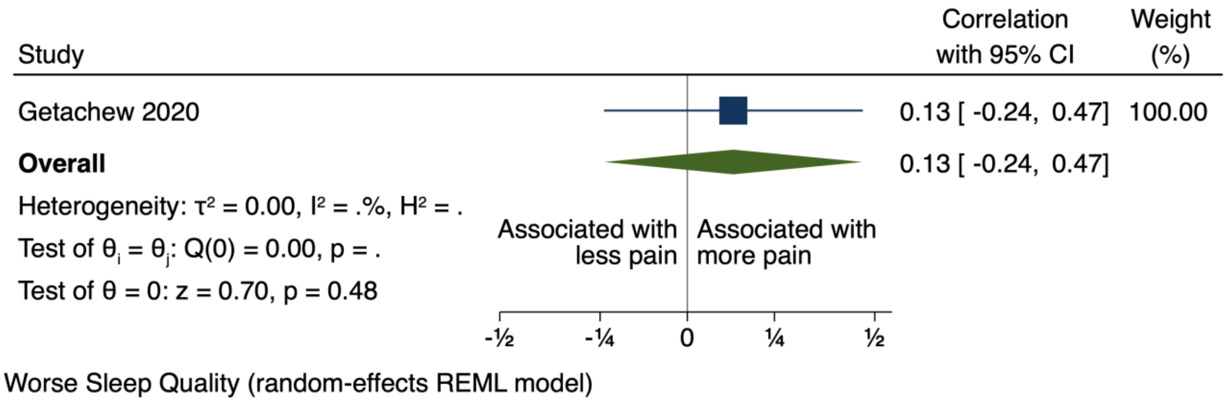

Supplement: S3 Appendix — (PDF) [file pone.0283446.s004.pdf]
