# Supplementary material for: Factors correlated with pain after total knee arthroplasty: A systematic review and meta-analysis
Source: PLoS One. 2023 Mar 24;18(3):e0283446. doi: 10.1371/journal.pone.0283446 (PMC10038299; doi:10.1371/journal.pone.0283446)
Supplement: S4 Appendix — (PDF) [file pone.0283446.s005.pdf]

S4 Appendix — Sensitivity analysis

The following table shows estimates of correlations for each of the sensitivity analyses. Estimates for the main analysis (i.e., no studies omitted) are also shown for comparison.

domain_str	concept	cc	cc_lb	cc_ub
All Studies	↑Mental Health	-.0852937	-.1735836	.0043621
All Studies	↑Outcome Expected	-.1423494	-.3641292	.094721
All Studies	↑Preoperative Function	-.0157737	-.2209335	.1907312
All Studies	↑ROCF Recall	.3421185	.0616148	.572538
All Studies	↑Catastrophizing	.3607808	.2353685	.4743794
All Studies	↑Comorbidity	.0322214	-.0608956	.1247877
All Studies	Cruciate Retaining	-.1398873	-.2508551	-.0252854
All Studies	↑Synovial Perfusion (Degree)	-.528002	-.7312685	-.2385985
All Studies	↑Fatigue	.4006917	.0573444	.6592825
All Studies	↑BMI	.0864361	-.0087192	.1800451
All Studies	↑Heat Pain Threshold	.1585547	-.0508364	.354598
All Studies	↑K-L Grade	-.1538206	-.2284379	-.0774014
All Studies	↑Warm Detection Threshold	.080546	-.1348203	.2886503
All Studies	↑miRNA-146a-5p	.1604646	-.0426043	.3508062
All Studies	↑Kinesophobia	.0012061	-.2286255	.2309155
All Studies	Male Gender	-.0079606	-.0987096	.082925
All Studies	↑Age	-.0328553	-.1176403	.0524106
All Studies	↑Pain Self-Efficacy	-.0292596	-.1968974	.1400458

All Studies	Patella Resurfaced	-.0472692	-.1640953	.07087
All Studies	↑Preoperative Pain	.1287325	.0628264	.1935257
All Studies	↑Surgery Duration	-.0012116	-.2309155	.2286255
All Studies	↑Symptomatic Joints	.152737	.0753108	.2283376
All Studies	↑Synovial Membrane Thickness	-.4550022	-.7016295	-.1110132
All Studies	↑Synovitis Severity	-.3840023	-.6689158	-.0007078
All Studies	↑Temporal Summation	.210876	.0510583	.3601678
All Studies	↑Synovial Perfusion (Volume)	-.511002	-.7237079	-.2096232
All Studies	Worse Sleep Quality	.1341878	-.3241869	.5415372
Study Participation	↑Mental Health	-.0755814	-.2167581	.0686603
Study Participation	↑Outcome Expected	-.1424058	-.456905	.2038635
Study Participation	↑Preoperative Function	-.0157778	-.3001231	.271159
Study Participation	↑Catastrophizing	.439657	.2173149	.6186406
Study Participation	↑Comorbidity	.0621851	-.0720388	.1942017
Study Participation	Cruciate Retaining	-.1398872	-.2664513	-.0085757
Study Participation	↑Fatigue	.4006918	.0517632	.6624355
Study Participation	↑BMI	.1015797	-.018966	.2192196

Study Participation	↑K-L Grade	-.1531539	-.2525015	-.0506056
Study Participation	Male Gender	-.0159879	-.1452506	.1138169
Study Participation	↑Age	-.0497386	-.1685098	.0704614
Study Participation	Patella Resurfaced	-.047269	-.1799814	.0871385
Study Participation	↑Preoperative Pain	.1064387	.0059582	.2047965
Study Participation	↑Symptomatic Joints	.1604733	.0548118	.2625855
Study Participation	Worse Sleep Quality	.1341879	-.3280942	.5446201
Study Attrition	↑Mental Health	-.1594349	-.2891942	-.0239217
Study Attrition	↑Preoperative Function	-.0536772	-.3432368	.2451973
Study Attrition	↑Catastrophizing	.4800008	.1900038	.6929598
Study Attrition	↑Comorbidity	.0376846	-.0747794	.1492019
Study Attrition	Cruciate Retaining	-.1398846	-.2615143	-.0138818
Study Attrition	↑Fatigue	.400694	.0536079	.6613961
Study Attrition	↑BMI	.0418484	-.0874288	.1697393
Study Attrition	↑Heat Pain Threshold	.1585574	-.057279	.3602341
Study Attrition	↑K-L Grade	-.153818	-.2436976	-.0613188
Study Attrition	↑Warm Detection Threshold	.0805487	-.1410001	.2944145
Study Attrition	Male Gender	-.0089947	-.1186882	.1009156
Study Attrition	↑Age	-.03693	-.1401105	.0670439
Study Attrition	↑Pain Self-Efficacy	-.0292568	-.2045158	.1478195

Study Attrition	Patella Resurfaced	-.0472664	-.1749382	.0819686
Study Attrition	↑Preoperative Pain	.1402615	.0495664	.2286622
Study Attrition	↑Symptomatic Joints	.1188633	-.055696	.2863716
Study Attrition	↑Temporal Summation	.2016515	-.0083138	.3945865
Study Attrition	Worse Sleep Quality	.1341905	-.3268017	.5436009
Prognostic Factor Measurement	↑Mental Health	-.0852779	-.1805175	.0115201
Prognostic Factor Measurement	↑Outcome Expected	-.142381	-.3648976	.0955985
Prognostic Factor Measurement	↑Preoperative Function	-.0157526	-.2283689	.1982654
Prognostic Factor Measurement	↑ROCF Recall	.3421057	.0616061	.5725434
Prognostic Factor Measurement	↑Catastrophizing	.3608019	.2312859	.4777171
Prognostic Factor Measurement	↑Comorbidity	.0322023	-.1221795	.185109
Prognostic Factor Measurement	Cruciate Retaining	-.1398864	-.252015	-.0240478
Prognostic Factor Measurement	↑Synovial Perfusion (Degree)	-.5280015	-.7314625	-.2382051
Prognostic Factor Measurement	↑Fatigue	.4006923	.0569549	.6595034
Prognostic Factor Measurement	↑BMI	.0864374	-.0102152	.1814925
Prognostic Factor Measurement	↑K-L Grade	-.1531527	-.2330902	-.0711565
Prognostic Factor Measurement	↑miRNA-146a-5p	.1604654	-.0433047	.3514214

Prognostic Factor Measurement	↑Kinesophobia	.0012068	-.2292068	.2314963
Prognostic Factor Measurement	Male Gender	-.0079595	-.1002597	.0844798
Prognostic Factor Measurement	↑Age	-.0328541	-.1192861	.0540753
Prognostic Factor Measurement	↑Pain Self-Efficacy	-.0292588	-.197707	.1408717
Prognostic Factor Measurement	Patella Resurfaced	-.0472682	-.1652719	.0720734
Prognostic Factor Measurement	↑Preoperative Pain	.1287339	.0607071	.1955727
Prognostic Factor Measurement	↑Surgery Duration	-.0012108	-.2314962	.2292069
Prognostic Factor Measurement	↑Symptomatic Joints	.1527383	.0735094	.230054
Prognostic Factor Measurement	↑Synovial Membrane Thickness	-.4550016	-.7018213	-.1106399
Prognostic Factor Measurement	↑Synovitis Severity	-.3840017	-.6691118	-.0003529
Prognostic Factor Measurement	↑Temporal Summation	.2255055	-.0300456	.4533881
Prognostic Factor Measurement	↑Synovial Perfusion (Volume)	-.5110015	-.7239023	-.2092327
Prognostic Factor Measurement	Worse Sleep Quality	.1341885	-.3244589	.541752
Outcome Measurement	↑Mental Health	-.0852795	-.181306	.012335
Outcome Measurement	↑Outcome Expected	-.142351	-.3632337	.0936981
Outcome Measurement	↑Preoperative Function	-.0157494	-.223382	.1932119

Outcome Measurement	↑Catastrophizing	.3607752	.2347048	.4749236
Outcome Measurement	↑Comorbidity	.0322414	-.1182191	.1812213
Outcome Measurement	Cruciate Retaining	-.1398893	-.3507725	.0845065
Outcome Measurement	↑Synovial Perfusion (Degree)	-.528012	-.7473536	-.2048623
Outcome Measurement	↑Fatigue	.4007021	.0572304	.6593471
Outcome Measurement	↑BMI	.0864482	-.0091589	.1804705
Outcome Measurement	↑Heat Pain Threshold	.1585667	-.0510352	.3547722
Outcome Measurement	↑K-L Grade	-.153809	-.2289502	-.0768641
Outcome Measurement	↑Warm Detection Threshold	.0805582	-.1350109	.2888283
Outcome Measurement	↑miRNA-146a-5p	.1604767	-.0428093	.3509863
Outcome Measurement	↑Kinesophobia	.0012185	-.2287956	.2310855
Outcome Measurement	Male Gender	-.0079486	-.0991654	.0833822
Outcome Measurement	↑Age	-.0328433	-.1181247	.0529005
Outcome Measurement	↑Pain Self-Efficacy	-.0292473	-.1971345	.1402876
Outcome Measurement	Patella Resurfaced	-.047257	-.1644405	.071223
Outcome Measurement	↑Preoperative Pain	.1287441	.0622001	.1941309
Outcome Measurement	↑Surgery Duration	-.0011992	-.2310855	.2287956

Outcome Measurement	↑Symptomatic Joints	.1527487	.0747801	.2288435
Outcome Measurement	↑Synovial Membrane Thickness	-.4549924	-.7016856	-.110904
Outcome Measurement	↑Synovitis Severity	-.3839917	-.6689731	-.000604
Outcome Measurement	↑Temporal Summation	.2108878	.0508014	.360392
Outcome Measurement	↑Synovial Perfusion (Volume)	-.5109928	-.7237648	-.209509
Outcome Measurement	Worse Sleep Quality	.1341999	-.3242665	.5416001
Study Confounding	↑Mental Health	-.0852324	-.196844	.0284424
Study Confounding	↑Outcome Expected	-.1423387	-.4085384	.1461688
Study Confounding	↑Preoperative Function	-.053658	-.3129129	.2129989
Study Confounding	↑Catastrophizing	.1873704	-.118748	.4609402
Study Confounding	↑Comorbidity	.0322468	-.1485123	.2108774
Study Confounding	Cruciate Retaining	-.1398377	-.25597	-.0198222
Study Confounding	↑Fatigue	.4007347	.0555935	.6602743
Study Confounding	↑BMI	.0864859	-.0152702	.1863771
Study Confounding	↑K-L Grade	-.1531053	-.2386079	-.0653411
Study Confounding	↑Kinesophobia	.0012573	-.2312269	.233514

Study Confounding	Male Gender	-.0079105	-.1054784	.0897141
Study Confounding	↑Age	-.0328054	-.1247995	.0596538
Study Confounding	↑Pain Self-Efficacy	-.0292086	-.2005044	.1437255
Study Confounding	Patella Resurfaced	-.0472186	-.1692902	.0761845
Study Confounding	↑Preoperative Pain	.1297417	.0493242	.2083987
Study Confounding	↑Surgery Duration	-.0011604	-.233514	.2312269
Study Confounding	↑Symptomatic Joints	.1189108	-.051452	.2824597
Study Confounding	Worse Sleep Quality	.1342382	-.3254116	.5425041
Statistical Analysis & Reporting	↑Mental Health	-.0911484	-.219432	.0402334
Statistical Analysis & Reporting	↑Outcome Expected	-.1421909	-.4300741	.1719405
Statistical Analysis & Reporting	↑Preoperative Function	-.0537848	-.3341957	.2353735
Statistical Analysis & Reporting	↑Catastrophizing	.3800155	.0377161	.6425011
Statistical Analysis & Reporting	↑Comorbidity	.0376198	-.1842784	.2558691
Statistical Analysis & Reporting	Cruciate Retaining	-.1398819	-.2607852	-.0146585
Statistical Analysis & Reporting	↑Fatigue	.4006959	.0538755	.6612477

Statistical Analysis & Reporting	↑BMI	.1015847	-.0122767	.2128454
Statistical Analysis & Reporting	↑K-L Grade	-.1531478	-.2451055	-.0584591
Statistical Analysis & Reporting	Male Gender	-.0089922	-.1177998	.1000287
Statistical Analysis & Reporting	↑Age	-.0369268	-.1391738	.0660993
Statistical Analysis & Reporting	↑Pain Self-Efficacy	-.0292544	-.203982	.1472794
Statistical Analysis & Reporting	Patella Resurfaced	-.0472637	-.1741945	.0812121
Statistical Analysis & Reporting	↑Preoperative Pain	.1610697	-2.22e-16	.2468102
Statistical Analysis & Reporting	↑Symptomatic Joints	.1527433	.0604436	.2424531
Statistical Analysis & Reporting	Worse Sleep Quality	.1341926	-.3266138	.5434558
