# Supplementary material for: Factors correlated with pain after total knee arthroplasty: A systematic review and meta-analysis
Source: PLoS One. 2023 Mar 24;18(3):e0283446. doi: 10.1371/journal.pone.0283446 (PMC10038299; doi:10.1371/journal.pone.0283446)
Supplement: S5 Appendix — (PDF) [file pone.0283446.s006.pdf]

S5 Appendix Definitions and labels of the factors

Factor	Definition
↑Age	Older age
↑Agreeableness	More agreeableness
↑Arthritis Helplessness	More worse arthritis helplessness
↑AT2R level	Higher AT2R level
↑BMI	Higher body mass index
↑Catastrophizing	More (Worse) catastrophizing
↑Comorbidity	More comorbidities
↑Contralateral Knee Pain	More (worse) contralateral knee pain
↑Cortisol	Higher cortisol
Cruciate Retaining	Cruciate Retaining surgery
↑Degree synovial perfusion	Higher degree of perfusion
↑Disability	More overall disability
↑Education	Higher educational attainment
↑Fatigue	More fatigue
↑Heart Rate Variability	Higher heart rate variability
↑Heat Pain Threshold	Higher heat pain threshold
Chinese ethnicity	Chinese (rather than Indian) ethnicity
↑Kinesophobia	More kinesophobia
↑K-L Grade	Higher worse Kellgren-Lawrence grade
↑Knee Extension	Greater better knee extension
↑Knee Flexion	Greater better knee flexion
↑Low Back Pain	More low back pain
Male gender	Male gender
↑Mental Health	Better improved mental health
↑miRNA-146a-5p	Higher values of miRNA-146a-5p
↑Opioid use	Higher opioid use
↑Outcome Expectation	Better outcome expected
↑Oxidative stress	More oxidative stress
↑Pain Expectation	Worse pain outcome expected
↑Pain Modulation	Better conditioned pain modulation
↑Pain Self-Efficacy	More better pain self-efficacy
↑Chronic pain sites	More chronic pain sites
Patella Resurfaced	Patella Resurfaced surgery
↑Preoperative Function	Better preoperative function
↑Preoperative Pain	More (worse) pain
↑ROCF Recall	Better recall on Rey-Osterrieth Complex Fig.ure test
↑Sleep dysfunction	More sleep dysfunction
↑Sleepiness daytime	More daytime sleepiness
↑Sleep quality	Worse sleep quality
↑Social Support	Better social support
Spinal Anesthesia	Spinal rather than general anesthesia
↑Walking aid use	Walking aid use

↑Surgery Duration	Longer surgery duration
↑ Suspected neuropathic pain	More suspected neuropathic pain
↑Sympathetic/Parasympathetic Activity	More sympathetic/parasympathetic activity
↑Symptomatic Joints	More symptomatic joints
↑Sleep quality	Worse sleep quality
↑Sleep quality	Worse sleep quality
↑Social Support	Better social support
Spinal Anesthesia	Spinal rather than general anesthesia
↑Walking aid use	Walking aid use
↑Surgery Duration	Longer surgery duration
↑ Suspected neuropathic pain	More suspected neuropathic pain
↑Sympathetic/Parasympathetic Activity	More sympathetic/parasympathetic activity
↑Symptomatic Joints	More symptomatic joints
↑Synovial membrane thickness	Thicker synovial membrane
↑Synovitis severity	More severe synovitis
↑Temporal Summation	More temporal summation
↑Trail Making Time	Longer worse trail making time
↑Volume of perfusion	Higher volume of perfusion
↑Warm Detection Threshold	Higher warm detection threshold
