# Supplementary material for: Factors correlated with pain after total knee arthroplasty: A systematic review and meta-analysis
Source: PLoS One. 2023 Mar 24;18(3):e0283446. doi: 10.1371/journal.pone.0283446 (PMC10038299; doi:10.1371/journal.pone.0283446)
Supplement: S6 Appendix — (PDF) [file pone.0283446.s007.pdf]

S6 Appendix Risk of Bias assessed by Quality in Prognostic Studies tool (QUIPS)

Study	Study participation	Study attrition	Prognostic factor measure	Outcome measure	Study confounding	Statistical analysis	Risk within a study
Attal et al.,[1] 2014	High	High	Moderate	High	High	High	High
Bruehl et al.,[2] 2023	Moderate	Low	Low	Low	Low	Low	Moderate
Bossman et al.,[3] 2017	High	High	Low	Low	High	High	High
Bugada et al.,[4] 2017	Moderate	Moderate	High	Moderate	High	High	High
Chen et al.,[5] 2021	Moderate	High	Moderate	Moderate	Moderate	Moderate	High
Cremeans-Smith et al.,[6] 2016	High	High	Low	Low	Moderate	Moderate	High
Dave et al.,[7] 2017	Moderate	High	Moderate	Moderate	High	Moderate	High
Dowsey et al.,[8] 2012	Moderate	Low	Moderate	Low	Moderate	Low	Moderate
Edwards et al.,[9] 2022	Moderate	High	High	Low	High	Moderate	High
Engel et al.,[10] 2004	High	High	Moderate	Low	High	High	High
Escobar et al.,[11] 2007	High	High	Low	Low	Moderate	Low	High
Fitzsimmons et al.,[12] 2018	Moderate	High	Low	Low	Low	Moderate	High
Getachew et al.,[13] 2021	Moderate	Moderate	Low	Low	Moderate	Moderate	Moderate
Giordiano et al.,[14] 2020	High	High	Moderate	Low	High	High	High
Hardy et al.,[15] 2022	Moderate	Moderate	Moderate	Low	High	High	High
Kornilov et al.,[16] 2018	Moderate	High	Low	Low	High	High	High
Lingard et al.,[17] 2007	Low	High	Low	Low	Low	Low	High
Lindner et al.,[18] 2018	High	Moderate	Moderate	Low	High	High	High
Luo et al,[19] 2019	Moderate	Low	Low	Moderate	High	High	High
Petersen et al.,[20] 2015	High	High	Moderate	Moderate	High	High	High
Petersen et al.,[21] 2017	High	Moderate	High	Moderate	High	High	High
Petersen et al.,[22] 2020	High	High	Moderate	Low	High	High	High
Perruccio et al.,[23] 2019	Low	Low	Low	Low	Moderate	Moderate	Moderate
Pua 2019 et al.,[24] 2019	Low	Low	Low	Low	Moderate	Low	Moderate
Sullivan et al.,[25] 2011	High	High	Low	Low	Moderate	High	High
Tilbury et al.,[26] 2018	Moderate	High	Low	Low	Moderate	Moderate	High
Wylde et al.,[27] 2012	High	Moderate	Low	Low	Moderate	Low	High
Van de Water et al.,[28] 2019	Low	Low	Low	Low	Low	Moderate	Moderate
Yang et al,[29] 2019	Moderate	Low	Low	Moderate	Moderate	Moderate	Moderate

Low risk of bias	Moderate risk of bias	High risk of bias
