# Supplementary material for: Factors correlated with pain after total knee arthroplasty: A systematic review and meta-analysis
Source: PLoS One. 2023 Mar 24;18(3):e0283446. doi: 10.1371/journal.pone.0283446 (PMC10038299; doi:10.1371/journal.pone.0283446)
Supplement: S7 Appendix — (PDF) [file pone.0283446.s008.pdf]

S7 Appendix Grading of Recommendation Assessment, Development and Evaluation (GRADE)

Grading of Recommendation Assessment, Development and Evaluation (GRADE) – 12 mo follow-up

Authors: Unni Olsen, Maren Falch Lindberg, Eva Denison

№ of studies	Certainty assessment at 12 mo follow-up						Certainty	Explanations
	Study design	Risk of bias	Inconsistency	Indirectness	Imprecision	Other considerations		
**Catastrophizing**								
2,[1,5,27]	observational studies	serious[a]	not serious	not serious	serious[b]	none	⊕⊕⊕◯ Moderate	[a.] High risk of bias on several domains
**Kellgren Lawrence grade**								
3,[3,8,11]	observational studies	serious[c]	not serious	not serious	serious[d]	none	⊕⊕◯◯ Low	[c.] High risk of bias in one study<br>[d.] Imprecise effect estimate
**Preoperative pain**								
9,[1-9]	observational studies	serious[e]	serious[f]	serious[g]	serious[h]	none	⊕◯◯◯ Very low	[e.] High risk of bias at several domains across studies.<br>[f.] Statistical heterogeneity and inconsistency in direction of associations.<br>[h.] Imprecise estimate
**Temporal summation**								

№ of studies	Certainty assessment at 12 mo follow-up						Certainty	Explanations
	Study design	Risk of bias	Inconsistency	Indirectness	Imprecision	Other considerations		
2,[7,11]	observational studies	very serious[i]	not serious	not serious	serious[j]	none	⨁◯◯◯ Very low	[i.] High risk of bias on several domains<br>[j.] Estimate based on a total sample n=361
**Symptomatic joint count**								
2,[2,5]	observational studies	serious[k]	not serious	not serious	not serious	none	⨁⨁⨁◯ Moderate	[k.] High risk of bias on two domains
**Mental health**								
6,[1-4,10,12]	observational studies	serious[l]	serious[m]	not serious	not serious	none	⨁⨁◯◯ Low	[l.] High risk of bias on several domais<br>[m.] Statistical heterogeneity, and some differences in how pain is measured. Small inconsistency in direction of effect.
**Preoperative function**								
2,[3,6]	observational studies	serious[n]	serious[o]	not serious	serious[p]	none	⨁◯◯◯ Very low	[n.] High risk of bias at several domains across studies.<br>[o.] Heterogeneity in measurement of risk factor<br>[p.] Imprecise effect estimate
**Gender**								

№ of studies	Certainty assessment at 12 mo follow-up						Certainty	Explanations
	Study design	Risk of bias	Inconsistency	Indirectness	Imprecision	Other considerations		
4,[1-4]	observational studies	serious[q]	not serious	not serious	serious[r]	none	⊕⊕◯◯ Low	[q.] High risk of bias on several domains<br>[r.] Imprecise effect estimate
**Age**								
4,[1-4]	observational studies	serious[s]	not serious	not serious	not serious	none	⊕⊕⊕◯ Moderate	[s.] High risk of bias on several domains
**Comorbidity**								
3,[1-3]	observational studies	serious[t]	not serious	not serious	not serious	none	⊕⊕⊕◯ Moderate	[t.] High risk of bias on several domains
**Body Mass Index**								
4, [1,3,10]	observational studies	serious[u]	serious[v]	not serious	not serious	none	⊕⊕◯◯ Low	[u.] High risk of bias on several domains<br>[v.] Some statistical heterogeneity
**Cruciate retaining**								
1,[3]	observational studies	not serious	not serious	not serious	serious[w]	none	⊕⊕⊕◯ Moderate	[w.] Estimate based on one study (n=473)
**Pain self-efficacy**								
1,[2]	observational studies	not serious	not serious	not serious	very serious[y]	none	⊕⊕◯◯ Low	[y.] Estimate based on one study (n=220)
**Outcome expected**								

№ of studies	Certainty assessment at 12 mo follow-up						Certainty	Explanations
	Study design	Risk of bias	Inconsistency	Indirectness	Imprecision	Other considerations		
1,[10]	observational studies	not serious	not serious	not serious	very serious[z]	none	⊕⊕◯◯ Low	[z.] Estimate based on one study (n=146). imprecise effect estimate.
**Patella resurfaced**								
1,[3]	observational studies	not serious	not serious	not serious	serious[aa]	none	⊕⊕⊕◯ Moderate	[aa.] Estimate based on one study (n=473)
**Surgery duration**								
1,[1]	observational studies	very serious[ab]	not serious	not serious	very serious[ac]	none	⊕◯◯◯ Very low	[ab.] Several domains assessed with high risk of bias [ac.] Estimate based on one study (n=120)
**Kinesophobia**								
1,[1]	observational studies	very serious[ad]	not serious	not serious	very serious[ae]	none	⊕◯◯◯ Very low	[ad.] High risk of bias on several domains [ae.] Estimate based on one study (n=120)
**Warm detection treshold**								
1,[11]	observational studies	very serious[af]	not serious	not serious	very serious[ag]	none	⊕◯◯◯ Very low	[af.] High risk of bias on four domains. [ag.] Estimate based on one study (n=130)
**Heat detection**								

№ of studies	Certainty assessment at 12 mo follow-up						Certainty	Explanations
	Study design	Risk of bias	Inconsistency	Indirectness	Imprecision	Other considerations		
1,[11]	observational studies	very serious[ah]	not serious	not serious	very serious[ai]	none	⊕◯◯◯ Very low	[ah.] High risk of bias on four domains. [ai.] Estimate based on one study (n=130)
**ROCF recall**								
1,[13]	observational studies	very serious[aj]	not serious	not serious	very serious[ak]	none	⊕◯◯◯ Very low	[aj.] High risk of bias on five domains [ak.] Estimate based on one study (n=69).
**Fatigue**								
1,[4]	observational studies	not serious	not serious	not serious	very serious[al]	none	⊕⊕◯◯ Low	[al.] Estimate based on one study (n=185)
**Sleep quality**								
1,[4]	observational studies	not serious	not serious	not serious	extremely serious[am]	none	⊕◯◯◯ Very low	[am.] Estimate based on one small study (n=185). Imprecise effect estimate
**miRNA-146a-5p**								
1,[9]	observational studies	very seriousn[an]	not serious	not serious	very serious[ao]	none	⊕◯◯◯ Very low	[an.] High risk of bias on four domains [ao.] Estimate based on one study with very low sample size (n=136)
**Synovial membran**								

№ of studies	Certainty assessment at 12 mo follow-up						Certainty	Explanations
	Study design	Risk of bias	Inconsistency	Indirectness	Imprecision	Other considerations		
1,[14]	observational studies	very serious[ap]	not serious	not serious	very serious[aq]	none	⊕◯◯◯ Very low	[ap.] High risk of bias on four domains [aq.] Estimate based on one study (n=26)
**Degree perfusion**								
1,[14]	observational studies	very serious[ar]	not serious	not serious	very serious[as]	none	⊕◯◯◯ Very low	[ar.] High risk of bias on four domains [as.] Estimate based on one study with very low sample size (n=26)
**Volume perfusion**								
1,[14]	observational studies	very serious[at]	not serious	not serious	very serious[au]	none	⊕◯◯◯ Very low	[at.] High risk of bias on four domains [au.] Estimate based on one study with very low sample size (n=26)
**Synovitis severity**								
1,[14]	observational studies	very serious[av]	not serious	not serious	very serious[aw]	none	⊕◯◯◯ Very low	[av.] High risk of bias on four domains [aw.] Estimate based on one study with very low sample size (n=26)

Grading of Recommendation Assessment, Development and Evaluation (GRADE) – 6 mo follow-up

Authors: Unni Olsen, Maren Falch Lindberg, Eva Denison

№ of studies	Certainty assessment 6 mo follow-up						Certainty	Explanations
	Study design	Risk of bias	Inconsistency	Indirectness	Imprecision	Other considerations		
**Mental health**								
6, [15,16,19-21,29]	observational studies	serious[a]	not serious	not serious	not serious	none	⊕⊕⊕◯ Moderate	[a.] High risk of bias across domains
**Preoperative pain**								
5, [15-17,20,29]	observational studies	serious[c]	not serious	not serious	serious[d]	none	⊕⊕◯◯ Low	[c.] High risk of bias at several domains across studies<br>[d.] Imprecise effect estimates.
**Social support**								
2, [15,16]	observational studies	not serious	not serious	not serious	not serious	none	⊕⊕⊕⊕ High	Not downgraded
**Age**								
5, [15-19]	observational studies	serious[e]	not serious	not serious	serious[f]	none	⊕⊕◯◯ Low	[e.] High risk of bias on several domains<br>[f.] Imprecise effect estimate
**Gender**								

№ of studies	Certainty assessment 6 mo follow-up						Certainty	Explanations
	Study design	Risk of bias	Inconsistency	Indirectness	Imprecision	Other considerations		
3, [15-17]	observational studies	serious[g]	serious[h]	not serious	serious[i]	none	⊕◯◯◯ Very low	[g.] High risk of bias on several domains [h.] Statistical heterogeneity [i.] Imprecise effect estimates
**Body Mass index**								
2, [15,17]	observational studies	not serious	not serious	not serious	very serious[j]	none	⊕⊕◯◯ Low	[j.] Imprecise effect estimates, one study with very low sample size and a study with very large sample size. The study with low sample size are given very low weight
**Comorbidity**								
3,[15,16, 20]	observational studies	serious[k]	not serious	not serious	serious[l]	none	⊕⊕◯◯ Low	[k.] High risk of bias on several domains [l.] Imprecise effect estimates
**Pain catastrophizing**								
4 [17,20,21,29]	observational studies	serious[m]	serious[n]	not serious	extremely serious[o]	none	⊕◯◯◯ Very low	[m.] High risk of bias on several domains n. Statistical heterogeneity [o.] Imprecise effect estimates

№ of studies	Certainty assessment 6 mo follow-up						Certainty	Explanations
	Study design	Risk of bias	Inconsistency	Indirectness	Imprecision	Other considerations		
**Pain modulation**								
1,[17]	observational studies	very serious[p]	not serious	not serious	very serious[q]	none	⊕◯◯◯ Very low	[p.] High risk of bias across domains<br>[q]. Imprecise effect estimates and low sample size (n=47)
**Pain self-efficacy**								
1, [22]	observational studies	serious[r]	not serious	not serious	very serious[s]	none	⊕◯◯◯ Very low	[r.] High risk of bias across domains<br>[s.] Imprecise effect estimates and low sample size (n=54)
**Temporal summation**								
2, [17, 19]	observational studies	very serious[t]	not serious	not serious	very serious[u]	none	⊕◯◯◯ Very low	[t.] High risk of bias across domains<br>u. Imprecise effect estimates and low total sample size (n=267).
**Low back pain**								
1, [16]	observational studies	serious[v]	not serious	not serious	serious[w]	none	⊕⊕◯◯ Low	[v.] High risk of bias across domains<br>w. One study (n=640)
**Sympathetic/parasympathetic activity**								

№ of studies	Certainty assessment 6 mo follow-up						Certainty	Explanations
	Study design	Risk of bias	Inconsistency	Indirectness	Imprecision	Other considerations		
1, [17]	observational studies	very serious[x]	not serious	not serious	very serious[y]	none	⊕◯◯◯ Very low	[x.] High risk of bias across domains<br>y. Imprecise effect estimates and low sample size (n=47)
**Heart rate variability**								
1, [17]	observational studies	very serious[z]	not serious	not serious	extremely serious[aa]	none	⊕◯◯◯ Very low	[z.] High risk of bias across domains<br>[aa.] Imprecise effect estimates and low sample size (n=47)
**Contralateral knee pain**								
1,[15]	observational studies	not serious	not serious	not serious	serious[ab]	none	⊕⊕⊕◯ Moderate	[ab.]One study, large sample (n=4026)
**Education**								
1,[15]	observational studies	not serious	not serious	not serious	serious[ac]	none	⊕⊕⊕◯ Moderate	[ac.] One study, large sample (n=4026)
**Chinese ethnicity**								
1,[15]	observational studies	not serious	not serious	not serious	serious[ad]	none	⊕⊕⊕◯ Moderate	[ad.] One study, large sample (n=4026)
**Knee extension**								
1,[15]	observational studies	not serious	not serious	not serious	serious[ae]	none	⊕⊕⊕◯ Moderate	[ae.] One study, large sample (n=4026)
**Knee flexion**								

№ of studies	Certainty assessment 6 mo follow-up						Certainty	Explanations
	Study design	Risk of bias	Inconsistency	Indirectness	Imprecision	Other considerations		
1,[15]	observational studies	not serious	not serious	not serious	serious[af]	none	⊕⊕⊕◯ Moderate	[af.] One study, large sample (n=4026)
**Arthritis helplessness**								
1, [22]	observational studies	serious[ag]	not serious	not serious	very serious[ag]	none	⊕◯◯◯ Very low	[ag.] One study low sample size (n=54)
**Preoperative function**								
1, [15]	observational studies	not serious	not serious	not serious	serious[ah]	none	⊕⊕⊕◯ Moderate	[ah.] One study, sample size was large (n=4026)
**Trail making time**								
1, [13]	observational studies	very serious[ai]	not serious	not serious	very serious[aj]	none	⊕◯◯◯ Very low	[ai.] High risk of bias on four domains [aj.] Imprecise effect estimate and low sample size (n=74)
**Suspected neuropathic pain**								
1, [20]	observational studies	serious[ak]	not serious	not serious	extremely serious[al]	none	⊕◯◯◯ Very low	[ak.] High risk of bias across domains [al] Imprecise effect estimates, and low sample size (74).
**Walking aid use**								

№ of studies	Certainty assessment 6 mo follow-up						Certainty	Explanations
	Study design	Risk of bias	Inconsistency	Indirectness	Imprecision	Other considerations		
1, [21]	observational studies	not serious	not serious	not serious	extremely serious[am]	none	⊕◯◯◯ Very low	[am.] One study, small sample size (n=107)
**Angiotensin II Type 2 receptor**								
1,[19]	observational studies	serious	not serious	not serious	very serious[an]	none	⊕◯◯◯ Very low	[an.] One study (n=220)
**Overall disability**								
1,[19]	observational studies	serious[ao]	not serious	not serious	very serious[ap]	none	⊕◯◯◯ Very low	[ao.] High risk of bias on one domain [ap] Imprecise effect estimate
**Pain expectation**								
1,[19]	observational studies	serious	not serious	not serious	very serious[aq]	none	⊕◯◯◯ Very low	[aq.] One study (n=220)
**Symptomatic joints**								
1,[19,29]	observational studies	serious[ar]	not serious	not serious	serious[as]	none	⊕⊕◯◯ Low	[ar.] High risk of bias on one or more domains. [as.] One study (n=175)

№ of studies	Certainty assessment 6 mo follow-up						Certainty	Explanations
**Ischemia**								
1, [28]	observational study	not serious	not serious	not serious	extremely serious [au]	none	⊕◯◯◯ Very low	[au.] One study (n=91)
**Oxydative stress**								
1, [28]	observational study	not serious	not serious	not serious	extremely[au] serious	none	⊕◯◯◯ Very low	[au.] One study (n=91)
**Sleep efficacy**								
1, [29]	observational studies	very serious [ba]	not serious	not serious	very serious	none	⊕◯◯◯ very low	[ba.]High risk of bias across domains. [bb,] One study (n=175)
**Chronic pain sites**								
1, [29]	observational studies	very serious	not serious	not serious	very serious	none	⊕◯◯◯ very low	[ba.]High risk of bias across domains. [bb,] One study (n=175)
**Agreeableness**								
1, [29]	observational studies	very serious	not serious	not serious	very serious	none	⊕◯◯◯ very low	[ba.]High risk of bias across domains. [bb,] One study (n=175)

№ of studies	Certainty assessment 6 mo follow-up						Certainty	Explanations
**Opioid use**								
1,[29]	observational study	very serious	not serious	not serious	very serious	none	⊕◯◯◯ Very low	[ba.]High risk of bias across domains. [bb,] One study (n=175)

Grading of Recommendation Assessment, Development and Evaluation (GRADE) – 3 mo follow-up

Authors: Unni Olsen, Maren Falch Lindberg, Eva Denison

№ of studies	Certainty assessment 3 mo follow-up						Certainty	Explanation
	Study design	Risk of bias	Inconsistency	Indirectness	Imprecision	Other considerations		
**Preoperative pain**								
3, [23-25]	observational studies	serious[a]	not serious	not serious	serious[b]	none	⊕⊕◯◯ Low	[a.] High risk of bias for several domains across studies [b.] Imprecise effect estimate
**Mental health**								
2, [12,23]	observational studies	serious[c]	not serious	not serious	not serious	none	⊕⊕⊕◯ Moderate	[c.] High risk of bias for several domains across studies
**Age**								
1, [23]	observational studies	not serious	not serious	not serious	serious[d]	none	⊕⊕⊕◯ Moderate	[d.] One study (n=477)
**Gender**								
1, [23]	observational studies	not serious	not serious	not serious	serious[e]	none	⊕⊕⊕◯ Moderate	[e.] One study (n=477)
**Comorbidity**								
1, [23]	observational studies	not serious	not serious	not serious	serious[f]	none	⊕⊕⊕◯ Moderate	[f.] One study (n=477)
**Body Mass Index**								

№ of studies	Certainty assessment 3 mo follow-up						Certainty	Explanation
	Study design	Risk of bias	Inconsistency	Indirectness	Imprecision	Other considerations		
1, [23]	observational studies	not serious	not serious	not serious	serious[g]	none	⊕⊕⊕◯ Moderate	[g.] One study (n=477)
**Low back pain**								
1, [23]	observational studies	not serious	not serious	not serious	serious[h]	none	⊕⊕⊕◯ Moderate	[h.] One study (n=477)
**Education**								
1, [24]	observational studies	serious[i]	not serious	not serious	serious[j]	none	⊕⊕◯◯ Low	[i.] High risk of bias. [j.] Imprecise effect estimate. One study (n=101)
**Cortisol**								
1, [24]	observational studies	serious[k]	not serious	not serious	serious[l]	none	⊕⊕◯◯ Low	[k.] High risk of bias for several domains. [l.] Imprecise effect estimate and one study (n=101)
**Symptomatic joints**								
1, [23]	observational studies	not serious	not serious	not serious	serious[m]	none	⊕⊕⊕◯ Moderate	[m] One study (n=477)
**Spinal aneaesthesia**								

№ of studies	Certainty assessment 3 mo follow-up						Certainty	Explanation
	Study design	Risk of bias	Inconsistency	Indirectness	Imprecision	Other considerations		
1, [24]	observational studies	serious[n]	not serious	not serious	extremely serious[o]	none	⊕◯◯◯ Very low	[n.] High risk of bias for several domains [o.] Imprecise effect estimate, one study (n=101)
**Sleep dysfunction**								
1, [26]	observational studies	serious[p]	not serious	not serious	serious[q]	none	⊕⊕◯◯ Low	[p.] High risk of bias on two domains [q.] One study (n=471)
**Daytime sleepiness**								
1, [26]	observational studies	serious[r]	not serious	not serious	serious[s]	none	⊕⊕◯◯ Low	[r.] High risk of bias on two domains [s.] One study. (n=471)
**Sleep quality**								
1, [26]	observational studies	serious[t]	not serious	not serious	serious[u]	none	⊕⊕◯◯ Low	[t.] High risk of bias on two domains [u.] One study. (n=471)

References for the GRADE assessment

1. Sullivan M, Tanzer M, Reardon G, Amirault D, Dunbar M, Stanish W. The role of presurgical expectancies in predicting pain and function one year following total knee arthroplasty. *Pain*. Oct 2011;152(10):2287-2293. doi:10.1016/j.pain.2011.06.014
2. Wylde V, Dixon S, Blom AW. The role of preoperative self-efficacy in predicting outcome after total knee replacement. *Musculoskeletal Care*. 2012;10(2):110-8. doi:10.1002/msc.1008
3. Dowsey MM, Nikpour M, Dieppe P, Choong PF. Associations between pre-operative radiographic changes and outcomes after total knee joint replacement for osteoarthritis. *Osteoarthritis Cartilage*. Oct 2012;20(10):1095-102. doi:10.1016/j.joca.2012.05.015
4. Getachew M, Lerdal A, Smastuen MC, et al. High levels of preoperative pain and fatigue are red flags for moderate-severe pain 12 months after total knee arthroplasty-A longitudinal cohort study. *Musculoskeletal Care*. Jun 2021;19(2):186-192. doi:10.1002/msc.1522
5. Dave AJ, Selzer F, Losina E, et al. The association of pre-operative body pain diagram scores with pain outcomes following total knee arthroplasty. *Osteoarthritis Cartilage*. May 2017;25(5):667-675. doi:10.1016/j.joca.2016.12.013
6. Kornilov N, Lindberg MF, Gay C, et al. Higher physical activity and lower pain levels before surgery predict non-improvement of knee pain 1 year after TKA. *Knee Surg Sports Traumatol Arthrosc*. Jun 2018;26(6):1698-1708. doi:10.1007/s00167-017-4713-5
7. Petersen KK, Arendt-Nielsen L, Simonsen O, Wilder-Smith O, Laursen MB. Presurgical assessment of temporal summation of pain predicts the development of chronic postoperative pain 12 months after total knee replacement. *Pain*. Jan 2015;156(1):55-61. doi:10.1016/j.pain.0000000000000022
8. van de Water R, Leichtenberg C, Nelissen R, et al. Preoperative Radiographic Osteoarthritis Severity Modifies the Effect of Preoperative Pain on Pain/Function After Total Knee Arthroplasty: Results at 1 and 2 Years Postoperatively. *J Bone Joint Surgery Am*. 2019;101(10):879.
9. Giordano R, Petersen KK, Andersen HH, et al. Preoperative serum circulating microRNAs as potential biomarkers for chronic postoperative pain after total knee replacement. *Mol Pain*. Jan-Dec 2020;16:1744806920962925. doi:10.1177/1744806920962925
10. Tilbury C, Haanstra TM, Verdegaal SHM, et al. Patients' pre-operative general and specific outcome expectations predict postoperative pain and function after total knee and total hip arthroplasties. *Scand J Pain*. 2018;18(3):457-466. doi:10.1515/sjpain-2018-0022
11. Petersen KK, Simonsen O, Laursen MB, Arendt-Nielsen L. The Role of Preoperative Radiological Severity, Sensory Testing, and Temporal Summation on Chronic Postoperative Pain following Total Knee Arthroplasty. *Clinical Journal of Pain*. 2017-1-1 2017;34(3):193-197.
12. Lingard EA, Riddle DL. Impact of psychological distress on pain and function following knee arthroplasty. *J Bone Joint Surg Am*. Jun 2007;89(6):1161-9. doi:10.2106/JBJS.F.00914
13. Attal N, Masselin-Dubois A, Martinez V, et al. Does cognitive functioning predict chronic pain? Results from a prospective surgical cohort. *Brain*. Mar 2014;137(Pt 3):904-17. doi:10.1093/brain/awt354
14. Petersen KK, Arendt-Nielsen L, Vela J, et al. Less Severe Preoperative Synovitis is Associated With Higher Self-reported Pain Intensity 12 Months After Total Knee Arthroplasty-An Exploratory Prospective Observational Study. *Clin J Pain*. Jan 2020;36(1):34-40. doi:10.1097/AJP.0000000000000768
15. Pua YH, Poon CL, Seah FJ, et al. Predicting individual knee range of motion, knee pain, and walking limitation outcomes following total knee arthroplasty. *Acta Orthop*. Apr 2019;90(2):179-186. doi:10.1080/17453674.2018.1560647
16. Escobar A, Quintana JM, Bilbao A, et al. Effect of patient characteristics on reported outcomes after total knee replacement. *Rheumatology (Oxford)*. Jan 2007;46(1):112-9. doi:10.1093/rheumatology/kel184
17. Bossmann T, Brauner T, Wearing S, Horstmann T. Predictors of chronic pain following total knee replacement in females and males: an exploratory study. *Pain Manag*. Sep 2017;7(5):391-403. doi:10.2217/pmt-2017-0023
18. Bugada D, Allegri M, Gemma M, et al. Effects of anaesthesia and analgesia on long-term outcome after total knee replacement: A prospective, observational, multicentre study. *Eur J Anaesthesiol*. Oct 2017;34(10):665-672. doi:10.1097/EJA.0000000000000656

19. Chen F, Gao W, Hu J, Yang X, Chai X, Wang D. Preoperative angiotensin II type 2 receptor is a predictor for developing chronic post-surgical pain after total knee arthroplasty surgery. *Life Sci*. Aug 1 2021;278:119654. doi:10.1016/j.lfs.2021.119654
20. Fitzsimmons M, Carr E, Woodhouse L, Bostick GP. Development and Persistence of Suspected Neuropathic Pain After Total Knee Arthroplasty in Individuals With Osteoarthritis. *PM R*. Sep 2018;10(9):903-909. doi:10.1016/j.pmrj.2018.01.010
21. Yang HY, Losina E, Lange JK, Katz JN, Collins JE. Longitudinal Trajectories of Pain and Function Improvement Following Total Knee Replacement. *ACR Open Rheumatol*. Jul 2019;1(5):308-317. doi:10.1002/acr2.1041
22. Engel C, Hamilton NA, Potter PT, Zautra AJ. Impact of two types of expectancy on recovery from total knee replacement surgery (TKR) in adults with osteoarthritis. *Behav Med*. Fall 2004;30(3):113-23. doi:10.3200/BMED.30.3.113-123
23. Perruccio AV, Fitzpatrick J, Power JD, et al. The effects of depression, low back pain and comorbidities on pain after total knee arthroplasty for osteoarthritis are modified by sex. *Arthritis Care Res (Hoboken)*. 2019;72:1074-1080.
24. Cremeans-Smith JK, Greene K, Delahanty DL. Physiological Indices of Stress Prior to and Following Total Knee Arthroplasty Predict the Occurrence of Severe Post-Operative Pain. *Pain Med*. May 2016;17(5):970-9. doi:10.1093/pm/pnv043
25. Lindner M, Nosseir O, Keller-Pliessnig A, Teigelack P, Teufel M, Tagay S. Psychosocial predictors for outcome after total joint arthroplasty: a prospective comparison of hip and knee arthroplasty. *BMC Musculoskelet Disord*. May 22 2018;19(1):159. doi:10.1186/s12891-018-2058-y
26. Luo ZY, Li LL, Wang D, Wang HY, Pei FX, Zhou ZK. Preoperative sleep quality affects postoperative pain and function after total joint arthroplasty: a prospective cohort study. *J Orthop Surg Res*. Nov 21 2019;14(1):378. doi:10.1186/s13018-019-1446-9

27. Hardy A, Sandiford MH, Menigaux C, Bauer T, Klouche S, Hardy P. Pain catastrophizing and pre-operative psychological state are predictive of chronic pain after joint arthroplasty of the hip, knee or shoulder: results of a prospective, comparative study at one year follow-up. Int Orthop. 2022;46(11):2461-9.
28. Bruehl S, Milne G, Schildcrout J, Shi Y, Anderson S, Shinar A, et al. Perioperative oxidative stress predicts subsequent pain-related outcomes in the 6 months after total knee arthroplasty. Pain. 2023;164(1):111-8. Epub 20220504.
29. Edwards RR, Campbell C, Schreiber KL, Meints S, Lazaridou A, Martel MO, et al. Multimodal prediction of pain and functional outcomes 6 months following total knee replacement: a prospective cohort study. BMC Musculoskelet Disord. 2022;23(1):302. Epub 20220329. doi: 10.1186/s12891-022-05239-3.
