# Supplementary material for: Factors correlated with pain after total knee arthroplasty: A systematic review and meta-analysis
Source: PLoS One. 2023 Mar 24;18(3):e0283446. doi: 10.1371/journal.pone.0283446 (PMC10038299; doi:10.1371/journal.pone.0283446)
Supplement: S8 Appendix — (DOCX) [file pone.0283446.s009.docx]

# S8 Appendix Search strategy

**Ovid MEDLINE(R) ALL**1946 to February 03, 2023

Date of search: 06.02.2023

| **#** | **Searches** | **Results** |
| --- | --- | --- |
| 1 | Arthroplasty, Replacement, Knee/ | 30636 |
| 2 | (tkr or tjkr or tka or tjka).tw,kf. | 18000 |
| 3 | (knee* adj3 (arthroplast* or replacement*)).tw,kf. | 41118 |
| 4 | (total adj2 knee*).tw,kf. | 32626 |
| 5 | (knee* adj2 prosthes*).tw,kf. | 3258 |
| 6 | or/1-5 | 48001 |
| 7 | risk/ or risk factors/ or logistic models/ or protective factors/ or risk assessment/ | 1360889 |
| 8 | prognosis/ or (prognos* or risk* or predict*).tw,kf. | 4980096 |
| 9 | (preoperative factor* or pre operative factor* or protective factor*).tw,kf. | 29962 |
| 10 | or/7-9 | 5367177 |
| 11 | and/6,10 | 13477 |
| 12 | (pain adj3 (post* or ongoing or on going or long* or persist* or prolong* or after or follow*)).tw,kw. | 113633 |
| 13 | pain, postoperative/ | 46992 |
| 14 | (Pain/ or chronic pain/ or musculoskeletal pain/) and (post* or ongoing or on going or long* or persist* or prolonged or after or follow*).tw,kf. | 76836 |
| 15 | cohort studies/ or follow-up studies/ or longitudinal studies/ or prospective studies/ or retrospective studies/ | 2442973 |
| 16 | pain.tw,kf. | 752663 |
| 17 | and/15-16 | 138109 |
| 18 | or/12-14,17 | 283724 |
| 19 | and/11,18 | 1910 |
| 20 | (function* or stiffness or contracture*).tw,kf. | 4452133 |
| 21 | (muscle adj3 (strength* or weakness or fatigue or tonus)).tw,kf. | 58902 |
| 22 | Contracture/ | 8663 |
| 23 | "Recovery of Function"/ | 58910 |
| 24 | "Range of Motion, Articular"/ | 58856 |
| 25 | locomotion/ or walking/ or gait/ or walking speed/ or stair climbing/ | 91069 |
| 26 | "Activities of Daily Living"/ or (adl or (daily adj3 activit*)).tw,kf. | 118280 |
| 27 | Movement/ | 80149 |
| 28 | muscle fatigue/ or muscle tonus/ or physical exertion/ or postural balance/ or Muscle Strength/ | 122161 |
| 29 | (sitting or lying or standing or balance or posture or rising or neeling or bend* or walk* or gait or stair* or extension* or stability or contracture* or movement* or motion* or locomotion* or mobility or twisting or pivoting or straighten* or swelling or grinding or clicking or squatting or running or jumping).tw,kf. | 2251833 |
| 30 | treatment outcome/ or treatment failure/ or outcome*.tw,kf. | 2983936 |
| 31 | patient reported outcome measures/ | 12861 |
| 32 | ("Knee injury and Osteoarthritis Outcome Score" or womac or koos or "American Knee Society Score" or AKSS or Kellgren Lawrence).tw,kf. | 11456 |
| 33 | or/20-32 | 8657877 |
| 34 | cohort studies/ or follow-up studies/ or longitudinal studies/ or prospective studies/ or retrospective studies/ | 2442973 |
| 35 | Postoperative Period/ | 56374 |
| 36 | (post* or after or follow* or cohort* or prospectiv* or longitudinal).tw,kf. | 10311850 |
| 37 | or/34-36 | 10896319 |
| 38 | and/11,33,37 | 7633 |
| 39 | or/19,38 | 7985 |
| 40 | limit 39 to yr="2000 -Current" | 7672 |

**Embase Classic+Embase**1947 to 2023 February 03

Date of search: 06.02.2023

| **#** | **Searches** | **Results** |
| --- | --- | --- |
| 1 | knee replacement/ or total knee arthroplasty/ | 22957 |
| 2 | (tkr or tjkr or tka or tjka).tw,kw. | 21400 |
| 3 | (knee adj3 (arthroplast* or replacement*)).tw,kw. | 50170 |
| 4 | (total adj2 knee*).tw,kw. | 38764 |
| 5 | (knee* adj2 prosthes*).tw,kw. | 4208 |
| 6 | or/1-5 | 58204 |
| 7 | risk factor/ or risk/ or protection/ or risk assessment/ | 2317272 |
| 8 | prognosis/ or (prognos* or risk* or predict*).tw,kw. | 6914343 |
| 9 | "prediction and forecasting"/ or prediction/ | 507868 |
| 10 | (preoperative factor* or pre operative factor* or protective factor*).tw,kw. | 39127 |
| 11 | or/7-10 | 7451606 |
| 12 | and/6,11 | 16836 |
| 13 | (pain adj3 (post* or ongoing or on going or long* or persist* or prolong* or after or follow*)).tw,kw. | 171006 |
| 14 | postoperative pain/ | 84175 |
| 15 | (pain/ or chronic pain/ or musculoskeletal pain/) and (post* or ongoing or on going or long* or persist* or prolonged or after or follow*).tw,kw. | 265570 |
| 16 | cohort analysis/ or follow up/ or longitudinal study/ or prospective study/ or retrospective study/ | 4170976 |
| 17 | pain.tw,kw. | 1155465 |
| 18 | and/16-17 | 275426 |
| 19 | or/13-15,18 | 578231 |
| 20 | and/12,19 | 3317 |
| 21 | knee function/ or muscle function/ or muscle rigidity/ or muscle contraction/ or muscle strength/ or muscle fatigue/ or muscle function/ or muscle stretching/ or muscle weakness/ | 265146 |
| 22 | contracture/ or flexion contracture/ or joint contracture/ or muscle contracture/ | 23339 |
| 23 | convalescence/ | 61188 |
| 24 | locomotion/ or climbing/ or stair climbing/ or jumping/ or walking/ or gait/ or walking speed/ | 241313 |
| 25 | daily life activity/ or (daily life activity or actvities of daily living or adl).tw,kw. | 118044 |
| 26 | exp musculoskeletal function/ or Movement/ | 1308286 |
| 27 | joint swelling/ or grinding/ | 14041 |
| 28 | (function* or stiffness or contracture*).tw,kw. | 5718299 |
| 29 | (muscle adj3 (strength* or weakness or fatigue or tonus)).tw,kw. | 85275 |
| 30 | (sitting or lying or standing or balance or posture or rising or neeling or bend* or walk* or gait or stair* or extension* or stability or contracture* or movement* or motion* or locomotion* or mobility or twisting or pivoting or straighten* or swelling or grinding or clicking or squatting or running or jumping).tw,kw. | 2834944 |
| 31 | treatment outcome/ or treatment failure/ or patient-reported outcome/ or clinical outcome/ or outcome*.tw,kw. | 4077768 |
| 32 | "knee injury and osteoarthritis outcome score"/ or "Western Ontario and McMaster Universities Osteoarthritis Index"/ or ("Knee injury and Osteoarthritis Outcome Score" or womac or koos or "American Knee Society Score" or AKSS or Kellgren Lawrence).tw,kw. | 20831 |
| 33 | or/21-32 | 11709889 |
| 34 | cohort analysis/ or follow up/ or longitudinal study/ or prospective study/ or retrospective study.mp. [mp=title, abstract, heading word, drug trade name, original title, device manufacturer, drug manufacturer, device trade name, keyword heading word, floating subheading word, candidate term word] | 4205964 |
| 35 | postoperative period/ | 254740 |
| 36 | (post* or after or follow* or cohort* or prospectiv* or longitudinal).tw,kw. | 14683003 |
| 37 | or/34-36 | 15476165 |
| 38 | and/12,33,37 | 9600 |
| 39 | or/20,38 | 10203 |
| 40 | limit 39 to yr="2000 -Current" | 9918 |
| 41 | limit 40 to conference abstract | 2285 |
| 42 | 40 not 41 | 7633 |

**CINAHL(** Ebsco):

Date of search: 04.02.2023

| **#** | **Query** | **Limiters/Expanders** | **Results** |
| --- | --- | --- | --- |
| S1 | (MH "Arthroplasty, Replacement, Knee+") | Search modes - Boolean/Phrase | 20,138 |
| S2 | TX tkr or tjkr or tka or tjka | Search modes - Boolean/Phrase | 8,869 |
| S3 | TX knee* N3 (arthroplast* or replacement*) | Search modes - Boolean/Phrase | 20,637 |
| S4 | TX (total N2 knee*) | Search modes - Boolean/Phrase | 17,065 |
| S5 | TX (knee* N2 prosthes*) | Search modes - Boolean/Phrase | 954 |
| S6 | S1 OR S2 OR S3 OR S4 OR S5 | Search modes - Boolean/Phrase | 28,038 |
| S7 | (MH "Risk Factors") | Search modes - Boolean/Phrase | 200,050 |
| S8 | (MH "Risk Assessment") | Search modes - Boolean/Phrase | 142,105 |
| S9 | MH "Prognosis") | Search modes - Boolean/Phrase | 96,876 |
| S10 | TX prognos* or risk* or predict* or preoperative factor* or protective factor* | Search modes - Boolean/Phrase | 1,290,387 |
| S11 | S7 OR S8 OR S9 OR S10 | Search modes - Boolean/Phrase | 1,417,505 |
| S12 | S6 AND S11 | Search modes - Boolean/Phrase | 7,153 |
| S13 | ( TX pain N2 (TX (post* or ongoing or "on going" or long* or persist* or prolong* or after or follow*) ) OR (MH "Postoperative Pain") OR TX pain AND (MH "Prospective Studies+") | Search modes - Boolean/Phrase | 99,496 |
| S14 | (MH "Pain+") OR (MH "Knee Pain+") OR (MH "Muscle Pain") AND TX post* or ongoing or "on going" or long* or persist* or prolong* or after or follow* | Search modes - Boolean/Phrase | 2,124,304 |
| S15 | S13 OR S14 | Search modes - Boolean/Phrase | 2,132,304 |
| S16 | S12 AND S15 | Search modes - Boolean/Phrase | 5,540 |
| S17 | (MH "Movement") OR (MH "Hopping") OR (MH "Jumping") OR (MH "Kneeling+") OR (MH "Extension+") OR (MH "Locomotion") OR (MH "Walking+") OR (MH "Gait+") OR (MH "Step") OR (MH "Range of Motion") OR (MH "Rising") OR (MH "Sitting") OR (MH "Squatting") OR (MH "Stair Climbing") OR (MH "Standing+") OR (MH "Stretching") | Search modes - Boolean/Phrase | 99,949 |
| S18 | (MH "Muscle Fatigue") OR (MH "Muscle Strength+") OR (MH "Muscle Tonus") | Search modes - Boolean/Phrase | 33,937 |
| S19 | TX (function* or stiffness or contracture*) | Search modes - Boolean/Phrase | 561,860 |
| S20 | TX (muscle N3 (strength* or weakness or fatigue or tonus)) | Search modes - Boolean/Phrase | 23,279 |
| S21 | (MH "Contracture+") | Search modes - Boolean/Phrase | 2,333 |
| S22 | (MH "Activities of Daily Living+") | Search modes - Boolean/Phrase | 80,269 |
| S23 | TX (actvities or daily living or adl) | Search modes - Boolean/Phrase | 50,034 |
| S24 | (MH "Treatment Outcomes+") OR (MH "Fatal Outcome") OR (MH "Treatment Failure") | Search modes - Boolean/Phrase | 445,301 |
| S25 | TX outcome* | Search modes - Boolean/Phrase | 826,791 |
| S26 | TX "Knee injury and Osteoarthritis Outcome Score" or womac or koos or "American Knee Society Score" or AKSS or Kellgren Lawrence) | Search modes - Boolean/Phrase | 12,338 |
| S27 | S17 OR S18 OR S19 OR S20 OR S21 OR S22 OR S23 OR S24 OR S25 OR S26 | Search modes - Boolean/Phrase | 1,646,810 |
| S28 | (MH "Postoperative Period") OR (MH "Prospective Studies+") | Search modes - Boolean/Phrase | 631,778 |
| S29 | TX (post* or after or follow* or cohort* or prospectiv* or longitudinal) | Search modes - Boolean/Phrase | 2,075,119 |
| S30 | S28 OR S29 | Search modes - Boolean/Phrase | 2,196,707 |
| S31 | S12 AND S27 AND S30 | Search modes - Boolean/Phrase | 3,861 |
| S32 | S16 OR S31  **Limiters** - Published Date: 20000101-20211031 | Search modes - Boolean/Phrase | 5,820 |

**The Cochrane Library**

Date of search: 04.02.2023

| ID | Search | Hits |
| --- | --- | --- |
| #1 | MeSH descriptor: [Arthroplasty, Replacement, Knee] 3 tree(s) exploded | 3316 |
| #2 | (tkr or tjkr or tka or tjka):ti,ab,kw | 4092 |
| #3 | (knee near/3 (arthroplast* or replacement*)):ti,ab,kw | 9586 |
| #4 | (total near/2 knee*):ti,ab,kw | 7859 |
| #5 | (knee near/2 prosthes*):ti,ab,kw | 1370 |
| #6 | #1 or #2 or #3 or #4 or #5 | 10168 |
| #7 | MeSH descriptor: [Risk] explode all trees | 49009 |
| #8 | MeSH descriptor: [Prognosis] this term only | 18006 |
| #9 | (prognos* or risk* or predict*):ti,ab,kw | 388820 |
| #10 | ((preoperative or "pre operative" or protective) near/2 factor*):ti,ab,kw | 1736 |
| #11 | #7 or #8 or #9 or #10 | 391654 |
| #12 | #6 and #11 | 1766 |
| #13 | (pain near/3 (post* or ongoing or "on going" or long* or persist* or prolong* or after or follow*)):ti,ab,kw | 64282 |
| #14 | MeSH descriptor: [Pain, Postoperative] explode all trees | 18546 |
| #15 | MeSH descriptor: [Pain] this term only | 14420 |
| #16 | MeSH descriptor: [Chronic Pain] this term only | 3620 |
| #17 | MeSH descriptor: [Musculoskeletal Pain] this term only | 638 |
| #18 | #14 or #15 or #16 or #17 | 36641 |
| #19 | (post* or ongoing or on going or long* or persist* or prolonged or after or follow*):ti,ab,kw | 1258510 |
| #20 | #18 and #19 | 31486 |
| #21 | MeSH descriptor: [Cohort Studies] explode all trees | 181114 |
| #22 | (pain):ti,ab,kw | 219075 |
| #23 | #21 and #22 | 25123 |
| #24 | #13 or #20 or #23 | 86668 |
| #25 | #12 and #24 | 490 |
| #26 | (function* or stiffness or contracture*):ti,ab,kw | 319588 |
| #27 | (muscle near/3 (strength* or weakness or fatigue or tonus)):ti,ab,kw | 25417 |
| #28 | MeSH descriptor: [Contracture] this term only | 219 |
| #29 | MeSH descriptor: [Recovery of Function] this term only | 6333 |
| #30 | MeSH descriptor: [Range of Motion, Articular] this term only | 5864 |
| #31 | MeSH descriptor: [Locomotion] explode all trees | 10245 |
| #32 | MeSH descriptor: [Walking] explode all trees | 7052 |
| #33 | MeSH descriptor: [Activities of Daily Living] this term only | 5970 |
| #34 | ("actvities of daily living" or adl):ti,ab,kw | 4258 |
| #35 | MeSH descriptor: [Movement] this term only | 2863 |
| #36 | MeSH descriptor: [Muscle Fatigue] this term only | 1172 |
| #37 | MeSH descriptor: [Muscle Tonus] this term only | 317 |
| #38 | MeSH descriptor: [Physical Exertion] explode all trees | 4234 |
| #39 | MeSH descriptor: [Postural Balance] this term only | 3582 |
| #40 | MeSH descriptor: [Muscle Strength] this term only | 5858 |
| #41 | (sitting or lying or standing or balance or posture or rising or neeling or bend* or walk* or gait or stair* or extension* or stability or contracture* or movement* or motion* or locomotion* or mobility or twisting or pivoting or straighten* or swelling or grinding or clicking or squatting or running or jumping):ti,ab,kw | 196958 |
| #42 | MeSH descriptor: [Treatment Outcome] this term only | 164625 |
| #43 | MeSH descriptor: [Treatment Failure] this term only | 3772 |
| #44 | (outcome):ti,ab,kw | 619907 |
| #45 | MeSH descriptor: [Patient Reported Outcome Measures] this term only | 1307 |
| #46 | ("Knee injury and Osteoarthritis Outcome Score" or womac or koos or "American Knee Society Score" or AKSS or "Kellgren Lawrence"):ti,ab,kw | 7065 |
| #47 | #26 or #27 or #28 or #29 or #30 or #31 or #32 or #33 or #34 or #35 or #36 or #37 or #38 or #39 or #40 or #41 or #42 or #43 or #44 or #45 or #46 | 895827 |
| #48 | MeSH descriptor: [Cohort Studies] explode all trees | 181114 |
| #49 | MeSH descriptor: [Postoperative Period] explode all trees | 6703 |
| #50 | (post* or after or follow* or cohort* or prospectiv* or longitudinal):ti,ab,kw | 1265709 |
| #51 | #48 or #49 or #50 | 1268391 |
| #52 | #47 and #51 | 684669 |
| #53 | #12 and #52 | 1227 |
| #54 | #25 or #53 with Cochrane Library publication date Between Jan 2000 and Feb 2023 | 1275 |

**PRDro (Physiotherapy Evidence Database):**

Date of search: 06.02.2023
Antall treff: Søk 1: 178 Søk 2: 13

Search 1: Abstract & Title : total knee* replacement*, Body Part: lower leg or knee, Published since:2000

Search 2: Abstract & Title:, knee* prosthes*, Body Part: lower leg or knee, Published since: 2000
