# Supplementary material for: Factors correlated with pain after total knee arthroplasty: A systematic review and meta-analysis
Source: PLoS One. 2023 Mar 24;18(3):e0283446. doi: 10.1371/journal.pone.0283446 (PMC10038299; doi:10.1371/journal.pone.0283446)
Supplement: S9 Appendix — (DOCX) [file pone.0283446.s010.docx]

# S9 Appendix Reason for exclusion of individual studies

# Reason for exclusion of individual studies

|  | **Authors and year** | **Reason for exclusion*** |
| --- | --- | --- |
|  | Abane et al., 2015 | 1. No regression performed |
|  | Abdel et al., 2014 | 1. No regression performed |
|  | Aderinto et al., 2005 | 5. Not separate analysis for OA |
|  | Adie et al., 2012 | 1. No regression performed |
|  | Ahmed et al., 2009 | 1. No regression performed |
|  | Alentorn-Geli et al., 2013 | 1. No regression performed |
|  | Alomran, 2015 | 1. No regression performed |
|  | Amusat et al., 2014 | 7. Insufficient information about the sample** |
|  | Aso et al., 2021., 2021 | 5. Not separate analysis for TKA |
|  | Andrawis et al., 2015 | 5. Not separate analysis for TKA |
|  | Arden et al., 2017 | 3. Inadequate study design |
|  | Arendt-Nielsen et al., 2018 | 4. Predictor not evaluated |
|  | Arendt-Nielsen et al., 2018 | 2. Duplicate publication |
|  | Ayers et al., 2005 | 5. Not separate analysis for OA |
|  | Ayers et al., 2013 | 5. Not separate analysis for OA |
|  | Bade et al., 2012 | 5. Not separate analysis intervention & control |
|  | Bade et al., 2014 | 5. Not separate analysis intervention & control |
|  | Barrack et al., 2014 | 3. Ineligible study design |
|  | Barroso et al., 2020 | 7. Insufficient information on instruments** |
|  | Bascuas et al., 2013 | 1. No regression performed for the outcome |
|  | Bauer et al., 2010 | 6. Insufficient follow-up time |
|  | Behrend et al., 2019 | 3. Inadequate study design |
|  | Belford et al., 2020 | 7. Insufficient data about the sample** |
|  | Bell et al., 2022 | 3. inadequate aim |
|  | Berghmans et al., 2015 | 2. Conference abstract |
|  | Berghmans et al., 2019 | 3. Inadequate aim |
|  | Bergschmidt et al., 2008 | 1. No regression performed |
|  | Bethge et al., 2010 | 1. No regression performed |
|  | Bian et al., 2021 | 3. Inadequate aim |
|  | Bierke et al., 2017 | 1. No regression performed |
|  | Bin et al., 2007 | 1. No regression performed |
|  | Bistolfi et al., 2017 | 1. No regression performed |
|  | Blackburn et al., 2012 | 1. No regression performed |
|  | Boerger et al., 2005 | 1. No regression performed |
|  | Bove et al., 2022 | 3. Inadequate aim |
|  | Boye Larsen et al., 2021 | 5. Pooled data |
|  | Braaksma et al., 2020 | 6. Insufficient follow-up time |
|  | Brander et al., 2003 | 5. Not separate analysis for OA |
|  | Brock et al., 2017 | 5. Not separate analysis for OA |
|  | Brockenbrough et al., 2007 | 7. Insufficient data about the sample** |
|  | Browne et al., 2013 | 2. Commentary |
|  | Brummett et al., 2011 | 2. Conference abstract |
|  | Brummett et al., 2013 | 2. Conference abstract |
|  | Brummett et al., 2015 | 5. Not separate analysis for TKA |
|  | Buvanendran et al., 2011 | 2. Conference abstract |
|  | Buvanendran et al., 2012 | 2. Conference abstract |
|  | Bumberger et al., 2021 | 3. Inadequate study design |
|  | Caracciolo et al., 2005 | 3. Inadequate aim |
|  | Carriere et al., 2022 | 4. Insufficient information** |
|  | Carvalho Junior et al., 2017 | 1. No regression performed |
|  | Chalidis et al., 2010 | 1. No regression performed for the outcome |
|  | Chang et al., 2010 | 5. Not separate analysis for TKA |
|  | Cheng et al., 2010 | 3. Inadequate study design |
|  | Cheuy et al., 2019 | 1. No regression performed for the outcome |
|  | Chodor et al., | 7. Insufficient information** |
|  | Chouteau et al., 2009 | 3. Ineligible study design |
|  | Chowdhry et al., 2014 | 3. Ineligible study design |
|  | Christensen et al., 2019 | 5. Not separate data for OA |
|  | Christensen et al., 2021 | 3. Inadequate aim |
|  | Clement et al., 2013a | 4. Outcome not evaluated |
|  | Clement et al., 2013b | 4. Outcome not evaluated |
|  | Clement et al., 2011 | 1. No regression performed |
|  | Clement et al., 2013c | 5. Not separate data for pain |
|  | Collins et al., 2017 | 5. Not separate analysis intervention & control |
|  | Collins et al., 2016 | 2. Conference abstract |
|  | Cooper et al., 2017 | 5. Not separate analysis intervention & control |
|  | Cornelius et al., 2017 | 2. Conference abstract |
|  | Cornelius et al., 2015 | 2. Conference abstract |
|  | Cremeans-Smith et al., 2012 | 2. Conference abstract |
|  | Cremeans-Smith et al., 2015a | 4. Outcome not evaluated |
|  | Cremeans-Smith et al., 2013 | 2. Conference abstract |
|  | Cremeans-Smith et al., 2015b | 4. Outcome not evaluated |
|  | Cremeans-Smith et al., 2018 | 2. Conference abstract |
|  | Dailiana et al., 2015 | 2. Duplicate |
|  | Dalury et al., 2009 | 1. No regression performed for the outcome |
|  | Davis et al., 2009 | 2. Conference abstract |
|  | Davis et al., 2017 | 2. Conference abstract |
|  | Dere et al., 2014 | 1. No regression performed for the outcome |
|  | Desmeules et al., 2013 | 5. Not separate analysis for OA |
|  | Dierick et al., 2004 | 1. No regression performed for the outcome |
|  | Djadoun et al., 2014 | 2. Conference abstract |
|  | Dossett et al., 2012 | 1. No regression performed |
|  | Dowsey et al., 2009 | 5. Not separate analysis for OA |
|  | Dowsey et al., 2014 | 5. Not separate analysis for OA |
|  | Dowsey et al., 2015 | 5. Not separate analysis for OA |
|  | Dowsey et al., 2016 | 5. Not separate analysis for OA |
|  | Doury-Panchout et al., 2015 | 3. Ineligible study design |
|  | Duivenvoorden et al., 2013 | 5. Not separate analysis for OA |
|  | Dumenci et al., 2019 | 5. Not separate analysis for OA |
|  | Dursteler et al., 2021 | 1. No adequate regression performed |
|  | Dutka et al., 2011 | 5. Not separate analysis for OA |
|  | Dutton et al., 2008 | 1. No regression performed for the outcome |
|  | Edwards et al., 2009 | 4. Outcome not evaluated |
|  | Ellis et al., 2012 | 1. No regression performed |
|  | Faller et al., 2003 | 2. Letter to editor |
|  | Farahini et al., 2012 | 5. Not separate analysis for OA |
|  | Farin et al., 2006 | 5. Not separate analysis for TKA |
|  | Fernandez-Fairen et al., 2013 | 1. No regression performed for the outcome |
|  | Ferreira et al., 2021 | 5. Not separate analysis for OA |
|  | Ferrer et al., 2020 | 1. No adequate regression performed |
|  | Filardo et al., 2017 | 5. Not separate data for pain |
|  | Filbay and Judge, 2017 | 2. Conference |
|  | Filbay et al., 2018 | 5. Not separate pain score |
|  | Fitzpatrick et al., 2017 | 2. Conference |
|  | Fitzsimmons et al., 2018 | 2. Duplicate publication |
|  | Fleeton et al., 2016 | 5. Allocation groups were pooled |
|  | Foran et al., 2004 | 6. Insufficient follow-up time |
|  | Forsythe et al., 2008 | 1. No regression performed |
|  | Franklin et al., 2008 | 5. Not separate analysis for OA |
|  | Franklin et al., 2013 | 2. Conference |
|  | Furu et al., 2016 | 5. Not separate analysis for OA |
|  | Gandhi et al., 2009a | 5. Not separate analysis for OA |
|  | Gandhi et al., 2010a | 5. Not separate analysis for TKA |
|  | Gandhi et al., 2010b | 5. Not separate analysis for TKA |
|  | Gandhi et al., 2010c | 2. Duplicate publication |
|  | Gandhi et al., 2009b | 5. Not separate analysis for TKA |
|  | Gandhi et al., 2009c | 5. Not separate analysis for TKA |
|  | Gandhi et al., 2009d | 5. Not separate analysis for TKA |
|  | Gates et al., 2016 | 5. Not separate analysis for TKA |
|  | Gates et al., 2017 | 4. Outcome not evaluated |
|  | Gatha et al., 2004 | 6. Insufficient follow-up time |
|  | Giesinger et al., 2016 | 2. Conference |
|  | Giordano et al., 2021 | 1. No regression performed |
|  | Gonzalez et al., 2014 | 5. Not separate analysis for TKA |
|  | Gouveia et al., 2022 | 7. Insufficient follow-up time |
|  | Graves et al., 2014 | 1. No regression performed |
|  | Gray et al., 2017 | 5. Used the total score of WOMAC |
|  | Greco et al., 2017 | 2. Conference abstract |
|  | Greenidge et al., 2009 | 2. Conference abstract |
|  | Grosu et al., 2013 | 2. Conference abstract |
|  | Group et al., 2009 | 1. No regression performed. |
|  | Guimaraes-Pereira et al., 2016 | 5. Not separate analysis for TKA |
|  | Gøthesen et al., 2014 | 5. Not separate analysis for OA |
|  | Ha and Ha, 2006 | 7. Insufficient information about age |
|  | Halket et al., 2010 | 5. Pooled analysis TKA/THA |
|  | Hamilton et al., 2015 | 1. No regression performed |
|  | Hamilton et al., 2017 | 2. Conference abstract |
|  | Hamilton et al., 2021 | 5. Not separate measure of pain |
|  | Hanratty et al., 2011 | 1. No regression performed |
|  | Hanusch et al., 2014 | 7. Insufficient follow-up time |
|  | Harden et al., 2003 | 4. Outcome not evaluated |
|  | Hasegawa et al., 2021 | 7. Insufficient information |
|  | Hasegawa et al., 2021 | 1. No regression performed |
|  | Hashimoto et al., 2019 | 1. No regression performed |
|  | Hemert et al., 2011 | 1. No regression performed |
|  | Hinarejos et al., 2016 | 1. No regression performed |
|  | Hirschmann et al., 2010 | 1. No regression performed |
|  | Hirschmann et al., 2013 | 1. No regression performed |
|  | Hitt et al., 2015 | 1. No regression performed |
|  | Hodges et al., 2018 | 5. Outcome not evaluated |
|  | Hodges et al., 2018 | 2. Duplicate publication |
|  | Hommel et al., 2017 | 1. No regression performed. |
|  | Hofstede et al., 2018 | 3. Inadequate study design |
|  | Hofstede et al., 2018 | 2. Duplicate publication |
|  | Hooper et al., 2012 | 1. No regression performed |
|  | Hourlier et al., 2014 | 1. No regression performed |
|  | Hovik et al., 2016 | 1. No regression performed |
|  | Hughes et al., 2018 | 2. Conference abstract |
|  | Hylkema et al., 2019 | 3. Inadequate aim |
|  | Haanstra et al., 2015 | 3. Inadequate aim |
|  | Ingleshwar et al., 2013 | 2. Conference abstract |
|  | Jacobs et al., 2016a | 2. Conference abstract |
|  | Jacobs et al., 2016b | 2. Conference abstract |
|  | Jain et al., 2017 | 7. Insufficient information |
|  | Jamsen et al., 2015 | 2. Conference abstract |
|  | Jarvenpaa et al., 2010a | 1. No regression performed |
|  | Jarvenpaa et al., 2010b | 2. Duplicate |
|  | Jefferies et al., 2012 | 3. Study design |
|  | Jiang et al., 2017 | 5. Pooled analysis OA/RA |
|  | Jolles et al., 2012 | 5. Insufficient aim |
|  | Jonbergen et al., 2011 | 1. No regression performed |
|  | Jones et al., 2012a | 6. Insufficient follow-up time |
|  | Jones et al., 2003 | 5. Not separate analysis for OA |
|  | Jones et al., 2012b | 2. Conference abstract. |
|  | Judge et al., 2012 | 7. Insufficient age of participant <18 years |
|  | Judge et al., 2010 | 2. Conference abstract |
|  | Julie et al., 2013 | 2. Conference abstract |
|  | Kahlenberg et al., 2018 | 1. No regression performed |
|  | Kang et al., 2010 | 3. Inadequate study design |
|  | Katakam et al., 2021 | 3. Inadequate study design |
|  | Katz et al., 2011 | 2. Conference abstract |
|  | Keeney et al., 2017 | 3. Inadequate aim |
|  | Kelly et al., 2006 | 3. Inadequate aim |
|  | Kennedy et al., 2008 | 3. Inadequate aim |
|  | Khanna,et al., 2016 | 2. Conference abstract |
|  | Kilicarslan et al., 2011 | 1. No regression performed |
|  | Kim et al., 2015 | 5. Not separate analysis for TKA |
|  | Kim et al., 2009 | 1. No regression performed |
|  | Klasan et a., 2022 | 3. Inadequate aim |
|  | Ko et al., 2010 | 2. Conference abstract |
|  | Kurien et al., 2018 | 3. Inadequate aim |
|  | Kurien et al., 2018 | 2. Duplicate publication |
|  | Kurien et al., 2022 | 3. Study design |
|  | Lam et al., 2003 | 1. No regression performed |
|  | Lamb et al., 2003 | 1. No regression performed |
|  | Lampe et al., 2016 | 5. Pooled data intervention & control |
|  | Lange et al., 2016 | 2. Conference abstract |
|  | Larsen et al., 2021 | 5. Pooled data intervention & control |
|  | Laskow et al., 2021 | 5. Not separate analysis for OA |
|  | Lebleu et al., 2019 | 5. Not separate analysis for TKA |
|  | Ledin et al., 2012 | 1. No regression performed |
|  | Lee et al., 2015 | 5. Not separate analysis for TKA |
|  | Leung et al., 2017 | 2. Conference abstract |
|  | Leung et al., 2019 | 5. Not separate analysis for TKA |
|  | Li et al., 2013 | 2. Conference abstract |
|  | Liebs et al., 2011 | 5. Not separate analysis for TKA |
|  | Lindberg et al., 2016 | 1. No regression performed |
|  | Lindberg et al., 2020 | 3. Inadequate aim |
|  | Lindner et al., 2018 | 2. Duplicate publication |
|  | Lingard et al., 2004 | 7. Insufficient data ** |
|  | Liu et al., 2020 | 1. No regression performed |
|  | Lizaur-Utrilla et al., 2012 | 3. Inadequate aim |
|  | Lungu et al., 2014 | 1. No regression performed |
|  | Lustig et al., 2012 | 5. Pooled data, more knees than patients |
|  | Lutzner et al., 2014 | 1. No regression performed |
|  | Lützner et al., 2014 | 1. No regression performed |
|  | Macaulay et al., 2010 | 2. Conference abstract |
|  | Maculé et al., 2005 | 1. No regression performed |
|  | Maffulli et al., 2011 | 3. Inadequate study design |
|  | Magaldi et al., 2019 | 3. Inadequate aim. |
|  | Mahomed et al., 2002 | 5. Pooled data TKA and THA |
|  | Mahoney et al., 2012 | 1. No regression performed |
|  | Malviya et al., 2009 | 1. No regression performed |
|  | Martinez et al., 2007 | 1. No regression performed |
|  | Mat et al., 2016 | 3. Inadequate study design and aim |
|  | Maus et al., 2017 | 1. No regression performed |
|  | Mawarrikado et al., 2022 | 3. Inadequate study design |
|  | Maxwell et al., 2013 | 6. Insufficient follow-up time |
|  | Mehta, 2007 | 1. No regression performed |
|  | Meessen et al., 2018 | 1. No regression performed |
|  | Mehta et al., 2014 | 2. Conference abstract |
|  | Mehta et al., 2015 | 3. Inadequate study design |
|  | Meijerink et al., 2009 | 6. Insufficient follow-up time |
|  | Mercurio et al., 2020 | 5. Not separate analysis for TKA |
|  | Metsna et al., 2014 | 1. No regression performed |
|  | Miozzari et al., 2013 | 2. Conference abstract |
|  | Mittal et al., 2012 | 1. No regression performed |
|  | Mizner et al., 2005 | 3. Inadequate aim |
|  | Moghtadaei et al., 2020 | 3. Inadequate study design |
|  | Mohammad et al., 2021 | 3. Inadequate study design |
|  | Molt et al., 2014 | 1. No regression performed |
|  | Morze et al., 2013 | 1. No regression performed |
|  | Motwani et al., 2013 | 3. Inadequate study design |
|  | Nandi et al., 2016 | 2. Conference abstract |
|  | Nankaku et al., 2018 | 3. Inadequate aim |
|  | Nankaku et al., 2018 | 2. Duplicate publication |
|  | Navarro Collado et al., 2000 | 5. Pooled data OA/RA |
|  | Naylor et al., 2008 | 5. Not separate results for OA |
|  | Neogi et al., 2010 | 2. Conference abstract |
|  | Neuburger et al., 2013 | 5. Not separate results for OA |
|  | Neuprez et al., 2018 | 5. Not separate results for TKA |
|  | Neuprez et al., 2018 | 2. Duplicate publication |
|  | Nielsen et al., 2018 | 6. Insufficient follow-up time |
|  | Nielsen et al., 2018 | 2. Duplicate publication |
|  | Niki et al., 2015 | 6. Insufficient follow-up time |
|  | Noiseux et al., 2014 | 5. Pooled data from intervention & control |
|  | Nuñez et al., 2011 | 3. Inadequate study design |
|  | Nwankwo et al., 2021 | 5. Not separate results for TKA |
|  | Oatis et al., 2012 | 2. Conference abstract |
|  | Oberbek et al, 2015 | 1. No regression performed |
|  | Oka et al., 2019 | 3. Inadequate aim |
|  | Okamoto et al., 2014 | 5. Pooled data, more knees than patients |
|  | Otero et al., 2016 | 6. Insufficient follow-up time. |
|  | Ozdemir et al., 2017 | 1. No regression performed |
|  | Page et al., 2014 | 2. Conference abstract |
|  | Page et al., 2015 | 1. No regression performed |
|  | Boyet al., 2022 | 5. Pooled data |
|  | Pan et al., 2019 | 3. Inadequate study design and aim |
|  | Papakostidou et al., 2012 | 7. Insufficient data on analysis** |
|  | Parsley et al., 2010 | 1. No regression performed |
|  | Paxton et al., 2016 | 6. Insufficient follow-up time |
|  | Pereira et al., 2016 | 5. Not separate results for TKA |
|  | Perruccio et al., 2010a | 5. Not separate results for TKA |
|  | Perruccio et al., 2010b | 2. Duplicate publication |
|  | Perruccio et al., 2011a | 1. No regression performed. |
|  | Perruccio et al., 2011b | 2. Conference abstract |
|  | Petersen et al, 2018 | 2. Duplicate publication |
|  | Pinsornsak et al., 2014 | 1. No regression performed |
|  | Pinto et al., 2013 | 5. Not separate results for TKA |
|  | Pinto et al., 2014 | 2. Conference abstract |
|  | Polkowski et al., 2013 | 6. Insufficient follow-up time |
|  | Pont et al., 2011 | 1. No regression performed |
|  | Pua et al., 2012 | 2. Conference abstract |
|  | Pua et al., 2017 | 4. Prognostic factor not evaluated |
|  | Pua et al., 2013 | 4. Prognostic factor not evaluated |
|  | Pua et al., 2015 | 1. No regression performed |
|  | Pua et al., 2016 | 2. Sample as prior study we used data from |
|  | Quintana et al., 2006 | 5. Not separate results for TKA |
|  | Radmer et al., 2006 | 5. Not separate results for OA |
|  | Rajamaki et al., 2015 | 5. Not separate results for OA |
|  | Rakel et al., 2013 | 2. Conference abstract |
|  | Ramaesh et al., 2014 | 5. Not separate results for OA |
|  | Razmjou et al., 2015 | 6. Insufficient follow-up time |
|  | Reid et al., 2014 | 3. Inadequate aim |
|  | Rice et al., 2018 | 5. Not separate results for OA |
|  | Richards et al., 2016 | 2. Conference abstract |
|  | Riddle et al., 2009 | 2. Conference abstract |
|  | Riddle et al., 2015 | 1. No regression performed |
|  | Riddle et al., 2018 | 5. Not separate results for TKA |
|  | Riddle et al., 2020 | 5. Pooled results from RCT |
|  | Rosen et al., 2013 | 3. Inadequate aim |
|  | Russell et al., 2014 | 1. No regression performed |
|  | Sakellariou et al., 2016 | 2. Conference abstract |
|  | Salazar et al., 2013 | 2. Conference abstract |
|  | Sanchez-Santos et al., 2014 | 2. Conference abstract |
|  | Sanchez-Santos et al., 2018 | 5. Not separate results for OA |
|  | Schaumburger et al., 2012 | 1. No regression performed |
|  | Schwartz et al., 2012 | 4. Prognostic factor not evaluated |
|  | Scott et al., 2010 | 4. Outcome not evaluated |
|  | Scott et al., 2012 | 4. Outcome not evaluated |
|  | Seol et al., 2016 | 3. Inadequate study design |
|  | Sharma et al., 2021 | 7. Insufficient data results are for TKA** |
|  | Shim et al., 2018 | 7. Age < 18 years |
|  | Sinclair et al., 2022 | 3. Inadequate study design |
|  | Singh et al., 2015 | 3. Inadequate study design |
|  | Siviero et al., 2020 | 7. Age < 18 years |
|  | Slevin et al., 2017 | 1. No regression performed |
|  | Smith et al., 2006 | 6. Insufficient follow-up time |
|  | Smith et al., 2014 | 5. Not separate results for TKA |
|  | Smith et al., 2012 | 1. No regression performed |
|  | Smith et al., 2019 | 6. Insufficient follow-up time |
|  | Smith et al., 2019 | 2. Duplicate publication |
|  | Soni et al., 2014 | 2. Conference abstract |
|  | Soni et al., 2016 | 2. Conference abstract |
|  | Stickles et al., 2001 | 1. No regression performed |
|  | Stone et al., 2017 | 3. Inadequate study design |
|  | Stratford et al., 2010 | 6. Insufficient follow-up time |
|  | Street et al., 2018 | 3. Inadequate study design |
|  | Sun et al., 2019 | 2. Letter to editor |
|  | Sugawara et al., 2017 | 3. Inadequate aim |
|  | Sveikata et al., 2017 | 1. No regression performed |
|  | Tabutin et al., 2005 | 1. No regression performed |
|  | Tan et al., 2014 | 5. Pooled results, more knees than patients. |
|  | Taniguchi et al., 2016 | 3. Inadequate aim |
|  | Tchetina et al., 2020 | 1. No regression performed |
|  | Thomazeau et al., 2016 | 5. Not separate results for TKA |
|  | Tilbury et al., 2016 | 6. Insufficient data |
|  | Tilbury et al., 2018 | 2. Duplicate publication |
|  | Toguchi et al., 2020 | 3. Inadequate study design |
|  | Tolk et al., 2020 | 1. No regression performed |
|  | Trace, 2006 | 3. Inadequate study design |
|  | Twiggs et al., 2019 | 3. Inadequate aim |
|  | Utrillas-Compaired et al., 2014 | 5. Not separate results for OA |
|  | Vaegter et al., 2017 | 1. No regression reported |
|  | van den Akker-Scheek et al., 2007 | 5. Not separate results for TKA |
|  | Van Hamersveld et al., 2018 | 4. Inadequate outcome |
|  | Van Hamersveld et al., 2018 | 4. Inadequate outcome |
|  | Van Hamersveld et al., 2018 | 2. Duplicate |
|  | van Loon et al., 2021 | 6. Insufficient follow-up time |
|  | Van Onsem et al., 2018 | 4. Inadequate outcome |
|  | Vekama et al., 2015 | 5. Not separate results for TKA |
|  | Vela et al., 2017 | 2. Conference abstract |
|  | Vila et al., 2020 | 5. Not separate analysis for TKA |
|  | Vina et al., 2014 | 2. Conference abstract |
|  | Vina et al., 2016 | 6. Insufficient follow-up time |
|  | Vogel et al., 2019 | 7. Insufficient data about eligibility |
|  | Wada et al., 2016 | 1. No regression reported |
|  | Walker et al., 2015 | 2. Conference abstract |
|  | Wenjun et al., 2017 | 3. Inadequate aim |
|  | Widmer et al., 2013 | 5. Not separate analysis for TKA |
|  | Williams et al., 2013 | 5. Not separate analysis for TKA |
|  | Winters et al., 2014 | 3. Inadequate study design |
|  | Wohlrab et al., 2005 | 1. No regression reported |
|  | Wollmerstedt et al., 2006 | 5. Not separate analysis for TKA |
|  | Woo et al., 2006 | 6. Data from article was unavailable |
|  | Wood et al., 2021 | 5. Not separate analysis for TKA |
|  | Wright et al., 2017 | 5. Not separate analysis for TKA |
|  | Wylde et al., 2013 | 1. No regression performed for the outcome |
|  | Wylde et al., 2015 | 5. Pooled results intervention and control arm |
|  | Wylde et al., 2017 | 2. Sample as prior study we used data from |
|  | Xu et al., 2020 | 5. Not separate measure of pain |
|  | Yakobov et al., 2018 | 4. Inadequate outcome |
|  | Yap et al., 2021 | 5. Not separate measure of pain |
|  | Yau et al., 2005 | 5. Not separate results for OA. |
|  | Young et al., 2017 | 3. Inadequate aim |
|  | Young-Shand et al., 2021 | 3. Inadequate aim |
|  | Zeni et al., 2010 | 5. Pooled results intervention & control |

Abbreviations: OA; osteoarthritis, RA; rheumatoid arthrithis, TKA; total knee arthroplasty. THA; total hip arthroplasty

*Reason for exclusion correspond with the PRISMA flow diagram. **Author did not respond to e-mail or gave insufficient information about the study

**Reference list for the excluded studies**

1. Abane, L., Anract, P., Boisgard, S., Descamps, S., Courpied, J. & Hamadouche, M. 2015. A Comparison Of Patient-Specific And Conventional Instrumentation For Total Knee Arthroplasty: A Multicentre Randomised Controlled Trial. *The Bone & Joint Journal,* 97**,** 56-63.
2. Abdel, M., Parratte, S., Blanc, G., Ollivier, M., Pomero, V., Viehweger, E. & Argenson, J. 2014. No Benefit Of Patient-Specific Instrumentation In Tka On Functional And Gait Outcomes: A Randomized Clinical Trial. 472.
3. Aderinto, J., Brenkel, I. J. & Chan, P. 2005. Natural History Of Fixed Flexion Deformity Following Total Knee Replacement: A Prospective Five-Year Study. *Journal Of Bone & Joint Surgery - British Volume,* 87**,** 934-6.
4. Adie, S., Dao, A., Harris, I. A., Naylor, J. M. & Mittal, R. 2012. Satisfaction With Joint Replacement In Public Versus Private Hospitals: A Cohort Study. *Anz Journal Of Surgery,* 82**,** 616-24.
5. Ahmed, I., Gray, A., Linden, M. & Nutton, R. 2009. Range Of Flexion After Primary Tka: The Effect Of Soft Tissue Release And Implant Design. *Orthopedics,* 32**,** 811.
6. Alentorn-Geli, E., Leal-Blanquet, J., Guirro, P., Hinarejos, P., Pelfort, X. & Puig-Verdié, L. 2013. Comparison Of Quality Of Life Between Elderly Patients Undergoing Tka. *Orthopedics,* 36**,** 253-253.
7. Alomran, A. 2015. Effect Of Patellar Denervation On Mid-Term Results After Non-Resurfaced Total Knee Arthroplasty. A Randomised, Controlled Trial. *Acta Orthopaedica Belgica,* 81**,** 609-613.
8. Amusat, N., Beaupre, L., Jhangri, G. S., Pohar, S. L., Simpson, S., Warren, S. & Jones, C. A. 2014. Diabetes That Impacts On Routine Activities Predicts Slower Recovery After Total Knee Arthroplasty: An Observational Study. *Journal Of Physiotherapy,* 60**,** 217-23.
9. Aso K, Ikeuchi M, Takaya S, Et Al. Chronic Postsurgical Pain After Total Knee Arthroplasty: A Prospective Cohort Study In Japanese Population. *Modern Rheumatology*. 2021;31(5):1038-1044.
10. Andrawis, J., Akhavan, S., Chan, V., Lehil, M., Pong, D. & Bozic, K. J. 2015. Higher Preoperative Patient Activation Associated With Better Patient-Reported Outcomes After Total Joint Arthroplasty. *Clinical Orthopaedics & Related Research,* 473**,** 2688-97.
11. Arden, N., Altman, D., Beard, D., Carr, A., Clarke, N., Collins, G., Cooper, C., Culliford, D., Delmestri, A., Garden, S., Griffin, T., Javaid, K., Judge, A., Latham, J., Mullee, M., Murray, D., Ogundimu, E., Pinedo-Villanueva, R., Price, A., Prieto-Alhambra, D. & Raftery, J. 2017. *Nihr Journals Library. Programme Grants For Applied Research,* 6**,** 06.
12. Arendt-Nielsen, L., Simonsen, O., Laursen, M. B., Roos, E. M., Rathleff, M. S., Rasmussen, S. & Skou, S. T. 2018. Pain And Sensitization After Total Knee Replacement Or Nonsurgical Treatment In Patients With Knee Osteoarthritis: Identifying Potential Predictors Of Outcome At 12 Months. *European Journal Of Pain,* 22**,** 1088-1102.
13. Arendt-Nielsen, L., Simonsen, O., Laursen, M. B., Roos, E. M., Rathleff, M. S., Rasmussen, S. & Skou, S. T. 2018. Pain And Sensitization After Total Knee Replacement Or Nonsurgical Treatment In Patients With Knee Osteoarthritis: Identifying Potential Predictors Of Outcome At 12 Months. *European Journal Of Pain,* 22**,** 1088-1102.
14. Ayers, D. C., Franklin, P. D., Ploutz-Snyder, R. & Boisvert, C. B. 2005. Total Knee Replacement Outcome And Coexisting Physical And Emotional Illness. *Clinical Orthopaedics & Related Research,* 440**,** 157-61.
15. Ayers, D. C., Li, W., Oatis, C., Rosal, M. C. & Franklin, P. D. 2013. Patient-Reported Outcomes After Total Knee Replacement Vary On The Basis Of Preoperative Coexisting Disease In The Lumbar Spine And Other Nonoperatively Treated Joints,The Neede For A Musculoskeletal Comorbidity Index. *Journal Of Bone And Joint Surgery - Series A,* 95**,** 1833-1837.
16. Bade, M., Kittelson, J., Kohrt, W. & Stevens-Lapsley, J. 2014. Predicting Functional Performance And Range Of Motion Outcomes After Total Knee Arthroplasty. *American Journal Of Physical Medicine & Rehabilitation,* 93**,** 579-585.
17. Bade, M. J., Wolfe, P., Zeni, J. A., Stevens-Lapsley, J. E. & Snyder-Mackler, L. 2012. Predicting Poor Physical Performance After Total Knee Arthroplasty. *Journal Of Orthopaedic Research,* 30**,** 1805-10.
18. Barrack, R. L., Ruh, E. L., Chen, J., Lombardi Jr, A. V., Berend, K. R., Parvizi, J., Della Valle, C. J., Hamilton, W. G. & Nunley, R. M. 2014. Impact Of Socioeconomic Factors On Outcome Of Total Knee Arthroplasty Knee. *Clinical Orthopaedics And Related Research,* 472**,** 86-97.
19. Barroso J, Wakaizumi K, Reckziegel D, Et Al. Prognostics For Pain In Osteoarthritis: Do Clinical Measures Predict Pain After Total Joint Replacement? *Plos One*. 2020;15(1):E0222370. Doi:10.1371/Journal.Pone.0222370
20. Bascuas, I., Tejero, M., Monleon, S., Boza, R., Muniesa, J. M. & Belmonte, R. 2013. Balance 1 Year After Tka: Correlation With Clinical Variables. *Orthopedics,* 36**,** E6-12.
21. Bauer, T., Biau, D., Colmar, M., Poux, X., Hardy, P. & Lortat-Jacob, A. 2010. Influence Of Posterior Condylar Offset On Knee Flexion After Cruciate-Sacrificing Mobile-Bearing Total Knee Replacement: A Prospective Analysis Of 410 Consecutive Cases. *Knee,* 17**,** 375-80.
22. Behrend H, Graulich T, Gerlach R, Spross C, Ladurner A. Blackburne-Peel Ratio Predicts Patients' Outcomes After Total Knee Arthroplasty. *Knee Surgery, Sports Traumatology, Arthroscopy*. 2019;27(5):1562-1569.
23. Belford K, Gallagher N, Dempster M, Et Al. Psychosocial Predictors Of Outcomes Up To One Year Following Total Knee Arthroplasty. *Knee*. Jun 2020;27(3):1028-1034. Doi:10.1016/J.Knee.2020.03.006
24. Bell JA, Emara AK, Barsoum WK, Bloomfield M, Briskin I, Higuera C, et al. Should an Age Cutoff Be Considered for Elective Total Knee Arthroplasty Patients? An Analysis of Operative Success Based on Patient-Reported Outcomes. J Knee Surg. 2022;10:10. Epub 20220610. doi: 10.1055/s-0042-1748821. PubMed PMID: 35688440.
25. Berghmans Ddp, Lenssen Af, Emans Pj, Van Rhijn Lw, De Bie Ra. Limited Predictive Value Of Pre-Surgical Level Of Functioning For Functioning At 3 And 12 Months After Tka. *Knee Surg Sports Traumatol Arthrosc*. May 2019;27(5):1651-1657. Doi:10.1007/S00167-018-5288-5
26. Berghmans, D., Lenssen, A., De Bie, R. A. & Van Rhijn, L. 2015. Functioning With Knee Osteoarthritis And One Year After A Tka: Are We Able To Predict Functional Recovery? *Physiotherapy United Kingdom,* 1**,** Es140-Es141.
27. Bergschmidt, P., Bader, R., Finze, S., Ansorge, S., Kundt, G. & Mittelmeier, W. 2008. [Bicondylar Knee Arthroplasty - Influence Of Preoperative Functional Restriction On Early Functional Postoperative Outcome]. *Zeitschrift Fur Orthopadie & Unfallchirurgie,* 146**,** 344-51.
28. Bethge, M., Bartel, S., Streibelt, M., Lassahn, C. & Thren, K. 2010. [Illness Perceptions And Functioning Following Total Knee And Hip Arthroplasty]. *Zeitschrift Fur Orthopadie & Unfallchirurgie,* 148**,** 387-92.
29. Bian T, Shao H, Zhou Y, Huang Y, Song Y. Does Psychological Distress Influence Postoperative Satisfaction And Outcomes In Patients Undergoing Total Knee Arthroplasty? A Prospective Cohort Study. *Bmc Musculoskeletal Disorders*. 2021;22(1):647.
30. Bierke, S. & Petersen, W. 2017. Influence Of Anxiety And Pain Catastrophizing On The Course Of Pain Within The First Year After Uncomplicated Total Knee Replacement: A Prospective Study. *Archives Of Orthopaedic & Trauma Surgery,* 137**,** 1735-1742.
31. Bin, S. I. & Nam, T. S. 2007. Early Results Of High-Flex Total Knee Arthroplasty: Comparison Study At 1 Year After Surgery. *Knee Surgery, Sports Traumatology, Arthroscopy,* 15**,** 350-5.
32. Bistolfi, A., Bettoni, E., Aprato, A., Milani, P., Berchialla, P., Graziano, E., Massazza, G. & Lee, G. C. 2017. The Presence And Influence Of Mild Depressive Symptoms On Post-Operative Pain Perception Following Primary Total Knee Arthroplasty. *Knee Surgery, Sports Traumatology, Arthroscopy,* 25**,** 2792-2800.
33. Blackburn, J., Qureshi, A., Amirfeyz, R. & Bannister, G. 2012. Does Preoperative Anxiety And Depression Predict Satisfaction After Total Knee Replacement? *Knee,* 19**,** 522-4.
34. Boerger, T. O., Aglietti, P., Mondanelli, N. & Sensi, L. 2005. Mini-Subvastus Versus Medial Parapatellar Approach In Total Knee Arthroplasty. *Clinical Orthopaedics & Related Research,* 440**,** 82-7.
35. Bove, A.M, Hausmann L.R.M, Piva S.R, Brach J.S, Lewis A, Fitzgerald G.K. Race Differences In Postacute Physical Therapy Utilization And Patient-Reported Function After Total Knee Arthroplasty. *Arthritis Care Res (Hoboken)*. Jan 2022;74(1):79-88. Doi:10.1002/Acr.24792
36. Boye Larsen D, Laursen M, Simonsen O, Arendt-Nielsen L, Petersen KK. The association between sleep quality, preoperative risk factors for chronic postoperative pain and postoperative pain intensity 12 months after knee and hip arthroplasty. Br J Pain. 2021;15(4):486-96.doi: 177/20494637211005803.
37. Braaksma, C., Oehlers, V., Veen Mr., Wolterbeek, N. Patient Characteristics Do Not Predict The Change In Physical Functioning Following Arthroplasty Measured By The Hoos-Ps And Koos-Ps. *Journal Of Orthopaedics*. 2020;20:122-124.
38. Brander, V. A., David Stulberg, S., Adams, A. D., Harden, R. N., Bruehl, S., Stanos, S. P. & Houle, T. 2003. Predicting Total Knee Replacement Pain: A Prospective, Observational Study. *Clinical Orthopaedics And Related Research***,** 27-36.
39. Brock, T. M., Shirley, M., Bardgett, M., Walker, M. & Deehan, D. J. 2017. Inadequate Pre-Operative Glycaemic Control In Patients With Diabetes Mellitus Adversely Influences Functional Recovery After Total Knee Arthroplasty : Patients With Impaired Glycaemic Control Exhibit Poorer Functional Outcomes At 1-Year Post-Arthroplasty. *Knee Surgery, Sports Traumatology, Arthroscopy,* 25**,** 1801-1806.
40. Brockenbrough, G. 2007. Radiographic Severity Of Oa May Not Predict Tka Outcomes. *Orthopedics Today,* 27**,** 9-9.
41. Browne, J. A. 2013. Movement Pain, Resting Pain And Depression Prior To Total Knee Replacement Predict Postoperative Pain. *Evidence-Based Nursing,* 16**,** 115-6.
42. Brummett, C. M., Hallstrom, B., Urquhart, A., Morris, M., Clauw, D. J. & Williams, D. A. 2011. Psychological Predictors Of Failure To Improve After Lower Extremity Joint Arthroplasty. *Arthritis And Rheumatism. Conference: Annual Scientific Meeting Of The American College Of Rheumatology And Association Of Rheumatology Health Professionals,* 63.
43. Brummett, C. M., Urquhart, A., Hallstrom, B., Tsodikov, A., Williams, D. A. & Clauw, D. J. 2013. The Impact Of Centralized Pain On Long-Term Analgesic Response To Lower Extremity Joint Arthroplasty: A Prospective, Observational Cohort Study. *Arthritis And Rheumatism,* 10**,** S898-S899.
44. Brummett, C. M., Urquhart, A. G., Hassett, A. L., Tsodikov, A., Hallstrom, B. R., Wood, N. I., Williams, D. A. & Clauw, D. J. 2015. Characteristics Of Fibromyalgia Independently Predict Poorer Long-Term Analgesic Outcomes Following Total Knee And Hip Arthroplasty. *Arthritis & Rheumatology,* 67**,** 1386-1394.
45. Buvanendran, A., Moric, M., Kroin, J. & Tuman, K. J. 2011. Risk Factors And Mechanisms For Persistent Postsurgical Pain After Total Knee Replacement. *Anesthesia And Analgesia. Conference,* 112.
46. Buvanendran, A., Moric, M., Kroin, J. & Tuman, K. J. 2012. Persistent Postsurgical Pain After Total Knee Replacement: Preoperative Health Status As A Risk Factor? *Anesthesia And Analgesia,* 1**,** S374.
47. Bumberger A, Borst K, Hobusch GM, Et Al. Higher Patient Knowledge And Resilience Improve The Functional Outcome Of Primary Total Knee Arthroplasty. *Wiener Klinische Wochenschrift*. 2021;133(11-12):543-549.
48. Caracciolo, B. & Giaquinto, S. 2005. Determinants Of The Subjective Functional Outcome Of Total Joint Arthroplasty. *Archives Of Gerontology & Geriatrics,* 41**,** 169-176.
49. Carriere JS, Martel MO, Loggia ML, et al. The Influence of Expectancies on Pain and Function Over Time After Total Knee Arthroplasty. Research Support, N.I.H., Extramural. *Pain Med*. Sep 30 2022;23(10):1767-1776.
50. Carvalho Junior, L. H., Teixeira, B. P., Bernardes, C. O., Soares, L. F., Goncalves, M. B. & Temponi, E. F. 2017. Range Of Motion Predictability After Total Knee Arthroplasty With Medial Pivot Prosthesis. *Revista Brasileira De Ortopedia,* 52**,** 197-202.
51. Chalidis, B. E., Petsatodis, G., Christodoulou, A. G., Christoforidis, J., Papadopoulos, P. P. & Pournaras, J. 2010. Is Obesity A Contraindication For Minimal Invasive Total Knee Replacement? A Prospective Randomized Control Trial. *Obesity Surgery,* 20**,** 1633-41.
52. Chang, C. B., Yoo, J. H., Koh, I. J., Kang, Y. G., Seong, S. C. & Kim, T. K. 2010. Key Factors In Determining Surgical Timing Of Total Knee Arthroplasty In Osteoarthritic Patients: Age, Radiographic Severity, And Symptomatic Severity. *Journal Of Orthopaedics & Traumatology,* 11**,** 21-7.
53. Cheng, T., Liu, T., Zhang, G., Peng, X. & Zhang, X. 2010. Does Minimally Invasive Surgery Improve Short-Term Recovery In Total Knee Arthroplasty? *Clinical Orthopaedics & Related Research,* 468**,** 1635-1648.
54. Cheuy, V. A., Loyd, B. J., Hafner, W., Kittelson, A. J., Waugh, D. & Stevens-Lapsley, J. E. 2019. Influence Of Diabetes Mellitus On The Recovery Trajectories Of Function, Strength, And Self-Report Measures After Total Knee Arthroplasty. *Arthritis Care And Research.*
55. Chodor P, Kruczynski J. Preoperative Risk Factors of Persistent Pain following Total Knee Arthroplasty. *Biomed Res Int*. 2022;2022:4958089.
56. Chouteau, J., Lerat, J. L., Testa, R., Moyen, B., Fessy, M. H. & Banks, S. A. 2009. Kinematics Of A Cementless Mobile Bearing Posterior Cruciate Ligament-Retaining Total Knee Arthroplasty. *Knee,* 16**,** 223-7.
57. Chowdhry, M., Bamne, A. B., Na, Y. G., Kang, Y. G. & Kim, T. K. 2014. Prevalence And Predictors Of Post-Operative Coronal Alignment Outliers And Their Association With The Functional Outcomes In Navigated Total Knee Arthroplasty. *Journal Of Arthroplasty,* 29**,** 2357-62.
58. Christensen, J. C., Mizner, R. L., Foreman, K. B., Lastayo, P. C., Peters, C. L. & Pelt, C. E. 2019. Preoperative Quadriceps Weakness Preferentially Predicts Postoperative Aberrant Movement Patterns During High-Demand Mobility Following Total Knee Arthroplasty. *The Knee,* 26**,** 79.
59. Christensen J, Peters C, Gililland J, Stoddard G, Pelt C. Physical Activity, Pain Interference And Comorbidities Relate To PROMIS Physical Function In Younger Adults Following Total Knee Arthroplasty. *Disabil Rehabil*. Dec 2021;43(26):3741-3747. Doi:10.1080/09638288.2020.1749944
60. Clement, N. D., Jenkins, P. J., Macdonald, D., Nie, Y. X., Patton, J. T., Breusch, S. J., Howie, C. R. & Biant, L. C. 2013a. Socioeconomic Status Affects The Oxford Knee Score And Short-Form 12 Score Following Total Knee Replacement. *Bone & Joint Journal,* 95**,** 52-8.
61. Clement, N. D., Macdonald, D., Burnett, R. & Breusch, S. J. 2013b. Diabetes Does Not Influence The Early Outcome Of Total Knee Replacement: A Prospective Study Assessing The Oxford Knee Score, Short Form 12, And Patient Satisfaction. *Knee,* 20**,** 437-41.
62. Clement, N. D., Macdonald, D., Howie, C. R. & Biant, L. C. 2011. The Outcome Of Primary Total Hip And Knee Arthroplasty In Patients Aged 80 Years Or More. *Journal Of Bone & Joint Surgery - British Volume,* 93**,** 1265-70.
63. Clement, N. D., Macdonald, D., Simpson, A. H. R. W. & Burnett, R. 2013c. Total Knee Replacement In Patients With Concomitant Back Pain Results In A Worse Functional Outcome And A Lower Rate Of Satisfaction. *Bone And Joint Journal,* 95**,** 1632-1639.
64. Collins, J. E., Donnell-Fink, L. A., Yang, H. Y., Usiskin, I. M., Lape, E. C., Wright, J., Katz, J. N. & Losina, E. 2017. Effect Of Obesity On Pain And Functional Recovery Following Total Knee Arthroplasty. *Journal Of Bone & Joint Surgery - American Volume,* 99**,** 1812-1818.
65. Collins, J. E., Yang, H. Y., Usiskin, I. M., Katz, J. N. & Losina, E. 2016. Does Morbid Obesity Negatively Affect Patient Reported Outcomes Following Total Knee Arthroplasty? *Arthritis And Rheumatology,* 68**,** 4003-4004.
66. Cooper, N. A., Rakel, B. A., Zimmerman, B., Tonelli, S. M., Herr, K. A., Clark, C. R., Noiseux, N. O., Callaghan, J. J. & Sluka, K. A. 2017. Predictors Of Multidimensional Functional Outcomes After Total Knee Arthroplasty. *Journal Of Orthopaedic Research,* 35**,** 2790-2798.
67. Cornelius, M., Edwards, R., Lazaridou, A. & Franceschelli, O. 2017. Pain Catastrophizing Predicts Post-Surgical Changes In Physical Functioning In Total Knee Replace-Ment Patients. *Journal Of Pain,* 18**,** S46.
68. Cornelius, M., Walker, J., Pejsa, M., Hand, M., Campbell, C., Haythornthwaite, J., Khanuja, P., Sterling, R., Smith, M. & Edwards, R. 2015. Pre-Surgical Quantitative Sensory Testing Predicts Persistent Postoperative Pain In Total Knee Replacement Patients. *Journal Of Pain,* 1**,** S26.
69. Cremeans-Smith, J. K., Contrera, K., Speering, L., Miller, E. T., Pfefferle, K. & Delahanty, D. L. 2012. Using Established Predictors Of Post-Traumatic Stress To Explain Variations In Recovery Outcomes Among Orthopaedic Patients. *Psychosomatic Medicine,* 74**,** A22-A23.
70. Cremeans-Smith, J. K., Contrera, K., Speering, L., Miller, E. T., Pfefferle, K., Greene, K. & Delahanty, D. L. 2015a. Using Established Predictors Of Post-Traumatic Stress To Explain Variations In Recovery Outcomes Among Orthopedic Patients. *Journal Of Health Psychology,* 20**,** 1296-304.
71. Cremeans-Smith, J. K., Greene, K. & Delahanty, D. L. 2013. Post-Operative Pain: Does Trauma History Exacerbate Or Mollify The Experience? *Psychosomatic Medicine,* 75**,** A46-A47.
72. Cremeans-Smith, J. K., Greene, K. & Delahanty, D. L. 2015b. Trauma History As A Resilience Factor For Patients Recovering From Total Knee Replacement Surgery. *Psychology & Health,* 30**,** 1005-16.
73. Cremeans-Smith, J. K., Greene, K. & Delahanty, D. L. 2018. Application Of The Cortisol-Crp Ratio To A Surgical Population: Predicting Depression, Pain, And Stress Reactivity Among Patients Undergoing Total Knee Arthroplasty. *Psychosomatic Medicine,* 80**,** A127.
74. Dailiana, Z. H., Papakostidou, I., Varitimidis, S., Liaropoulos, L., Zintzaras, E., Karachalios, T., Michelinakis, E. & Malizos, K. N. 2015. Patient-Reported Quality Of Life After Primary Major Joint Arthroplasty: A Prospective Comparison Of Hip And Knee Arthroplasty. *Bmc Musculoskeletal Disorders,* 16**,** 366.
75. Dalury, D. F., Mulliken, B. D., Adams, M. J., Lewis, C., Sauder, R. R., Bushey, J. A., Dalury, D. F., Mulliken, B. D., Adams, M. J., Lewis, C., Sauder, R. R. & Bushey, J. A. 2009. Early Recovery After Total Knee Arthroplasty Performed With And Without Patellar Eversion And Tibial Translation. A Prospective Randomized Study. *Journal Of Bone & Joint Surgery, American Volume,* 91**,** 1339-1343.
76. Davis, A., Badley, E. M., Hogg-Johnson, S., Ibrahim, S., Perruccio, A. V., Wong, R. & Beaton, D. E. 2009. Understanding Early Recovery Following Primary Total Hip And Knee Replacement. *Arthritis And Rheumatism,* 10**,** 1938.
77. Davis, A., Ibrahim, S., Hogg-Johnson, S., Wong, R., Beaton, D., Chesworth, B., Gandhi, R., Mahomed, N., Perruccio, A., Rajgopal, V. & Waddell, J. 2017. Achieving Important Improvement In Womac Pain And Function Impacts Satisfaction 1 Year Following Total Knee Replacement. *Osteoarthritis And Cartilage,* 25**,** S218.
78. Dere, D., Paker, N., Soy Bugdayci, D. & Tekdos Demircioglu, D. 2014. Effect Of Body Mass Index On Functional Recovery After Total Knee Arthroplasty In Ambulatory Overweight Or Obese Women With Osteoarthritis. *Acta Orthopaedica Et Traumatologica Turcica,* 48**,** 117-21.
79. Desmeules, F., Dionne, C. E., Belzile, E. L., Bourbonnais, R., Champagne, F. & Fremont, P. 2013. Determinants Of Pain, Functional Limitations And Health-Related Quality Of Life Six Months After Total Knee Arthroplasty: Results From A Prospective Cohort Study. *Bmc Sports Science, Medicine And Rehabilitation,* 5**,** 2.
80. Dierick, F., Aveniere, T., Cossement, M., Poilvache, P., Lobet, S. & Detrembleur, C. 2004. Outcome Assessment In Osteoarthritic Patients Undergoing Total Knee Arthroplasty. *Acta Orthopaedica Belgica,* 70**,** 38-45.
81. Djadoun, S., France, M. N., Grosu, I., Thienpont, E. & Lavand'homme, P. 2014. Impact Of Diabetes Mellitus On Nature And Quality Of Persistent Pain After Total Knee Arthroplasty. *Regional Anesthesia And Pain Medicine,* 1**,** E286.
82. Dossett, H. G., Swartz, G. J., Estrada, N. A., Lefevre, G. W. & Kwasman, B. G. 2012. Kinematically Versus Mechanically Aligned Total Knee Arthroplasty. *Orthopedics,* 35**,** E160-9.
83. Dowsey, M. M., Broadhead, M. L., Stoney, J. D. & Choong, P. F. 2009. Outcomes Of Total Knee Arthroplasty In English- Versus Non-English-Speaking Patients. *Journal Of Orthopaedic Surgery,* 17**,** 305-9.
84. Dowsey, M. M., Nikpour, M. & Choong, P. F. 2014. Outcomes Following Large Joint Arthroplasty: Does Socio-Economic Status Matter? *Bmc Musculoskeletal Disorders,* 15**,** 148.
85. Dowsey, M. M., Smith, A. J. & Choong, P. F. M. 2015. Latent Class Growth Analysis Predicts Long Term Pain And Function Trajectories In Total Knee Arthroplasty: A Study Of 689 Patients. *Osteoarthritis & Cartilage,* 23**,** 2141-2149.
86. Dowsey, M. M., Spelman, T. & Choong, P. F. M. 2016. Development Of A Prognostic Nomogram For Predicting The Probability Of Nonresponse To Total Knee Arthroplasty 1 Year After Surgery. *Journal Of Arthroplasty,* 31**,** 1654-1660.
87. Doury-Panchout, F., Metivier, J. C. & Fouquet, B. 2015. Kinesiophobia Negatively Influences Recovery Of Joint Function Following Total Knee Arthroplasty. *European Journal Of Physical & Rehabilitation Medicine.,* 51**,** 155-61.
88. Duivenvoorden, T., Vissers, M. M., Verhaar, J. A., Busschbach, J. J., Gosens, T., Bloem, R. M., Bierma-Zeinstra, S. M. & Reijman, M. 2013. Anxiety And Depressive Symptoms Before And After Total Hip And Knee Arthroplasty: A Prospective Multicentre Study. *Osteoarthritis & Cartilage,* 21**,** 1834-40.
89. Dumenci L, Perera RA, Keefe FJ, et al. Model-based pain and function outcome trajectory types for patients undergoing knee arthroplasty: a secondary analysis from a randomized clinical trial. *Osteoarthritis Cartilage*. Jun 2019;27(6):878-884. doi:10.1016/j.joca.2019.01.004
90. Dursteler C, Salazar Y, Rodriguez U, Pelfort X, Verdie LP. Conditioned pain modulation predicts persistent pain after knee replacement surgery. *Pain Rep*. Jan-Feb 2021;6(1):e910. doi:10.1097/PR9.0000000000000910
91. Dutka, J., Skowronek, M., Sosin, P. & Skowronek, P. 2011. Subvastus And Medial Parapatellar Approaches In Tka: Comparison Of Functional Results. *Orthopedics,* 34**,** 148.
92. Dutton, A. Q., Yeo, S. J., Yang, K. Y., Lo, N. N., Chia, K. U., Chong, H. C., Dutton, A. Q., Yeo, S.-J., Yang, K.-Y., Lo, N.-N., Chia, K.-U. & Chong, H.-C. 2008. Computer-Assisted Minimally Invasive Total Knee Arthroplasty Compared With Standard Total Knee Arthroplasty. A Prospective, Randomized Study. *Journal Of Bone & Joint Surgery, American Volume,* 90**,** 2-9.
93. Edwards, R. R., Haythornthwaite, J. A., Smith, M. T., Klick, B. & Katz, J. N. 2009. Catastrophizing And Depressive Symptoms As Prospective Predictors Of Outcomes Following Total Knee Replacement. *Pain Research & Management,* 14**,** 307-11.
94. Ellis, H. B., Howard, K. J., Khaleel, M. A. & Bucholz, R. 2012. Effect Of Psychopathology On Patient-Perceived Outcomes Of Total Knee Arthroplasty Within An Indigent Population. *Journal Of Bone And Joint Surgery - Series A,* 94**,** E84.1-E84.8.
95. Faller, H., Kirschner, S. & Konig, A. 2003. Psychological Distress Predicts Functional Outcomes At Three And Twelve Months After Total Knee Arthroplasty. *General Hospital Psychiatry,* 25**,** 372-373.
96. Farahini, H., Moghtadaei, M., Bagheri, A. & Akbarian, E. 2012. Factors Influencing Range Of Motion After Total Knee Arthroplasty. *Iranian Red Crescent Medical Journal,* 14**,** 417-21.
97. Farin, E., Glattacker, M. & Jackel, W. H. 2006. Predictors Of Rehabilitation Outcome In Patients After Total Hip And Total Knee Arthroplasty - A Multilevel Analysis. [German]. *Physikalische Medizin Rehabilitationsmedizin Kurortmedizin,* 16**,** 82-91.
98. Fernandez-Fairen, M., Hernández-Vaquero, D., Murcia, A., Torres, A. & Llopis, R. 2013. Trabecular Metal In Total Knee Arthroplasty Associated With Higher Knee Scores: A Randomized Controlled Trial. *Clinical Orthopaedics & Related Research,* 471**,** 3543-3553.
99. Ferreira AM, Salim R, Fogagnolo F, de Oliveira LFL, Riberto M, Kfuri M. The Value of a Standardized Knee Functional Assessment in Predicting the Outcomes of Total Knee Arthroplasty. *J Knee Surg*. Jan 28 2021;doi:10.1055/s-0040-1722321
100. Ferrer T, Hinarejos P, Goicoechea N, et al. Anxiety is the cause of the worse outcomes of allergic patients after total knee arthroplasty. *Knee Surg Sports Traumatol Arthrosc*. Oct 2020;28(10):3135-3141. doi:10.1007/s00167-019-05780-0
101. Filardo, G., Merli, G., Roffi, A., Marcacci, T., Berti Ceroni, F., Raboni, D., Bortolotti, B., Kon, E. & Marcacci, M. 2017. Kinesiophobia And Depression Affect Total Knee Arthroplasty Outcome In A Multivariate Analysis Of Psychological And Physical Factors On 200 Patients. *Knee Surgery, Sports Traumatology, Arthroscopy,* 25**,** 3417-3423.
102. Filbay, S. R. & Judge, A. 2017. Evaluating Patients' Expectations From A Novel Patientcentered Perspective Predicts Surgical Outcome And Dissatisfaction Following Knee Arthroplasty. *Osteoarthritis And Cartilage,* 25**,** S336-S337.
103. Filbay, S. R., Judge, A., Delmestri, A., Arden, N. K., Altman, D., Beard, D., Carr, A., Cooper, C., Culliford, D., Griffin, T., Javaid, K., Latham, J., Murray, D., Pinedo-Villanueva, R., Price, A. & Prieto-Alhambra, D. 2018. Evaluating Patients' Expectations From A Novel Patient-Centered Perspective Predicts Knee Arthroplasty Outcome. *Journal Of Arthroplasty,* 33**,** 2146-2152.E4.
104. Fitzpatrick, J., Badley, E. M., Rampersaud, Y. R., Power, J. D., Gandhi, R., Veillette, C., Mahomed, N. N., Davey, J. R., Syed, K. & Perruccio, A. V. 2017. The Influence Of Patient Characteristics On Pain Following Total Joint Arthroplasty For Osteoarthritis: Effect Modification By Sex And Age. *Osteoarthritis And Cartilage,* 25**,** S340.
105. Fitzsimmons, M., Carr, E., Woodhouse, L. & Bostick, G. P. 2018. Development And Persistence Of Suspected Neuropathic Pain After Total Knee Arthroplasty In Individuals With Osteoarthritis. *Pm R,* 10**,** 903-909.
106. Fleeton, G., Harmer, A. R., Nairn, L., Crosbie, J., March, L., Crawford, R., Van Der Esch, M. & Fransen, M. 2016. Self-Reported Knee Instability Before And After Total Knee Replacement Surgery. *Arthritis Care & Research,* 68**,** 463-71.
107. Foran, J. R., Mont, M. A., Etienne, G., Jones, L. C. & Hungerford, D. S. 2004. The Outcome Of Total Knee Arthroplasty In Obese Patients. *Journal Of Bone & Joint Surgery - American Volume,* 86**,** 1609-15.
108. Forsythe, M. E., Dunbar, M. J., Hennigar, A. W., Sullivan, M. J. L. & Gross, M. 2008. Prospective Relation Between Catastrophizing And Residual Pain Following Knee Arthroplasty: Two-Year Follow-Up. *Pain Research And Management,* 13**,** 335-341.
109. Franklin, P. D., Harrold, L. R., Li, W., Allison, J., Ayers, D. & Lewis, C. 2013. Important Predictors Of Patient-Reported Outcomes After Tkr And Thr Are Not Included In Risk Models Based On Administrative Data. *Arthritis And Rheumatism,* 10**,** S910.
110. Franklin, P. D., Li, W. & Ayers, D. C. 2008. The Chitranjan Ranawat Award: Functional Outcome After Total Knee Replacement Varies With Patient Attributes. *Clinical Orthopaedics & Related Research,* 466**,** 2597-604.
111. Furu, M., Ito, H., Nishikawa, T., Nankaku, M., Kuriyama, S., Ishikawa, M., Nakamura, S., Azukizawa, M., Hamamoto, Y. & Matsuda, S. 2016. Quadriceps Strength Affects Patient Satisfaction After Total Knee Arthroplasty. *Journal Of Orthopaedic Science,* 21**,** 38-43.
112. Gandhi, R., Davey, J. R. & Mahomed, N. 2009a. Patient Expectations Predict Greater Pain Relief With Joint Arthroplasty. *Journal Of Arthroplasty,* 24**,** 716-21.
113. Gandhi, R., Razak, F., Davey, J. R. & Mahomed, N. N. 2010a. Metabolic Syndrome And The Functional Outcomes Of Hip And Knee Arthroplasty. *Journal Of Rheumatology,* 37**,** 1917-22.
114. Gandhi, R., Razak, F., Davey, J. R. & Mahomed, N. N. 2010b. Metabolic Syndrome And The Functional Outcomes Of Hip And Knee Arthroplasty. *Journal Of Rheumatology,* 37**,** 1917-1922.
115. Gandhi, R., Razak, F., Davey, J. R., Rampersaud, Y. R. & Mahomed, N. N. 2010c. Effect Of Sex And Living Arrangement On The Timing And Outcome Of Joint Replacement Surgery. *Canadian Journal Of Surgery,* 53**,** 37-41.
116. Gandhi, R., Razak, F., Tso, P., Davey, J. R. & Mahomed, N. N. 2009b. Greater Perceived Helplessness In Osteoarthritis Predicts Outcome Of Joint Replacement Surgery. *Journal Of Rheumatology,* 36**,** 1507-11.
117. Gandhi, R., Tso, P., Davis, A. & Mahomed, N. N. 2009c. Outcomes Of Total Joint Arthroplasty In Academic Versus Community Hospitals. *Canadian Journal Of Surgery,* 52**,** 413-6.
118. Gandhi, R., Tsvetkov, D., Davey, J. R., Syed, K. A. & Mahomed, N. N. 2009d. Relationship Between Self-Reported And Performance-Based Tests In A Hip And Knee Joint Replacement Population. *Clinical Rheumatology,* 28**,** 253-257.
119. Gates, L. S., Bowen, C. J. & Arden, N. K. 2016. Can Clinical Foot And Ankle Assessments Improve The Prediction Of Patient Reported Outcomes In Knee Arthroplasty? *Osteoarthritis And Cartilage,* 1**,** S208.
120. Gates, L. S., Bowen, C. J., Sanchez-Santos, M. T., Delmestri, A. & Arden, N. K. 2017. Do Foot & Ankle Assessments Assist The Explanation Of 1 Year Knee Arthroplasty Outcomes? *Osteoarthritis & Cartilage,* 25**,** 892-898.
121. Gatha, N. M., Clarke, H. D., Fuchs, R., Scuderi, G. R. & Insall, J. N. 2004. Factors Affecting Postoperative Range Of Motion After Total Knee Arthroplasty. *The Journal Of Knee Surgery,* 17**,** 196-202.
122. Giesinger, J. M., Giesinger, K., Loth, F. L., Simpson, H. A., Howie, C. & Hamilton, D. F. 2016. Impact Of Obesity On Patient-Reported Outcomes After Total Knee Replacement. *Value In Health,* 19**,** A544.
123. Giordano R, Petersen KK, Santoro M, et al. Circulating long non-coding RNA signature in knee osteoarthritis patients with postoperative pain one-year after total knee replacement. *Scand J Pain*. Oct 26 2021;21(4):823-830. doi:10.1515/sjpain-2021-0069
124. Gonzalez Saenz De Tejada, M., Escobar, A., Bilbao, A., Herrera-Espineira, C., Garcia-Perez, L., Aizpuru, F. & Sarasqueta, C. 2014. A Prospective Study Of The Association Of Patient Expectations With Changes In Health-Related Quality Of Life Outcomes, Following Total Joint Replacement. *Bmc Musculoskeletal Disorders,* 15**,** 248.
125. Gouveia B, Fonseca S, Pozza DH, Xara D, Sa Rodrigues A. Relationship between Postoperative Pain and Sociocultural Level in Major Orthopedic Surgery. Adv Orthop. 2022;2022:7867719. Epub 20221011. doi: 10.1155/2022/7867719. PubMed PMID: 36267670; PubMed Central PMCID: PMCPMC9578872.Graves, C. M., Otero, J. E., Gao, Y., Goetz, D. D., Willenborg, M. D. & Callaghan, J. J. 2014. Patient Reported Allergies Are A Risk Factor For Poor Outcomes In Total Hip And Knee Arthroplasty. *Journal Of Arthroplasty,* 29**,** 147-9.
126. Graves, C. M., Otero, J. E., Gao, Y., Goetz, D. D., Willenborg, M. D. & Callaghan, J. J. 2014. Patient Reported Allergies Are A Risk Factor For Poor Outcomes In Total Hip And Knee Arthroplasty. *Journal Of Arthroplasty,* 29**,** 147-9.
127. Gray, A., Ramos, S., Howard, K., Bryson, B. & Ellis, H. B. 2017. The Pain Disability Questionnaire Pdq: Evaluating The Efficacy Of The Psychosocial And Functional Subscales For 12-Month Post-Treatment Outcomes After Total Knee Arthroplasty. *Current Orthopaedic Practice,* 28**,** 573-579.
128. Greco, C., Belfer, I., Vulakovich, K., Landsittel, D., Dai, F. & Chelly, J. 2017. Associations Of Psychosocial Factors With Chronic Postsurgical Pain Following Total Knee Arthroplasty. *Journal Of Pain,* 18**,** S76.
129. Greenidge, N., Davis, A. M., Hawker, G. A. & Badley, E. M. 2009. The Effect Of Pre-Operative Number Of Affected Joints On Pain And Function Following Total Joint Replacement For Oa: A Longitudinal Study. *Osteoarthritis And Cartilage,* 1**,** S194.
130. Grosu, I., Thienpont, E., France, M. N. & Lavand'homme, P. 2013. Persistent Pain After Total Knee Arthroplasty Tka: Incidence, Characteristics And Predictive Factors. *Regional Anesthesia And Pain Medicine. Conference: 38th Annual Asra Regional Anesthesia And Acute Pain Medicine Meeting,* 38.
131. Group, K. A. T. T., Johnston, L., Maclennan, G., Mccormack, K., Ramsay, C. & Walker, A. 2009. The Knee Arthroplasty Trial Kat Design Features, Baseline Characteristics, And Two-Year Functional Outcomes After Alternative Approaches To Knee Replacement. *Journal Of Bone & Joint Surgery - American Volume,* 91**,** 134-41.
132. Guimaraes-Pereira, L., Valdoleiros, I., Reis, P. & Abelha, F. 2016. Evaluating Persistent Postoperative Pain In One Tertiary Hospital: Incidence, Quality Of Life, Associated Factors, And Treatment. *Anesthesiology And Pain Medicine,* 6.
133. Gøthesen, O., Espehaug, B., Havelin, L. I., Petursson, G., Hallan, G., Strøm, E., Dyrhovden, G. & Furnes, O. 2014. Functional Outcome And Alignment In Computer-Assisted And Conventionally Operated Total Knee Replacements: A Multicentre Parallel-Group Randomised Controlled Trial. *Bone & Joint Journal,* 96**,** 609-618.
134. Ha, C. & Ha, H. 2006. Minimally Invasive Vs. Standard Total Knee Arthroplasty: A Prospective Randomized Comparison Study. *Journal Of The Korean Orthopaedic Association,* 41**,** 841-849.
135. Halket, A., Stratford, P. W., Kennedy, D. M. & Woodhouse, L. J. 2010. Using Hierarchical Linear Modeling To Explore Predictors Of Pain After Total Hip And Knee Arthroplasty As A Consequence Of Osteoarthritis. *Journal Of Arthroplasty,* 25**,** 254-62.
136. Hamilton, D. F., Burnett, R., Patton, J. T., Howie, C. R., Moran, M., Simpson, A. H. R. W. & Gaston, P. 2015. Implant Design Influences Patient Outcome After Total Knee Arthroplasty: A Prospective Double-Blind Randomised Controlled Trial. *Bone & Joint Journal,* 97**,** 64-70.
137. Hamilton, D. F., Loth, F. L., Giesinger, J. G., Giesinger, K., Macdonald, D. J., Simpson, H. & Howie, C. R. 2017. Impact Of Obesity On Patient-Reported Outcomes Following Total Knee Arthroplasty. *Journal Of Orthopaedic Research. Conference,* 35.
138. Hamilton DF, Shim J, Howie CR, Macfarlane GJ. Patients follow three distinct outcome trajectories following total knee arthroplasty. *Bone Joint J*. Jun 2021;103-B(6):1096-1102. doi:10.1302/0301-620X.103B6.BJJ-2020-1821.R1
139. Hanratty, B., Bennett, D., Thompson, N. & Beverland, D. 2011. A Randomised Controlled Trial Investigating The Effect Of Posterior Capsular Stripping On Knee Flexion And Range Of Motion In Patients Undergoing Primary Knee Arthroplasty. *The Knee,* 18**,** 474-479.
140. Hanusch, B. C., O'connor, D. B., Ions, P., Scott, A. & Gregg, P. J. 2014. Effects Of Psychological Distress And Perceptions Of Illness On Recovery From Total Knee Replacement. *Bone & Joint Journal,* 96**,** 210-6.
141. Harden, R. N., Bruehl, S., Stanos, S., Brander, V., Chung, O. Y., Saltz, S., Adams, A. & Stulberg, S. D. 2003. Prospective Examination Of Pain-Related And Psychological Predictors Of Crps-Like Phenomena Following Total Knee Arthroplasty: A Preliminary Study. *Pain,* 106**,** 393-400.
142. Hasegawa M, Tone S, Naito Y, Sudo A. Preoperative pain catastrophizing affects pain outcome after total knee arthroplasty. *J Orthop Sci*. Jul 3 2021;doi:10.1016/j.jos.2021.05.011
143. Hasegawa M, Tone S, Naito Y, Sudo A. Predicting outcomes after total knee arthroplasty using intraoperative knee kinematics measured by navigation. Mod Rheumatol. 2022;20:20. Epub 20221020. doi: 10.1093/mr/roac130. PubMed PMID: 36264180.
144. Hashimoto, S., Hatayama, K., Terauchi, M., Saito, K., Higuchi, H. & Chikuda, H. 2019. Preoperative Hand-Grip Strength Can Be A Predictor Of Stair Ascent And Descent Ability After Total Knee Arthroplasty In Female Patients. *Journal Of Orthopaedic Science.*
145. Hemert, W., Senden, R., Grimm, B., Linde, M., Lataster, A. & Heyligers, I. 2011. Early Functional Outcome After Subvastus Or Parapatellar Approach In Knee Arthroplasty Is Comparable. *Knee Surgery, Sports Traumatology, Arthroscopy,* 19**,** 943-951.
146. Hinarejos, P., Ferrer, T., Leal, J., Torres-Claramunt, R., Sanchez-Soler, J. & Monllau, J. C. 2016. Patient-Reported Allergies Cause Inferior Outcomes After Total Knee Arthroplasty. *Knee Surgery, Sports Traumatology, Arthroscopy,* 24**,** 3242-3246.
147. Hirschmann, M. T., Hoffmann, M., Krause, R., Jenabzadeh, R. A., Arnold, M. P. & Friederich, N. F. 2010. Anterolateral Approach With Tibial Tubercle Osteotomy Versus Standard Medial Approach For Primary Total Knee Arthroplasty: Does It Matter? *Bmc Musculoskeletal Disorders,* 11**,** 167.
148. Hirschmann, M. T., Testa, E., Amsler, F. & Friederich, N. F. 2013. The Unhappy Total Knee Arthroplasty Tka Patient: Higher Womac And Lower Kss In Depressed Patients Prior And After Tka. *Knee Surgery, Sports Traumatology, Arthroscopy,* 21**,** 2405-11.
149. Hitt, K., Pierce, T., Jauregui, J., Cherian, J., Elmallah, R., Leibowitz, E., Logan, S. & Mont, M. 2015. Use Of A Flexible Intramedullary Rod And Its Influence On Patient Satisfaction And Femoral Size In Total Knee Arthroplasty. *Journal Of Long-Term Effects Of Medical Implants,* 25**,** 201-208.
150. Hodges, A., Harmer, A. R., Dennis, S., Nairn, L., March, L., Crawford, R., Parker, D. & Fransen, M. 2018. Prevalence And Determinants Of Physical Activity And Sedentary Behaviour Before And Up To 12months After Total Knee Replacement: A Longitudinal Cohort Study. *Clinical Rehabilitation***,** 269215518769986.
151. Hodges, A., Harmer, A. R., Dennis, S., Nairn, L., March, L., Crawford, R., Parker, D. & Fransen, M. 2018. Prevalence And Determinants Of Physical Activity And Sedentary Behaviour Before And Up To 12months After Total Knee Replacement: A Longitudinal Cohort Study. *Clinical Rehabilitation***,** 269215518769986.
152. Hommel, H., Abdel, M. & Perka, C. 2017. Kinematic Femoral Alignment With Gap Balancing And Patient-Specific Instrumentation In Total Knee Arthroplasty: A Randomized Clinical Trial. *European Journal Of Orthopaedic Surgery & Traumatology : Orthopedie Traumatologie,* 27**,** 683-688.
153. Hofstede, S. N., Gademan, M. G. J., Stijnen, T., Nelissen, R. G. H. H., Marang-Van De Mheen, P. J., Bierma-Zeinstra, S. M. A., Van Dijk, M., Kaarsemaker, S., Van Kampen, P. M., Nolte, P. A., Poolman, R. W., Pronk, Y., Reijman, M., Stevens, M., Thomassen, B. J. W., Verdegaal, S. H. M. & Vliet Vlieland, T. P. M. 2018. The Influence Of Preoperative Determinants On Quality Of Life, Functioning And Pain After Total Knee And Hip Replacement: A Pooled Analysis Of Dutch Cohorts. *Bmc Musculoskeletal Disorders,* 19.
154. Hofstede, S. N., Gademan, M. G. J., Stijnen, T., Nelissen, R. G. H. H., Marang-Van De Mheen, P. J., Bierma-Zeinstra, S. M. A., Van Dijk, M., Kaarsemaker, S., Van Kampen, P. M., Nolte, P. A., Poolman, R. W., Pronk, Y., Reijman, M., Stevens, M., Thomassen, B. J. W., Verdegaal, S. H. M. & Vliet Vlieland, T. P. M. 2018. The Influence Of Preoperative Determinants On Quality Of Life, Functioning And Pain After Total Knee And Hip Replacement: A Pooled Analysis Of Dutch Cohorts. *Bmc Musculoskeletal Disorders,* 19.
155. Hooper, G. J., Rothwell, A. G., Hooper, N. M., Frampton, C., Hooper, G. J., Rothwell, A. G., Hooper, N. M. & Frampton, C. 2012. The Relationship Between The American Society Of Anesthesiologists Physical Rating And Outcome Following Total Hip And Knee Arthroplasty: An Analysis Of Thenew Zealand Joint Registry. *Journal Of Bone & Joint Surgery, American Volume,* 94**,** 1065-1070.
156. Hourlier, H. & Fennema, P. 2014. Intraoperative Fluoroscopy Improves Surgical Precision In Conventional Tka. *Knee Surgery, Sports Traumatology, Arthroscopy,* 22**,** 1619-1625.
157. Hovik, L. H., Winther, S. B., Foss, O. A. & Gjeilo, K. H. 2016. Preoperative Pain Catastrophizing And Postoperative Pain After Total Knee Arthroplasty: A Prospective Cohort Study With One Year Follow-Up. *Bmc Musculoskeletal Disorders,* 17**,** 214.
158. Hughes, A. J., Richards, J. M., Campbell, C. M., Haythornthwaite, J. A., Edwards, R. R. & Smith, M. T. 2018. Sleep-Pain Behaviors Predict Insomnia, Pain, And Physical Function Over One Year Following Total Knee Arthroplasty In Individuals With Knee Osteoarthritis. *Sleep,* 41**,** A338.
159. Hylkema, T. H., Stevens, M., Selzer, F., Amick, B. A., Katz, J. N. & Brouwer, S. 2019. Activity Impairment And Work Productivity Loss After Total Knee Arthroplasty: A Prospective Study. *Journal Of Arthroplasty.*
160. Haanstra, T. M., Tilbury, C., Kamper, S. J., Tordoir, R. L., Vliet Vlieland, T. P., Nelissen, R. G., Cuijpers, P., De Vet, H. C., Dekker, J., Knol, D. L. & Ostelo, R. W. 2015. Can Optimism, Pessimism, Hope, Treatment Credibility And Treatment Expectancy Be Distinguished In Patients Undergoing Total Hip And Total Knee Arthroplasty? *Plos One [Electronic Resource],* 10**,** E0133730.
161. Ingleshwar, A., Barbo, A., Landon, G. C., Siff, S. J., De Achaval, S. & Suarez-Almazor, M. E. 2013. Ethnic Variations At Time Of Surgery And During Follow-Up In Patients Undergoing Total Knee Arthroplasty. *Arthritis And Rheumatism,* 10**,** S353.
162. Jacobs, C. A., Christensen, C. P. & Karthikeyan, T. 2016a. Greater Medial Compartment Forces During Tka Associated With Improved Patient Satisfaction And Function. *Journal Of Orthopaedic Research. Conference,* 34.
163. Jacobs, C. A., Christensen, C. P. & Karthikeyan, T. 2016b. Persistent Postoperative Pain More Common For Total Knee Arthroplasty Patients With An Intact Anterior Cruciate Ligament At The Time Of Surgery. *Journal Of Orthopaedic Research. Conference,* 34.
164. Jain, D., Nguyen, L. L., Bendich, I., Nguyen, L. L., Lewis, C. G., Huddleston, J. I., Duwelius, P. J., Feeley, B. T. & Bozic, K. J. 2017. Higher Patient Expectations Predict Higher Patient-Reported Outcomes, But Not Satisfaction, In Total Knee Arthroplasty Patients: A Prospective Multicenter Study. *Journal Of Arthroplasty,* 32**,** S166-S170.
165. Jamsen, E., Vekama, L. & Puolakka, T. 2015. Self-Rated Health And The Functional Outcome Of Primary Knee Replacement In The Aged. *European Geriatric Medicine,* 1**,** S149.
166. Jarvenpaa, J., Kettunen, J., Kroger, H. & Miettinen, H. 2010a. Obesity May Impair The Early Outcome Of Total Knee Arthroplasty. *Scandinavian Journal Of Surgery: Sjs,* 99**,** 45-9.
167. Jarvenpaa, J., Kettunen, J., Kroger, H. & Miettinen, H. 2010b. Obesity May Impair The Early Outcome Of Total Knee Arthroplasty. A Prospective Study Of 100 Patients. *Scandinavian Journal Of Surgery,* 99**,** 45-49.
168. Jefferies, P., Coffey, L. & Gallagher, P. 2012. The Efficacy Of Psychosocial Factors In Predicting Pain And Functional Outcomes Following Knee Replacement Surgery. *Evidence-Based Nursing,* 15**,** 92-3.
169. Jiang, Y., Sanchez-Santos, M. T., Judge, A. D., Murray, D. W. & Arden, N. K. 2017. Predictors Of Patient-Reported Pain And Functional Outcomes Over 10 Years After Primary Total Knee Arthroplasty: A Prospective Cohort Study. *Journal Of Arthroplasty,* 32**,** 92-100.E2.
170. Jolles, B., Grzesiak, A., Eudier, A., Dejnabadi, H., Voracek, C., Pichonnaz, C., Aminian, K. & Martin, E. 2012. A Randomised Controlled Clinical Trial And Gait Analysis Of Fixed- And Mobile-Bearing Total Knee Replacements With A Five-Year Follow-Up. 94.
171. Jonbergen, H., Scholtes, V., Kampen, A. & Poolman, R. 2011. A Randomised, Controlled Trial Of Circumpatellar Electrocautery In Total Knee Replacement Without Patellar Resurfacing. *Journal Of Bone And Joint Surgery. British Volume,* 93**,** 1054-1059.
172. Jones, C. A., Cox, V., Jhangri, G. S. & Suarez-Almazor, M. E. 2012a. Delineating The Impact Of Obesity And Its Relationship On Recovery After Total Joint Arthroplasties. *Osteoarthritis & Cartilage,* 20**,** 511-8.
173. Jones, C. A., Jhangri, G. S. & Suarez-Almazor, M. E. 2012b. Factors Influencing Long-Term Recovery Of Total Knee Arthroplasty. *Arthritis And Rheumatism,* 10**,** S467-S468.
174. Jones, C. A., Voaklander, D. C. & Suarez-Alma, M. E. 2003. Determinants Of Function After Total Knee Arthroplasty. *Physical Therapy,* 83**,** 696-706.
175. Judge, A., Arden, N. K., Cooper, C., Kassim Javaid, M., Carr, A. J., Field, R. E. & Dieppe, P. A. 2012. Predictors Of Outcomes Of Total Knee Replacement Surgery. *Rheumatology,* 51**,** 1804-13.
176. Judge, A., Dieppe, P. A., Arden, N. K., Cooper, C., Carr, A., Javaid, K. & Field, R. 2010. Developing A Predictive Tool For Outcomes Of Total Knee Replacement: South West London Elective Orthopaedic Centre Database. *Rheumatology,* 1**,** I74.
177. Julie, K. C. S., Contrera, K., Speering, L., Miller, E. T., Pfefferle, K., Greene, K. & Delahanty, D. L. 2013. Using Established Predictors Of Post-Traumatic Stress To Explain Variations In Recovery Outcomes Among Orthopaedic Patients. *Psychosomatic Medicine,* 75**,** A-149.
178. Kahlenberg, C. A., Trivellas, M., Lee, Y. Y. & Padgett, D. E. 2018. Preoperative Valgus Alignment Does Not Predict Inferior Outcome Of Total Knee Arthroplasty. *Hss Journal,* 14**,** 50-54.
179. Kang, J. H., Hsieh, M. S. & Lin, H. C. 2010. Comparison Of Treatment Outcomes Following Total Knee Arthroplasty Among Patients With Rheumatoid Arthritis And Osteoarthritis: A Nationwide Population-Based Study. *Rheumatology,* 49**,** 1409-10.
180. Katakam A, Bragdon CR, Chen AF, Melnic CM, Bedair HS. Elevated Body Mass Index Is a Risk Factor for Failure to Achieve the Knee Disability and Osteoarthritis Outcome Score-Physical Function Short Form Minimal Clinically Important Difference Following Total Knee Arthroplasty. *J Arthroplasty*. May 2021;36(5):1626-1632. doi:10.1016/j.arth.2020.12.019
181. Katz, J. N., Wright, E. A., Lingard, E. A. & Losina, E. 2011. Association Between Severe Pain In The Early Months Following Total Knee Replacement And Functional Outcomes Over Five Year Follow-Up. *Osteoarthritis And Cartilage,* 1**,** S41-S42.
182. Keeney, B. J., Koenig, K. M., Paddock, N. G., Moschetti, W. E., Sparks, M. B. & Jevsevar, D. S. 2017. Do Aggregate Socioeconomic Status Factors Predict Outcomes For Total Knee Arthroplasty In A Rural Population? *Journal Of Arthroplasty,* 32**,** 3583-3590.
183. Kelly, M., Rumi, M., Kothari, M., Parentis, M., Bailey, K., Parrish, W. & Pellegrini, V. 2006. Comparison Of The Vastus-Splitting And Median Parapatellar Approaches For Primary Total Knee Arthroplasty: A Prospective, Randomized Study. *Journal Of Bone And Joint Surgery. American Volume,* 88**,** 715-720.
184. Kennedy, D. M., Stratford, P. W., Riddle, D. L., Hanna, S. E. & Gollish, J. D. 2008. Assessing Recovery And Establishing Prognosis Following Total Knee Arthroplasty. *Physical Therapy,* 88**,** 22-32.
185. Khanna, V. 2016. Association Between Preoperative Sarcopenia And Functional Outcome Of The Patients Following Total Knee Replacement. *Osteoporosis International,* 1**,** S300.
186. Kilicarslan, K., Yalcin, N., Cicek, H., Dogramaci, Y., Ugurlu, M., Ozkan, H. & Yildirim, H. 2011. The Effect Of Total Synovectomy In Total Knee Arthroplasty: A Prospective Randomized Controlled Study. *Knee Surgery, Sports Traumatology, Arthroscopy,* 19**,** 932-935.
187. Kim, S. H., Yoon, K. B., Yoon, D. M., Yoo, J. H. & Ahn, K. R. 2015. Influence Of Centrally Mediated Symptoms On Postoperative Pain In Osteoarthritis Patients Undergoing Total Knee Arthroplasty: A Prospective Observational Evaluation. *Pain Practice,* 15**,** E46-53.
188. Kim, T. K., Cho, H. J., Kang, Y. G., Kim, S. J. & Chang, C. B. 2009. Improved Early Clinical Outcomes Of Rp/Ps Mobile-Bearing Total Knee Arthroplasties. *Clinical Orthopaedics & Related Research,* 467**,** 2901-10.
189. Klasan A, Rice DA, Kluger MT, Borotkanics R, McNair PJ, Lewis GN, et al. A combination of high preoperative pain and low radiological grade of arthritis is associated with a greater intensity of persistent pain 12 months after total knee arthroplasty. Bone Joint J. 2022;104-B(11):1202-8. doi: 10.1302/0301-620X.104B11.BJJ-2022-0630.R1. PubMed PMID: 36317350.
190. Ko, Y., Lo, N. N., Yeo, S. J., Yang, K. Y., Yeo, W., Chong, H. C. & Thumboo, J. 2010. Determining The Optimal Timing For Total Knee Replacement. *Value In Health,* 13**,** A313.
191. Kurien, T., Arendt-Nielsen, L., Petersen, K. K., Graven-Nielsen, T. & Scammell, B. E. 2018. Preoperative Neuropathic Pain Like Symptoms And Central Pain Mechanisms In Knee Osteoarthritis Predicts Poor Outcome 6 Months After Total Knee Replacement Surgery. *Journal Of Pain,* 16**,** 16.
192. Kurien, T., Arendt-Nielsen, L., Petersen, K. K., Graven-Nielsen, T. & Scammell, B. E. 2018. Preoperative Neuropathic Pain Like Symptoms And Central Pain Mechanisms In Knee Osteoarthritis Predicts Poor Outcome 6 Months After Total Knee Replacement Surgery. *Journal Of Pain,* 16**,** 16.
193. Kurien T, Kerslake RW, Graven-Nielsen T, Arendt-Nielsen L, Auer DP, Edwards K, et al. Chronic postoperative pain after total knee arthroplasty: The potential contributions of synovitis, pain sensitization and pain catastrophizing-An explorative study. Eur J Pain. 2022;26(9):1979-89. Epub 20220819. doi: 10.1002/ejp.2018.
194. Lam, L. O., Swift, S. & Shakespeare, D. 2003. Fixed Flexion Deformity And Flexion After Knee Arthroplasty. What Happens In The First 12 Months After Surgery And Can A Poor Outcome Be Predicted? *Knee,* 10**,** 181-5.
195. Lamb, S. E. & Frost, H. 2003. Recovery Of Mobility After Knee Arthroplasty: Expected Rates And Influencing Factors. *Journal Of Arthroplasty,* 18**,** 575-582.
196. Lampe, F., Marques, C. J., Fiedler, F., Sufi-Siavach, A., Carita, A. I. & Matziolis, G. 2016. Patient-Specific And Intra-Operatively Modifiable Factors Assessed By Computer Navigation Predict Maximal Knee Flexion One Year After Tka. *Knee Surgery, Sports Traumatology, Arthroscopy,* 24**,** 3457-3465.
197. Lange, J., Disegna, S. T., Yang, W., Li, W. & Franklin, P. D. 2016. Identifying Preoperative Patient Characteristics That Correlate With Early Improvement Or Decline Following Total Knee Arthroplasty. *Journal Of Orthopaedic Research. Conference,* 34.
198. Larsen DB, Laursen M, Edwards RR, Simonsen O, Arendt-Nielsen L, Petersen KK. The Combination of Preoperative Pain, Conditioned Pain Modulation, and Pain Catastrophizing Predicts Postoperative Pain 12 Months After Total Knee Arthroplasty. *Pain Medicine*. 2021;22(7):1583-1590.
199. Laskow T, Zhu J, Buta B, et al. Risk Factors for Non-Resilient Outcomes in Older Adults after Total Knee Replacement in the FORCE-TJR Cohort. *J Gerontol A Biol Sci Med Sci*. Sep 4 2021;04doi:10.1093/gerona/glab257
200. Lebleu J, Poilvache H, Mahaudens P, De Ridder R, Detrembleur C. Predicting physical activity recovery after hip and knee arthroplasty? A longitudinal cohort study. *Brazilian Journal of Physical Therapy*. 2019;25(1):30-39.
201. Ledin, H., , P. & , L. 2012. Tourniquet Use In Total Knee Replacement Does Not Improve Fixation, But Appears To Reduce Final Range Of Motion. *Acta Orthopaedica,* 83**,** 499-503.
202. Lee, A., Chan, S. K. C., Samy, W., Chiu, C. H. & Gin, T. 2015. Effect Of Hypovitaminosis D On Postoperative Pain Outcomes And Short-Term Health-Related Quality Of Life After Knee Arthroplasty. *Medicine United States,* 94**,** E1812.
203. Leung, Y. Y., Chakraborty, B., Lim, Z., Yeo, S., Lo, N., Tan, M., Wong, S., Chong, H., Yeo, W., Wylde, V., Dieppe, P. & Thumboo, J. 2017. Preoperative Pain Sensitization And Total Knee Replacement Outcome. *Osteoarthritis And Cartilage,* 25**,** S372.
204. Leung, Y. Y., Lim, Z., Fan, Q., Wylde, V., Xiong, S., Yeo, S. J., Lo, N. N., Chong, H. C., Yeo, W., Tan, M. H., Chakraborty, B., Bak-Siew Wong, S. & Thumboo, J. 2019. Pre-Operative Pressure Pain Thresholds Do Not Meaningfully Explain Satisfaction Or Improvement In Pain After Knee Replacement: A Cohort Study. *Osteoarthritis And Cartilage,* 27**,** 49-58.
205. Li, W., Harrold, L. R., Allison, J., Lewis, C., Bowen, T., Franklin, P. D. & Ayers, D. 2013. Does Functional Gain And Pain Relief After Tkr And Thr Differ By Patient Obese Status? *Arthritis And Rheumatism,* 10**,** S909-S910.
206. Liebs, T. R., Herzberg, W., Roth-Kroeger, A. M., Rüther, W. & Hassenpflug, J. 2011. Women Recover Faster Than Men After Standard Knee Arthroplasty. *Clinical Orthopaedics & Related Research,* 469**,** 2855-2865.
207. Lindberg, M. F., Miaskowski, C., Rustøen, T., Rosseland, L. A., Cooper, B. A. & Lerdal, A. 2016. Factors That Can Predict Pain With Walking, 12 Months After Total Knee Arthroplasty. *Acta Orthopaedica,* 87**,** 600-606.
208. Lindberg MF, Schweitz TU, Aamodt A, Gay C, Lerdal A. High pre- and postoperative symptom burden in non-responders to total knee arthroplasty. *PLoS One*. 2020;15(5):e0233347. doi:10.1371/journal.pone.0233347
209. Lindner, M., Nosseir, O., Keller-Pliessnig, A., Teigelack, P., Teufel, M. & Tagay, S. 2018. Psychosocial Predictors For Outcome After Total Joint Arthroplasty: A Prospective Comparison Of Hip And Knee Arthroplasty. *Bmc Musculoskeletal Disorders,* 19**,** 159.
210. Lingard, E. A., Katz, J. N., Wright, E. A. & Sledge, C. B. 2004. Predicting The Outcome Of Total Knee Arthroplasty. *Journal Of Bone And Joint Surgery - Series A,* 86**,** 2179-2186.
211. Liu K, Yang D, Zan P, et al. Preoperative low scores of Life Satisfaction Rating predicts poor outcomes after total knee arthroplasty: a prospective observational study. *Journal of Orthopaedic Surgery*. 2020;15(1):145.
212. Lizaur-Utrilla, A., Sanz-Reig, J. & Trigueros-Rentero, M. 2012. Greater Satisfaction In Older Patients With A Mobile-Bearing Compared With Fixed-Bearing Total Knee Arthroplasty. *Journal Of Arthroplasty,* 27**,** 207-212.
213. Lungu, E., Desmeules, F., Dionne, C. E., Belzile, E. L. & Vendittoli, P. A. 2014. Prediction Of Poor Outcomes Six Months Following Total Knee Arthroplasty In Patients Awaiting Surgery. *Bmc Musculoskeletal Disorders,* 15**,** 299.
214. Lustig, S., Scholes, C. J., Stegeman, T. J., Oussedik, S., Coolican, M. R. J. & Parker, D. A. 2012. Sagittal Placement Of The Femoral Component In Total Knee Arthroplasty Predicts Knee Flexion Contracture At One-Year Follow-Up. *International Orthopaedics,* 36**,** 1835-1839.
215. Lutzner, C., Kirschner, S. & Lutzner, J. 2014. Patient Activity After Tka Depends On Patient-Specific Parameters. *Clinical Orthopaedics & Related Research,* 472**,** 3933-40.
216. Lützner, J., Hartmann, A., Lützner, C. & Kirschner, S. 2014. Is Range Of Motion After Cruciate-Retaining Total Knee Arthroplasty Influenced By Prosthesis Design? A Prospective Randomized Trial. *Journal Of Arthroplasty,* 29**,** 961-965.
217. Macaulay, W., Kim, A. D., Geller, J. A., Nellans, K. W., Morrison, T. A., Choi, J. K., Nyce, J. & Coyle, R. 2010. The Effect Of Hypovitaminosis D Among Primary Knee Arthroplasty Patients. *Osteoporosis International,* 1**,** S105-S106.
218. Maculé, F., Sastre, S., Lasurt, S., Sala, P., Segur, J. & Mallofré, C. 2005. Hoffa's Fat Pad Resection In Total Knee Arthroplasty. *Acta Orthopaedica Belgica,* 71**,** 714-717.
219. Maffulli, G., Bridgman, S. & Maffulli, N. 2011. Total Knee Arthoplasty: Better Short-Term Results After Subvastus Approach. *Knee Surgery, Sports Traumatology, Arthroscopy,* 19**,** 1047; Author Reply 1048.
220. Magaldi, R. J., Staff, I., Stovall, A. E., Stohler, S. A. & Lewis, C. G. 2019. Impact Of Resilience On Outcomes Of Total Knee Arthroplasty. *The Journal Of Arthroplasty,* 34**,** 2620-2623. E1.
221. Mahomed, N. N., Liang, M. H., Cook, E. F., Daltroy, L. H., Fortin, P. R., Fossel, A. H. & Katz, J. N. 2002. The Importance Of Patient Expectations In Predicting Functional Outcomes After Total Joint Arthroplasty. *Journal Of Rheumatology,* 29**,** 1273-9.
222. Mahoney, O. M., Kinsey, T. L., D'errico, T. J., Shen, J., Mahoney, O. M., Kinsey, T. L., D'errico, T. J. & Shen, J. 2012. The John Insall Award: No Functional Advantage Of A Mobile Bearing Posterior Stabilized Tka. *Clinical Orthopaedics & Related Research,* 470**,** 33-44.
223. Malviya, A., Lingard, E. A., Weir, D. J. & Deehan, D. J. 2009. Predicting Range Of Movement After Knee Replacement: The Importance Of Posterior Condylar Offset And Tibial Slope. *Knee Surgery, Sports Traumatology, Arthroscopy,* 17**,** 491-8.
224. Martinez, V., Fletcher, D., Bouhassira, D., Sessler, D. I. & Chauvin, M. 2007. The Evolution Of Primary Hyperalgesia In Orthopedic Surgery: Quantitative Sensory Testing And Clinical Evaluation Before And After Total Knee Arthroplasty. *Anesthesia & Analgesia,* 105**,** 815-21.
225. Mat, E. I. M., Sharifudin, M., Shokri, A. & Ab, R. S. 2016. Preoperative Physiotherapy And Short-Term Functional Outcomes Of Primary Total Knee Arthroplasty. *Singapore Medical Journal,* 57**,** 138-143.
226. Maus, U., Marques, C. J., Scheunemann, D., Lampe, F., Lazovic, D., Hommel, H., Vogel, D., Haunschild, M. & Pfitzner, T. 2017. No Improvement In Reducing Outliers In Coronal Axis Alignment With Patient-Specific Instrumentation. *Knee Surgery, Sports Traumatology, Arthroscopy,* 25**,** 25.
227. Mawarikado Y, Inagaki Y, Fujii T, Imagita H, Fukumoto T, Kubo T, et al. Preoperative Factors Affecting Patient-reported Outcome Measures for Total Knee Arthroplasty. Prog Rehabil Med. 2022;7:20220049. Epub 20220915. doi: 10.2490/prm.20220049.
228. Maxwell, J., Niu, J., Singh, J. A., Nevitt, M. C., Law, L. F. & Felson, D. 2013. The Influence Of The Contralateral Knee Prior To Knee Arthroplasty On Post-Arthroplasty Function: The Multicenter Osteoarthritis Study. *Journal Of Bone & Joint Surgery - American Volume,* 95**,** 989-93.
229. Meessen J, Fiocco M, Leichtenberg CS, Vliet Vlieland TPM, Slagboom PE, Nelissen R. Frailty Questionnaire Is Not a Strong Prognostic Factor for Functional Outcomes in Hip or Knee Arthroplasty Patients. *Geriatric Orthopaedic Surgery & Rehabilitation*. 2018;10:2151459318808164.
230. Mehta, S. & Lotke, P. A. 2007. Impact Of Surgeon Handedness And Laterality On Outcomes Of Total Knee Arthroplasties: Should Right-Handed Surgeons Do Only Right Tkas? *American Journal Of Orthopedics Belle Mead, N.J.,* 36**,** 530-533.
231. Mehta, S., Palaganas, M., Perruccio, A. V. & Davis, A. M. 2014. Do Women Have Poorer Outcomes Following Total Knee Replacement For Osteoarthritis? *Osteoarthritis And Cartilage,* 1**,** S15-S16.
232. Mehta, S. P., Perruccio, A. V., Palaganas, M. & Davis, A. M. 2015. Do Women Have Poorer Outcomes Following Total Knee Replacement? *Osteoarthritis & Cartilage,* 23**,** 1476-82.
233. Meijerink, H. J., Brokelman, R. B. G., Van Loon, C. J. M., Van Kampen, A. & De Waal Malefijt, M. C. 2009. Surgeon's Expectations Do Not Predict The Outcome Of A Total Knee Arthroplasty. *Archives Of Orthopaedic And Trauma Surgery,* 129**,** 1361-1365.
234. Mercurio M, Gasparini G, Carbone EA, Galasso O, Segura-Garcia C. Personality traits predict residual pain after total hip and knee arthroplasty. *International Orthopaedics*. 2020;44(7):1263-1270.
235. Metsna, V., Vorobjov, S., Lepik, K. & Märtson, A. 2014. Anterior Knee Pain Following Total Knee Replacement Correlates With The Oarsi Score Of The Cartilage Of The Patella. *Acta Orthopaedica,* 85**,** 427-432.
236. Miozzari, H. H., Sagawa Junior, Y., Hoffmeyer, P., Suva, D., Armand, S. & Turcot, K. 2013. Gait Analysis And Patients Outcome After Tka Comparing Dependent Vs. Independent Bone Cut Technique: A Preliminary Study. *Swiss Medical Weekly,* 198**,** 2s-3s.
237. Mittal, R., Ko, V., Adie, S., Naylor, J., Dave, J., Dave, C., Harris, I. A., Hackett, D., Ngo, D. & Dietsch, S. 2012. Tourniquet Application Only During Cement Fixation In Total Knee Arthroplasty: A Double-Blind, Randomized Controlled Trial. *Anz Journal Of Surgery,* 82**,** 428-33.
238. Mizner, R. L., Petterson, S. C., Stevens, J. E., Axe, M. J. & Snyder-Mackler, L. 2005. Preoperative Quadriceps Strength Predicts Functional Ability One Year After Total Knee Arthroplasty. *Journal Of Rheumatology,* 32**,** 1533-9.
239. Moghtadaei M, Yeganeh A, Hosseinzadeh N, et al. The Impact of Depression, Personality, and Mental Health on Outcomes of Total Knee Arthroplasty. *Clinics in Orthopedic Surgery*. 2020;12(4):456-463.
240. Mohammad HR, Gooberman-Hill R, Delmestri A, Broomfield J, Patel R, Huber J, et al. Risk factors associated with poor pain outcomes following primary knee replacement surgery: Analysis of data from the clinical practice research datalink, hospital episode statistics and patient reported outcomes as part of the STAR research programme. PLoS ONE. 2021;16(12):e0261850. Epub 20211231. doi: 10.1371/journal.pone.0261850.
241. Molt, M. & Toksvig-Larsen, S. 2014. Similar Early Migration When Comparing Cr And Ps In Triathlon? Tka: A Prospective Randomised Rsa Trial. *The Knee,* 21**,** 949-954.
242. Morze, C. J., Johnson, N. R., Williams, G., Moroney, M., Lamberton, T. & Mcauliffe, M. 2013. Knee Pain During The First Three Months After Unilateral Total Knee Arthroplasty: A Multi-Centre Prospective Cohort Study. *Journal Of Arthroplasty,* 28**,** 1565-70.
243. Motwani, P., Jariwala, A. & Valentine, N. 2013. Does Navigation Total Knee Replacement Really Make A Difference? *Journal Of Musculoskeletal Research,* 16.
244. Nandi, M., Cornelius, M., Campbell, C., Smith, M., Haythornthwaite, J., Wright, J., Edwards, R. & Strichartz, G. 2016. Sex Differences In Pain And Functioning Among Total Knee Arthroplasty Patients. *Journal Of Pain,* 1**,** S1-S2.
245. Nankaku, M., Ito, H., Furu, M., Kuriyama, S., Nakamura, S., Ikeguchi, R. & Matsuda, S. 2018. Preoperative Factors Related To The Ambulatory Status At 1 Year After Total Knee Arthroplasty. *Disability & Rehabilitation,* 40**,** 1929-1932.
246. Nankaku, M., Ito, H., Furu, M., Kuriyama, S., Nakamura, S., Ikeguchi, R. & Matsuda, S. 2018. Preoperative Factors Related To The Ambulatory Status At 1 Year After Total Knee Arthroplasty. *Disability & Rehabilitation,* 40**,** 1929-1932.
247. Navarro Collado, M. J., Peiro, S., Trenor Gomis, C., Ruiz Jareno, L., Perez Igualada, A. & Guerola Soler, N. 2000. [Factors Related To Functional Outcomes And Quality Of Life After Knee Arthroplasty]. *Medicina Clinica,* 114**,** 250-4.
248. Naylor, J. M., Harmer, A. R. & Heard, R. C. 2008. Severe Other Joint Disease And Obesity Independently Influence Recovery After Joint Replacement Surgery: An Observational Study. *Australian Journal Of Physiotherapy,* 54**,** 57-64.
249. Neogi, T., Niu, J., Frey-Law, L., Arendt-Nielsen, L., Singh, J., Scholz, J. & Woolf, C. 2010. Role Of Central Sensitization In Persistent Pain Post-Knee Replacement: The Most Study. *Arthritis And Rheumatism,* 10**,** 1385.
250. Neuburger, J., Hutchings, A., Black, N. & Van Der Meulen, J. H. 2013. Socioeconomic Differences In Patient-Reported Outcomes After A Hip Or Knee Replacement In The English National Health Service. *Journal Of Public Health,* 35**,** 115-24.
251. Neuprez, A., Neuprez, A. H., Kaux, J. F., Kurth, W., Daniel, C., Thirion, T., Huskin, J. P., Gillet, P., Bruyere, O. & Reginster, J. Y. 2018. Early Clinically Relevant Improvement In Quality Of Life And Clinical Outcomes 1 Year Postsurgery In Patients With Knee And Hip Joint Arthroplasties. *Cartilage,* 9**,** 127-139.
252. Neuprez, A., Neuprez, A. H., Kaux, J. F., Kurth, W., Daniel, C., Thirion, T., Huskin, J. P., Gillet, P., Bruyere, O. & Reginster, J. Y. 2018. Early Clinically Relevant Improvement In Quality Of Life And Clinical Outcomes 1 Year Postsurgery In Patients With Knee And Hip Joint Arthroplasties. *Cartilage,* 9**,** 127-139
253. Nielsen, C. S., Nebergall, A., Huddleston, J., Kallemose, T., Malchau, H. & Troelsen, A. 2018. Medial Overhang Of The Tibial Component Is Associated With Higher Risk Of Inferior Knee Injury And Osteoarthritis Outcome Score Pain After Knee Replacement. *Journal Of Arthroplasty,* 33**,** 1394-1398.
254. Nielsen, C. S., Nebergall, A., Huddleston, J., Kallemose, T., Malchau, H. & Troelsen, A. 2018. Medial Overhang Of The Tibial Component Is Associated With Higher Risk Of Inferior Knee Injury And Osteoarthritis Outcome Score Pain After Knee Replacement. *Journal Of Arthroplasty,* 33**,** 1394-1398.
255. Niki, Y., Takeda, Y., Harato, K. & Suda, Y. 2015. Factors Affecting The Achievement Of Japanese-Style Deep Knee Flexion After Total Knee Arthroplasty Using Posterior-Stabilized Prosthesis With High-Flex Knee Design. *Journal Of Orthopaedic Science,* 20**,** 1012-8.
256. Noiseux, N., Callaghan, J., Clark, C., Zimmerman, M., Sluka, K. & Rakel, B. 2014. Preoperative Predictors Of Pain Following Total Knee Arthroplasty. *Journal Of Arthroplasty,* 29**,** 1383-1387.
257. Nuñez, M., Lozano, L., Nuñez, E., Segur, J. M. & Sastre, S. 2011. Factors Influencing Health-Related Quality Of Life After Tka In Patients Who Are Obese. *Clinical Orthopaedics & Related Research,* 469**,** 1148-1153.
258. Nwankwo VC, Jiranek WA, Green CL, Allen KD, George SZ, Bettger JP. Resilience and pain catastrophizing among patients with total knee arthroplasty: a cohort study to examine psychological constructs as predictors of post-operative outcomes. *Health & Quality of Life Outcomes*. 2021;19(1):136.
259. Oatis, C. A., Li, W., Rosal, M., Ayers, D. & Franklin, P. D. 2012. Associations Between Body Mass Index And Physical Activity Following Total Knee Replacement. *Arthritis And Rheumatism,* 10**,** S1123-S1124.
260. Oberbek, J. & Synder, M. 2015. Impact Of Body Mass Index Bmi On Early Outcomes Of Total Knee Arthroplasty. *Ortopedia Traumatologia Rehabilitacja,* 17**,** 127-34.
261. Oka T, Ono R, Tsuboi Y, et al. Effect of preoperative sedentary behavior on clinical recovery after total knee arthroplasty: a prospective cohort study. *Clinical Rheumatology*. 2019;39(3):891-898.
262. Okamoto, S., Okazaki, K., Mitsuyasu, H., Matsuda, S., Mizu-Uchi, H., Hamai, S., Tashiro, Y. & Iwamoto, Y. 2014. Extension Gap Needs More Than 1-Mm Laxity After Implantation To Avoid Post-Operative Flexion Contracture In Total Knee Arthroplasty. *Knee Surgery, Sports Traumatology, Arthroscopy,* 22**,** 3174-80.
263. Otero, J. E., Graves, C. M., Gao, Y., Olson, T. S., Dickinson, C. C., Chalus, R. J., Vittetoe, D. A., Goetz, D. D. & Callaghan, J. J. 2016. Patient-Reported Allergies Predict Worse Outcomes After Hip And Knee Arthroplasty: Results From A Prospective Cohort Study. *Journal Of Arthroplasty,* 31**,** 2746-2749.
264. Ozdemir, M., Demirkale, I., Sesen, H., Taskesen, A., Okkaoglu, M. C. & Altay, M. 2017. Affective Temperament Does Not Influence Satisfaction After Total Knee Arthroplasty. *Medicine,* 96**,** E6852.
265. Page, M. G., Katz, J., Curtis, K., Fuss, S., Cohen, N., Escobar, E. M. R. & Clarke, H. 2014. Not All Post-Operative Pain Is Alike: A Pain Trajectory Analysis In Total Knee Arthroplasty Patients. *Pain Research And Management,* 19**,** E90.
266. Page, M. G., Katz, J., Romero Escobar, E. M., Lutzky-Cohen, N., Curtis, K., Fuss, S. & Clarke, H. A. 2015. Distinguishing Problematic From Nonproblematic Postsurgical Pain: A Pain Trajectory Analysis After Total Knee Arthroplasty. *Pain,* 156**,** 460-468.
267. Palanne R, Rantasalo M, Vakkuri A, Olkkola KT, Vahlberg T, Skants N. Fat tissue is a poor predictor of 1 year outcomes after total knee arthroplasty: A secondary analysis of a randomized clinical trial. Scand J Surg. 2022:14574969221139722. Epub 20221212. doi: 10.1177/14574969221139722.
268. Pan, X., Wang, J., Lin, Z., Dai, W. & Shi, Z. 2019. Depression And Anxiety Are Risk Factors For Postoperative Pain-Related Symptoms And Complications In Patients Undergoing Primary Total Knee Arthroplasty In The United States. *Journal Of Arthroplasty.*
269. Papakostidou, I., Dailiana, Z. H., Papapolychroniou, T., Liaropoulos, L., Zintzaras, E., Karachalios, T. S. & Malizos, K. N. 2012. Factors Affecting The Quality Of Life After Total Knee Arthroplasties: A Prospective Study. *Bmc Musculoskeletal Disorders,* 13**,** 116.
270. Parsley, B. S., Bertolusso, R., Harrington, M., Brekke, A. & Noble, P. C. 2010. Influence Of Gender On Age Of Treatment With Tka And Functional Outcome. *Clinical Orthopaedics & Related Research,* 468**,** 1759-64.
271. Paxton, E. W., Torres, A., Love, R. M., Barber, T. C., Sheth, D. S. & Inacio, M. C. S. 2016. Total Joint Replacement: A Multiple Risk Factor Analysis Of Physical Activity Level 1–2 Years Postoperatively. *Acta Orthopaedica,* 87**,** 44-49.
272. Pereira, D. L., Meleiro, H. L., Correia, I. A. & Fonseca, S. 2016. [Pain After Major Elective Orthopedic Surgery Of The Lower Limb And Type Of Anesthesia: Does It Matter?]. *Revista Brasileira De Anestesiologia,* 66**,** 628-636.
273. Perruccio, A. V., Badley, E. M., Hogg-Johnson, S. & Davis, A. M. 2010a. Characterizing Self-Rated Health During A Period Of Changing Health Status. *Social Science & Medicine,* 71**,** 1636-43.
274. Perruccio, A. V., Badley, E. M., Hogg-Johnson, S. & Davis, A. M. 2010b. Characterizing Self-Rated Health During A Period Of Changing Health Status. *Social Science & Medicine,* 71**,** 1636-1643.
275. Perruccio, A. V., Davis, A. M., Hogg-Johnson, S. & Badley, E. M. 2011a. Importance Of Self-Rated Health And Mental Well-Being In Predicting Health Outcomes Following Total Joint Replacement Surgery For Osteoarthritis. *Arthritis Care & Research,* 63**,** 973-81.
276. Perruccio, A. V., Power, J. D., Badley, E. M., Gandhi, R., Mahomed, N. N. & Davis, A. M. 2011b. Outcomes Following Total Knee Replacement Surgery For Oa: There Is More Than Just The Knee To Consider. *Osteoarthritis And Cartilage,* 1**,** S22.
277. Petersen, K. K., Simonsen, O., Laursen, M. B. & Arendt-Nielsen, L. 2017. The Role Of Preoperative Radiological Severity, Sensory Testing, And Temporal Summation On Chronic Postoperative Pain Following Total Knee Arthroplasty. *Clinical Journal Of Pain.,* 16.
278. Pinsornsak, P., Naratrikun, K. & Chumchuen, S. 2014. The Effect Of Infrapatellar Fat Pad Excision On Complications After Minimally Invasive Tka: A Randomized Controlled Trial. 472.
279. Pinto, P., Mcintyre, T., Ferrero, R., Almeida, A. & Araujo-Soares, V. 2014. Risk Factors For Moderate And Severe Persistent Pain In Patients Undergoing Total Knee And Hip Arthroplasty: A Prospective Predictive Study. *Pain Practice,* 1**,** 94-95.
280. Pinto, P. R., Mcintyre, T., Ferrero, R., Almeida, A. & Araujo-Soares, V. 2013. Risk Factors For Moderate And Severe Persistent Pain In Patients Undergoing Total Knee And Hip Arthroplasty: A Prospective Predictive Study. *Plos One [Electronic Resource],* 8**,** E73917.
281. Polkowski, I. G. G., Ruh, E. L., Barrack, T. N., Nunley, R. M. & Barrack, R. L. 2013. Is Pain And Dissatisfaction After Tka Related To Early-Grade Preoperative Osteoarthritis? Knee. *Clinical Orthopaedics And Related Research,* 471**,** 162-168.
282. Pont, C. P., Ortiz, V. A. D. C., Pastor, B. S., Cano, L. G., Mesquida, M. E. P., Cobo, E. P., Marques, M. C. & González, M. B. 2011. Predictive Factors Of The Functional Level After Total Knee Arthroplasty. *Rehabilitacion,* 45**,** 240-246.
283. Pua, Y., Ong, P., Clark, R., Lee, A., Tan, J. & Bryant, A. 2012. A Prediction Model For Fear-Induced Activity Limitation After Total Knee Arthroplasty: A Prospective Cohort Study. *Proceedings Of Singapore Healthcare,* 21**,** S354.
284. Pua, Y. H., Ong, P. H., Chong, H. C., Yeo, W., Tan, C. & Lo, N. N. 2013. Knee Extension Range Of Motion And Self-Report Physical Function In Total Knee Arthroplasty: Mediating Effects Of Knee Extensor Strength. *Bmc Musculoskeletal Disorders,* 14**,** 33.
285. Pua, Y. H., Seah, F. J., Clark, R. A., Lian-Li Poon, C., Tan, J. W. & Chong, H. C. 2017. Factors Associated With Gait Speed Recovery After Total Knee Arthroplasty: A Longitudinal Study. *Semin Arthritis Rheum,* 46**,** 544-551.
286. Pua, Y. H., Seah, F. J., Seet, F. J., Tan, J. W., Liaw, J. S. & Chong, H. C. 2015. Sex Differences And Impact Of Body Mass Index On The Time Course Of Knee Range Of Motion, Knee Strength, And Gait Speed After Total Knee Arthroplasty. *Arthritis Care & Research,* 67**,** 1397-405.
287. Pua, Y.H., P., Jie-Ting Seah, F., Clark, R. A., Lian-Li Poon, C., Wei-Ming Tan, J., Hwei-Chi, C., Pua, Y.-H., Seah, F. J.-T., Poon, C. L.-L., Tan, J. W.-M. & Chong, H.-C. 2016. Development Of A Prediction Model To Estimate The Risk Of Walking Limitations In Patients With Total Knee Arthroplasty. *Journal Of Rheumatology,* 43**,** 419-426.
288. Quintana, J. M., Escobar, A., Arostegui, I., Bilbao, A., Azkarate, J., Goenaga, J. I. & Arenaza, J. C. 2006. Health-Related Quality Of Life And Appropriateness Of Knee Or Hip Joint Replacement. *Archives Of Internal Medicine,* 166**,** 220-6.
289. Radmer, S., Andresen, R. & Sparmann, M. 2006. Simultaneous Bilateral Total Knee Arthroplasty In Patients With Rheumatoid Arthritis. *Zeitschrift Fur Orthopadie Und Ihre Grenzgebiete,* 144**,** 472-476.
290. Rajamaki, T., Jamsen, E., Puolakka, P., Nevalainen, P. & Moilanen, T. 2015. Diabetes Is Associated With Persistent Pain After Hip And Knee Replacement. 86.
291. Rakel, B., Noiseux, N., Zimmerman, B., Callaghan, J., Clark, C. & Sluka, K. 2013. Predictors Of Pain Following Total Knee Arthroplasty. *Journal Of Pain,* 1**,** S15.
292. Ramaesh, R., Jenkins, P., Lane, J. V., Knight, S., Macdonald, D. & Howie, C. 2014. Personality, Function And Satisfaction In Patients Undergoing Total Hip Or Knee Replacement. *Journal Of Orthopaedic Science,* 19**,** 275-81.
293. Razmjou, H., Boljanovic, D., Wright, S., Murnaghan, J. & Holtby, R. 2015. Association Between Neuropathic Pain And Reported Disability After Total Knee Arthroplasty. *Physiotherapy Canada,* 67**,** 311-318.
294. Reid, M. J., Booth, G., Khan, R. J. & Janes, G. 2014. Patellar Eversion During Total Knee Replacement: A Prospective, Randomized Trial. *Journal Of Bone & Joint Surgery - American Volume,* 96**,** 207-13.
295. Rice, D., Kluger, M., Mcnair, P., Lewis, G., Somogyi, A., Borotkanics, R., Barratt, D. & Walker, M. 2018. Persistent Postoperative Pain After Total Knee Arthroplasty: A Prospective Cohort Study Of Potential Risk Factors. *British Journal Of Anaesthesia,* 121**,** 804.
296. Richards, J., Pejsa, M., Hand, M., Cornelius, M., Campbell, C., Haythornthwaite, J., Edwards, R. & Smith, M. 2016. Psychometric Evaluation And Predictive Validity Of The Sleep And Pain Behaviors Survey In Knee Osteoarthritis Patients Undergoing Total Knee Replacement. *Journal Of Pain,* 1**,** S17.
297. Riddle, D. L. 2018. Prevalence And Predictors Of Symptom Resolution And Functional Restoration In The Index Knee After Knee Arthroplasty: A Longitudinal Study. *Archives Of Physical Medicine & Rehabilitation,* 99**,** 887-892.
298. Riddle, D. L., Perera, R. A., Jiranek, W. A. & Dumenci, L. 2015. Using Surgical Appropriateness Criteria To Examine Outcomes Of Total Knee Arthroplasty In A United States Sample. *Arthritis Care & Research,* 67**,** 349-357.
299. Riddle DL, Slover J, Keefe FJ, Ang D, Dumenci L, Perera RA. Racial differences in pain and function following knee arthroplasty: a secondary analysis from a multicenter randomized clinical trial. *Osteoarthritis and cartilage*. 2020;28:S431‐S432. doi:10.1016/j.joca.2020.02.670
300. Riddle, D. L., Wade, J. B., Jiranek, W. A. & Kong, X. 2009. Preoperative Pain Catastrophizing Predicts Pain Outcome Following Knee Arthroplasty. *Osteoarthritis And Cartilage,* 1**,** S156.
301. Rosen, A. S., Neville, L., Pulido, P. A., Patil, S., Walker, R. H. & Cop, S. N. 2013. Outcome And Range Of Motion Using A High-Flexion Cruciate-Retaining Tka. *Orthopedics,* 36**,** E1198-E1202.
302. Russell, R. D., Huo, M. H., De Jong, L. & Jones, R. E. 2014. Preoperative Flexion Does Not Influence Postoperative Flexion After Rotating-Platform Total Knee Arthroplasty. *Knee Surgery, Sports Traumatology, Arthroscopy,* 22**,** 1644-8.
303. Sakellariou, V. I., Poultsides, L. A., Yan, M. A., Bae, J., Liu, S., Sculco, T. P. & Ma, Y. 2016. Risk Assessment For Chronic Pain And Patient Satisfaction After Total Knee Arthroplasty. *Orthopedics,* 39**,** 55-62.
304. Salazar, Y., Rodriguez, U., Dursteler, C., Puig, L., Montes, A. & Escolano, F. 2013. Painful Knee Prosthesis: Relationship Between Endogenous Analgesia And Persistent Post-Surgical Pain: Preliminary Data. *European Journal Of Anaesthesiology,* 51**,** 205-206.
305. Sanchez-Santos, M. T., Garriga, C., Judge, A., Batra, R. N., Price, A. J., Liddle, A. D., Javaid, M. K., Cooper, C., Murray, D. W. & Arden, N. K. 2018. Development And Validation Of A Clinical Prediction Model For Patient-Reported Pain And Function After Primary Total Knee Replacement Surgery. *Scientific Reports,* 8**,** 3381.
306. Sanchez-Santos, M. T., Judge, A., Batra, R. N., Murray, D., Price, A., Liddle, A. D., Javaid, M. K., Cooper, C. & Arden, N. K. 2014. A Clinical Tool For The Prediction Of Patient-Reported Outcomes After Knee Replacement Surgery. *Osteoarthritis And Cartilage,* 1**,** S412.
307. Schaumburger, J., Lechler, P., Riedt, S., Springorum, H. R., Rath, B., Baier, C., Kock, F. X., Grifka, J. & Handel, M. 2012. [Patient Satisfaction And Muscle Torque After Total Knee Replacement In Dependence On Body Mass Index]. [German]. *Zeitschrift Fur Orthopadie Und Unfallchirurgie,* 150**,** 641-647.
308. Schwartz, I., Kandel, L., Sajina, A., Litinezki, D., Herman, A. & Mattan, Y. 2012. Balance Is An Important Predictive Factor For Quality Of Life And Function After Primary Total Knee Replacement. *Journal Of Bone & Joint Surgery - British Volume,* 94**,** 782-786.
309. Scott, C. E., Bugler, K. E., Clement, N. D., Macdonald, D., Howie, C. R. & Biant, L. C. 2012. Patient Expectations Of Arthroplasty Of The Hip And Knee. *Journal Of Bone & Joint Surgery - British Volume,* 94**,** 974-81.
310. Scott, C. E. H., Howie, C. R., Macdonald, D. & Biant, L. C. 2010. Predicting Dissatisfaction Following Total Knee Replacement: A Prospective Study Of 1217 Patients. *Journal Of Bone And Joint Surgery - Series B,* 92**,** 1253-1258.
311. Seol, J. H., Seon, J. K. & Song, E. K. 2016. Comparison Of Postoperative Complications And Clinical Outcomes Between Simultaneous And Staged Bilateral Total Knee Arthroplasty. *Journal Of Orthopaedic Science,* 21**,** 766-769.
312. Sharma S, Kumar V, Sood M, Malhotra R. Effect of Preoperative Modifiable Psychological and Behavioural Factors on Early Outcome Following Total Knee Arthroplasty in an Indian Population. *Indian Journal of Orthopaedics*. 2021;55(4):939-947.
313. Shim, J., Mclernon, D. J., Hamilton, D., Simpson, H. A., Beasley, M. & Macfarlane, G. J. 2018. Development Of A Clinical Risk Score For Pain And Function Following Total Knee Arthroplasty: Results From The Trio Study. *Rheumatol Adv Pract,* 2**,** Rky021.
314. Sinclair ST, Klika AK, Jin Y, Higuera CA, Piuzzi NS, on behalf of the Cleveland Clinic OMEAG. The Impact of Surgeon Variability on Patient-Reported Outcome Measures, Length of Stay, Discharge Disposition, and 90-Day Readmission in TKA. *J Bone Joint Surg Am*. Nov 16 2022;104(22):2016-2025.
315. Singh, G., Han, F., Kaki, R. R., Shen, L. & Nathan, S. S. 2015. Does Limited Tourniquet Usage In Primary Total Knee Arthroplasty Result In Better Functional Outcomes? *Annals Of The Academy Of Medicine, Singapore,* 44**,** 302-306.
316. Siviero P, Marseglia A, Biz C, et al. Quality of life outcomes in patients undergoing knee replacement surgery: longitudinal findings from the QPro-Gin study. *BMC Musculoskelet Disord*. Jul 4 2020;21(1):436. doi:10.1186/s12891-020-03456-2
317. Slevin, O., Amsler, F. & Hirschmann, M. T. 2017. No Correlation Between Coronal Alignment Of Total Knee Arthroplasty And Clinical Outcomes: A Prospective Clinical Study Using 3d-Ct. *Knee Surgery, Sports Traumatology, Arthroscopy,* 25**,** 3892-3900.
318. Smith, A. J., Lloyd, D. G. & Wood, D. J. 2006. A Kinematic And Kinetic Analysis Of Walking After Total Knee Arthroplasty With And Without Patellar Resurfacing. *Clinical Biomechanics,* 21**,** 379-86.
319. Smith JRA, Mathews JA, Osborne L, Bakewell Z, Williams JL. Why do patients not kneel after total knee replacement? Is neuropathic pain a contributing factor? Observational Study. *Knee*. Mar 2019;26(2):427-434. doi:10.1016/j.knee.2018.12.009
320. Smith JRA, Mathews JA, Osborne L, Bakewell Z, Williams JL. Why do patients not kneel after total knee replacement? Is neuropathic pain a contributing factor? Observational Study. *Knee*. Mar 2019;26(2):427-434. doi:10.1016/j.knee.2018.12.009
321. Smith, J. W., Marcus, R. L., Peters, C. L., Pelt, C. E., Tracy, B. L. & Lastayo, P. C. 2014. Muscle Force Steadiness In Older Adults Before And After Total Knee Arthroplasty. *Journal Of Arthroplasty,* 29**,** 1143-8.
322. Smith, J. W., Martins, T. B., Gopez, E., Johnson, T., Hill, H. R. & Rosenberg, T. D. 2012. Significance Of C-Reactive Protein In Osteoarthritis And Total Knee Arthroplasty Outcomes. *Therapeutic Advances In Musculoskeletal Disease,* 4**,** 315-25.
323. Soni, A., Arden, N. K., Price, A. J., Tracey, I. & Javaid, M. 2016. Neuropathic Pain As A Predictor Of Short And Long-Term Outcome Following Knee Replacement Surgery. *Osteoarthritis And Cartilage,* 1**,** S420-S421.
324. Soni, A., Gwylim, S., Arden, N., Tracey, I., Price, A. & Javaid, M. 2014. Pre-Operative Experimental Thermal Sensitivity Predicts Oxford Knee Score 1-Year Post-Operatively: A Preliminary Study. *Osteoarthritis And Cartilage,* 1**,** S414-S415.
325. Stickles, B., Phillips, L., Brox, W. T., Owens, B. & Lanzer, W. L. 2001. Defining The Relationship Between Obesity And Total Joint Arthroplasty. *Obesity Research,* 9**,** 219-23.
326. Stone, O. D., Duckworth, A. D., Curran, D. P., Ballantyne, J. A. & Brenkel, I. J. 2017. Severe Arthritis Predicts Greater Improvements In Function Following Total Knee Arthroplasty. *Knee Surgery, Sports Traumatology, Arthroscopy,* 25**,** 2573-2579.
327. Stratford, P. W., Kennedy, D. M. & Robarts, S. F. 2010. Modelling Knee Range Of Motion Post Arthroplasty: Clinical Applications. *Physiotherapy Canada,* 62**,** 378-87.
328. Street, B. D., Adkin, A. & Gage, W. 2018. Reported Balance Confidence And Movement Reinvestment Of Younger Knee Replacement Patients Are More Like Younger Healthy Individuals, Than Older Patients. *Gait & Posture,* 61**,** 130-134.
329. Sun, J. Y. 2019. Presurgical Comorbidities as Risk Factors For Chronic Postsurgical Pain Following Total Knee Replacement: More Details, More Significance. *Clinical Journal of Pain,* 35**,** 725.
330. Sugawara, Y., Ishijima, M., Kurosawa, H., Shimura, Y., Kaneko, H., Liu, L., Futami, I., Iwase, Y. & Kaneko, K. 2017. Preoperative Timed Single Leg Standing Time Is Associated With The Postoperative Activity Of Daily Living In Aged Disabled Patients With End-Stage Knee Osteoarthritis At Six-Months After Undergoing Total Knee Arthroplasty. *Modern Rheumatology,* 27**,** 326-331.
331. Sveikata, T., Porvaneckas, N., Kanopa, P., Molyte, A., Klimas, D., Uvarovas, V. & Venalis, A. 2017. Age, Sex, Body Mass Index, Education, And Social Support Influence Functional Results After Total Knee Arthroplasty. *Geriatric Orthopaedic Surgery & Rehabilitation,* 8**,** 71-77.
332. Tabutin, J., Banon, F., Catonne, Y., Grobost, J., Tessier, J. L. & Tillie, B. 2005. Should We Resurface The Patella In Total Knee Replacement? Experience With The Nex Gen Prothesis. *Knee Surgery, Sports Traumatology, Arthroscopy,* 13**,** 534-8.
333. Tan, S. C., Chan, Y. H., Chong, H. C., Chin, P. L., Yew, A., Chia, S. L., Tay, D., Lo, N. N. & Yeo, S. J. 2014. Association Of Surgeon Factors With Outcome Scores After Total Knee Arthroplasty. *Journal Of Orthopaedic Surgery,* 22**,** 378-82.
334. Taniguchi M, Sawano S, Kugo M, Maegawa S, Kawasaki T, Ichihashi N. Physical Activity Promotes Gait Improvement in Patients With Total Knee Arthroplasty. *J Arthroplasty* 2016; **31**: 984-8.
335. Tchetina EV, Glemba KE, Markova GA, et al. Development of Postoperative Pain in Patients with End-Stage Knee Osteoarthritis Is Associated with Upregulation of Genes Related to Extracellular Matrix Degradation, Inflammation, and Apoptosis Measured in the Peripheral Blood before Knee Surgery. *Life (Basel)*. Sep 30 2020;10(10):30. doi:10.3390/life10100224
336. Thomazeau, J., Rouquette, A., Martinez, V., Rabuel, C., Prince, N., Laplanche, J. L., Nizard, R., Bergmann, J. F., Perrot, S. & Lloret-Linares, C. 2016. Predictive Factors Of Chronic Post-Surgical Pain At 6 Months Following Knee Replacement: Influence Of Postoperative Pain Trajectory And Genetics. *Pain Physician,* 19**,** E729-41.
337. Tilbury, C., Holtslag, M. J., Tordoir, R. L., Leichtenberg, C. S., Verdegaal, S. H., Kroon, H. M., Fiocco, M., Nelissen, R. G. & Vliet Vlieland, T. P. 2016. Outcome Of Total Hip Arthroplasty, But Not Of Total Knee Arthroplasty, Is Related To The Preoperative Radiographic Severity Of Osteoarthritis. A Prospective Cohort Study Of 573 Patients. *Acta Orthopaedica,* 87**,** 67-71.
338. Tilbury, C., Haanstra, T. M., Verdegaal, S. H. M., Nelissen, R., De Vet, H. C. W., Vliet Vlieland, T. P. M. & Ostelo, R. W. 2018. Patients' Pre-Operative General And Specific Outcome Expectations Predict Postoperative Pain And Function After Total Knee And Total Hip Arthroplasties. *Scand J Pain,* 18**,** 457-466.
339. Toguchi K, Nakajima A, Akatsu Y, et al. Predicting clinical outcomes after total knee arthroplasty from preoperative radiographic factors of the knee osteoarthritis. *BMC Musculoskelet Disord*. Jan 6 2020;21(1):9. doi:10.1186/s12891-019-3029-7
340. Tolk JJ, Waarsing JEH, Janssen RPA, van Steenbergen LN, Bierma-Zeinstra SMA, Reijman M. Development of Preoperative Prediction Models for Pain and Functional Outcome After Total Knee Arthroplasty Using The Dutch Arthroplasty Register Data. *J Arthroplasty*. Mar 2020;35(3):690-698 e2. doi:10.1016/j.arth.2019.10.010
341. Trace, R. 2006. Morbidly Obese Tka Patients Fare Poorly, Have Less Function, More Complications. *Orthopedics Today,* 26**,** 8-8.
342. Twiggs, J. G., Wakelin, E. A., Fritsch, B. A., Liu, D. W., Solomon, M. I., Parker, D. A., Klasan, A. & Miles, B. P. 2019. Clinical And Statistical Validation Of A Probabilistic Prediction Tool Of Total Knee Arthroplasty Outcome. *Journal Of Arthroplasty.*
343. Utrillas-Compaired, A., De La Torre-Escuredo, B. J., Tebar-Martinez, A. J. & Asunsolo-Del Barco, A. 2014. Does Preoperative Psychologic Distress Influence Pain, Function, And Quality Of Life After Tka? *Clinical Orthopaedics & Related Research,* 472**,** 2457-65.
344. Vaegter, H. B., Handberg, G., Emmeluth, C. & Graven-Nielsen, T. 2017. Preoperative Hypoalgesia After Cold Pressor Test And Aerobic Exercise Is Associated With Pain Relief 6 Months After Total Knee Replacement. *Clinical Journal Of Pain,* 33**,** 475-484.
345. Van Den Akker-Scheek, I., Stevens, M., Groothoff, J. W., Bulstra, S. K. & Zijlstra, W. 2007. Preoperative Or Postoperative Self-Efficacy: Which Is A Better Predictor Of Outcome After Total Hip Or Knee Arthroplasty? *Patient Education & Counseling,* 66**,** 92-9.
346. Van Hamersveld, K. T., Marang-Van De Mheen, P. J., Nelissen, R. & Toksvig-Larsen, S. 2018. Migration Of All-Polyethylene Compared With Metal-Backed Tibial Components In Cemented Total Knee Arthroplasty. *Acta Orthopaedica***,** 1-6.
347. Van Hamersveld, K. T., Marang-Van De Mheen, P. J., Nelissen, R. & Toksvig-Larsen, S. 2018. Migration Of All-Polyethylene Compared With Metal-Backed Tibial Components In Cemented Total Knee Arthroplasty. *Acta Orthopaedica***,** 1-6.
348. Van Hamersveld, K. T., Marang-Van De Mheen, P. J., Nelissen, R. & Toksvig-Larsen, S. 2018. Migration Of All-Polyethylene Compared With Metal-Backed Tibial Components In Cemented Total Knee Arthroplasty. *Acta Orthopaedica***,** 1-6.
349. van Loon C, Baas N, Huey V, Lesko J, Meermans G, Vergroesen D. Early outcomes and predictors of patient satisfaction after TKA: a prospective study of 200 cases with a contemporary cemented rotating platform implant design. *J Exp Orthop*. Apr 17 2021;8(1):30. doi:10.1186/s40634-021-00347-w
350. Van Onsem, S., Verstraete, M., Dhont, S., Zwaenepoel, B., Van Der Straeten, C. & Victor, J. 2018. Improved Walking Distance And Range Of Motion Predict Patient Satisfaction After Tka. *Knee Surgery, Sports Traumatology, Arthroscopy,* 8**,** 08.
351. Vekama, L., Puolakka, T., Honkasalo, M., Huhtala, H., Moilanen, T. & Jämsen, E. 2015. Functional Gain Following Knee Replacement In Patients Aged 75 And Older: A Prospective Follow-Up Study. *Aging Clinical & Experimental Research,* 27**,** 865-876.
352. Vela, J., Kjaer Petersen, K., Arendt-Nielsen, L., Meyer Andersen, M. & Simonsen, O. 2017. Preoperative Synovitis In Knee Osteoarthritis Is Predictive For Pain 1 Year After Total Knee Arthroplasty. *Scandinavian Journal Of Pain,* 16**,** 184.
353. Vila MR, Todorovic MS, Tang C, et al. Cognitive flexibility and persistent post-surgical pain: the FLEXCAPP prospective observational study. *Br J Anaesth*. May 2020;124(5):614-622. doi:10.1016/j.bja.2020.02.002
354. Vina, E. R., Hannon, M. J. & Kwoh, C. K. 2014. Improvement Following Total Knee Replacement Tkr Surgery: Exploring Preoperative Symptoms And Change In Preoperative Symptoms. *Arthritis And Rheumatology,* 10**,** S1297.
355. Vina, E. R., Hannon, M. J. & Kwoh, C. K. 2016. Improvement Following Total Knee Replacement Surgery: Exploring Preoperative Symptoms And Change In Preoperative Symptoms. *Seminars In Arthritis & Rheumatism,* 45**,** 547-55.
356. Vogel, M., Riediger, C., Krippl, M., Frommer, J., Lohmann, C. & Illiger, S. 2019. Negative Affect, Type D Personality, Quality Of Life, And Dysfunctional Outcomes Of Total Knee Arthroplasty. *Pain Res Manag,* 2019**,** 6393101.
357. Wada, O., Nagai, K., Hiyama, Y., Nitta, S., Maruno, H. & Mizuno, K. 2016. Diabetes Is A Risk Factor For Restricted Range Of Motion And Poor Clinical Outcome After Total Knee Arthroplasty. *Journal Of Arthroplasty,* 31**,** 1933-7.
358. Walker, J., Campbell, C., Pejsa, M., Hand, M., Haythornthwaite, J., Edwards, R., Khanuja, P., Sterling, R., Cornelius, M. & Smith, M. 2015. Pre-And Post-Operative Sleep Is Associated With Postoperative Pain And Functioning Following Total Knee Replacements. *Journal Of Pain,* 1**,** S63.
359. Wenjun, L., Ayers, D. C., Lewis, C. G., Bowen, T. R., Allison, J. J., Franklin, P. D. & Li, W. 2017. Functional Gain And Pain Relief After Total Joint Replacement According To Obesity Status. *Journal Of Bone & Joint Surgery, American Volume,* 99**,** 1183-1189.
360. Widmer, B. J., Scholes, C. J., Lustig, S., Conrad, L., Oussedik, S. I. & Parker, D. A. 2013. Intraoperative Computer Navigation Parameters Are Poor Predictors Of Function 1 Year After Total Knee Arthroplasty. *Journal Of Arthroplasty,* 28**,** 56-61.
361. Williams, D. P., Price, A. J., Beard, D. J., Hadfield, S. G., Arden, N. K., Murray, D. W. & Field, R. E. 2013. The Effects Of Age On Patient-Reported Outcome Measures In Total Knee Replacements. *Bone & Joint Journal,* 95**,** 38-44.
362. Winters, J. D., Christiansen, C. L. & Stevens-Lapsley, J. E. 2014. Preliminary Investigation Of Rate Of Torque Development Deficits Following Total Knee Arthroplasty. *Knee,* 21**,** 382-6.
363. Wohlrab, D., Ditl, J., Herrschelmann, R., Schietsch, U., Hein, W. & Hube, R. 2005. [Does The Nexgen Lps Flex Mobile Knee Prosthesis Offer Advantages Compared To The Nexgen Lps?--A Comparison Of Clinical And Radiological Results]. *Zeitschrift Fur Orthopadie Und Ihre Grenzgebiete,* 143**,** 567-72.
364. Wollmerstedt, N., Glatzel, M., Kirschner, S., Schneider, J., Faller, H. & König, A. 2006. Comparative Analysis Of Patient-Centered Outcome Of Total Hip And Knee Arthroplasty. *Zeitschrift Fur Orthopadie Und Ihre Grenzgebiete,* 144**,** 464-471.
365. Woo, Y., Lee, H. & Kim, J. 2006. Effectiveness Of Patellar Replacementin Total Knee Arthroplasty: Randomized, Prospective Study Using Simultaneous Bilateral Total Knee Arthroplasty. *Journal Of The Korean Orthopaedic Association,* 41**,** 675-679.
366. Wood TJ, Gazendam AM, Kabali CB, Hamilton Arthroplasty G. Postoperative Outcomes Following Total Hip and Knee Arthroplasty in Patients with Pain Catastrophizing, Anxiety, or Depression. *J Arthroplasty*. Jun 2021;36(6):1908-1914. doi:10.1016/j.arth.2021.02.018
367. Wright, D., Hoang, M., Sofine, A., Silva, J. P. & Schwarzkopf, R. 2017. Pain Catastrophizing As A Predictor For Postoperative Pain And Opiate Consumption In Total Joint Arthroplasty Patients. *Archives Of Orthopaedic And Trauma Surgery,* 137**,** 1623-1629.
368. Wylde, V., Palmer, S., Learmonth, I. D. & Dieppe, P. 2013. The Association Between Pre-Operative Pain Sensitisation And Chronic Pain After Knee Replacement: An Exploratory Study. *Osteoarthritis & Cartilage,* 21**,** 1253-6.
369. Wylde, V., Sayers, A., Lenguerrand, E., Gooberman-Hill, R., Pyke, M., Beswick, A. D., Dieppe, P. & Blom, A. W. 2015. Preoperative Widespread Pain Sensitization And Chronic Pain After Hip And Knee Replacement: A Cohort Analysis. *Pain,* 156**,** 47-54.
370. Wylde, V., Trela-Larsen, L., Whitehouse, M. R. & Blom, A. W. 2017. Preoperative Psychosocial Risk Factors For Poor Outcomes At 1 And 5 Years After Total Knee Replacement: A Cohort Study Of 266 Patients. *Acta Orthopaedica,* 88**,** 530-536.
371. Xu J, Twiggs J, Parker D, Negus J. The Association Between Anxiety, Depression, and Locus of Control With Patient Outcomes Following Total Knee Arthroplasty. *J Arthroplasty*. Mar 2020;35(3):720-724. doi:10.1016/j.arth.2019.10.022
372. Yakobov, E., Stanish, W., Tanzer, M., Dunbar, M., Richardson, G. & Sullivan, M. J. L. 2018. The Prognostic Value Of Pain Catastrophizing In Health-Related Quality Of Life Judgments After Total Knee Arthroplasty. *Health & Quality Of Life Outcomes,* 16**,** 126.
373. Yap YYW, Edwards KL, Soutakbar H, Fernandes GS, Scammell BE. Oxford knee score 1 year after TKR for osteoarthritis with reference to a normative population: What can patients expect? *Osteoarthritis and Cartilage Open*. June 2021;3(2)doi:10.1016/j.ocarto.2021.100143
374. Yau, W. P., Chiu, K. Y., Tang, W. M. & Ng, T. P. 2005. Residual Posterior Femoral Condyle Osteophyte Affects The Flexion Range After Total Knee Replacement. *International Orthopaedics,* 29**,** 375-9.
375. Young, S., Walker, M., Bayan, A., Briant-Evans, T., Pavlou, P., Farrington, B., Young, S. W. & Walker, M. L. 2017. The Chitranjan S. Ranawat Award : No Difference In 2-Year Functional Outcomes Using Kinematic Versus Mechanical Alignment In Tka: A Randomized Controlled Clinical Trial. *Clinical Orthopaedics & Related Research,* 475**,** 9-20.
376. Young-Shand KL, Dunbar MJ, Laende EK, Mills Flemming JE, Astephen Wilson JL. Early Identification of Patient Satisfaction Two Years After Total Knee Arthroplasty. *Journal of Arthroplasty*. 36(7):2473-2479.
377. Zeni, J. A., Jr. & Snyder-Mackler, L. 2010. Preoperative Predictors Of Persistent Impairments During Stair Ascent And Descent After Total Knee Arthroplasty. *Journal Of Bone & Joint Surgery - American Volume,* 92**,** 1130
