# Supplementary material for: Factors correlated with pain after total knee arthroplasty: A systematic review and meta-analysis
Source: PLoS One. 2023 Mar 24;18(3):e0283446. doi: 10.1371/journal.pone.0283446 (PMC10038299; doi:10.1371/journal.pone.0283446)
Supplement: Attachment — Submitted filename: Response to reviewers..docx [file pone.0283446.s011.docx]

**Response to reviewers’ comments on PONE-D-22-27666: Factors correlated with pain after total knee arthroplasty: a systematic review and meta-analysis.**

Dear Editor and Reviewers,

Thank you for your valuable feedback on our manuscript. We have carefully reviewed your comments and suggestions and have made the necessary revisions to improve the manuscript. Our responses to each comment and resulting changes are provided below.

Editor comments to author:

1. Please ensure that your manuscript meets PLOS ONE's style requirements, including those for file naming.

**Response:** We confirm that the manuscript meets PLOS ONE`s style requirements, including those for file naming.

2. Please ensure that your search is up to date and any relevant studies published since October 2021 are included in your systematic review.

**Response:** The literature search is updated and include articles published until February 6^th,^ 2023, and have included an additional three papers in our systematic review. New statistical analyses have also been performed to reflect these changes.

3. If you wish to make changes to your Data Availability statement, please describe these changes in your cover letter.

**Response:** We have submitted the STATA data files, and described this in the cover letter

We hope that these revisions demonstrate our commitment to producing a high-quality manuscript and look forward to your decision. Please do not hesitate to contact us if you have any further comments or concerns.

Best regards,

Unni Olsen
